# Enhanced Efficacy of Aurora Kinase Inhibitors in G2/M Checkpoint Deficient *TP53* Mutant Uterine Carcinomas Is Linked to the Summation of LKB1–AKT–p53 Interactions

**DOI:** 10.3390/cancers13092195

**Published:** 2021-05-03

**Authors:** Katherine N. Lynch, Joyce F. Liu, Nikolas Kesten, Kin-Hoe Chow, Aniket Shetty, Ruiyang He, Mosammat Faria Afreen, Liping Yuan, Ursula A. Matulonis, Whitfield B. Growdon, Michael G. Muto, Neil S. Horowitz, Colleen M. Feltmate, Michael J. Worley, Ross S. Berkowitz, Christopher P. Crum, Bo R. Rueda, Sarah J. Hill

**Affiliations:** 1 Department of Medical Oncology, Dana-Farber Cancer Institute, Boston, MA 02215, USA; katherinen_lynch@dfci.harvard.edu (K.N.L.); Joyce_liu@dfci.harvard.edu (J.F.L.); nikolas_kesten@dfci.harvard.edu (N.K.); m.fariafreen@gmail.com (M.F.A.); Ursula_Matulonis@dfci.harvard.edu (U.A.M.); 2 Division of Molecular and Cellular Oncology, Dana-Farber Cancer Institute, Boston, MA 02215, USA; 3 Department of Medicine, Brigham and Women’s Hospital, Boston, MA 02115, USA; 4 Center for Functional Cancer Epigenetics, Dana-Farber Cancer Institute, Boston, MA 02215, USA; 5 Center for Patient Derived Models, Dana-Farber Cancer Institute, Boston, MA 02215, USA; Kin-Hoe_Chow@dfci.harvard.edu (K.-H.C.); Aniket_Shetty@dfci.harvard.edu (A.S.); 6 Department of Biochemistry, Cambridge University, Cambridge CB2 1QW, UK; ruiyang_he@dfci.harvard.edu; 7 Department of Pathology, Brigham and Women’s Hospital, Boston, MA 02115, USA; Lyuan1@bwh.harvard.edu (L.Y.); ccrum@bwh.harvard.edu (C.P.C.); 8 Division of Gynecologic Oncology, Department of Obstetrics and Gynecology, Massachusetts General Hospital, Boston, MA 02114, USA; wgrowdon@mgh.harvard.edu (W.B.G.); brueda@mgh.harvard.edu (B.R.R.); 9 Vincent Center for Reproductive Biology, Department of Obstetrics and Gynecology, Massachusetts General Hospital, Boston, MA 02114, USA; 10 Obstetrics, Gynecology and Reproductive Biology, Harvard Medical School, Boston, MA 02115, USA; mmuto@bwh.harvard.edu (M.G.M.); nhorowitz@mgh.harvard.edu (N.S.H.); cfeltmate@bwh.harvard.edu (C.M.F.); mjworley@partners.org (M.J.W.J.); ross_berkowitz@dfci.harvard.edu (R.S.B.); 11 Division of Gynecologic Oncology, Department of Obstetrics and Gynecology, Brigham and Women’s Hospital, Boston, MA 02115, USA; 12 Department of Pathology, Harvard Medical School, Boston, MA 02115, USA

**Keywords:** Aurora kinase, LKB1, uterine cancer, p53, G2/M cell cycle checkpoint

## Abstract

**Simple Summary:**

Cancers arising from the lining of the uterus, endometrial cancers, are the most common gynecologic malignancy in the United States. Once endometrial cancer escapes the uterus and grows in distant locations, there are limited therapeutic options. The most aggressive and lethal endometrial cancers carry alterations in the protein p53, which is a critical guardian of many cellular functions. The role of these p53 alterations in endometrial cancer is not well understood. The goal of this work was to use p53 altered models of endometrial cancer to understand which, if any, therapeutically targetable vulnerabilities these p53 alterations may confer in endometrial cancer. Here we show that many of these p53 altered cells have problems with cell division which can be targeted with novel single and combination therapies. These discoveries may lead to relevant new therapies for difficult to treat advanced stage endometrial cancers.

**Abstract:**

Uterine carcinoma (UC) is the most common gynecologic malignancy in the United States. *TP53* mutant UCs cause a disproportionate number of deaths due to limited therapies for these tumors and the lack of mechanistic understanding of their fundamental vulnerabilities. Here we sought to understand the functional and therapeutic relevance of *TP53* mutations in UC. We functionally profiled targetable *TP53* dependent DNA damage repair and cell cycle control pathways in a panel of *TP53* mutant UC cell lines and patient-derived organoids. There were no consistent defects in DNA damage repair pathways. Rather, most models demonstrated dependence on defective G2/M cell cycle checkpoints and subsequent upregulation of Aurora kinase-LKB1-p53-AKT signaling in the setting of baseline mitotic defects. This combination makes them sensitive to Aurora kinase inhibition. Resistant lines demonstrated an intact G2/M checkpoint, and combining Aurora kinase and WEE1 inhibitors, which then push these cells through mitosis with Aurora kinase inhibitor-induced spindle defects, led to apoptosis in these cases. Overall, this work presents Aurora kinase inhibitors alone or in combination with WEE1 inhibitors as relevant mechanism driven therapies for *TP53* mutant UCs. Context specific functional assessment of the G2/M checkpoint may serve as a biomarker in identifying Aurora kinase inhibitor sensitive tumors.

## 1. Introduction

Uterine carcinoma (UC) is the most common gynecologic malignancy in the United States with high-grade endometrioid and serous subtypes, which are typically *TP53* mutant, being the most lethal and difficult to treat [1,2]. Once these tumors spread beyond the uterus, limited therapeutic options exist, largely due to our minimal understanding of the major mechanistic defects and drivers of this disease [3]. Genomic sequencing reveals somatic mutations in only a few genes segregating UCs into well-defined molecular subsets [4]. However, beyond genetic alterations in the mismatch repair pathway or *POLE* gene potentially increasing immunotherapy sensitivity [5], few targetable genetic alterations exist across UC subtypes [4].

*TP53* mutations are one of the most common genomic alterations across all high-grade UC subtypes and associate with higher mortality; however, the functional and therapeutic relevance of these alterations remains ambiguous [4,6]. p53 has been widely studied across every malignancy and has many functions during cellular stress, including cell cycle arrest or apoptosis after DNA damage [7,8,9]. However, DNA damage repair (DDR) capacity and cell cycle checkpoint integrity across *TP53* mutant uterine tumors has not been fully characterized.

Of interest are current clinical trials with DDR agents in recurrent UCs [10,11,12]. In one recent clinical trial, a subset of uterine serous tumor patients demonstrated response to the WEE1 inhibitor AZD1775 [13]. AZD1775 can both alter the cell cycle and destabilize replication forks causing DNA damage [14,15,16,17]. It is unclear from this trial whether the functional defect in responsive patients was in cell cycle control or replication fork stability [13]. Indeed, many *TP53* mutant tumors harbor mutations in oncogenes like *MYC*, which might contribute to oncogene driven replication stress [4], while others harbor mutations or copy number alterations in genes with cell cycle regulatory functions such as LKB1 (*STK11*) or *AURKA*, which combined with a *TP53* mutation may alter cell cycle checkpoints [18,19].

Thus, we sought to elucidate the functional and therapeutic relevance of *TP53* mutations in UCs, especially in the capacity of these tumors to repair DNA damage and control cell cycle checkpoints. We demonstrate that most of these tumors lack innate defects in the homologous recombination (HR) and stalled replication fork protection DDR pathways; rather, they harbor innate mitotic progression or spindle defects. This makes them heavily dependent on both defective G2/M checkpoints and upregulated Aurora kinase–LKB1–p53–AKT signaling for survival, increasing sensitivity to the Aurora kinase inhibitor Alisertib. For Alisertib resistant cells, addition of a WEE1 kinase inhibitor promotes mitotic progression after Alisertib induced mitotic alterations, leading to cell death. Our data indicate that Aurora kinase inhibitors alone or in combination with WEE1 inhibitors may offer unique mechanism driven targeted therapies for patients with G2/M checkpoint deficient *TP53* mutant UCs.

## 2. Materials and Methods

For additional Materials and Methods, please see Supplementary Materials and Methods.

### 2.1. TP53 Sequencing

gDNA was prepared from cell lines using Qiagen’s DNeasy Blood and Tissue kit (Cat. #69504). gDNA was analyzed using Genewiz’s *TP53* Sanger sequencing pipeline for exons 1–10 of the *TP53* gene. These results were used to validate publicly available *TP53* mutation status for these cell lines [20,21,22,23].

### 2.2. Next Generation Sequencing of Organoids

gDNA was generated from all six organoid models and parent tumors using Qiagen’s DNeasy Blood and Tissue kit and submitted to the Center for Patient-Derived Models at Dana-Farber Cancer Institute. To validate the parent tumors and organoids as coming from the same patient, all six pairs underwent STR profiling and proved to be exact matches. The organoids underwent copy number and somatic mutation analysis using low pass whole genome sequencing and targeted exome sequencing (ModelSeq panel).

### 2.3. Bulk RNA Sequencing

Organoids were treated with either vehicle or 1 uM AZD1775. After 14 h, the organoids were scraped from the plate, washed in PBS, Matrigel extracted in Corning’s Cell Recovery Solution (Cat. #354253), washed in PBS, and snap frozen. RNA was extracted using Qiagen’s RNeasy kit (Cat. #74104) with on-column DNAse digest (Qiagen Cat. #79254). Total RNA was sequenced using Novogene’s standard Illumina RNAseq platform. VIPER was used for sequencing cleanup and differential expression analysis [24]. GO overrepresentation analysis was performed for significantly upregulated genes using clusterProfiler (logFC > 0, adj. *p* value < 0.05) [25].

### 2.4. Accession Number

The sequencing data discussed in this study have been deposited in the Gene Expression Omnibus (GEO) database and are accessible through GEO Series accession number GSE171516.

## 3. Results

### 3.1. Functional Testing of DDR Pathways in a Panel of TP53 Mutant UC Models Reveals No Significant Defects

Since p53 is a master regulator of the cell cycle and survival amid DNA damage, we asked whether high-grade *TP53* mutant UCs harbor defects in DDR increasing susceptibility to DDR targeted therapies [7,8,9], which target defects in repair of double strand DNA breaks (DSB) by HR and protection of stalled replication forks [26]. Functional assays for HR defects include (1) assessing tumor cell formation of post-damage RAD51 nuclear foci, and (2) challenging cells with DSB inducing agents such as gamma irradiation, PARP inhibitors, and platinum crosslinking agents [27]. Cells with intact HR capacity form foci and are resistant to DSB inducing agents. Functional assays for stalled replication fork protection include (1) DNA fiber assays, and (2) challenging cells with agents that stall replication forks such as gemcitabine, ATR inhibitors, or WEE1 inhibitors [27,28].

**Figure 1:**
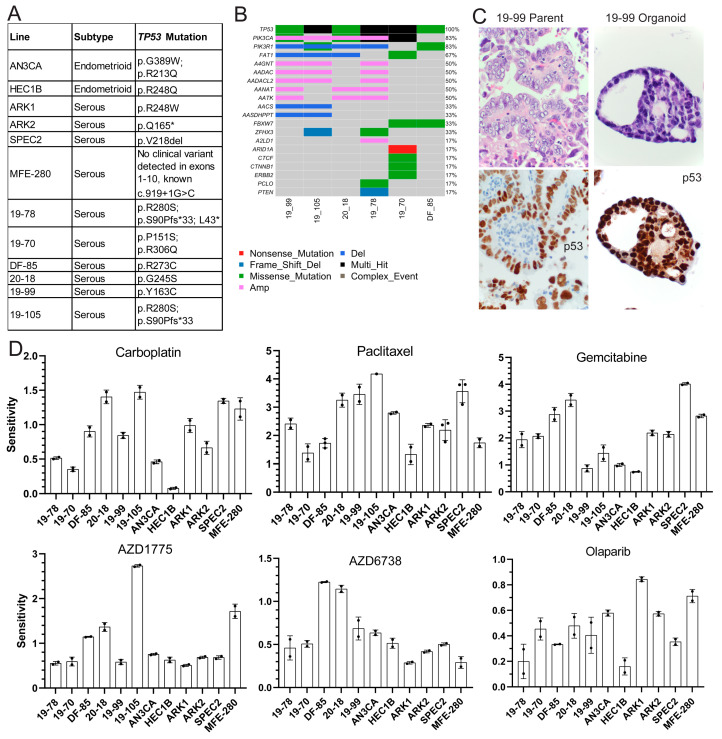
***TP53* mutant uterine carcinoma models show varying responses to classic and DNA damage repair targeted therapies.** (**A**) Histologic subtype and *TP53* mutation status of six cell lines and six patient-derived organoids analyzed in this study. (**B**) The most common somatic mutations or copy number alterations identified in six organoid models by targeted panel exome and low pass whole genome sequencing are shown here with the gene name on the left, the percent of organoids with the altered gene on the right, and the key to organoid line and alteration type on the bottom. (**C**) Hematoxylin and eosin stains of matched parent tumor (top left) and organoid (top right) and p53 immunohistochemistry of the same matched parent tumor (bottom left) and organoid (bottom right). Photos were taken at 63X and then cropped to focus on a single organoid or single tumor region. (**D**) All organoids and cell lines were tested for sensitivity to gemcitabine, AZD6738, Olaparib, AZD1775, carboplatin, and paclitaxel. All lines were growth rate corrected. The sensitivity (area over the growth rate corrected dose curve) is shown here with bars representing the average sensitivity and error bars representing the standard deviation between two to three replicates.

To functionally assess these pathways in *TP53* mutant UCs, we compiled a panel of *TP53* mutant UC cell lines and established multiple patient-derived organoids (PDOs) (Figure 1A–C and Figure S1A,B) [20]. Models were of endometrioid and serous histologies, and organoid sites of origin included both primary hysterectomies and solid or ascites recurrences (Figure 1A and Figure S1A). *TP53* mutations were validated using Sanger or next-generation sequencing in cell lines and organoids, respectively (Figure 1A,B) [20,21,22,23]. The somatic mutation and copy number profile of the organoids was established using next-generation sequencing (Figure 1B), and organoids were histologically matched to parent tumors using hematoxylin and eosin, p53, and PAX8 stains (Figure 1C and Figure S1B). *TP53* was the most common mutation in the organoids followed by various alterations in *PIK3CA* (Figure 1B), but the functional significance of these somatic alterations is unclear.

To test for HR defects, models were treated with either 0 or 5Gy and stained for the HR protein RAD51 and the classic marker of DNA damage γH2AX eight hours later (Figure S2A). The number of co-localizing RAD51/γH2AX foci per nucleus was counted from 100 nuclei for each line (Figure S2A). All models formed post-damage nuclear RAD51 foci, indicating no significant HR defect (Figure S2A).

All models were analyzed for replication fork protection capacity using a DNA fiber assay [29] following treatment with low dose (0.1 mM) hydroxyurea (HU), a classic replication fork stalling agent (Figure S2B). In this assay, the cells are pulsed with two nucleoside analogs, treated with a DNA damaging agent, and the analog-labeled nascent DNA strands are visualized using analog-specific antibodies. The cells are assessed for protection of the second (green) track by comparing the ratio of the second (green) track to the first (red) [28]. If the cell can protect the forks, the ratio will be near one [28]. If there is a fork protection defect, the ratio will be significantly less than 1. All cells showed some fork degradation after HU, but only SPEC2 and 19-105 showed larger drops, suggesting a possible functional defect (Figure S2B). The significance of smaller drops was unclear, and given that different cells cycle at different rates, the ultimate meaning of instability observed in this assay is unclear.

### 3.2. TP53 Mutant UCs Show Varying Sensitivities to Classic Chemotherapies and Targeted DDR Agents

Each model was tested for sensitivity to classic chemotherapeutic agents carboplatin and paclitaxel, and DDR agents targeting different repair pathways, including gemcitabine and the ATR inhibitor AZD6738 for replication fork stalling, Olaparib for HR defects, and the WEE1 inhibitor AZD1775 for cell cycle arrest alteration with some replication fork stalling. All models were treated with dose ranges for each agent to determine sensitivity (Figure 1D and Figure S3). Since each line cycles at a different rate, growth rate corrected dose curves were generated based on mathematical normalization of the treated readouts compared to a day zero readout [30]. The sensitivity, which represents the area over the growth rate corrected dose curve (Figure 1D), and representative growth rate corrected dose curves (Figure S3) are shown for each line with each agent. The greater the area over the growth rate corrected dose curve, the more sensitive the line is to the agent. Sensitivities to carboplatin varied as expected in these higher grade lesions which are sometimes unresponsive to this agent in advanced disease settings (Figure 1D). All lines demonstrated some degree of sensitivity to paclitaxel, which causes spindle formation defects, suggesting possible cell cycle checkpoint issues (Figure 1D). All lines showed resistance to Olaparib, only showing toxicity at the highest dose (Figure 1D and Figure S3) matching their capacity to form post-damage RAD51 foci (Figure S2A) and the lack of genomic alterations in HR genes in the organoids (Figure 1B).

Two lines demonstrated sensitivity to the ATR inhibitor AZD6738 (20-18 and DF-85), indicating its potential utility in only a subset of patients (Figure 1D) [31]. All lines but HEC1B, AN3CA, and 19-99 demonstrated sensitivity to the classic fork stalling nucleoside analogue gemcitabine (Figure 1D) indicating a possible defect upon replication fork arrest due to inhibition of DNA synthesis undetected by DNA fiber analysis (Figure S2B). Finally, only four lines (DF-85, MFE-280, 19-105, and 20-18) showed sensitivity to the WEE1 inhibitor AZD1775 (Figure 1D).

Overall, the focused sensitivities to AZD1775 and the common sensitivity to paclitaxel suggested the potential for a cell cycle checkpoint defect in these cells. The focused sensitivities to AZD6738, AZD1775, and gemcitabine also suggested that these tumors may have mechanistically different replication fork protection defects best detected by challenging cells with each agent and then comparing them.

### 3.3. TP53 Mutant UCs Do Not Show Alterations in Replication Fork Protection but Some Harbor Cell Cycle Checkpoint Difficulties in Response to WEE1 Inhibition

Given the sporadic replication fork and cell cycle defects in our functional assays, we focused on the WEE1 inhibitor AZD1775. This agent causes both cell cycle issues and replication stress and has recently shown some efficacy in a subset of *TP53* mutant uterine serous carcinomas [13]. Therefore, we performed a deeper analysis on some of our models to determine what mechanistic defect caused our observed AZD1775 sensitivity in only a few lines and if other small molecules or combinations show increased efficacy in a larger subset of *TP53* mutant UCs.

We initially treated a subset of cell lines with AZD1775 and assessed upregulation of the replication stress DNA damage response at various timepoints over 48 h by examining expression of key replication stress response proteins involved at stalled forks including early markers like phosphorylated RPA (pRPA) and γH2AX, and later markers like phosphorylated CHK1 (pCHK1) and phosphorylated KAP1 (pKAP1), both targets of the replication stress kinase ATR (Figure S4A) [32]. In all lines, the replication stress pathway was successfully engaged, as evidenced by upregulation of pRPA, γH2AX, pCHK1, and pKAP1, albeit at different timepoints in each cell line likely due to varying cell cycle rates (Figure S4A). To ensure that no differences in replication stress response upregulation were due to lack of target engagement, we assessed WEE1 inhibition by examining RRM2 and phosphorylated CDC2 (pCDC2) levels, which should both decrease post-treatment [16,33]. In all three cell lines tested AZD1775 successfully engaged WEE1, evidenced by decreased phosphorylated CDC2 (Figure S4A). However, faithful degradation of RRM2 was not always observed (Figure S4A). These results suggested the lack of a replication stress response defect and raised the possibility of a cell cycle checkpoint as the critical AZD1775 target in these cells.

**Figure 2:**
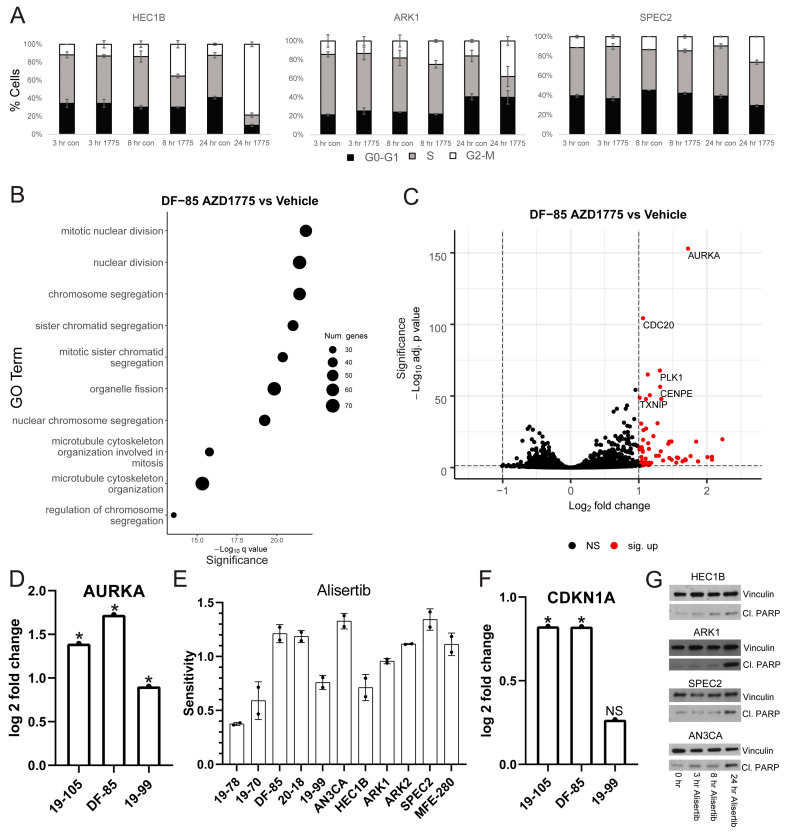
**WEE1 inhibition induces significant *AURKA* upregulation in AZD1775 sensitive uterine carcinoma models, unmasking a strong vulnerability of *TP53* mutant uterine carcinomas to Aurora kinase inhibition.** (**A**) BrdU/propidium iodide cell cycle flow cytometry analysis was performed on a subset of cell lines at various timepoints after vehicle (con) or 0.5 uM AZD1775 (1775) treatment. The bars show different phases of the cell cycle (white G2-M, gray S, black G0-G1), and error bars represent standard deviation between two to three replicates. (**B**) Bulk RNA sequencing analysis of the treated organoids compared to the untreated set revealed significant enrichment for genes related to G2/M after GO overrepresentation analysis. In the graph, GO terms are listed on the *Y*-axis, circles represent the number of genes in the GO term group, and the *X*-axis represents increasing significance. (**C**,**D**) One of the most significantly upregulated genes in each organoid line post-AZD1775 treatment was *AURKA*, as shown in the volcano plot for DF-85 treated vs. vehicle in C and the log2 fold change bar graph in each line in D. For the volcano plot in C, *p*-values of increasing significance are shown on the *Y*-axis and increasing log2 fold change is shown on the *X*-axis. A black circle indicates genes with non-significant (NS) change, and a red circle indicates significantly upregulated genes. For the bar graph in D, *p*-values were generated using an FDR-adjusted Wald test comparing expression in treatment versus control. * *p* < 0.05. (**E**) All cell lines and organoids were tested for sensitivity to the dual Aurora A/B kinase inhibitor Alisertib. Each sensitivity curve was run twice, and all lines were growth rate corrected. The sensitivity (area over the growth rate corrected dose curve) is shown here with bars representing the average sensitivity and error bars representing the standard deviation between two replicates. (**F**) The log2 fold change of AZD1775 treatment compared to control bar graph for *CDKN1A* expression in each line is shown here. *p*-values were generated using an FDR-adjusted Wald test comparing expression in treatment versus control. * *p* < 0.05 and NS = not significant. (**G**) Four of the cell lines were treated with 0.25 uM Alisertib for 24 h and harvested either at the start of the time course (0 hr) or at varying points over the time course. Western blots were performed on lysates from the cell lines to analyze for the apoptosis marker cleaved PARP and for Vinculin as a control.

Thus, we treated the same cell lines with AZD1775 and harvested for cell cycle flow cytometry analysis at various timepoints (Figure 2A). For each line tested, AZD1775 induced some degree of G2/M arrest at 24 h. HEC1B demonstrated the most pronounced arrest suggesting that it has an intact G2/M checkpoint. In contrast, ARK1 and SPEC2 demonstrated smaller arrests, suggesting possible G2/M checkpoint defects. The different degrees of arrest may be important in understanding sensitivity to various late G2/M arresting agents and determining whether the cells arrested in G2 or M phase was important in pinpointing the location of the checkpoint defect in ARK1 and SPEC2.

The cell lines were again treated with AZD1775 and assessed for histone H3 phosphorylated on serine 10 (H3pS10) to mark mitosis at various timepoints after treatment (Figure S4B). Each line showed an expected increase in mitotic entry marked by increased H3pS10 positive cells at eight hours followed by some degree of G2 arrest marked by decreased H3pS10 by 24 h (Figure S4B).

The decreased ability of ARK1 and SPEC2 to arrest in G2/M compared to HEC1B, combined with no clear difference between these three cell lines in their ability to upregulate the replication stress response, hinted that the underlying functional defect in ARK1 and SPEC2 is likely in a part of cell cycle checkpoint regulation not targeted by WEE1. The next question was what the mechanism of the checkpoint defect, if any, was in ARK1 and SPEC2 cells.

### 3.4. WEE1 Inhibition Induces Upregulation of Aurora Kinase Signaling in TP53 Mutant UCs

To determine if other cell cycle pathway defects might influence AZD1775 sensitivity or if there was another molecular defect in these tumors, we studied the transcriptional profiles of cells with varying sensitivity to AZD1775. We profiled a panel of our organoids including resistant (19-99), moderately sensitive (DF-85), and highly sensitive (19-105) lines. Each organoid line was treated with AZD1775 over a time course. Western blots were performed to assess WEE1 engagement (pCDC2, RRM2) and replication stress response upregulation (pRPA, γH2AX, pKAP1, and pCHK1) in each line. As with cell lines, each organoid line had successful WEE1 engagement shown by pCDC2 downregulation but not RRM2 alteration, and successful upregulation of the replication stress response shown by upregulation of pRPA, γH2AX, pKAP1, and pCHK1 at varying times after treatment (Figure S4C). A vehicle and AZD1775-treated sample of each organoid line was submitted for bulk RNA sequencing analysis to assess for alterations in other pathways (Figure 2B,C and Figure S5A,B, and Tables S1–S3).

In each line, gene ontology (GO) overrepresentation analysis on post-AZD1775 treatment upregulated genes revealed marked upregulation in G2/M related pathways (Figure 2B and Figure S5) [25], most pronounced in the more responsive lines DF-85 and 19-105. In all three lines, one of the most upregulated genes post-treatment was Aurora kinase A (*AURKA*) (Figure 2C,D and Figure S5, and Tables S1–S3). Given the G2/M checkpoint deficiency after WEE1 inhibition in ARK1 and SPEC2 (Figure 2A) and the strong upregulation of Aurora kinase after WEE1 inhibition in the most sensitive organoids (DF-85 and 19-105) (Figure 2C,D and Figure S5B), we hypothesized that some *TP53* mutant UCs may have issues with late G2/M checkpoint function and be more reliant on Aurora kinase signaling at baseline or when challenged during mitosis. This Aurora kinase dependence may be a targetable vulnerability with the right small molecules.

To test this, we treated our models with Alisertib (MLN8237), which inhibits Aurora kinase A and to a lesser extent Aurora kinase B at different concentrations (Figure 2E and Figure S3) [34,35]. All of the cell lines and some of the organoids were more sensitive to Alisertib than AZD1775, and the remaining organoids were at least as sensitive to Alisertib as to AZD1775 (Figure 2E and Figure S6A). Aurora kinase inhibitors have had success in other tumor types, including ovarian cancer, suggesting further exploration of the efficacy and mechanism of action of these agents in UC was warranted [36,37,38,39].

Since Alisertib targets both Aurora A and Aurora B kinases at different doses, we tested a subset of the cell lines for cell cycle arresting capacity and sensitivity to the Aurora A specific inhibitor MK5108 and the Aurora B specific inhibitor Barasertib (AZD1152) to determine if inhibition of one or both kinases was more effective (Figure S6B,C) [34,35]. Alisertib resistant HEC1B along with Alisertib sensitive ARK1, SPEC2, and AN3CA were treated with either MK5108 or Barasertib and harvested for cell cycle flow cytometry analysis at various timepoints post-treatment. HEC1B showed a robust G2/M arrest at 24 h for both MK5108 and Barasertib, reflecting its intact G2/M checkpoint (Figure 2A and Figure S6B). ARK1 and AN3CA showed the strongest arrest for Barasertib with a lesser arrest for MK5108 suggesting difficulties in G2/M arrest after Aurora kinase A inhibition (Figure S6B). SPEC2 showed a stronger arrest for MK5108 than for Barasertib, suggesting issues with G2/M arrest after Aurora B inhibition (Figure S6B). ARK1, AN3CA, and SPEC2 showed moderately higher sensitivity to Barasertib over MK5108, however, HEC1B, ARK1, and AN3CA were all more or at least equally sensitive to Alisertib compared to Barasertib or MK5108 (Figure S6C). Overall, the more Alisertib sensitive lines do not arrest as strongly in G2/M amid Aurora kinase A inhibition and are less sensitive to Aurora kinase A inhibition alone. Taken together, these results suggest something unique about the dual Aurora kinase A/B inhibitory properties of Alisertib in UCs.

We next tested if Alisertib exerts its cytotoxicity through apoptosis in UC, as occurs in other tumor types, despite the *TP53* mutations in our UC cells (Figure 1A) [34,35]. In this regard, we found that WEE1 inhibitor sensitive lines DF-85 and 19-105 upregulated the p53 target p21 (*CDKN1A*) after AZD1775 treatment (Figure 2F). p21 has a role in inducing and protecting against apoptosis in p53-dependent and independent manners, suggesting that these *TP53* mutant UCs may undergo apoptosis in response to cell cycle challenges such as WEE1 or Aurora kinase inhibition [40,41]. Thus, we assessed a subset of our cell lines for apoptosis by examining levels of the apoptosis marker cleaved PARP after Alisertib treatment (Figure 2G). HEC1B, SPEC2, ARK1, and AN3CA demonstrated increased cleaved PARP after Alisertib treatment (Figure 2G) suggesting that even in these *TP53* mutant cells, apoptosis was induced.

Overall, these results suggested that later G2/M arrest at the time of spindle formation targeted by Aurora kinase inhibition may be a more effective therapeutic target than WEE1 inhibition in *TP53* mutant UCs. We next questioned how these agents induced apoptosis in *TP53* mutant cells.

### 3.5. Alisertib Induces Apoptosis in TP53 Mutant UCs by Targeting Aurora Kinase–LKB1–p53–AKT Interlinked Signaling in a Mutant p53 Dependent Mechanism

**Figure 3:**
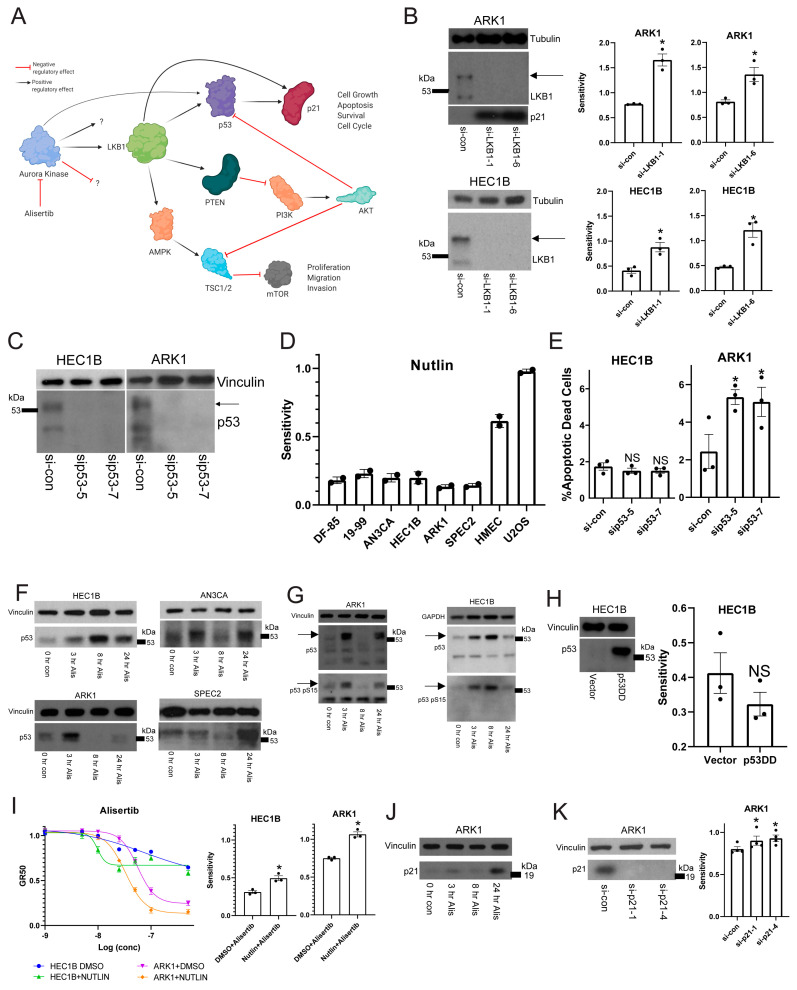
**Aurora kinase inhibitor cytotoxicity is mediated in part through the function of mutant p53 and LKB1 signaling.** (**A**) A cartoon demonstrating how Aurora kinase is linked to both the p53/p21 and PI3K/AKT arms of LKB1 kinase signaling. (**B**) ARK1 and HEC1B cells were transfected with either a control siRNA (si-con) or one of two LKB1 specific siRNAs (si-LKB1-1 or si-LKB1-6) and then treated with a dose range of Alisertib. Western blots showing LKB1 depletion, and p21 upregulation in the case of ARK1, are shown on the left. Two bands were detected, and the top band indicated by an arrow represents the phosphorylated form. Sensitivities (area over the growth rate corrected dose curve) are shown on the right for each cell line with each siRNA. Bars represent the average of three replicates, and error bars represent standard error of the mean. *p*-values were calculated using a paired *t*-test. * *p* < 0.05. (**C**) Western blots demonstrating p53 depletion in HEC1B and ARK1 with two p53 specific siRNAs (sip53-5 and sip53-7) compared to a control. Two isoforms of p53 are detected, but the top isoform running at 53kDa indicated by an arrow here represents the full length p53 changing with treatment in the remaining figures. (**D**) A representative subset of *TP53* mutant uterine cancer cell lines and organoids along with *TP53* wild type human mammary epithelial cells (HMEC) and U2OS cells were treated with a dose range of Nutlin and analyzed for survival. All lines were growth rate corrected, and the sensitivity (area over the growth rate corrected dose curve) is shown here for each line. The bars represent the average sensitivity, and error bars represent the standard deviation for two replicates. (**E**) HEC1B and ARK1 were transfected with two separate p53-specific siRNAs compared to control (si-con) and analyzed for apoptotic dead cells 72 h later. The average percentage of apoptotic dead cells is shown here with error bars representing the standard error of the mean and *p*-values generated using a *t*-test for three replicates. * *p* < 0.05 and NS = not significant. (**F**) Cell lines were treated with 0.25 uM Alisertib (Alis) over a 24 h period compared to a 0 h control (con), and lysates were prepared at various timepoints and blotted for full length p53 with vinculin as a loading control. (**G**) Cell lines were treated with 0.25 uM Alisertib (Alis) over a 24 h period compared to a 0 h control (con), and lysates were prepared at various timepoints and stained for p53 phosphorylated on serine 15 (p53 pS15). Loading controls included vinculin, GAPDH, and p53. An arrow indicates the 53kDa molecular weight band that represents full length p53 and is phosphorylated and changing with treatment. (**H**) HEC1Bs were stably transfected with empty vector or a dominant negative p53 (p53DD) and then treated with a dose range of Alisertib. A western blot for p53 is shown on the left, showing the overexpressed protein correctly running at 53kDa, and the sensitivity (area over the growth rate corrected dose curve) for Alisertib is shown on the right. Bars represent the average of three replicates, and error bars represent the standard error of the mean. *p*-values were generated using a *t*-test comparing vector to p53DD. NS = not significant. (**I**) ARK1 and HEC1B cells were treated with a dose range of Alisertib in combination with either a fixed dose of DMSO or a fixed dose of 5 uM Nutlin. Representative growth rate corrected dose curves are shown on the left with a key to treatments/cell lines on the bottom, and the sensitivity (area over the growth rate corrected dose curve) for both lines is shown on the right. Bars represent the average of three replicates, and error bars represent standard error of the mean. *p*-values were calculated using a paired *t*-test comparing vehicle-Alisertib to Nutlin-Alisertib. * *p* < 0.05. (**J**) ARK1 cells were tested for p21 upregulation after 0.25 uM Alisertib (Alis) treatment over a 24 h period. An untreated control (con) was harvested at 0 h. p21 expression was studied by western blot over the time course with vinculin as a loading control. (**K**) ARK1 cells were transfected with either a control siRNA (si-con) or one of two p21 specific siRNAs (si-p21-1 or si-p21-4) and then treated with a dose curve of Alisertib. On the left is a western blot for p21 in the control or p21 depleted cells with vinculin as a loading control. On the right is the sensitivity (area over the growth rate corrected dose curve) for the growth rate corrected dose curves of the cells. Four replicates were performed with each siRNA. Error bars represent the standard error of the mean, and *p*-values were generated using a *t*-test comparing si-con to gene specific siRNAs. * *p* < 0.05.

Aurora kinases link to apoptosis signaling pathways regulated by the LKB1 serine/threonine kinase, including p53 and PI3K/AKT (Figure 3A) [18,35]. LKB1 is a tumor suppressor involved in many cellular functions surrounding cell cycle control, energy, metabolism, and polarity which it regulates through AMPK/mTOR signaling [18,42]. In one pathway LKB1 regulates p53 and p21 cell cycle and apoptosis control, and in another linked pathway LKB1 regulates PI3K/AKT mediated proliferation and anti-apoptotic signaling (Figure 3A) [18]. The Alisertib target Aurora kinase A phosphorylates and regulates LKB1 [35,43], and also phosphorylates and activates p53 [34,35] (Figure 3A). Given the connections between Aurora kinase A, LKB1, p53, and AKT, our question was if Alisertib induced apoptosis through inhibition somewhere within the web of LKB1 signaling despite the genomic alterations in *TP53* and the PI3K/AKT family in most of our models (Figure 1A,B) [20,21,22,23].

To test this, we investigated potential synthetic lethality with Aurora kinase inhibition and LKB1 loss of function. In both the G2/M checkpoint defective/Alisertib sensitive ARK1 and the G2/M checkpoint proficient/Alisertib resistant HEC1B lines, LKB1 depletion increased Alisertib sensitivity (Figure 3B). Additionally, p21, which is not expressed in HEC1B [44], was upregulated after LKB1 inhibition in ARK1 cells serving as a control, since LKB1 normally regulates p21 (Figure 3B). This result indicated that Alisertib induced apoptosis occurred either through dysregulation or inhibition of the p53 or AKT mediated arms of the LKB1 pathway, or both. Thus, we next asked which LKB1 pathway Alisertib targeted.

We initially hoped to test whether p53 was involved in the Alisertib response. However, all of our organoids and cell lines are p53 mutant (Figure 1A). Thus, we first had to address whether the mutant p53 was expressed and active in our UC models. We examined p53 expression in two of the lines, HEC1B and ARK1, after treatment with control or p53 specific siRNAs (Figure 3C) to determine if the mutant p53 was expressed and which isoforms were relevant. We found two specific and shared isoforms with the higher isoform expressed near 53kDa as expected for full length p53, and both isoforms were reproducibly depleted by the *TP53* specific siRNAs (Figure 3C).

Given this, our next question was whether or not the expressed mutant p53 had any baseline function. To test for an inhibitory function, we treated a subset of our cell lines and organoids with Nutlin, which causes p53 activation by inhibiting the p53 regulatory partner MDM2 [45,46]. Cells with functional p53 at baseline are highly sensitive to Nutlin [45,46]. As controls, we tested U2OS cells and human mammary epithelial cells (HMECs), both known to be *TP53* wild type. We found that all our tested models are Nutlin resistant compared to the U2OS and HMEC controls (Figure 3D), suggesting the mutant p53 did not have an inhibitory baseline function.

To assess if the mutant p53 had a protective baseline function, we depleted p53 with multiple gene specific siRNAs and assessed for apoptotic death (Figure 3E). We found that in ARK1 but not in HEC1B, p53 depletion leads to increased apoptosis (Figure 3E). Since ARK1 appears to have a deficient G2/M checkpoint while HEC1B is proficient, we hypothesized that perhaps the mutant p53 serves a protective baseline function allowing ARK1 cells to progress through mitosis with some degree of mitotic defects. When the mitotic defects are enhanced at the right point in mitosis, such as with the spindle defects induced by Alisertib, then this mutant p53 may serve a different function.

Thus, we next tested if mutant p53 has a role in the Alisertib response. First, we studied p53 expression in SPEC2, AN3CA, HEC1B, and ARK1 after Alisertib treatment to see if p53 was upregulated in response to treatment. We found the full length 53kDa molecular weight band of mutant p53 upregulated in all lines at various timepoints post-treatment (Figure 3F). In ARK1, AN3CA, and SPEC2, this upregulated band appeared as a doublet or a smear, suggesting that the mutant p53 was undergoing some form of post-translational modification in response to Alisertib. Indeed, p53 is phosphorylated during cell cycle arrest or other states of cellular stress releasing it from control by its regulatory partner MDM2 [7,8,45,46]. Thus, we tested for phosphorylation of mutant p53 after treatment. We found that the post-Alisertib upregulated p53 in HEC1B and ARK1 is phosphorylated on Serine 15, suggesting it is active (Figure 3G). The next question was if mutant p53 has a role in the apoptotic response to Alisertib.

To test this, we tried multiple methods of altering p53 function in combination with Alisertib. First we overexpressed a dominant negative form of p53 (p53DD) in HEC1Bs, which should bind and block the function of any p53, to determine whether losing p53 function altered Alisertib sensitivity (Figure 3H) [47]. This dominant negative p53 was heavily expressed at the same molecular weight of 53 kDa as the endogenous p53 (Figure 3C,F,G) but caused only a small and not statistically significant decrease in HEC1B Alisertib sensitivity, suggesting that p53 function may be needed to enhance the Alisertib response (Figure 3H). Thus, we next asked whether activating the mutant p53 by releasing it from MDM2 with Nutlin might increase sensitivity to Alisertib (Figure 3I). Indeed, treatment of HEC1B or ARK1 cells with an Alisertib dose curve combined with a constant dose of Nutlin compared to vehicle increased the sensitivity of these cells to Alisertib (Figure 3I). Taken together, these data indicate that mutant p53 and thus the p53 arm of the LKB1 pathway is not inhibited by Alisertib, rather it actively influences Alisertib induced cell death (Figure 3I). The next question was whether the p53 and LKB1 target p21 had any role in Alisertib response, given its upregulation in response to other cell cycle altering drugs like WEE1 inhibitors (Figure 2F and Figure 3A).

To test for a p21 role in response to Alisertib in *TP53* mutant UCs we treated ARK1 with Alisertib as HEC1B does not express p21 [44], harvested at various timepoints, and found p21 upregulated post-treatment (Figure 3J). We next asked whether p21 was protective or pro-apoptotic by depleting p21 in ARK1 cells and studying Alisertib response after p21 loss (Figure 3K). Upon p21 depletion, ARK1 cells showed a modest increase in Alisertib sensitivity, suggesting either a limited role or possibly a small anti-apoptotic role for p21 during Alisertib induced G2/M arrest (Figure 3K).

**Figure 4:**
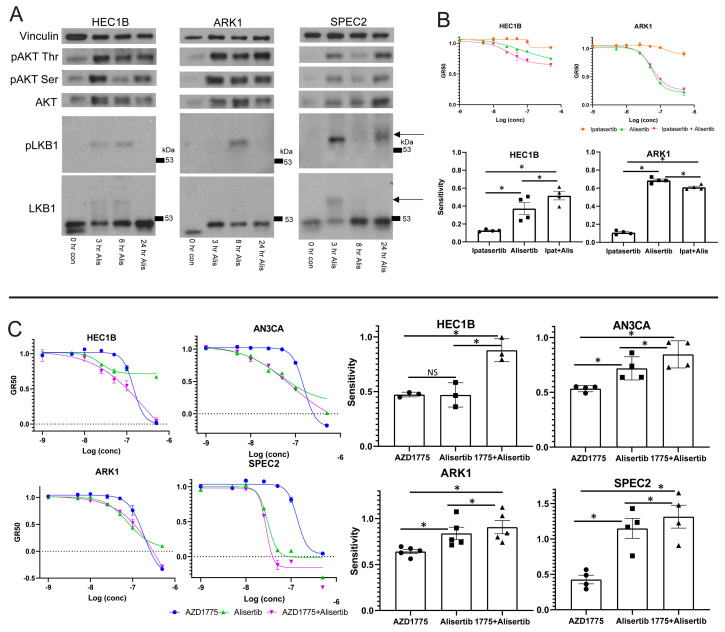
**Combining Aurora kinase and WEE1 kinase inhibitors overcomes Aurora kinase inhibitor resistance.** (**A**) Cell lines were treated with 0.25 uM Alisertib (Alis) over a 24 h period compared to a 0 h untreated control (con), and lysates were prepared from cells harvested at 3, 8, and 24 h post-treatment and analyzed for LKB1 and AKT expression and modification (AKT phosphorylated on Serine 473 (pAKT Ser) or Threonine 308 (pAKT Thr) and LKB1 phosphorylated on Serine 428 (pLKB1)). In the LKB1 and pLKB1 blots, an arrow indicates the higher molecular weight phosphorylated band. (**B**) An Alisertib resistant (HEC1B) and sensitive (ARK1) cell line was treated with either Alisertib, Ipatasertib, or a combination of Alisertib and Ipatasertib (Ipat + Alis). Representative growth rate corrected dose curves are shown for each line on the left, and sensitivity (area over the growth rate corrected dose curve) is shown on the right for each line with each treatment. Bars represent the average of four replicates, and error bars represent the standard error of the mean. *p*-values were calculated using both a two-way ANOVA test and a paired *t*-test comparing different single treatments to the combination or each other. * *p* < 0.05 for both tests. Comparisons are indicated by a horizontal line over the bars for the treatments being compared (Alisertib vs. Ipatasertib, Alisertib vs. Ipatasertib+Alisertib, and Ipatasertib vs. Ipatasertib+Alisertib). (**C**) An Alisertib resistant (HEC1B) and three sensitive (ARK1, SPEC2, AN3CA) cell lines were treated with either Alisertib, AZD1775, or a combination of Alisertib and AZD1775. Representative growth rate corrected dose curves are shown for each line on the left, and sensitivity (area over the growth rate corrected dose curve) is shown on the right for each line with each treatment. Bars represent the average of three to five replicates, and error bars represent standard error of the mean. *p*-values were calculated using both a two-way ANOVA test and a paired *t*-test comparing different single treatments to the combination or each other. * *p* < 0.05 for both tests. Comparisons are indicated by a horizontal line over the bars for the treatments being compared (Alisertib vs. AZD1775, Alisertib vs. AZD1775+Alisertib, and AZD1775 vs. AZD1775+ Alisertib).

Our next question was whether or not the AKT arm of LKB1 signaling was inhibited by or is actively involved in the Alisertib response, especially since there are mutations in members of this pathway in most of our models (Figure 1B) [20,23]. LKB1 and AKT activation require phosphorylation [18,48], and we hypothesized that if Alisertib induced death relies upon LKB1–AKT signaling, there may be differences in the activation of this pathway in Alisertib resistant versus sensitive cells. Accordingly, we assessed phosphorylated LKB1 or AKT upregulation after Alisertib treatment in multiple UC lines (Figure 4A). In Alisertib resistant HEC1B and sensitive SPEC2 and ARK1, phosphorylated LKB1 and AKT reproducibly increased at various timepoints between three and 24 h after Alisertib treatment. In addition, AKT was phosphorylated to a lesser extent at baseline in all three lines (Figure 4A), reflecting its potential baseline activation by alterations in the pathway which are common in UC [20,23]. Taken together, this indicated that LKB1–AKT signaling was already active at baseline possibly serving some protective function, upregulated in response to Alisertib, and intact to the point of AKT phosphorylation in all UC lines tested. Our next question was whether the upregulated AKT was active in the cellular response to Alisertib.

To address this, we treated ARK1 and HEC1B with either a dose curve of Alisertib, the pan-AKT inhibitor Ipatasertib, or Alisertib combined with Ipatasertib, hypothesizing that if AKT plays an active role in the cellular response to Alisertib, then its inhibition should alter Alisertib sensitivity (Figure 4B) [48,49]. In ARK1 and HEC1B, cells showed no response to Ipatasertib as a single agent (Figure 4B). The Alisertib+Ipatasertib combination induced only small changes compared to Alisertib alone. In Alisertib resistant HEC1B, the combination of Alisertib with Ipatasertib caused a small but significant increase in sensitivity over Alisertib (Figure 4B). In contrast, in Alisertib sensitive ARK1 cells, the combination caused a modest but significant decrease in sensitivity to Alisertib. These results suggest that the AKT arm of the LKB1 signaling pathway is a major target of Alisertib but is incompletely downregulated by it, as shown by the minute changes induced by AKT inhibition in combination with Alisertib over Alisertib alone. The fact that the pathway is a target suggests it plays a role in protecting G2/M checkpoint deficient cells at baseline, as these cells undergo apoptosis when the pathway is inhibited by Alisertib. Further inhibiting the pathway, at least with AKT inhibition, did not strongly enhance Alisertib induced cell death, suggesting that Alisertib inhibits more of the LKB1–AKT signaling web than just AKT.

Taken together, these data suggested that (1) both Alisertib sensitive (ARK1 and SPEC2) and resistant (HEC1B) cells rely upon LKB1–p53–AKT interconnected signaling, and (2) Alisertib engages the p53 and AKT arms of the LKB1 pathway simultaneously likely utilizing the p53 arm to induce cell death and blocking the protective AKT arm. However, Alisertib sensitivity requires an additional functional defect as evidenced by the resistance of HEC1B when compared to ARK1 or SPEC2. Consequently, our next question was what the additional defect mediating Alisertib sensitivity was and how Alisertib resistance in cells lacking such a defect, like HEC1B, can be overcome.

### 3.6. Combining Aurora Kinase and WEE1 Inhibitors Overcomes Aurora Kinase Inhibitor Resistance

HEC1B demonstrated the strongest cell cycle checkpoint arrest in response to WEE1 or Aurora kinase inhibition (Figure 2A and Figure S6B), and we hypothesized that their Alisertib resistance was due to this functional G2/M checkpoint. Alisertib induces mitotic spindle defects, halting mitosis at metaphase [50]. In head and neck cancers, regardless of *TP53* mutation status, combining WEE1 and Aurora kinase inhibitors leads to enhanced cell death over either agent alone because WEE1 inhibition triggers mitotic progression in cells with Alisertib induced spindle defects [38]. We speculated that major spindle defects induced by Aurora kinase inhibition combined with both G2/M checkpoint defects and dysregulation of the Aurora kinase regulated LKB1–p53–AKT pathway may allow for efficient Alisertib killing of most *TP53* mutant UC cells. In those with intact G2/M checkpoints, like HEC1B, WEE1 inhibitor-induced mitotic progression is needed in tandem with Aurora kinase inhibitor-induced spindle defects for more efficient cell death.

To test this, we treated HEC1B, ARK1, SPEC2, and AN3CA with either Alisertib, AZD1775, or Alisertib+AZD1775 and assessed cell survival (Figure 4C). In the Alisertib sensitive lines AN3CA, ARK1, and SPEC2, there was a minor but statistically significant increase in sensitivity for the combination over Alisertib (Figure 4C). In contrast, in Alisertib resistant HEC1B cells, the combination of AZD1775 with Alisertib led to a large and statistically significant increase in sensitivity compared to either agent alone, and also larger than combining either an MDM2 or an AKT inhibitor with Alisertib (Figure 3I and Figure 4B,C). 

This increased sensitivity in the resistant HEC1B line and to a smaller extent in the three Alisertib sensitive lines could be due to (1) the combination of DNA damage induced by AZD1775 with mitotic irregularities induced by Alisertib, or (2) the combination of progression into mitosis induced by AZD1775 with mitotic irregularities induced by Alisertib. To determine which occurred, all four lines were treated with vehicle, Alisertib, AZD1775, or Alisertib+AZD1775 and assessed for replication stress response and cell cycle proteins by western blot (Figure S7A). All lines tested showed some upregulation of the replication stress response proteins pRPA, γH2AX, pKAP1, and pCHK1 in response to AZD1775 alone or in combination with Alisertib, indicating no replication stress response defects (Figure S7A). AZD1775 clearly engaged WEE1, evidenced by decreased pCDC2 expression over time (Figure S7A). In Alisertib resistant HEC1B, AZD1775 alone or in combination with Alisertib induced progression into mitosis evidenced by strong H3pS10 expression (Figure S7A). Comparatively, Alisertib sensitive AN3CA and ARK1 had H3pS10 expression and progression to mitosis with AZD1775 alone, but they had less H3pS10 expression and possibly less progression through mitosis in the setting of the combination (Figure S7A). However, these lines also showed less G2/M arrest with AZD1775, MK5108, and Barasertib alone compared to HEC1B (Figure 2A and Figure S6B). SPEC2 demonstrated strong H3pS10 with either single agent or the combination at 24 h which was expected given its lack of arrest with all agents tested thus far (Figure 2A and Figure S6B). Thus, we hypothesized that HEC1B maintains a strong G2/M checkpoint compared to the other cell lines and the double Aurora and WEE1 kinase block is needed to advance mitosis amid mitotic/spindle abnormalities, leading to cell death.

**Figure 5:**
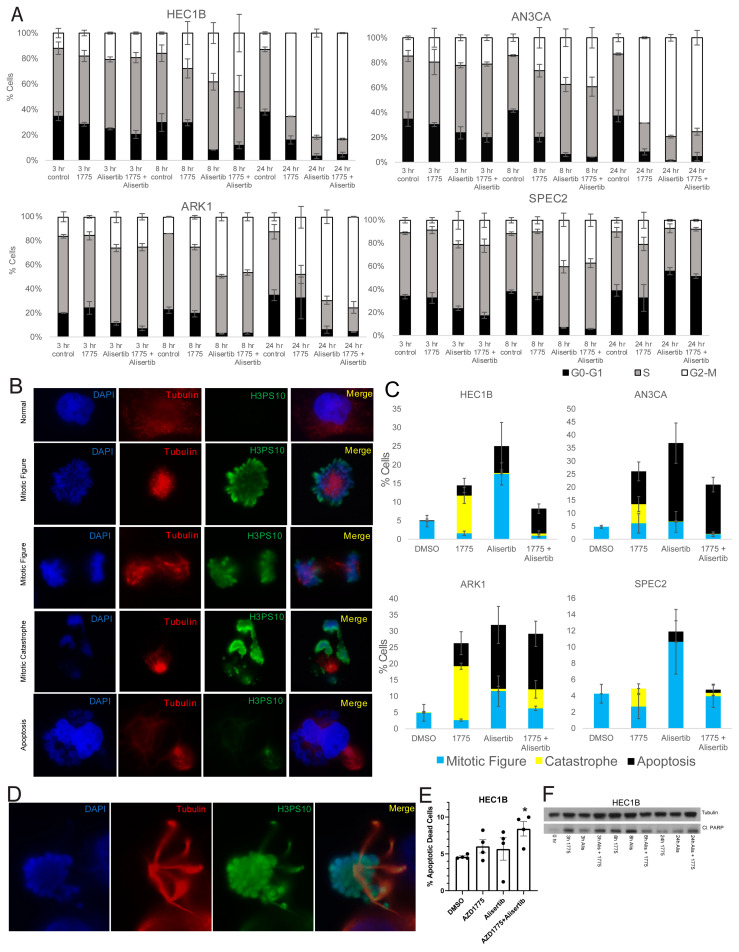
**Combining Aurora kinase and WEE1 inhibitors causes increased apoptotic death in Aurora kinase inhibitor resistant cells.** (**A**) Cell lines were treated with vehicle (control) or either 0.25 uM Alisertib, 0.5 uM AZD1775 (1775), or a combination of Alisertib+AZD1775 and harvested at 3, 8, and 24 h post treatment for BrdU cell cycle flow cytometry analysis. Bar graphs showing the percentage of cells in G0-G1 (Black), S (Gray), and G2/M (White) at each timepoint after each treatment are shown here. Error bars represent standard deviation between two to three replicates. (**B**,**C**) Cell lines were treated with vehicle (DMSO) or either 0.25 uM Alisertib, 0.5 uM AZD1775 (1775), or a combination of Alisertib and AZD1775 and harvested at 24 h for immunofluorescence analysis of mitosis and apoptosis. Cells were stained for H3pS10 to mark mitosis, tubulin to mark spindles, and DAPI to mark nuclei. Representative images of each cell type or phase counted are shown on the left in B, and bar graphs of the counts are shown on the right in C. For the images, photos were taken at 63X and then cropped to focus on a group of cells. Normal or Alisertib altered mitotic figures (blue), mitotic catastrophes (yellow) and apoptotic blebbing cells (black) were counted per at least 100 nuclei for each treatment. Bars represent averages of three replicates, and error bars represent standard deviation. (**D**) A representative altered mitotic figure with abnormal spindles is shown here for Alisertib treated HEC1B cells at 24 h, which were counted in C. For the image, a photo was taken at 100X and then cropped to focus on the single mitotic figure. (**E**) HEC1B cells were treated with vehicle (DMSO) or either 0.25 uM Alisertib, 0.5 uM AZD1775, or a combination of Alisertib and AZD1775 and harvested 24 h post-treatment for apoptosis flow using Apotracker. The percentage of dead apoptotic cells for each treatment is shown for four replicates. Bars represent averages of four replicates, and error bars represent standard error of the mean. *t*-tests were used to calculate *p*-values, and a * above a bar indicates significance compared to the DMSO control. * *p* < 0.05. (**F**) HEC1B were treated with nothing (0 hr) or either 0.25 uM Alisertib (Alis), 0.5 uM AZD1775 (1775), or a combination of Alisertib+AZD1775, harvested at various times post-treatment, and analyzed by western blot for cleaved PARP and tubulin as a control.

To test this, AN3CA, ARK1, SPEC2, and HEC1B were treated with vehicle, Alisertib, AZD1775, or Alisertib+AZD1775, and cell cycle progression was assessed by flow cytometry analysis. HEC1B, ARK1, and AN3CA all showed significant G2/M arrest with both Alisertib or Alisertib combined with AZD1775 while SPEC2 did not, reflecting its inability to arrest after any challenge to G2 or M progression (Figure 2A, Figure 5A and Figure S6B). For HEC1B, ARK1, and AN3CA, this indicated some form of arrest either in G2 or potentially mitosis.

To better assess mitotic figure formation, mitotic catastrophe, or apoptotic blebbing post-treatment, AN3CA, ARK1, SPEC2, and HEC1B were treated with vehicle, Alisertib, AZD1775, or Alisertib+AZD1775 and stained for H3pS10 to mark mitosis and tubulin to mark mitotic spindles 24 h after treatment. Cells with normal or Alisertib altered mitotic figures, mitotic catastrophes, and apoptotic blebs were counted in each setting (Figure 5B–D). Alisertib altered mitotic figures demonstrated distinct spindle formations and never revealed anaphase forms (Figure 5D). Overall, the result for each treatment fit what was expected for the treatment and checkpoint function background.

In the vehicle treated group, the percentage of mitotic cells was uniform near 5% for all four cell lines (Figure 5C). For AZD1775, we showed that treatment at the 0.5 uM concentration used here pushes cells into mitosis at approximately eight hours post-treatment (Figure S4B), so at this 24 h timepoint, we would expect to see the aftermath of that push. Indeed, increased mitotic catastrophes were present in all four cell lines, and increased apoptotic blebbing cells were present in HEC1B, AN3CA, and ARK1 after single agent AZD1775 (Figure 5C).

For Alisertib, we expect to see significantly more mitotic figures and potentially some level of apoptosis based on flow cytometry and western analysis (Figure 2G and Figure 5A). All four cell lines showed some level of apoptotic blebbing cells and the most mitotic figures with Alisertib compared to other treatments (Figure 5C). Fitting with the Alisertib sensitive phenotype, the apoptotic blebbing cells were the highest after Alisertib alone for ARK1, SPEC2, and AN3CA (Figure 5C).

Finally, for the Alisertib+AZD1775 combination, we saw some percentage of apoptosis, mitotic figures, and catastrophes in all four cell lines (Figure 5C). Knowing that AZD1775 induces a push into mitosis around eight hours of treatment and that Alisertib induces increased numbers of altered mitotic figures simultaneous to this, by 24 h we expect to see the aftermath of a push through mitosis of a malformed mitotic figure. In cells with dysfunctional G2/M checkpoints like ARK1, SPEC2, and AN3CA, we still observed some mitotic figures, likely reflecting the dysfunctional G2/M checkpoint regardless of treatment (Figure 5C). In addition, apoptotic blebs were present in ARK1 and AN3CA likely due to their known Alisertib sensitivity and the lack of an effect of AZD1775 on their already defective checkpoints (Figure 5C). In contrast, for HEC1B there were less than 1% mitotic cells and only apoptosis with the combination (Figure 5C), fitting with the hypothesis that the AZD1775 block of the checkpoint in these cells pushes the Alisertib induced abnormal mitotic figures through mitosis when they otherwise would have arrested, and then these abnormal mitotic cells enter apoptosis. Since blebbing represents only one phase of apoptosis, we next wanted to confirm our findings with additional apoptosis markers, especially in SPEC2 where apoptotic cells were not as easily observed.

**Figure 6:**
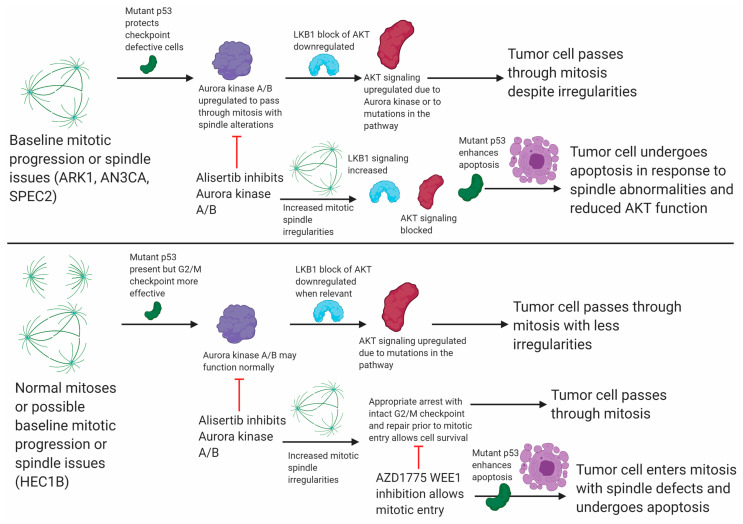
**Aurora kinase signaling is an effective target in *TP53* mutant uterine carcinomas with G2/M checkpoint dysfunction.** Illustration of Alisertib effects on cells with or without functional G2/M checkpoints and how the addition of AZD1775 helps increase Alisertib efficacy.

To demonstrate the increased apoptosis induced by the combination in HEC1B cells beyond just what was visible by eye, HEC1B were treated with vehicle, Alisertib, AZD1775, or Alisertib+AZD1775 and assessed for increased apoptosis by flow cytometry and cleaved PARP western blot 24 h after treatment. The combination was the only treatment to induce significant apoptosis over control by flow cytometry in HEC1B (Figure 5E), and at 24 h the combination had the highest level of cleaved PARP, albeit both single drugs and the combination induced cleaved PARP as well at earlier timepoints (Figure 5F). The same flow cytometry was performed for ARK1, SPEC2, and AN3CA (Figure S7B). SPEC2 showed significant apoptosis only with Alisertib fitting with the previous counts (Figure S7B and Figure 5C), ARK1 with both AZD1775 and Alisertib, and AN3CA most strongly with the combination but also with Alisertib (Figure S7B). A similar western blot time course for ARK1 revealed the strongest cleaved PARP at 24 h for the combination similar to HEC1B (Figure S7C). Taken together these data support that combined WEE1–Aurora kinase inhibition is needed to overcome intact G2/M checkpoints and induce apoptosis in baseline Aurora kinase inhibitor resistant cells, like HEC1B (Figure 6).

## 4. Discussion

The functional and therapeutic significance of *TP53* mutations in UC has not been well defined. Here, using a panel of *TP53* mutant UC cell lines and PDOs, we determined the key functional defect in these tumors is not in the replication stress DNA damage response but rather in mitosis and G2/M cell cycle checkpoints. This defect reveals a unique dependency in many of these tumors on Aurora kinase–LKB1–p53–AKT mediated cell cycle checkpoint control, often altered in UCs through *TP53*, *PIK3CA*, or *AKT1* mutations [18,42]. This dependency reveals a novel mechanism for *TP53* mutant UC cell survival and two new therapies to target this mechanism, including Aurora kinase inhibitors either alone or in combination with WEE1 inhibitors.

Key to discovering this mechanistic defect was functional assessment of G2/M cell cycle checkpoints across a panel of *TP53* mutant models. Our data indicate that at baseline many *TP53* mutant UC cells, represented here by ARK1, AN3CA, and SPEC2, harbor difficulties with G2/M checkpoints and mitosis yet survive due to a combination of mutant p53 protein function and upregulation of Aurora kinase–LKB1–p53–AKT signaling (Figure 2, Figure 3, Figure 4, Figure 5 and Figure 6). Alisertib treatment generates increased altered mitotic figures in these already G2/M checkpoint defective cells (Figure 5C,D). Simultaneously, it alters the LKB1–p53–AKT signaling they normally rely upon to progress through mitosis with these defects, in tandem leading to apoptotic cell death (Figure 6). In contrast, in other *TP53* mutant UC cells, represented by HEC1B, there is an intact G2/M checkpoint that handles mitotic irregularities appropriately at baseline regardless of LKB1–AKT pathway status (Figure 2A, Figure 5A,C, Figure 6, and Figure S6B). In the setting of Aurora kinase inhibition in these cells, altered mitoses may form, but the intact checkpoint allows the cells to repair these issues without as much baseline reliance on AKT signaling (Figure 5A,C and Figure 6). We show that combining WEE1 and Aurora kinase inhibition in these cells allows for altered mitoses to form and forces them to progress in mitosis leading to apoptotic cell death (Figure 4C, Figure 5, and Figure 6). This mechanism driven combination may be effective not just in uterine but potentially in *TP53* mutant ovarian cancer as well, where Alisertib has been tested as a single agent or in combination with paclitaxel with some success [39]. Understanding the functional defects (e.g., G2/M checkpoint) or essential drivers (e.g., AKT/PI3K) in every unique tumor to identify the best combinations will be critical in further studies.

In elucidating this mechanism, we have defined an unexpected fundamental role for the commonly mutated PI3K/AKT pathway in UC [4]. The pathway has roles in many major cellular processes including control of cell growth, apoptosis, the cell cycle, and cellular polarity amongst others and was thought to drive growth and survival in these cells [18]. However, we show that genomic alterations in the PI3K/AKT signaling pathway leading to constitutive activation, such as those in our models (Figure 1B) [20,23], may combine with G2/M checkpoint defects to help tumor cells survive traversing the cell cycle despite issues with irregular mitoses, highlighting this pathway as a critical dependency and major target for an unexpected reason (Figure 4A,B and Figure 6).

In addition, we demonstrate an unexpected role for the mutant p53 present in these tumors. We show that mutant p53 has a protective role at baseline in G2/M checkpoint deficient ARK1 cells, is upregulated and phosphorylated in response to Alisertib treatment, and that increasing mutant p53 activity through Nutlin induced release from MDM2 control enhances Alisertib toxicity (Figure 3). Taken together, these results suggest mutant p53 may still participate in cell cycle control or apoptosis amid cellular stress at baseline and due to treatment. This raises the possibility that activating mutant p53 alongside small molecules targeting other molecular defects may be worth exploring in UC [45,46].

## 5. Conclusions

Overall, these results reveal many *TP53* mutant UCs have difficulties traversing mitosis and depend upon both late G2/M checkpoint defects and Aurora kinase–LKB1–AKT–p53 interconnected signaling to survive, making them highly sensitive to Aurora kinase inhibition. These defects are not readily detected by genomic sequencing or histological analyses; rather, functional testing of key cell cycle checkpoints after challenges with different small molecules is necessary to detect the defect and predict therapy response. In a clinical setting, this may be accomplished by assessing checkpoint efficacy after different therapeutic challenges in PDOs as a biomarker for response to each respective therapy. Further work will be needed in PDOs across all UC histology types (clear cell, carcinosarcoma, etc.) with all types of *TP53* mutations (deletion, point mutation, etc.) to validate this possible biomarker. For those *TP53* mutant UCs with intact G2/M checkpoints, combining WEE1 and Aurora kinase inhibitors is an effective strategy. These therapies represent relevant mechanism driven strategies for patients with these difficult to treat tumors who have limited options beyond standard chemotherapy and surgery.

## Data Availability

All bulk RNA sequencing differential expression matrices, Modelseq, and low pass whole genome sequencing data have been deposited in the Gene Expression Omnibus (GEO) database and are accessible through GEO Series accession number GSE171516.

## Supplementary Materials

The following are available online at https://www.mdpi.com/article/10.3390/cancers13092195/s1, Figure S1: Uterine carcinoma patient-derived organoid model properties, Figure S2: DNA damage repair defect profiling in a panel of *TP53* mutant uterine carcinoma models does not reveal any common functional defects, Figure S3: Dose curves for all lines with all agents, Figure S4: *TP53* mutant uterine carcinomas demonstrate cell cycle checkpoint and not DNA damage repair defects, Figure S5: Bulk RNA sequencing of organoids reveals Aurora kinase upregulation after WEE1 inhibitor treatment, Figure S6: Organoids and cell lines show significant sensitivity and cell cycle arrest with Aurora kinase inhibition, Figure S7: Uterine carcinoma cell lines show cell cycle arrest issues and increased apoptosis after various treatments, Table S1: Differential expression results for DF-85 comparing vehicle to AZD1775 treatment, Table S2: Differential expression results for 19-99 comparing vehicle to AZD1775 treatment, Table S3: Differential expression results for 19-105 comparing vehicle to AZD1775 treatment.

## References

[1] Zhang L., Kwan S.Y., Wong K.K., Solaman P.T., Lu K.H., Mok S.C. (2020). Pathogenesis and Clinical Management of Uterine Serous Carcinoma. Cancers.

[2] Arend R.C., Jones B.A., Martinez A., Goodfellow P. (2018). Endometrial cancer: Molecular markers and management of advanced stage disease. Gynecol. Oncol..

[3] Hamilton C.A., Pothuri B., Arend R.C., Backes F.J., Gehrig P.A., Soliman P.T., Thompson J.S., Urban R.R., Burke W.M. (2021). Endometrial cancer: A society of gynecologic oncology evidence-based review and recommendations, part II. Gynecol. Oncol..

[4] Kandoth C., Schultz N., Cherniack A.D., Akbani R., Liu Y., Shen H., Robertson A.G., Pashtan I., Shen R., Cancer Genome Atlas Research Network (2013). Integrated genomic characterization of endometrial carcinoma. Nature.

[5] Musacchio L., Boccia S.M., Caruso G., Santangelo G., Fischetti M., Tomao F., Perniola G., Palaia I., Muzii L., Pignata S. (2020). Immune Checkpoint Inhibitors: A Promising Choice for Endometrial Cancer Patients?. J. Clin. Med..

[6] Monti P., Menichini P., Speciale A., Cutrona G., Fais F., Taiana E., Neri A., Bomben R., Gentile M., Gattei V. (2020). Heterogeneity of TP53 Mutations and P53 Protein Residual Function in Cancer: Does It Matter?. Front. Oncol..

[7] Sammons M.A., Nguyen T.-A.T., McDade S.S., Fischer M. (2020). Tumor suppressor p53: From engaging DNA to target gene regulation. Nucleic Acids Res..

[8] Lahalle A., Lacroix M., De Blasio C., Cissé M.Y., Linares L.K., Le Cam L. (2021). The p53 Pathway and Metabolism: The Tree That Hides the Forest. Cancers.

[9] Demir Ö., Barros E.P., Offutt T.L., Rosenfeld M., Amaro R.E. (2021). An integrated view of p53 dynamics, function, and reactivation. Curr. Opin. Struct. Biol..

[10] Lee E.K., Fader A.N., Santin A.D., Liu J.F. (2021). Uterine serous carcinoma: Molecular features, clinical management, and new and future therapies. Gynecol. Oncol..

[11] Romero I., Rubio M.J., Medina M., Matias-Guiu X., Santacana M., Schoenenberger J.-A., Guerra E.M., Cortés A., Minig L., Coronado P. (2020). An olaparib window-of-opportunity trial in patients with early-stage endometrial carcinoma: POLEN study. Gynecol. Oncol..

[12] Fumet J.-D., Limagne E., Thibaudin M., Truntzer C., Bertaut A., Rederstorff E., Ghiringhelli F. (2020). Precision medicine phase II study evaluating the efficacy of a double immunotherapy by durvalumab and tremelimumab combined with olaparib in patients with solid cancers and carriers of homologous recombination repair genes mutation in response or stable after olaparib treatment. BMC Cancer.

[13] Liu J.F., Xiong N., Campos S.M., Wright A.A., Krasner C., Schumer S., Horowitz N., Veneris J., Tayob N., Morrissey S. (2021). Phase II Study of the WEE1 Inhibitor Adavosertib in Recurrent Uterine Serous Carcinoma. J. Clin. Oncol..

[14] Pilié P.G., Tang C., Mills G.B., Yap T.A. (2019). State-of-the-art strategies for targeting the DNA damage response in cancer. Nat. Rev. Clin. Oncol..

[15] Di Rorà A.G.L., Cerchione C., Martinelli G., Simonetti G. (2020). A WEE1 family business: Regulation of mitosis, cancer progression, and therapeutic target. J. Hematol. Oncol..

[16] Young L.A., O’Connor L.O., De Renty C., Veldman-Jones M.H., Dorval T., Wilson Z., Jones D.R., Lawson D., Odedra R., Maya-Mendoza A. (2019). Differential Activity of ATR and WEE1 Inhibitors in a Highly Sensitive Subpopulation of DLBCL Linked to Replication Stress. Cancer Res..

[17] Fang Y., McGrail D.J., Sun C., Labrie M., Chen X., Zhang D., Ju Z., Vellano C.P., Lu Y., Li Y. (2019). Sequential Therapy with PARP and WEE1 Inhibitors Minimizes Toxicity while Maintaining Efficacy. Cancer Cell.

[18] Korsse S., Peppelenbosch M., Van Veelen W. (2013). Targeting LKB1 signaling in cancer. Biochim. Biophys. Acta (BBA) Bioenerg..

[19] Umene K., Yanokura M., Banno K., Irie H., Adachi M., Iida M., Nakamura K., Nogami Y., Masuda K., Kobayashi Y. (2015). Aurora kinase A has a significant role as a therapeutic target and clinical biomarker in endometrial cancer. Int. J. Oncol..

[20] Van Nyen T., Moiola C.P., Colas E., Annibali D., Amant F. (2018). Modeling Endometrial Cancer: Past, Present, and Future. Int. J. Mol. Sci..

[21] Ramondetta L., Mills G.B., Burke T.W., Wolf J.K. (2000). Adenovirus-mediated expression of p53 or p21 in a papillary serous endometrial carcinoma cell line (SPEC-2) results in both growth inhibition and apoptotic cell death: Potential application of gene therapy to endometrial cancer. Clin. Cancer Res..

[22] Kwan S.-Y., Au-Yeung C.-L., Yeung T.-L., Rynne-Vidal A., Wong K.-K., Risinger J.I., Lin H.-K., Schmandt R.E., Yates M.S., Mok S.C. (2020). Ubiquitin Carboxyl-Terminal Hydrolase L1 (UCHL1) Promotes Uterine Serous Cancer Cell Proliferation and Cell Cycle Progression. Cancers.

[23] Barretina J., Caponigro G., Stransky N., Venkatesan K., Margolin A.A., Kim S., Wilson C.J., Lehár J., Kryukov G.V., Sonkin D. (2012). The Cancer Cell Line Encyclopedia enables predictive modelling of anticancer drug sensitivity. Nature.

[24] Cornwell M., Vangala M., Taing L., Herbert Z., Köster J., Li B., Sun H., Li T., Zhang J., Qiu X. (2018). VIPER: Visualization Pipeline for RNA-seq, a Snakemake workflow for efficient and complete RNA-seq analysis. BMC Bioinform..

[25] Yu G., Wang L.-G., Han Y., He Q.-Y. (2012). clusterProfiler: An R Package for Comparing Biological Themes Among Gene Clusters. OMICS A J. Integr. Biol..

[26] Fuh K., Mullen M., Blachut B., Stover E., Konstantinopoulos P., Liu J., Matulonis U., Khabele D., Mosammaparast N., Vindigni A. (2020). Homologous recombination deficiency real-time clinical assays, ready or not?. Gynecol. Oncol..

[27] Hill S.J., Clark A.P., Silver D.P., Livingston D.M. (2014). BRCA1 Pathway Function in Basal-Like Breast Cancer Cells. Mol. Cell. Biol..

[28] Hill S.J., Decker B., Roberts E.A., Horowitz N.S., Muto M.G., Worley M.J., Feltmate C.M., Nucci M.R., Swisher E.M., Nguyen H. (2018). Prediction of DNA Repair Inhibitor Response in Short-Term Patient-Derived Ovarian Cancer Organoids. Cancer Discov..

[29] Nieminuszczy J., Schwab R.A., Niedzwiedz W. (2016). The DNA fibre technique—Tracking helicases at work. Methods.

[30] Hafner M., Niepel M., Chung M., Sorger P.K. (2016). Growth rate inhibition metrics correct for confounders in measuring sensitivity to cancer drugs. Nat. Methods.

[31] Takeuchi M., Tanikawa M., Nagasaka K., Oda K., Kawata Y., Oki S., Agapiti C., Sone K., Miyagawa Y., Hiraike H. (2019). Anti-Tumor Effect of Inhibition of DNA Damage Response Proteins, ATM and ATR, in Endometrial Cancer Cells. Cancers.

[32] Saldivar J.C., Cortez D., Cimprich K.A. (2017). The essential kinase ATR: Ensuring faithful duplication of a challenging genome. Nat. Rev. Mol. Cell Biol..

[33] Pfister S.X., Markkanen E., Jiang Y., Sarkar S., Woodcock M., Orlando G., Mavrommati I., Pai C.-C., Zalmas L.-P., Drobnitzky N. (2015). Inhibiting WEE1 Selectively Kills Histone H3K36me3-Deficient Cancers by dNTP Starvation. Cancer Cell.

[34] Lok W., Klein R.Q., Saif M.W. (2010). Aurora kinase inhibitors as anti-cancer therapy. Anti-Cancer Drugs.

[35] Du R., Huang C., Liu K., Li X., Dong Z. (2021). Targeting AURKA in Cancer: Molecular mechanisms and opportunities for Cancer therapy. Mol. Cancer.

[36] Galetta D., Cortes-Dericks L. (2020). Promising Therapy in Lung Cancer: Spotlight on Aurora Kinases. Cancers.

[37] Daver N., Wei A.H., Pollyea D.A., Fathi A.T., Vyas P., Dinardo C.D. (2020). New directions for emerging therapies in acute myeloid leukemia: The next chapter. Blood Cancer J..

[38] Lee J.W., Parameswaran J., Sandoval-Schaefer T., Eoh K.J., Yang D.-H., Zhu F., Mehra R., Sharma R., Gaffney S.G., Perry E.B. (2019). Combined Aurora Kinase A (AURKA) and WEE1 Inhibition Demonstrates Synergistic Antitumor Effect in Squamous Cell Carcinoma of the Head and Neck. Clin. Cancer Res..

[39] Falchook G., Coleman R.L., Roszak A., Behbakht K., Matulonis U., Ray-Coquard I., Sawrycki P., Duska L.R., Tew W., Ghamande S. (2019). Alisertib in Combination With Weekly Paclitaxel in Patients With Advanced Breast Cancer or Recurrent Ovarian Cancer. JAMA Oncol..

[40] Manu K.A., Cao P.H.A., Chai T.F., Casey P.J., Wang M. (2019). p21cip1/waf1 Coordinates Autophagy, Proliferation and Apoptosis in Response to Metabolic Stress. Cancers.

[41] El-Deiry W.S. (2016). p21(WAF1) Mediates Cell-Cycle Inhibition, Relevant to Cancer Suppression and Therapy. Cancer Res..

[42] Peña C.G., Castrillón D.H. (2017). LKB1 as a Tumor Suppressor in Uterine Cancer: Mouse Models and Translational Studies. Tissue Eng..

[43] Zheng X., Chi J., Zhi J., Zhang H., Yue D., Zhao J., Li D., Li Y., Gao M., Guo J. (2017). Aurora-A-mediated phosphorylation of LKB1 compromises LKB1/AMPK signaling axis to facilitate NSCLC growth and migration. Oncogene.

[44] Asaka R., Miyamoto T., Yamada Y., Ando H., Mvunta D.H., Kobara H., Shiozawa T. (2015). Sirtuin 1 promotes the growth and cisplatin resistance of endometrial carcinoma cells: A novel therapeutic target. Lab. Investig..

[45] Konopleva M., Martinelli G., Daver N., Papayannidis C., Wei A., Higgins B., Ott M., Mascarenhas J., Andreeff M. (2020). MDM2 inhibition: An important step forward in cancer therapy. Leukemia.

[46] Gupta A., Shah K., Oza M.J., Behl T. (2019). Reactivation of p53 gene by MDM2 inhibitors: A novel therapy for cancer treatment. Biomed. Pharmacother..

[47] Hahn W.C., Dessain S.K., Brooks M.W., King J.E., Elenbaas B., Sabatini D.M., DeCaprio J.A., Weinberg R.A. (2002). Enumeration of the Simian Virus 40 Early Region Elements Necessary for Human Cell Transformation. Mol. Cell. Biol..

[48] Uko N.E., Güner O.F., Matesic D.F., Bowen J.P. (2020). Akt Pathway Inhibitors. Curr. Top. Med. Chem..

[49] Roncolato F., Lindemann K., Willson M.L., Martyn J., Mileshkin L. (2019). PI3K/AKT/mTOR inhibitors for advanced or recurrent endometrial cancer. Cochrane Database Syst. Rev..

[50] Marxer M., Ma H.T., Man W.Y., Poon R.Y.C. (2013). p53 deficiency enhances mitotic arrest and slippage induced by pharmacological inhibition of Aurora kinases. Oncogene.

