# Supplementary material for: Enhanced Efficacy of Aurora Kinase Inhibitors in G2/M Checkpoint Deficient TP53 Mutant Uterine Carcinomas Is Linked to the Summation of LKB1–AKT–p53 Interactions
Source: Cancers (Basel). 2021 May 3;13(9):2195. doi: 10.3390/cancers13092195 (PMC8125555; doi:10.3390/cancers13092195)
Supplement: Supplementary file 1 [file cancers-13-02195-s001.zip › Lynch and Hill Supplementary Matierals/Lynch and Hill Supplementary Text and Figures 3-27-21.pdf]

SUPPLEMENTARY MATERIALS

Included here are Supplementary materials and methods, followed by Supplementary figure and table legends, followed by Supplementary references, followed by seven Supplementary figures. Supplementary Tables are provided as separate Excel files.

SUPPLEMENTARY MATERIALS AND METHODS

Organoid generation and culture: All organoid lines tested negative for mycoplasma by PCR. Tissue processing, organoid generation, and organoid culture were performed as described previously [1]. Culture media consisted of 50% WNT conditioned media [2]/50% Advanced DMEM/F-12 (Invitrogen Cat. #12634010) supplemented with 1% penicillin streptomycin, 1X Glutamax (Life Technologies Cat. # 35050061), 1% HEPES (Life Technologies Cat. #15630080), 100 ng/mL R-spondin 1 (Peprotech Cat. # 120-38), 100 ng/mL Noggin (Peprotech Cat. # 120-10C), 50ng/mL EGF (Peprotech Cat. #100-15), 10ng/mL FGF-10 (Peprotech Cat. # 100-26), 10 ng/mL FGF2 (Peprotech Cat. # 100-18B), 1X B27 (Life Technologies Cat. #17504044), 10mM Nicotinamide (Sigma Aldrich Cat. # N0636), 1.25mM N-acetylcysteine (Sigma Aldrich Cat. # A9165), 1uM Prostaglandin E2 (R&D Systems Cat. # 2296), 10uM SB202190 (Sigma Aldrich Cat. # S7076), and 500nM A8301 (Sigma Aldrich Cat. # SML0788).

Cell line culture: All cell lines tested negative for mycoplasma by PCR. Cell lines were obtained either from ATCC or via MTA for those not commercially available. ARK1 and ARK2 cells were grown in RPMI (Life Technologies #11875-093), 10% FBS (Sigma Cat. #F2442), and 1% penicillin streptomycin. MFE 280, SPEC2, HEC1B, and AN3CA cells were grown in MEM (Corning Cat. #10-010-CV), 10% FBS, 1% penicillin streptomycin, 1% sodium pyruvate (Life

Technologies Cat. #11360-070), 1% Non-essential amino acids (Life Technologies Cat. #11140-050), and 2% vitamins (Life Technologies Cat. #11120-052). Human mammary epithelial cells were cultured as described previously [3].

ModelSeq: gDNA was fragmented to 200bp on a Covaris M220 instrument according to the manufacturer's protocol. Libraries were prepared using Swift S2 Acel reagents on a Beckman Coulter Biomek i7 liquid handling platform from approximately 100ng of DNA according to manufacturer's protocol with 14 cycles of PCR amplification. Finished libraries were quantified by Qubit fluorometer and fragment size distribution was evaluated by Agilent TapeStation 2200. Eight libraries were combined to a total amount of 1500ng (187.5 ng/library) and dried down in a vacuum concentrator with no heat. Dried indexed library pools were resuspended using Twist Biosciences reagents and hybrid capture was performed with a 16 hour hybridization incubation using custom ModelSeq probes according to the manufacturer's protocol. Post-capture library pools were quantified by Qubit fluorometer and Agilent TapeStation 2200. Library pools were further evaluated for quality and pool balance with shallow sequencing on an Illumina MiSeq. Subsequently, libraries were sequenced on an Illumina NovaSeq6000 targeting 50 million 100bp read pairs by the Molecular Biology Core facilities at Dana-Farber Cancer Institute. DNA variant data were derived using a standard pipeline established by the Center for Patient Derived Models for PDX and cell line models. The 'raw' data were mapped to hg38 human reference genome to produce final alignment files in BAM format. Final hg19 BAM files were coordinate sorted by Samtools-1.7. Duplicate reads were marked and removed from the BAM files using Picard-2.0.1. Variant calling was performed using GATK-4.1.0.0/Mutect2 pipeline with the default parameters and filters except with the following modifications: (i) 'af-

of-alleles-not-in-resource' was set to 0 (ii) 'MateOnSameContigOrNoMappedMateReadFilter' was disabled and (iii) a germline resource from the gnomAD database was included. The capture target intervals used for Mutect2 were POPv2 and POPv2 for cohorts one and two respectively. Further, a Panel of Normals was created for cohort two using three "CASE" samples provided. The generated variant calls were further filtered using FilterMutectCalls module of GATK4 and the final output in VCF format was annotated with Ensemble Variant Effect Predictor (ensembl-vep-96.0) using vcf2maf-1.6.16 [4]. The calls were additionally annotated with OncoKB dataset using oncokb-annotator and sorted as MAF files.

Ultra-low pass whole genome sequencing for copy number analysis: Whole genome sequencing libraries were prepared using SeqWell plexWell reagents to enzymatically fragment, index, and normalize large batches of DNA samples from input amounts ranging from 5ng to 25ng. Libraries were sequenced to a target depth of 0.1x coverage with paired-end reads. R package ichorCNA was used to obtain segmented copy number data after adjusting for tumor purity and ploidy. The R package CNTools was used to derive a reduced segment matrix, combining all breakpoints in the sample set into a set of common segments.

Cell cycle flow cytometry analysis: Cells were plated at 50% confluence the night before and then treated with appropriate drugs the next day. For AZD1775 alone, cells were treated with 0.5uM AZD1775 for various timepoints. For AZD1775 and Alisertib combination experiments, cells were treated with vehicle, 0.5uM AZD1775, 0.25uM Alisertib, or 0.5uM AZD1775+0.25uM Alisertib. For Barasertib or MK5108 experiments, cells were treated with 0.25uM drug or vehicle. One hour prior to harvest, 10uM BrdU (Biolegend Cat. # 423401) was added to the cells. At the appropriate timepoint, cells were trypsinized, washed with PBS, and

fixed in ice cold 70% ethanol. Cells were stored at -20°C until ready for staining. At the appropriate time, cells were centrifuged, ethanol aspirated, washed in 0.5% Tween-PBS (PBS-T), centrifuged, PBS-T aspirated, and fixed in fresh 2N HCl for 15 minutes at room temperature. Cells were washed in PBS-T, and then cells were incubated in 0.1M Na2B407 pH 8.5 in BSA for 30 minutes at room temperature. Cells were centrifuged, sodium borate was aspirated, and then cells were incubated in FITC-anti-BrdU (BD Cat. # 556028) in 1% BSA at room temperature in the dark for 30 minutes. Cells were washed in PBS-T, and then PI/RNASE running buffer (BD Cat. # 550825) was added and the cells were analyzed on a BD LSR Fortessa Flow Cytometer.

DNA Fiber Assays: DNA fiber assays were performed on organoids and cell lines as described previously [1]. 0.1mM hydroxyurea was used as the drug treatment.

RAD51 focus formation assay: For cell lines, cells were plated onto coverslips (Electron Microscopy Sciences Cat. #72228-01) the night before at 70% confluence. Organoids were scraped from the wells into a 1.5mL Eppendorf tube. The cells on coverslips or organoids in tubes were then treated with either 0Gy or 5Gy and allowed to recover at 37°C for 8hr. Cell lines were fixed in wells with 4% paraformaldehyde (PFA, Electron Microscopy Sciences Cat. #15710-S). Organoids were spun onto slides using cytofunnels (Fisher Cat. #5991039) and a cytospin and then fixed with 4% PFA. Cells were then permeabilized with 0.5% Triton-X-100, washed in PBS, and stained in primary antibody mixture for 30 minutes at 37°C. Cells were washed in PBS and then stained in secondary antibody mixture for 30 minutes at 37°C. Cells were washed in PBS, mounted in DAPI, and viewed and photographed. Primary antibodies included RAD51 (Santa Cruz Cat. #sc-8349) and γH2AX (Millipore Cat. #05-636). Secondary

antibodies included 488 anti-rabbit (Abcam Cat. #150077) and 594 anti-mouse (Abcam Cat. #150116). Nuclei were photographed, and the number of nuclei containing greater than or equal to three co-localizing RAD51/γH2AX foci were counted from at least 100 nuclei per replicate. Two replicates were performed per line.

Drug treatments for dose curves: Organoids or cell lines were plated in 96 well plates (Fisher Cat. # 165305) in drug containing cell line or organoid media as described previously [1]. Media with drug was added to cells on day one and left on for five days at which point Cell Titer-Glo analysis was performed. Except for Carboplatin which was obtained in solution from the Dana-Farber pharmacy, all drugs were resuspended in DMSO. Dose curves were as follows:

Alisertib (MedChemExpress Cat. #HY-10971): 0uM (vehicle at highest DMSO volume), 0.001uM, 0.005uM, 0.01uM, 0.025uM, 0.05uM, 0.5uM, 1uM.

Olaparib (Selleck Cat. #S1060): 0uM (vehicle at highest DMSO volume), 0.005uM, 0.05uM, 0.1uM, 1uM, 2.5uM, 5uM, 10uM.

Carboplatin (Teva NDC 0703-4248-01): Cell lines 0uM (vehicle at highest ddH20 volume), 0.05uM, 0.5uM, 5uM, 10uM, 20uM, 30 uM. Organoids 0uM (vehicle at highest ddH20 volume), 0.5uM, 5uM, 10uM, 20uM, 30 uM, 50uM, 75uM.

AZD1775 (Selleck Cat. #S1525): 0uM (vehicle at highest DMSO volume), 0.005uM, 0.01uM, 0.05uM, 0.1uM, 0.5 uM, 1uM.

Gemcitabine (Selleck Cat. #S1714): 0uM (vehicle at highest DMSO volume), 0.001uM, 0.005uM, 0.01uM, 0.05uM, 0.5uM, 1uM.

AZD6738 (Selleck Cat. #S7693): 0uM (vehicle at highest DMSO volume), 0.005uM, 0.01uM, 0.05uM, 0.5uM, 1uM, 2.5uM.

Paclitaxel (Selleck Cat. #S1150): 0uM (vehicle at highest DMSO volume), 0.001uM, 0.005uM, 0.01uM, 0.05uM, 0.5uM, 1uM.

Barasertib (MedChemExpress Cat. #HY-10126): 0uM (vehicle at highest DMSO volume), 0.001uM, 0.005uM, 0.01uM, 0.025uM, 0.05uM, 0.5uM, 1uM.

MK5108 (MedChemExpress Cat. #HY-13252): 0uM (vehicle at highest DMSO volume), 0.001uM, 0.005uM, 0.01uM, 0.025uM, 0.05uM, 0.5uM, 1uM.

Nutlin-3 (Selleck Cat. #S1061): 0uM (vehicle at highest DMSO volume), 0.005uM, 0.05uM, 0.5uM, 1uM, 5uM, 10uM, 20uM.

For Alisertib combined with Nutlin-3 experiments, Alisertib was plated at a dose range of 0uM (vehicle at highest DMSO volume), 0.001uM, 0.005uM, 0.01uM, 0.025uM, 0.05uM, 0.1uM, 0.5uM combined with a fixed dose of either vehicle or 5uM Nutlin-3.

For siRNA or p53DD experiments, Alisertib was plated at a dose range of 0uM (vehicle at highest DMSO volume), 0.001uM, 0.005uM, 0.01uM, 0.025uM, 0.05uM, 0.1uM, 0.5uM.

For Ipatasertib (MedChemExpress Cat. #HY-15186)/Alisertib combination experiments in Figure 4B, cells were treated with either Alisertib alone, Ipatasertib alone, or Alisertib+Ipatasertib combined at the following dose range: 0uM (vehicle at highest DMSO volume), 0.001uM, 0.005uM, 0.01uM, 0.025uM, 0.05uM, 0.1uM, 0.5uM.

For AZD1775/Alisertib combination experiments in Figure 4C, cells were treated with either Alisertib, AZD1775, or Alisertib+AZD1775 combined at the following dose range: 0uM

(vehicle at highest DMSO volume), 0.001uM, 0.005uM, 0.01uM, 0.025uM, 0.05uM, 0.1uM, 0.5uM.

On day zero of each experiment, between four to eight wells of untreated cells were treated with Cell Titer-Glo (Promega Cat. #G7572) and read on a BMG LabTech CLARIOStar plate reader as a growth rate normalization control. At the end of each experiment (day 5) cells were treated with Cell Titer-Glo and read on the plate reader. Growth rate corrected GR50s and area over the curve (AOCs) were calculated as previously described in Excel and GraphPad Prism [5]. The sensitivity shown in the figures represents the area over the growth rate corrected dose curves.

Histone 3 phospho-Serine 10 (H3pS10) Immunofluorescence: Cells were plated onto coverslips the night before at 70% confluence. Cells were treated with vehicle, 0.5uM AZD1775, 0.25uM Alisertib, or 0.5uM AZD1775+0.25uM Alisertib for 24 hours. Cells were fixed in wells with 4% paraformaldehyde. Cells were then permeabilized with 0.5% Triton-X-100, washed in PBS, and stained in primary antibody mixture for 30 minutes at 37°C. Cells were washed in PBS and then stained in secondary antibody mixture for 30 minutes at 37°C. Cells were washed in PBS, mounted in DAPI, viewed, and photographed. Primary antibodies included H3 phospho S10 (Cell Signaling Cat. #3377T) and Tubulin (Sigma Cat. #T5168). Secondary antibodies included 488 anti-rabbit (Abcam Cat. #150077) and 594 anti-mouse (Abcam Cat. #150116). Nuclei were photographed, and the number of normal nuclei, mitotic figures, mitotic catastrophes, and apoptotic blebs were counted from at least 100 nuclei for each of three replicates per cell line.

siRNA transfection: Cells were plated at 40% confluence on day 0. On days 1 and 2, cells were transfected with 20pmol of each siRNA using Lipofectamine RNAiMax (Life Technologies Cat. #13778075) and Optimem (Life Technologies Cat. #31985088). Cells were harvested or plated for additional assays on day 3. siRNAs included the following: siControl (Qiagen AllStars Negative Control Cat. #1027280), siLKB1 #1 (Dharmacon Cat. #D-005035-01-0002, UGAAAGGGAUGCUUGAGUA), siLKB1 #6 (Cat. #D-005035-06-0002, GCAUGACUGUGGUGCCGUA), siCDKN1A #1 (Dharmacon Cat. # D-003471-01-0002, GAUGGAACUUCGACUUUGU), siCDKN1A #4 (Dharmacon Cat. #D-003471-04-0002, CGACUGUGAUGCGCUAAUG) sip53 #5 (Dharmacon Cat. #D-003329-05-0002, GAGGUUGGCUCUGACUGUA), and sip53 #7 (Dharmacon Cat. #D-003329-07-0002, GCACAGAGGAAGAGAAUCU).

Generation of p53DD expressing HEC1Bs: HEC1B cells were transfected with either pBABE vector or p53DD containing pBABE [6] using Optimem and Lipofectamine 2000 (Life Technologies Cat. #11668027). Cells were allowed to recover for 24 hours and then G418 (Sigma Cat. #G8168) was added to the media to select plasmid containing cells. Expression was confirmed by western blot.

Western Blots: Cell or organoid pellets were harvested after treatments as indicated in each figure legend and resuspended in NETN300 lysis buffer (300mM NaCl, 50mM Tris pH 7.5, 1mM EDTA, 0.5% NP40, 10% Glycerol) containing EDTA-free protease inhibitor (Roche Cat. #04693132001). Cells were lysed with rocking at 4°C for at least fifteen minutes and then spun at maximum speed in a tabletop microcentrifuge for 15 minutes at 4°C. The supernatant was

saved, and the protein concentration obtained using BioRad Bradford reagent (Cat. #5000201). Lysates were normalized to the same concentration using Laemmli loading buffer (Boston Bioproducts Cat. #BP-110NR) with β-mercaptoethanol. Lysates were run in 4-12% Bis-Tris gels (Invitrogen Cat. #NP0336BOX) in MOPS (Invitrogen Cat. #NP0001) and transferred to 0.45um Nitrocellulose membranes (Amersham Cat. #10600002). Membranes were blotted with the appropriate primary antibody from the table below, blotted with appropriate HRP conjugated rabbit (Cell Signaling Cat. #7074S) or mouse (Cell Signaling Cat. #7076S) secondaries and developed using Biolegend's Western Ready ECL Substrate Premium Solution (Cat. #426319). Primary antibodies included the list in the below table.

**Antibody**	**Vendor**	**Catalog #**
pLKB1 Ser 428	Cell Signaling	3482S
LKB1	Cell Signaling	3047S
pAKT Ser 473	Cell Signaling	4060T
pAKT Thr 308	Cell Signaling	2965S
p53	Santa Cruz	sc-6243
AKT	Cell Signaling	2920S
p21	Cell Signaling	2946T
Vinculin	Santa Cruz	sc-25336
Cleaved PARP	Biolegend	669901
Tubulin	Sigma	T-5168
Phospho-p53 Ser 15	Cell Signaling	9284S
H3PS10	Biolegend	650801
CHK1	Santa Cruz	sc-8408
pKAP1	Cell Signaling	4127S
pCDC2 Tyr 15	Cell Signaling	4539S
pCHK1 Ser 345	Cell Signaling	2341S
RRM2	Abnova	H00006241-M01
pRPA	Novus	NB100544
CDC2	Cell Signaling	9116S
ATR	Cell Signaling	13934S

H3pS10 flow cytometry: Cells were plated at 50% confluence on day 0. On day one cells were treated with either vehicle or 0.5uM AZD1775 and harvested at various timepoints. For harvest, cells were trypsinized, washed in Biolegend's Cell Stain Buffer (Cat. #420201), and then incubated in Cell Stain Buffer with Zombie NIR dye (Biolegend Cat. #423105) for 20 minutes at room temperature. Cells were washed in Cell Stain buffer and fixed for 20 minutes at room temperature in Biolegend's FluoroFix Buffer (Cat. #422101). Cells were washed in Biolegend's Intracellular Staining Permeabilization Wash Buffer (Cat. #421002), and then incubated for 20 minutes at room temperature in permeabilization wash buffer with APC-H3pS10 antibody (Biolegend Cat. # 650805). Cells were washed with permeabilization wash buffer, resuspended in cell stain buffer, and analyzed on a BD LSR Fortessa.

Histology: Organoid embedding, and organoid and parent tumor PAX8 (Proteintech Cat# 10336-1-AP) and p53 (Agilent Technologies Cat. # M7001) immunohistochemical staining was performed as described previously [1].

Apotracker flow: Cells were plated at 50% confluence on day 0. On day one, cells were treated with vehicle, 0.5uM AZD1775, 0.25uM Alisertib, or 0.5uM AZD1775+0.25uM Alisertib. After 24 hours cells were trypsinized, washed in Biolegend's Cell Stain Buffer (Cat. #420201), and incubated in Biolegend's Cell Stain Buffer with Zombie NIR dye (Biolegend Cat. #423105) and Apotracker dye (Biolegend Cat. #427401) for 20 minutes at room temperature. Cells were washed once in cell stain buffer and then immediately analyzed on a BD LSR Fortessa.

Cartoon generation: Cartoons in Figures 3A and 6 were created with BioRender.com.

SUPPLEMENTARY FIGURE LEGENDS

Figure S1. Uterine carcinoma patient-derived organoid model properties. A) Table demonstrating the site from which malignant tissue or fluid was obtained for organoid generation and the treatment status of the patient at the time of the procedure. **B)** Hematoxylin and Eosin **(**H&E) stains of a matched parent tumor (top left) and organoid (top right) and PAX8 stains of the same matched parent tumor (bottom left) and organoid (bottom right).

Figure S2. DNA damage repair defect profiling in a panel of *TP53* mutant uterine carcinoma models does not reveal any common functional defects. A) Cell lines and organoids were treated with 0 or 5Gy gamma irradiation and 8 hours later the number of nuclei with >/= 3 co-localizing γH2AX and RAD51 foci were counted. On the left are representative images of groups of untreated nuclei (top panels), nuclei 8hr post 5Gy (middle panels), and a magnification of one nucleus 8hr post 5Gy demonstrating co-localizing foci (bottom panels). On the right are bar graphs demonstrating the number of nuclei with >/= 3 co-localizing foci for each cell line or organoid analyzed with or without damage. Bars represent the average of two replicates, and error bars represent standard deviation between replicates. **B)** DNA fiber analysis was performed on each organoid and cell line with or without hydroxyurea treatment. On the left, representative control or HU degraded fibers are shown. In the middle a representative count of the ratio of CldU to IdU with or without HU is shown for organoid line 20-18. On the far right is a table of the average CldU/IdU ratio with or without HU for each cell or organoid line.

Figure S3. Dose curves for all lines with all agents. Organoids and cell lines were treated with Alisertib, Olaparib, Gemcitabine, Carboplatin, AZD1775, Paclitaxel, or AZD6738. The dose curves were growth rate corrected, and a representative growth rate corrected GR50 dose curve is shown here for each line for each drug. The key to the lines is on the right of each graph.

Figure S4. *TP53* mutant uterine carcinomas demonstrate cell cycle checkpoint and not DNA damage repair defects. A) A subset of cell lines were treated with 0.1uM AZD1775 (1775) for 48 hours and harvested at varying intervals compared to a 0 hour control (con) for western blot analysis of DNA damage (γH2AX, ATR, pKAP1, pCHK1, pRPA) and cell cycle (pCDC2 and RRM2) proteins over time with Vinculin as a loading control. **B)** A subset of cell lines were treated with vehicle or 0.5uM AZD1775 and analyzed for histone H3 phospho-serine 10 (H3pS10) expression at various timepoints post treatment via flow cytometry. Bars represent the average percent of viable H3pS10 positive cells at each timepoint and error bars represent the standard deviation between three replicates. **C)** WEE1 inhibitor strongly sensitive (19-105), mildly sensitive (DF-85) and resistant (19-99) organoids were treated with 1uM AZD1775 for 24 hours and analyzed for DNA damage and cell cycle protein expression at varying timepoints by western blot compared to a 0 hour control (con).

Figure S5. Bulk RNA sequencing of organoids reveals Aurora kinase upregulation after WEE1 inhibitor treatment. Select organoid lines were treated with either vehicle or AZD1775 and submitted for bulk RNA sequencing 14 hours after treatment. The gene ontology (GO) overrepresentation analysis maps and volcano plots of the comparison between vehicle and AZD1775 treatment are shown here for **A)** 19-99 and **B)** 19-105. For GO term plots, GO terms

are listed on the Y-axis, circles represent the number of genes in the GO term group, and the X-axis represents increasing significance. For Volcano plots, p-values of increasing significance are shown on the Y-axis and increasing Log2fold change is shown on the X-axis. A black circle indicates genes with non-significant (NS) change, a blue circle indicates significantly downregulated genes, and a red circle indicates significantly upregulated genes.

Figure S6. Organoids and cell lines show significant sensitivity and cell cycle arrest with Aurora kinase inhibition. A) Comparison of sensitivities for all cell lines and organoids between AZD1775 and Alisertib. **B)** A subset of cell lines were treated with 0.25uM MK5108 or Barasertib and harvested over a 24 hour period and subjected to BrdU-PI cell cycle flow cytometry analysis. The percentage of cells in G0/G1 (black), S (grey), or G2/M (white) are shown here for two to three replicates at each timepoint and treatment for these cell lines. Bars represent average percentage of cells in each phase, and error bars represent standard deviation between replicates. **C)** A subset of cell lines were tested for sensitivity to MK5108 or Barasertib. Each sensitivity curve was run twice, and all lines were growth rate corrected. The sensitivity (area over the growth rate corrected dose curve) for MK5108 and Barasertib compared to Alisertib is shown here with two replicates for each line with bars representing the average sensitivity and error bars representing the standard deviation between two replicates.

Figure S7. Uterine carcinoma cell lines show cell cycle arrest issues and increased apoptosis after various treatments. A) Four cell lines were treated with either 0.25uM Alisertib, 0.5uM AZD1775 (1775), or a combination of Alisertib and AZD1775 and cells harvested at 3, 8, or 24 hours post treatment compared to an untreated control (0 hr). Lysates from the cells were blotted

for DNA damage proteins including pKAP1, pCHK1, pRPA, and γH2AX and cell cycle proteins including pCDC2 and H3pS10. Vinculin was used as a loading control. **B)** AN3CA, ARK1, and SPEC2 were treated with vehicle or either 0.25uM Alisertib, 0.5uM AZD1775, or a combination of Alisertib and AZD1775 and harvested 24 hours post treatment for apoptosis flow cytometry using Apotracker. The percentage of dead apoptotic cells for each treatment is shown for three to four replicates. Bars represent averages of three to four replicates, and error bars represent standard error of the mean. t-tests were used to calculate p-values, and a * above a bar indicates significance compared to the DMSO control. *$p<0.05$. **C)** ARK1 cells were treated with vehicle (untreated) or either 0.25uM Alisertib, 0.5uM AZD1775, or a combination of Alisertib and AZD1775, harvested at various times post treatment, and analyzed by western blot for cleaved PARP and vinculin as a control.

SUPPLEMENTARY TABLE LEGENDS

Table S1. Differential expression results for DF-85 comparing vehicle to AZD1775 treatment: Differential expression results from VIPER analysis comparing two replicates each of vehicle versus AZD1775 treated organoids (DF-85). The gene ID, log2foldchange after treatment, adjusted p-value comparing control to treated cells, Ensemble ID, Entrez Gene ID, Gene description, associated GO IDs, and associated GO terms are shown.

Table S2. Differential expression results for 19-99 comparing vehicle to AZD1775 treatment: Differential expression results from VIPER analysis comparing two replicates each of vehicle versus AZD1775 treated organoids (19-99). The gene ID, log2foldchange after

treatment, adjusted p-value comparing control to treated cells, Ensemble ID, Entrez Gene ID, Gene description, associated GO IDs, and associated GO terms are shown.

Table S3. Differential expression results for 19-105 comparing vehicle to AZD1775 treatment: Differential expression results from VIPER analysis comparing two replicates each of vehicle versus AZD1775 treated organoids (19-105). The gene ID, log2foldchange after treatment, adjusted p-value comparing control to treated cells, Ensemble ID, Entrez Gene ID, Gene description, associated GO IDs, and associated GO terms are shown.

SUPPLEMENTARY REFERENCES

1. Hill, S.J.; Decker, B.; Roberts, E.A.; Horowitz, N.S.; Muto, M.G.; Worley, M.J., Jr.; Feltmate, C.M.; Nucci, M.R.; Swisher, E.M.; Nguyen, H.; et al. Prediction of DNA Repair Inhibitor Response in Short-Term Patient-Derived Ovarian Cancer Organoids. *Cancer Discov* **2018**, *8*, 1404-1421, doi:10.1158/2159-8290.CD-18-0474.
2. Willert, K.; Brown, J.D.; Danenberg, E.; Duncan, A.W.; Weissman, I.L.; Reya, T.; Yates, J.R., 3rd; Nusse, R. Wnt proteins are lipid-modified and can act as stem cell growth factors. *Nature* **2003**, *423*, 448-452, doi:10.1038/nature01611.
3. Wang, H.; Xiang, D.; Liu, B.; He, A.; Randle, H.J.; Zhang, K.X.; Dongre, A.; Sachs, N.; Clark, A.P.; Tao, L.; et al. Inadequate DNA Damage Repair Promotes Mammary Transdifferentiation, Leading to BRCA1 Breast Cancer. *Cell* **2019**, *178*, 135-151 e119, doi:10.1016/j.cell.2019.06.002.

4. Nicorici D, S.M., Edgren H, Kangaspeska S, Murumagi A, Kallioniemi O, Virtanen S, Kilkku O. FusionCatcher – a tool for finding somatic fusion genes in paired-end RNA-sequencing data. *bioRxiv* **2014**, doi:https://doi.org/10.1101/011650.
5. Hafner, M.; Niepel, M.; Chung, M.; Sorger, P.K. Growth rate inhibition metrics correct for confounders in measuring sensitivity to cancer drugs. *Nat Methods* **2016**, *13*, 521-527, doi:10.1038/nmeth.3853.
6. Hahn, W.C.; Dessain, S.K.; Brooks, M.W.; King, J.E.; Elenbaas, B.; Sabatini, D.M.; DeCaprio, J.A.; Weinberg, R.A. Enumeration of the simian virus 40 early region elements necessary for human cell transformation. *Mol Cell Biol* **2002**, *22*, 2111-2123, doi:10.1128/mcb.22.7.2111-2123.2002.

Figure S1

A

Organoid/Patient ID	Site	Treatment Status
19-78	Uterine primary	Untreated
19-70	Uterine primary	Untreated
DF-85	Ascites	Recurrence after multiple therapies
20-18	Omental metastasis	Recurrence after multiple therapies
19-99	Uterine primary	Untreated
19-105	Ascites	Recurrence after multiple therapies

B

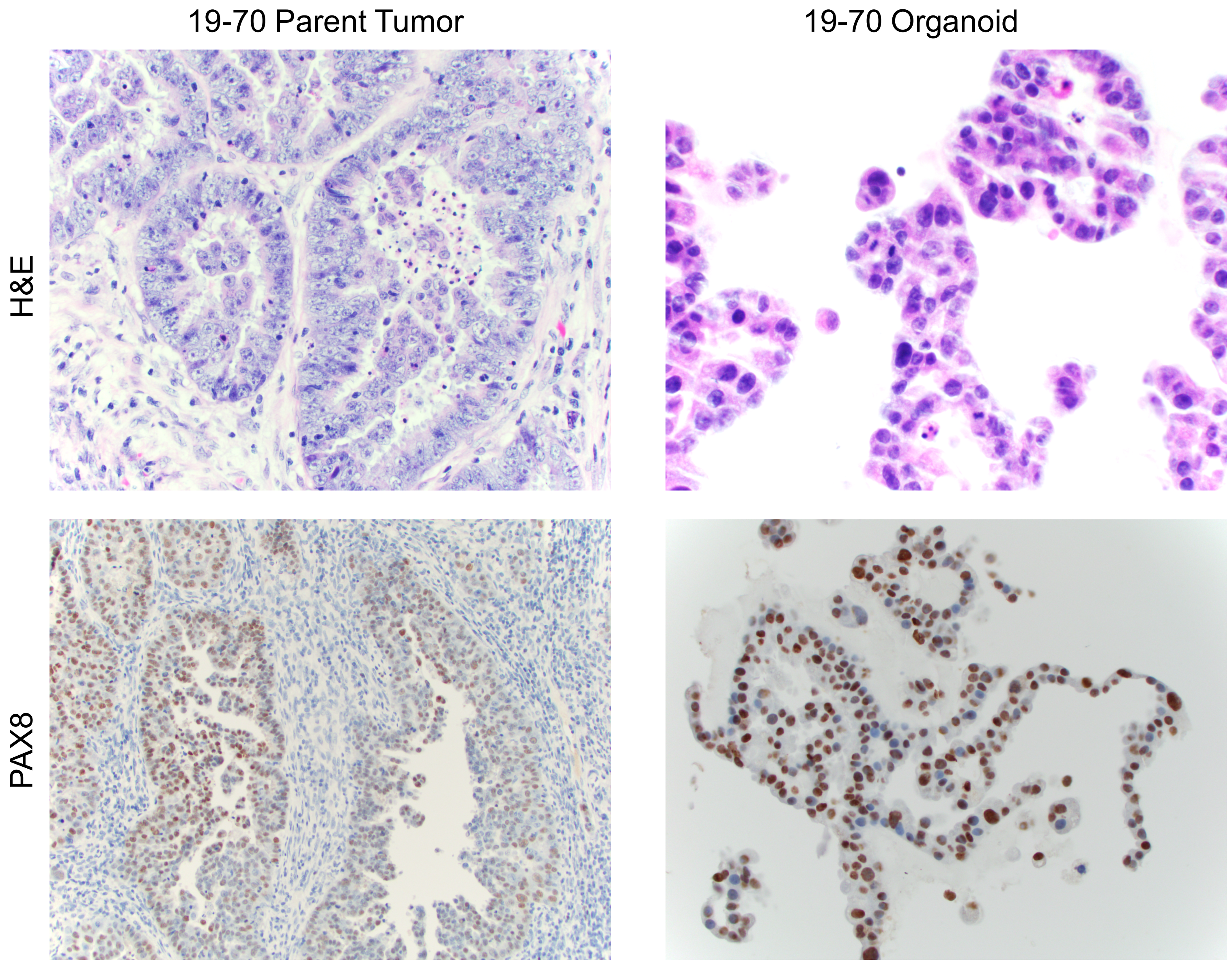

Figure S2

A

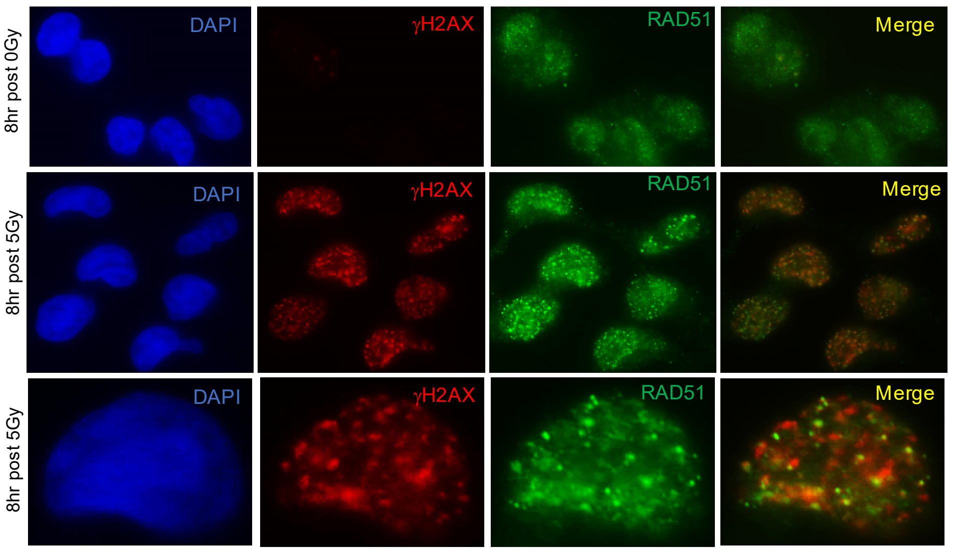

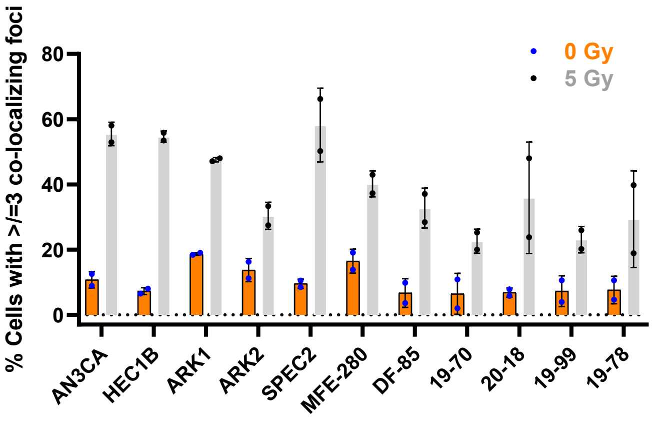

B

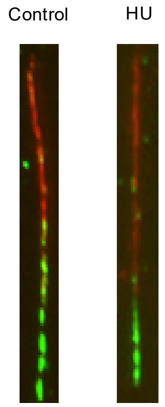

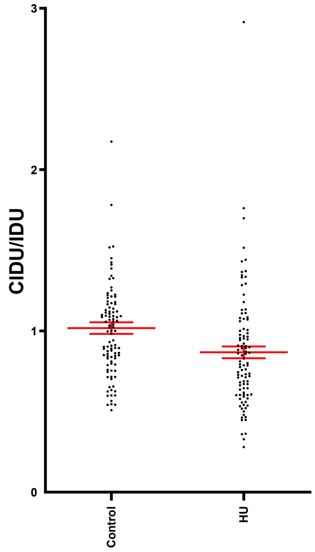

Line	Control	HU
AN3CA	1.25	1.16
HEC1B	1.02	0.80
ARK1	1.06	0.89
ARK2	1.07	0.91
SPEC2	1.19	0.71
MFE-280	0.95	0.85
DF-85	0.97	0.86
19-70	0.96	0.83
19-99	1.01	0.92
19-105	1.56	0.95
20-18	1.02	0.87
19-78	1.13	1.08

Figure S3

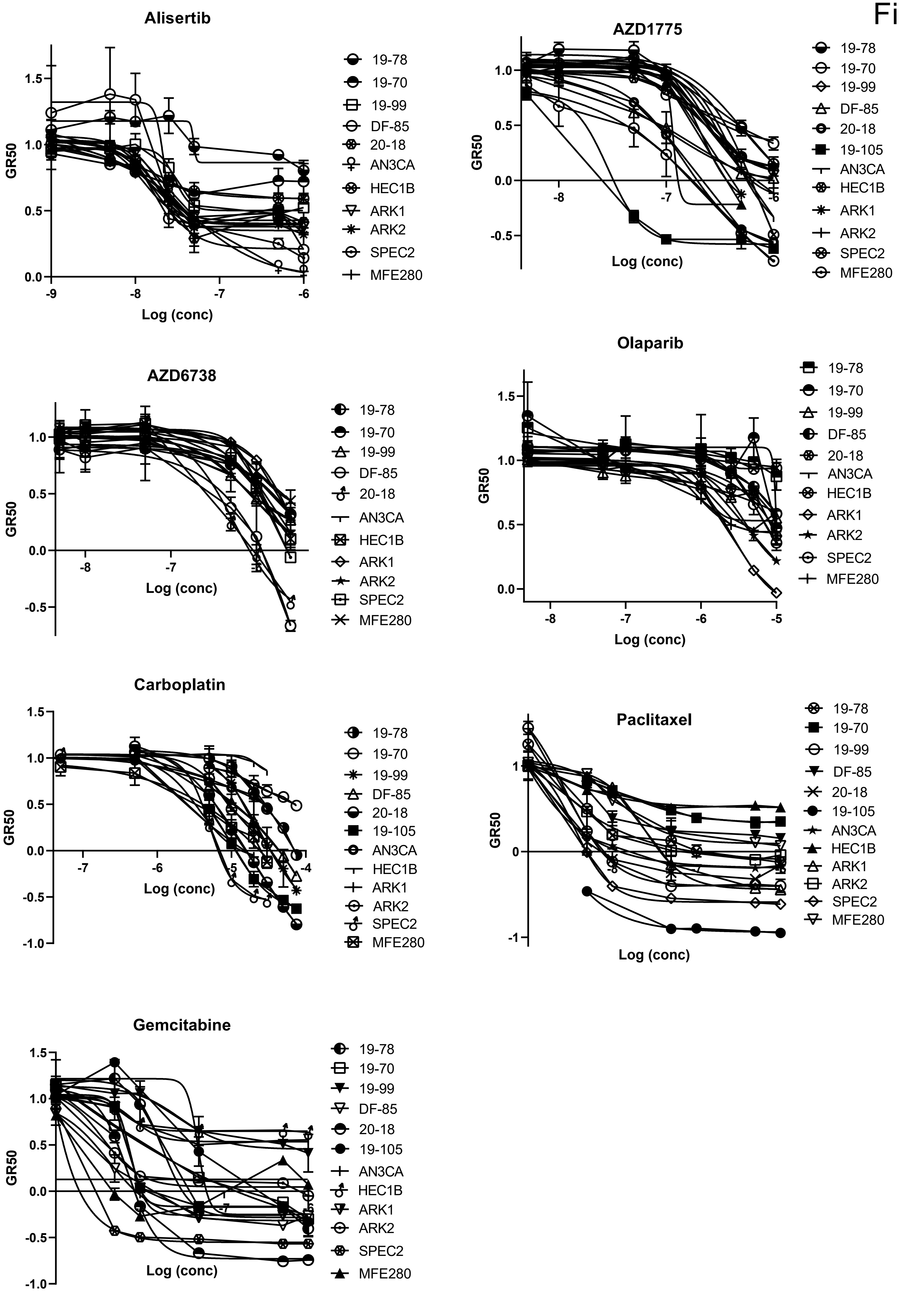
Alisertib
GR50
Log (conc)
19-78
19-70
19-99
DF-85
20-18
AN3CA
HEC1B
ARK1
ARK2
SPEC2
MFE280
AZD1775
GR50
Log (conc)
19-78
19-70
19-99
DF-85
20-18
19-105
AN3CA
HEC1B
ARK1
ARK2
SPEC2
MFE280
AZD6738
GR50
Log (conc)
19-78
19-70
19-99
DF-85
20-18
AN3CA
HEC1B
ARK1
ARK2
SPEC2
MFE280
Olaparib
GR50
Log (conc)
19-78
19-70
19-99
DF-85
20-18
AN3CA
HEC1B
ARK1
ARK2
SPEC2
MFE280
Carboplatin
GR50
Log (conc)
19-78
19-70
19-99
DF-85
20-18
19-105
AN3CA
HEC1B
ARK1
ARK2
SPEC2
MFE280
Paclitaxel
GR50
Log (conc)
19-78
19-70
19-99
DF-85
20-18
19-105
AN3CA
HEC1B
ARK1
ARK2
SPEC2
MFE280
Gemcitabine
GR50
Log (conc)
19-78
19-70
19-99
DF-85
20-18
19-105
AN3CA
HEC1B
ARK1
ARK2
SPEC2
MFE280

Figure S4

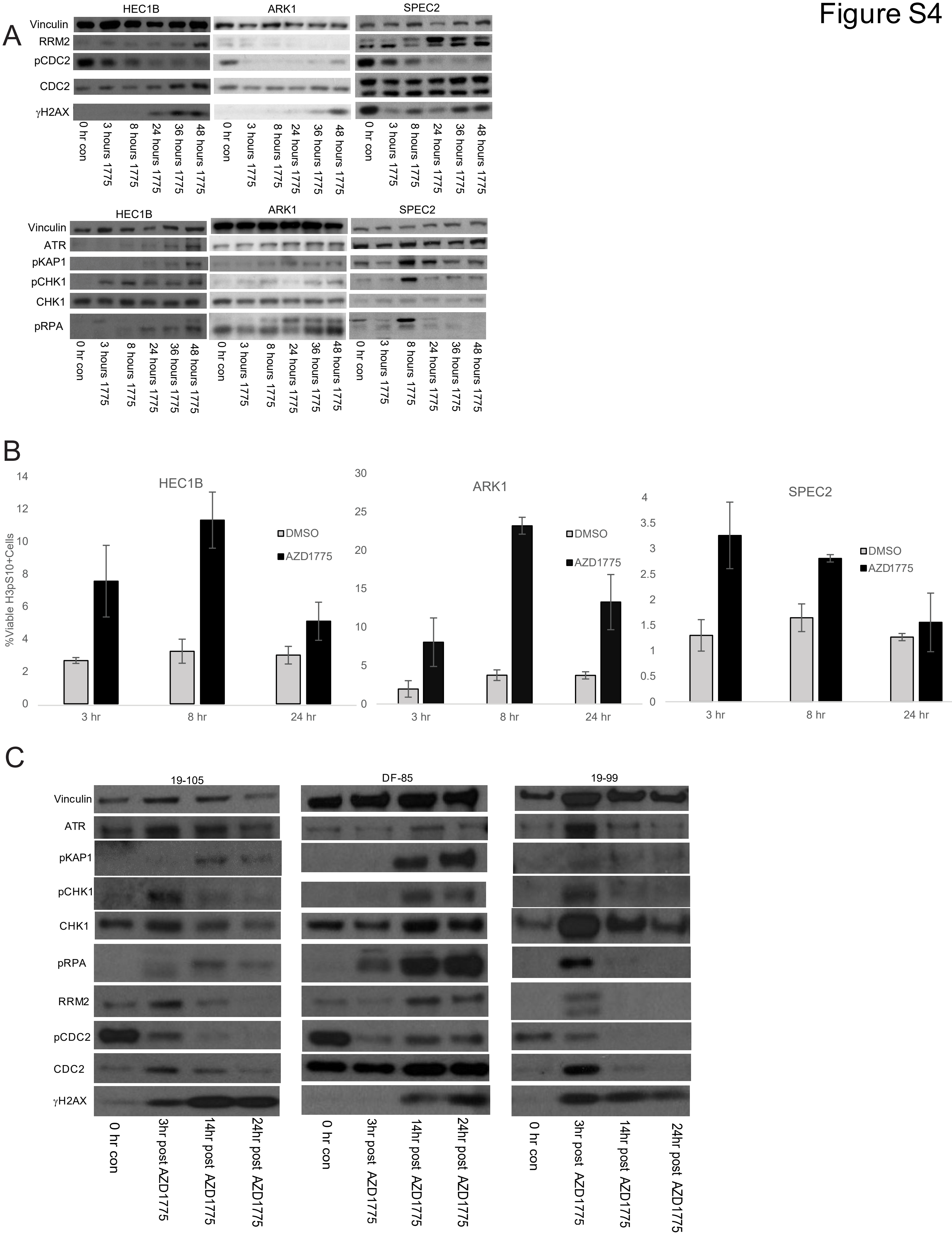
A
HEC1B
ARK1
SPEC2
Vinculin
RRM2
pCDC2
CDC2
γH2AX
0 hr con
3 hours 1775
8 hours 1775
24 hours 1775
36 hours 1775
48 hours 1775
HEC1B
ARK1
SPEC2
Vinculin
ATR
pKAP1
pCHK1
CHK1
pRPA
0 hr con
3 hours 1775
8 hours 1775
24 hours 1775
36 hours 1775
48 hours 1775
B
HEC1B
%Viable H3pS10+Cells
0
2
4
6
8
10
12
14
DMSO
AZD1775
3 hr
8 hr
24 hr
ARK1
0
5
10
15
20
25
30
DMSO
AZD1775
3 hr
8 hr
24 hr
SPEC2
0
0.5
1
1.5
2
2.5
3
3.5
4
DMSO
AZD1775
3 hr
8 hr
24 hr
C
19-105
DF-85
19-99
Vinculin
ATR
pKAP1
pCHK1
CHK1
pRPA
RRM2
pCDC2
CDC2
γH2AX
0 hr con
3hr post AZD1775
14hr post AZD1775
24hr post AZD1775

Figure S5

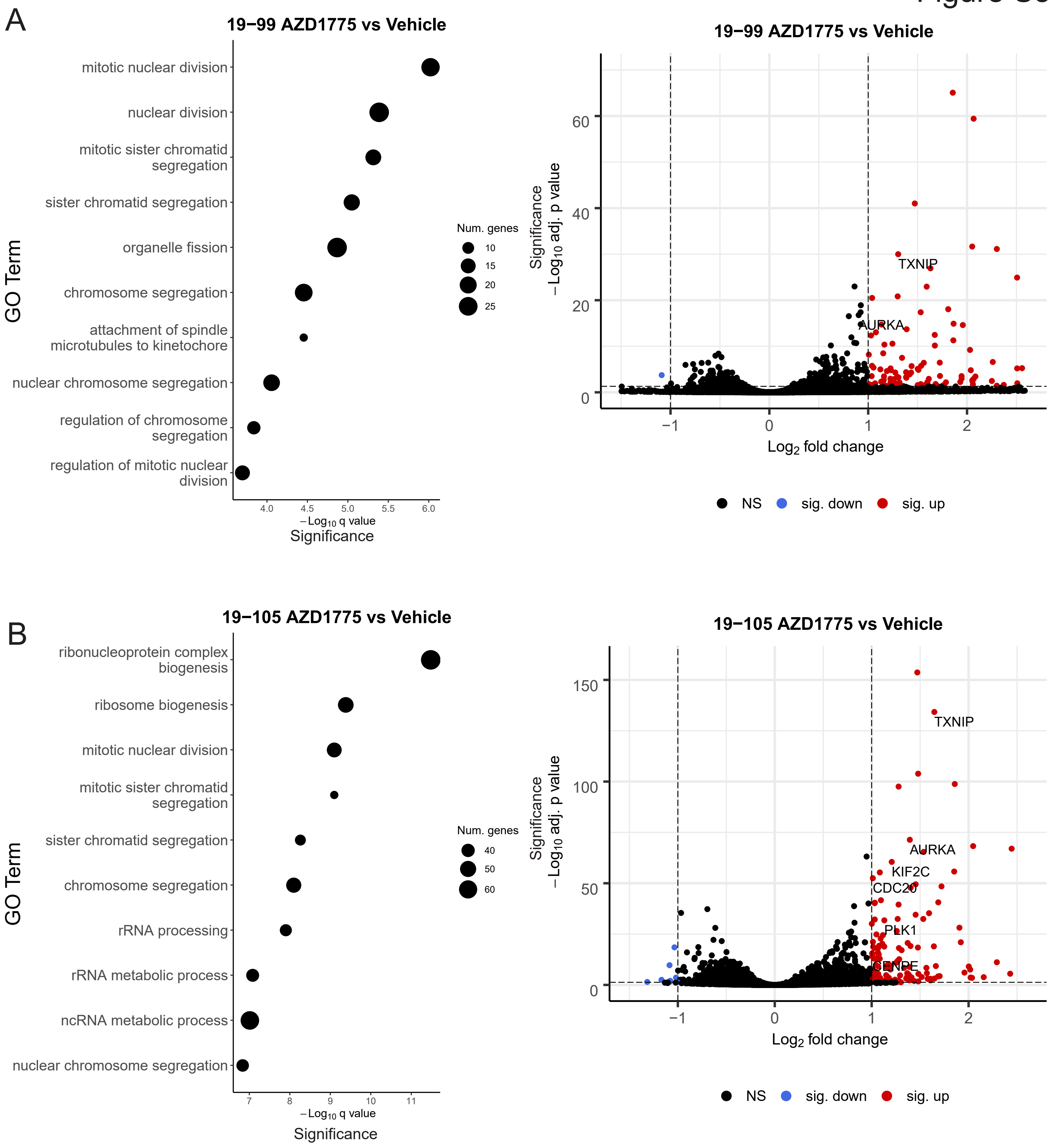
A
19−99 AZD1775 vs Vehicle
mitotic nuclear division
nuclear division
mitotic sister chromatid segregation
sister chromatid segregation
organelle fission
chromosome segregation
attachment of spindle microtubules to kinetochore
nuclear chromosome segregation
regulation of chromosome segregation
regulation of mitotic nuclear division
GO Term
4.0
4.5
5.0
5.5
6.0
−Log10 q value
Significance
Num. genes
10
15
20
25
19−99 AZD1775 vs Vehicle
Significance
−Log10 adj. p value
60
40
20
0
TXNIP
AURKA
−1
0
1
2
Log2 fold change
NS
sig. down
sig. up
B
19−105 AZD1775 vs Vehicle
ribonucleoprotein complex biogenesis
ribosome biogenesis
mitotic nuclear division
mitotic sister chromatid segregation
sister chromatid segregation
chromosome segregation
rRNA processing
rRNA metabolic process
ncRNA metabolic process
nuclear chromosome segregation
GO Term
7
8
9
10
11
−Log10 q value
Significance
Num. genes
40
50
60
19−105 AZD1775 vs Vehicle
Significance
−Log10 adj. p value
150
100
50
0
TXNIP
AURKA
KIF2C
CDC20
PLK1
CENPE
−1
0
1
2
Log2 fold change
NS
sig. down
sig. up

Figure S6

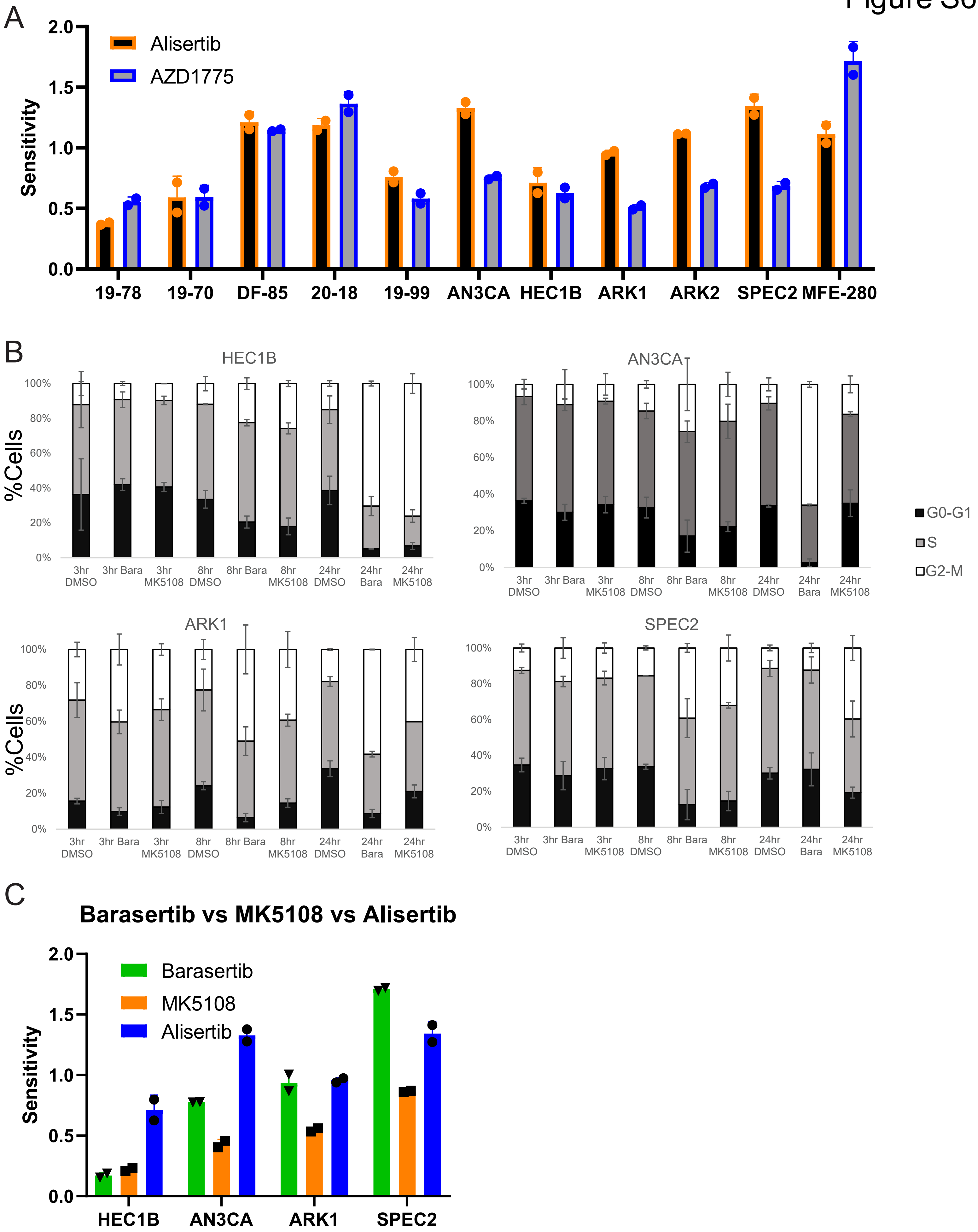
A
Alisertib
AZD1775
Sensitivity
2.0
1.5
1.0
0.5
0.0
19-78
19-70
DF-85
20-18
19-99
AN3CA
HEC1B
ARK1
ARK2
SPEC2
MFE-280
B
HEC1B
AN3CA
ARK1
SPEC2
%Cells
100%
80%
60%
40%
20%
0%
3hr DMSO
3hr Bara
3hr MK5108
8hr DMSO
8hr Bara
8hr MK5108
24hr DMSO
24hr Bara
24hr MK5108
G0-G1
S
G2-M
C
Barasertib vs MK5108 vs Alisertib
Barasertib
MK5108
Alisertib
Sensitivity
2.0
1.5
1.0
0.5
0.0
HEC1B
AN3CA
ARK1
SPEC2

Figure S7

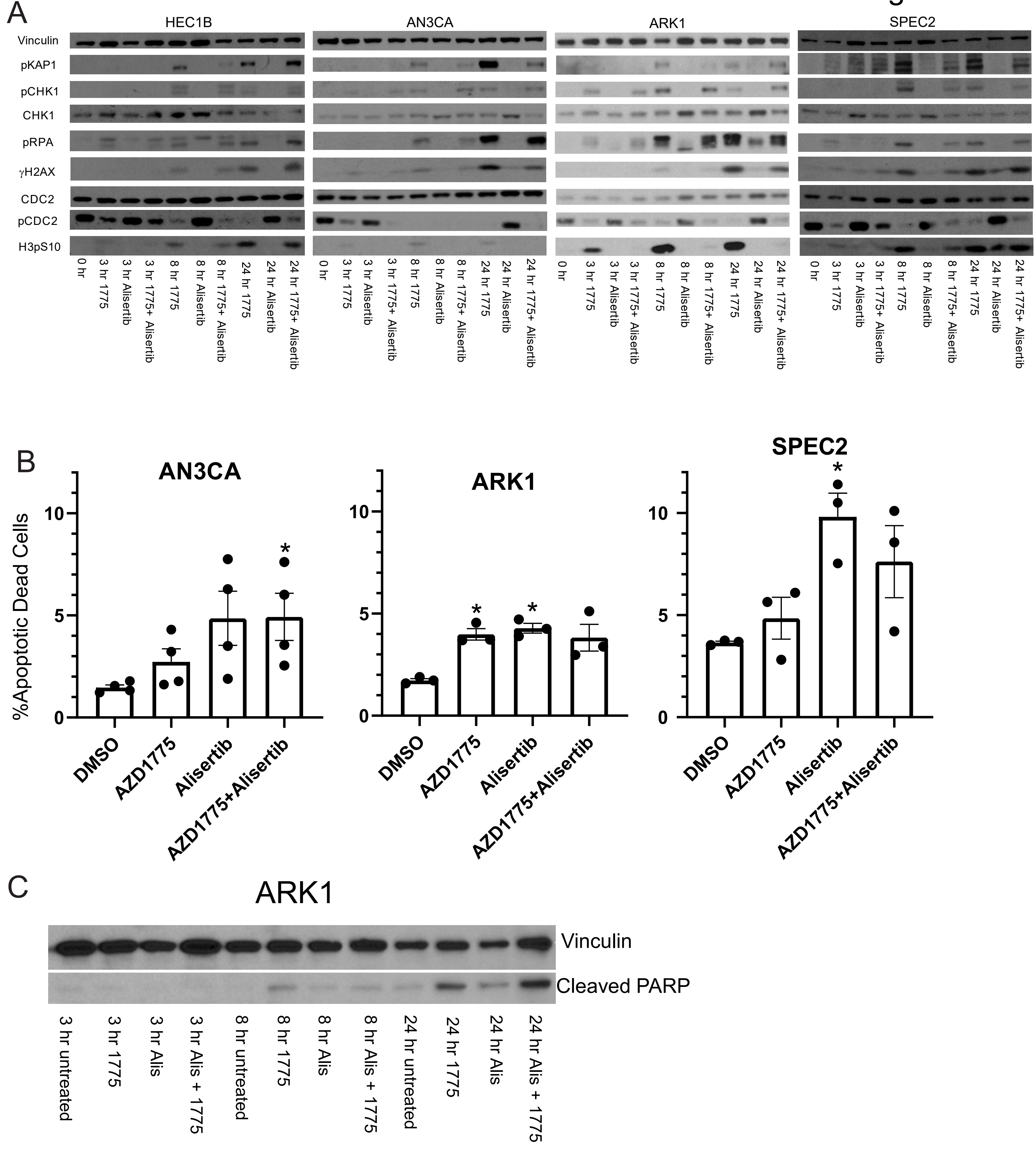
A
HEC1B
AN3CA
ARK1
SPEC2
Vinculin
pKAP1
pCHK1
CHK1
pRPA
γH2AX
CDC2
pCDC2
H3pS10
0 hr
3 hr 1775
3 hr Alisertib
3 hr 1775+ Alisertib
8 hr 1775
8 hr Alisertib
8 hr 1775+ Alisertib
24 hr 1775
24 hr Alisertib
24 hr 1775+ Alisertib
B
AN3CA
ARK1
SPEC2
%Apoptotic Dead Cells
0
5
10
*
DMSO
AZD1775
Alisertib
AZD1775+Alisertib
C
ARK1
Vinculin
Cleaved PARP
3 hr untreated
3 hr 1775
3 hr Alis
3 hr Alis + 1775
8 hr untreated
8 hr 1775
8 hr Alis
8 hr Alis + 1775
24 hr untreated
24 hr 1775
24 hr Alis
24 hr Alis + 1775
