# Supplementary material for: Enhanced Efficacy of Aurora Kinase Inhibitors in G2/M Checkpoint Deficient TP53 Mutant Uterine Carcinomas Is Linked to the Summation of LKB1–AKT–p53 Interactions
Source: Cancers (Basel). 2021 May 3;13(9):2195. doi: 10.3390/cancers13092195 (PMC8125555; doi:10.3390/cancers13092195)
Supplement: Supplementary file 1 [file cancers-13-02195-s001.zip › Lynch and Hill Supplementary Matierals/original blot/Figure 2G.pptx]

## Slide 1
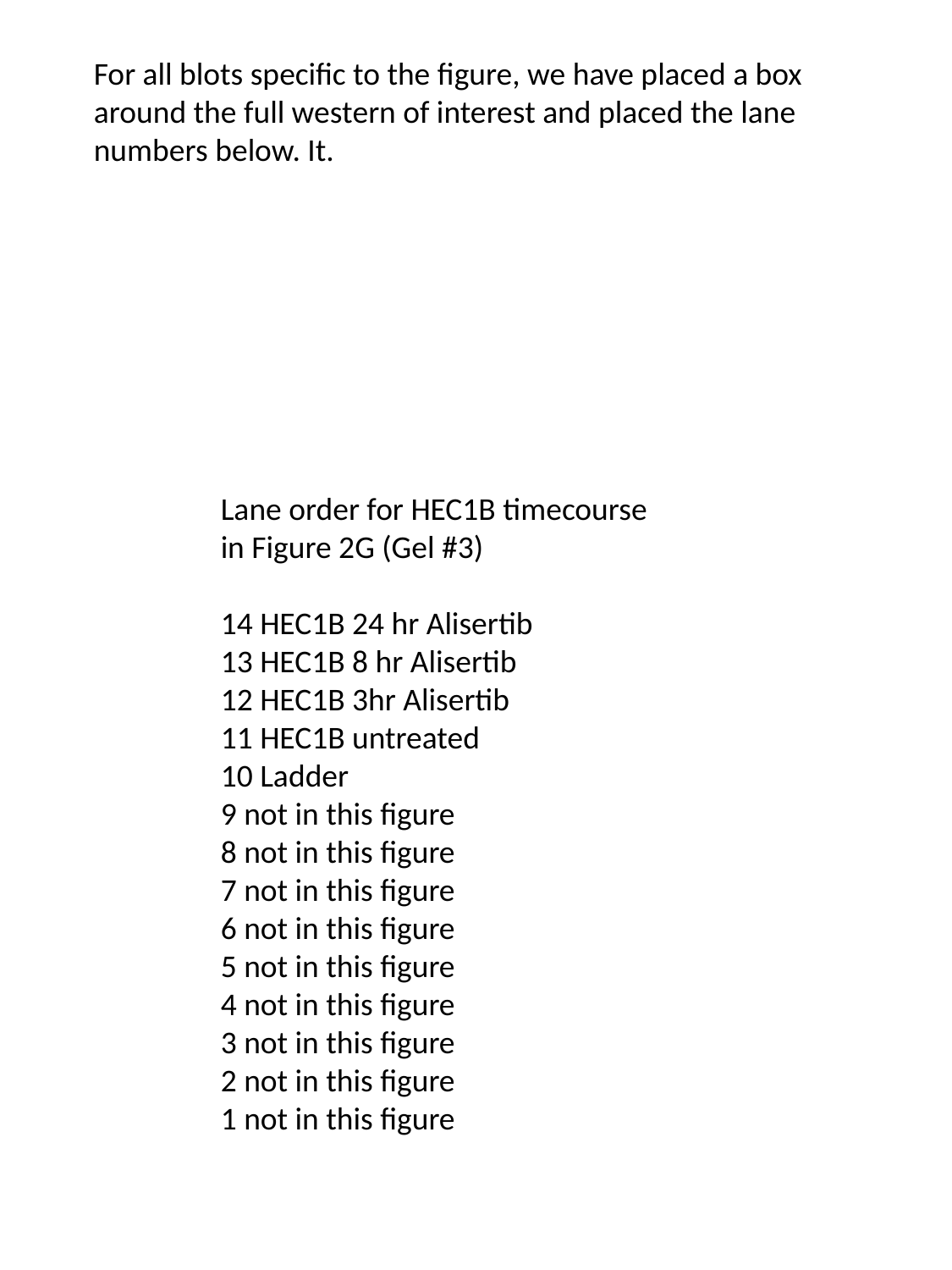

For all blots specific to the figure, we have placed a box around the full western of interest and placed the lane numbers below. It.
Lane order for HEC1B timecourse in Figure 2G (Gel #3)
14 HEC1B 24 hr Alisertib
13 HEC1B 8 hr Alisertib
12 HEC1B 3hr Alisertib
11 HEC1B untreated
10 Ladder
9 not in this figure
8 not in this figure
7 not in this figure
6 not in this figure
5 not in this figure
4 not in this figure
3 not in this figure
2 not in this figure
1 not in this figure

## Slide 2
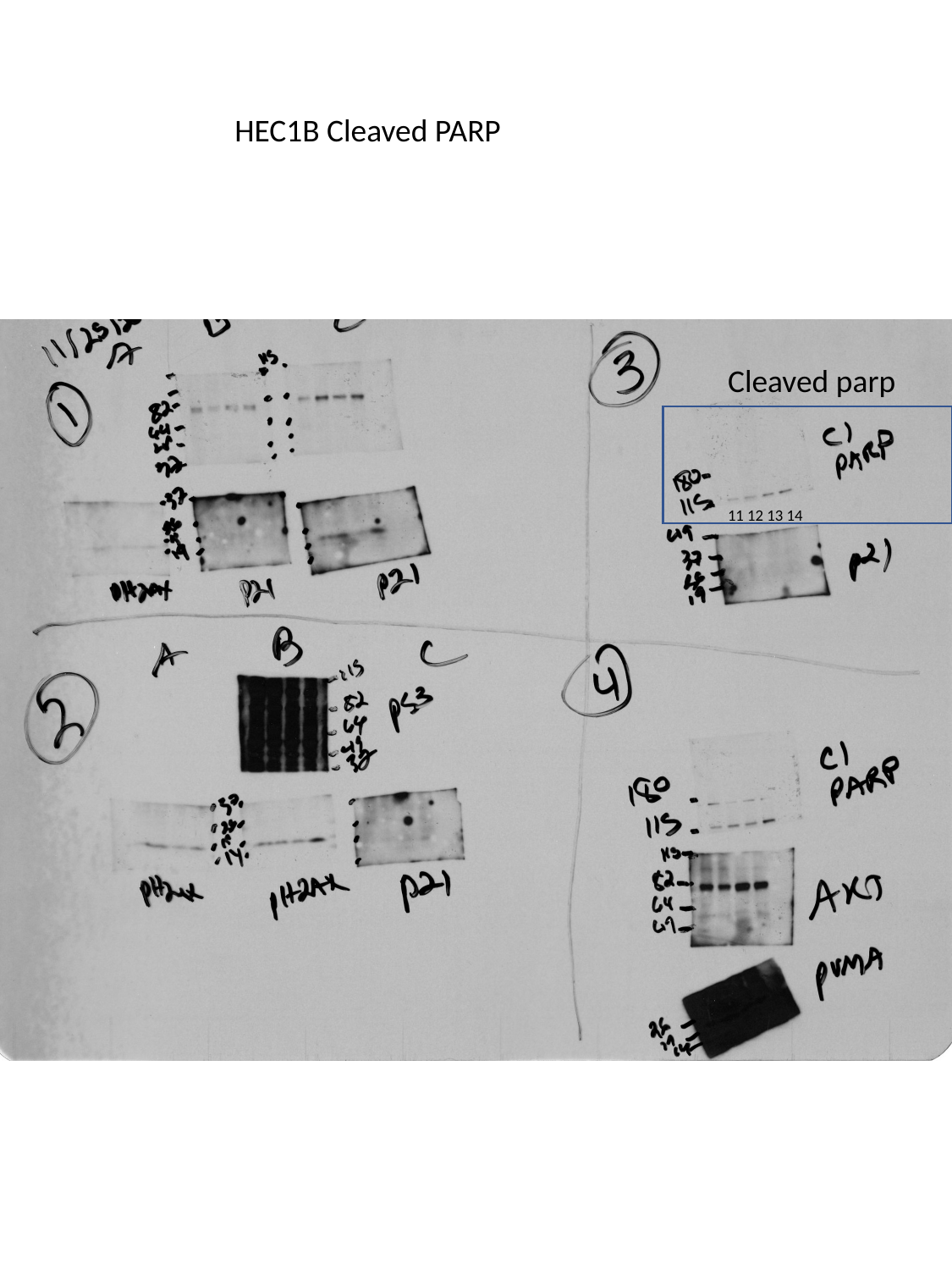

HEC1B Cleaved PARP
Cleaved parp
11 12 13 14

## Slide 3
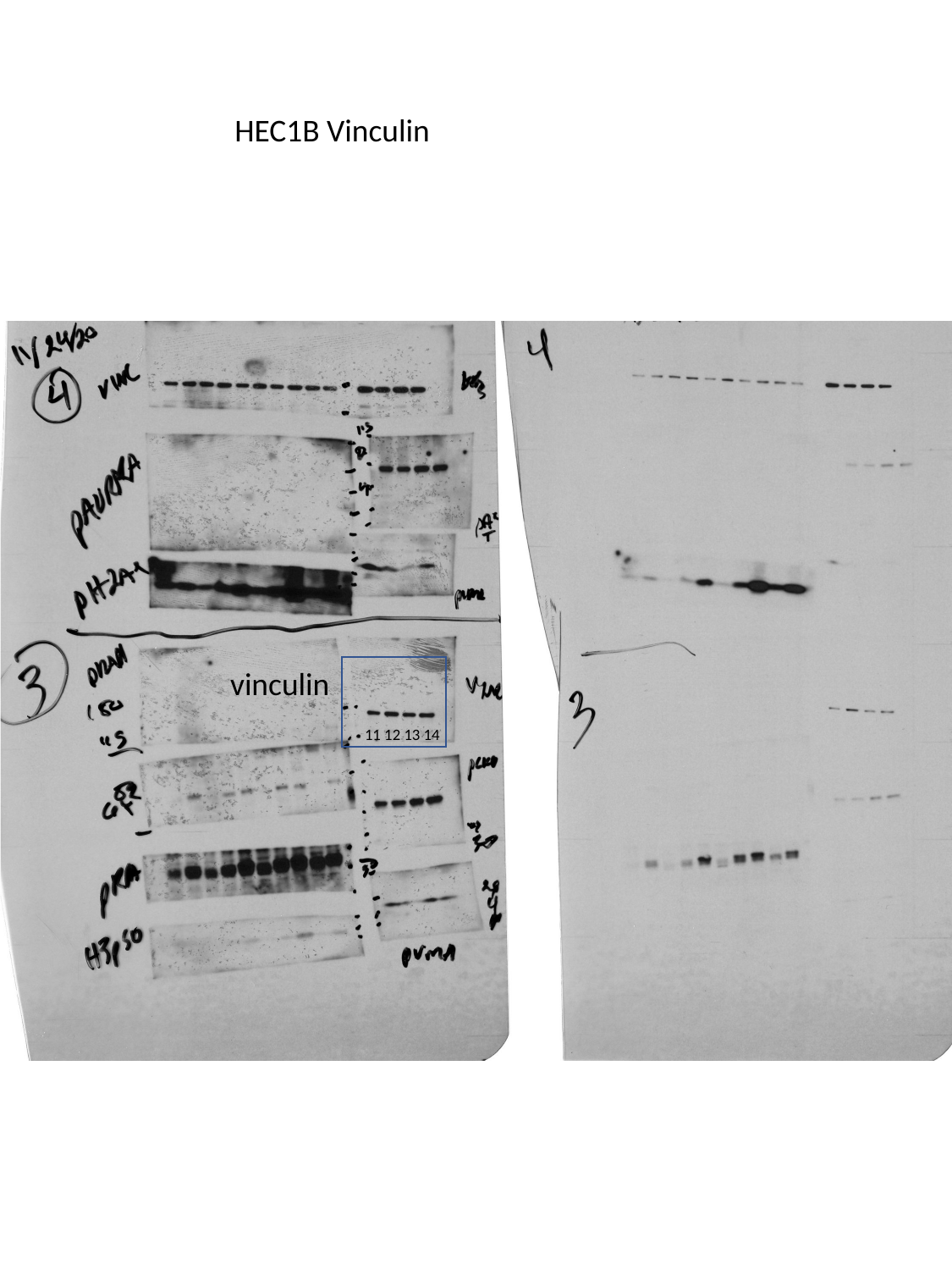

HEC1B Vinculin
vinculin
11 12 13 14

## Slide 4
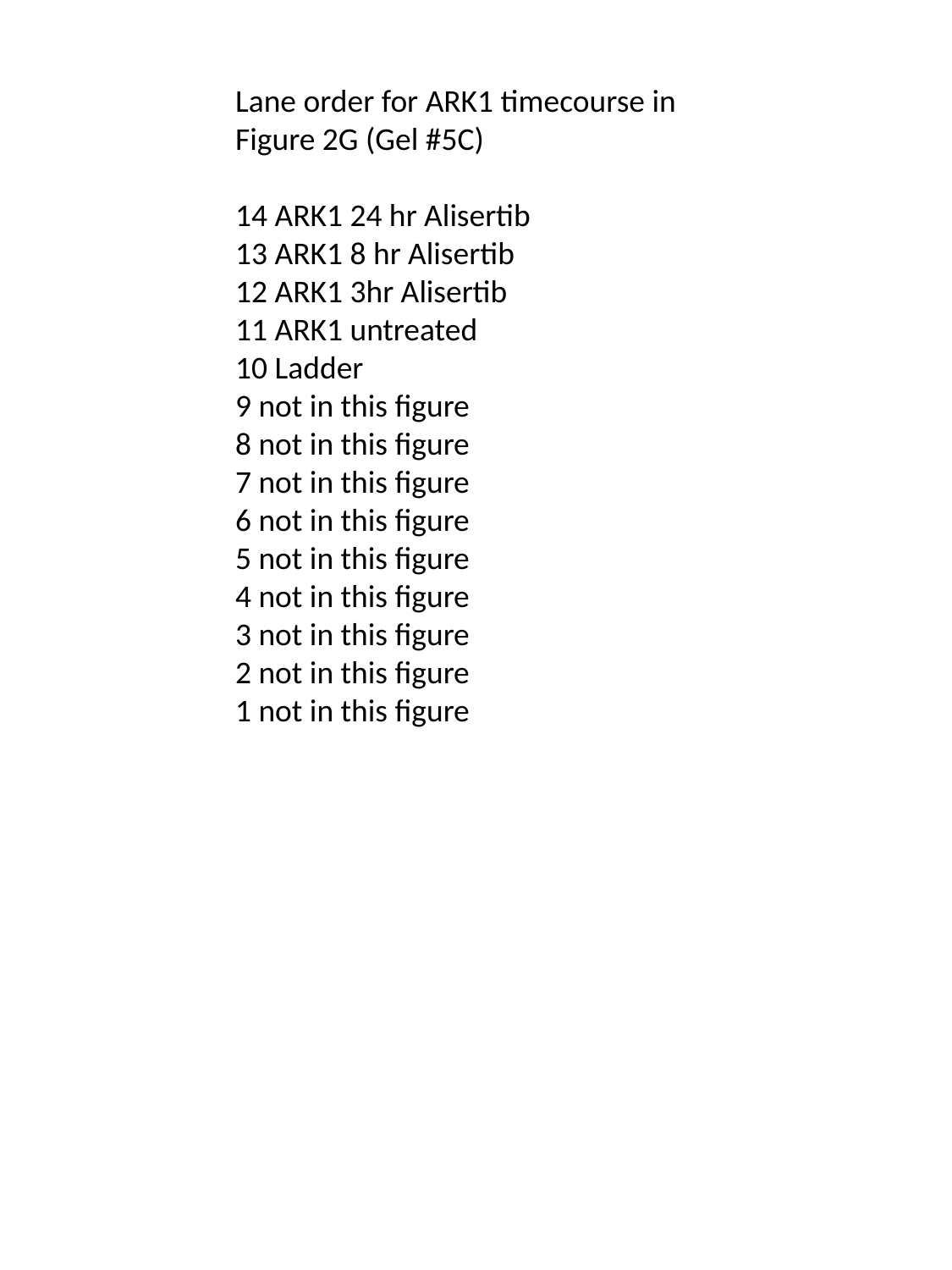

Lane order for ARK1 timecourse in Figure 2G (Gel #5C)
14 ARK1 24 hr Alisertib
13 ARK1 8 hr Alisertib
12 ARK1 3hr Alisertib
11 ARK1 untreated
10 Ladder
9 not in this figure
8 not in this figure
7 not in this figure
6 not in this figure
5 not in this figure
4 not in this figure
3 not in this figure
2 not in this figure
1 not in this figure

## Slide 5
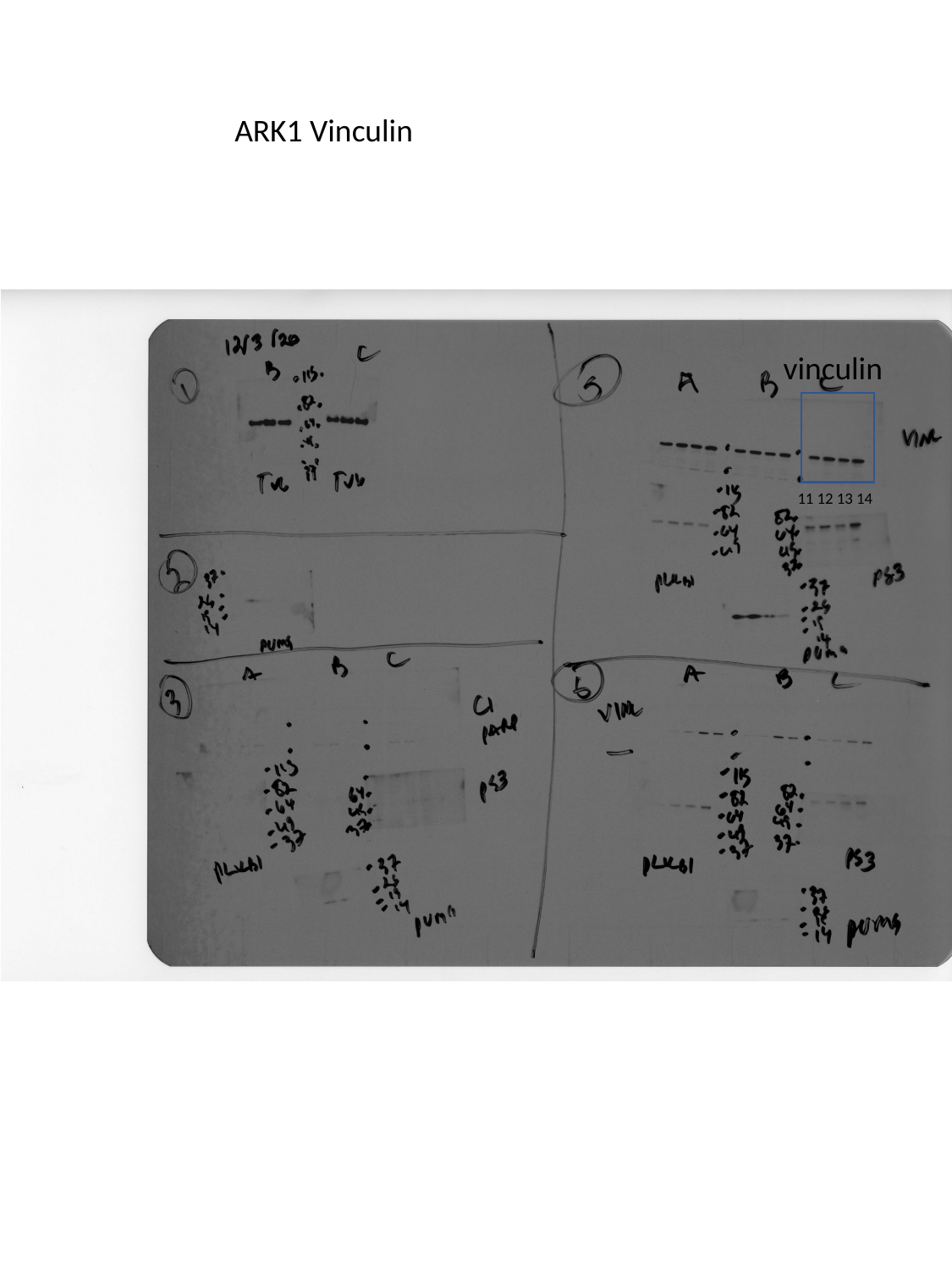

ARK1 Vinculin
vinculin
11 12 13 14

## Slide 6
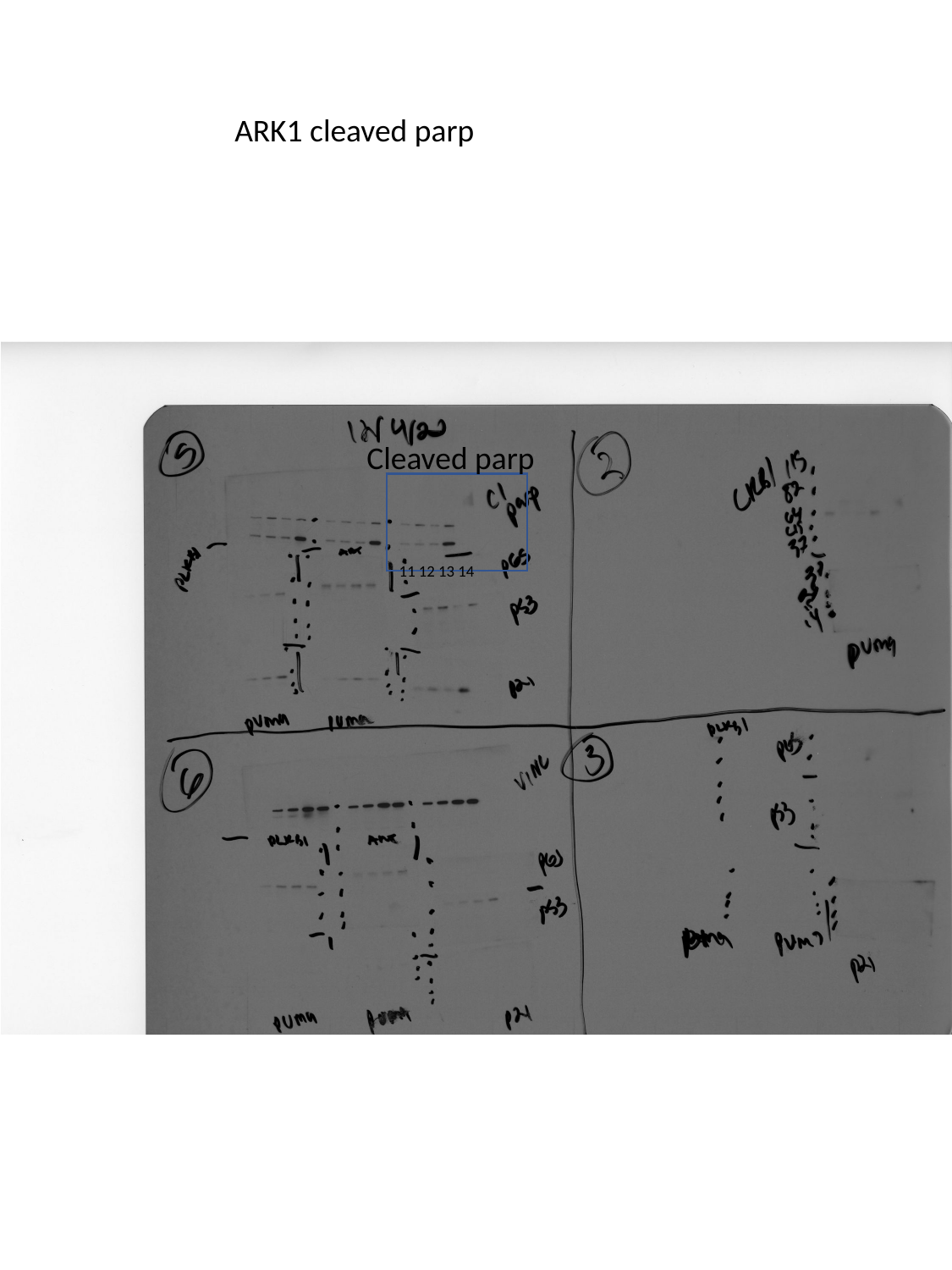

ARK1 cleaved parp
Cleaved parp
11 12 13 14

## Slide 7
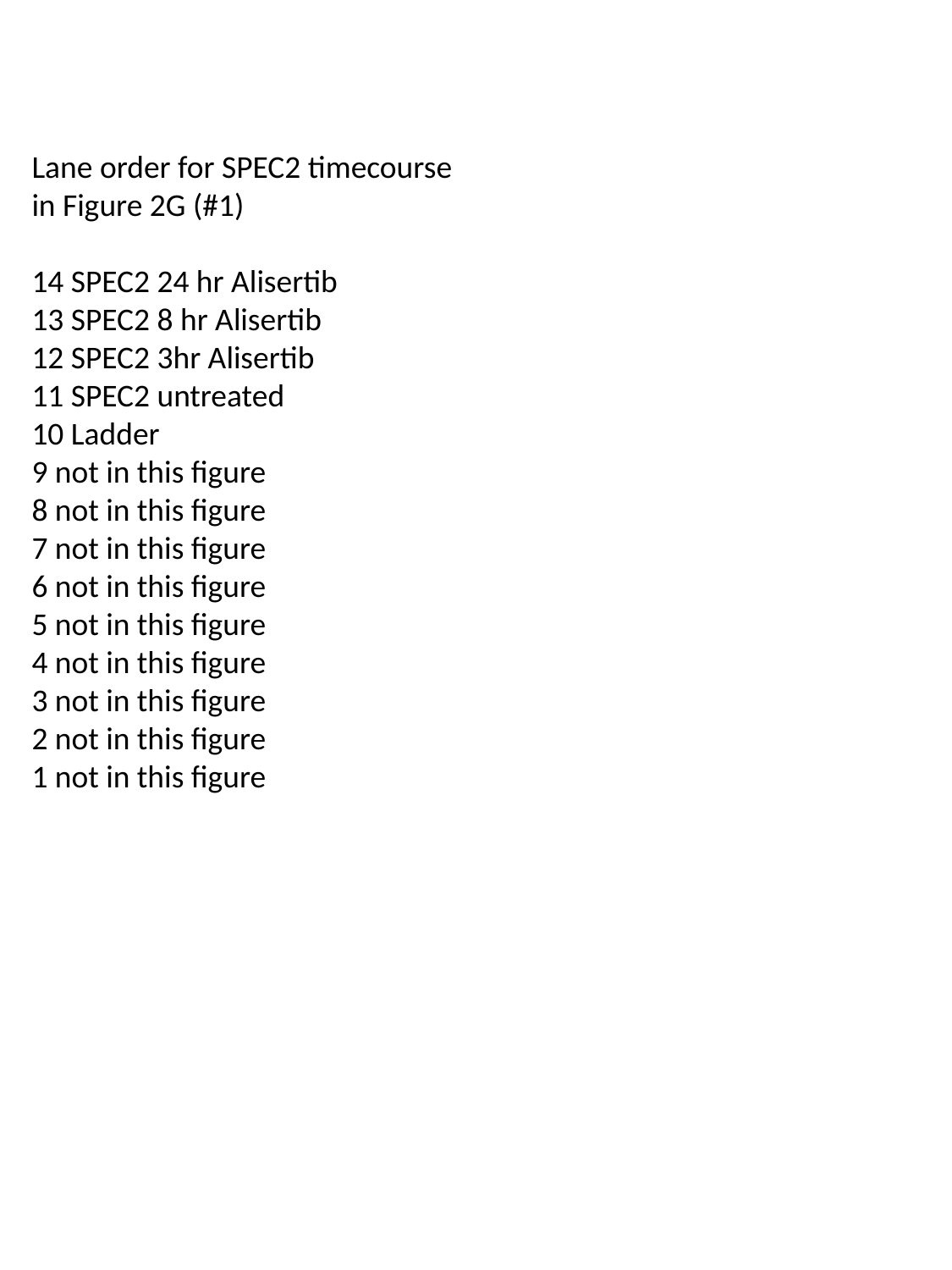

Lane order for SPEC2 timecourse in Figure 2G (#1)
14 SPEC2 24 hr Alisertib
13 SPEC2 8 hr Alisertib
12 SPEC2 3hr Alisertib
11 SPEC2 untreated
10 Ladder
9 not in this figure
8 not in this figure
7 not in this figure
6 not in this figure
5 not in this figure
4 not in this figure
3 not in this figure
2 not in this figure
1 not in this figure

## Slide 8
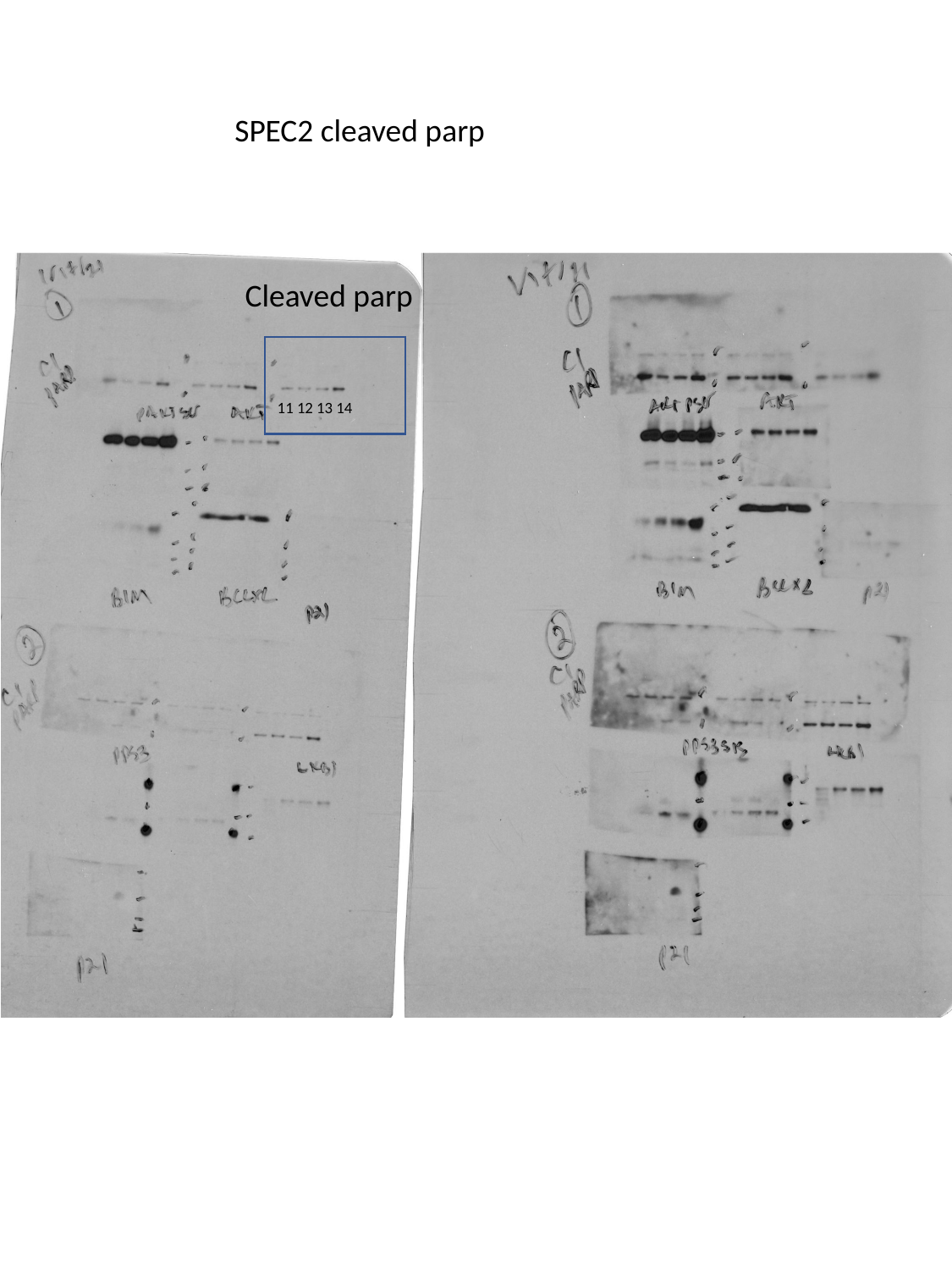

SPEC2 cleaved parp
Cleaved parp
11 12 13 14

## Slide 9
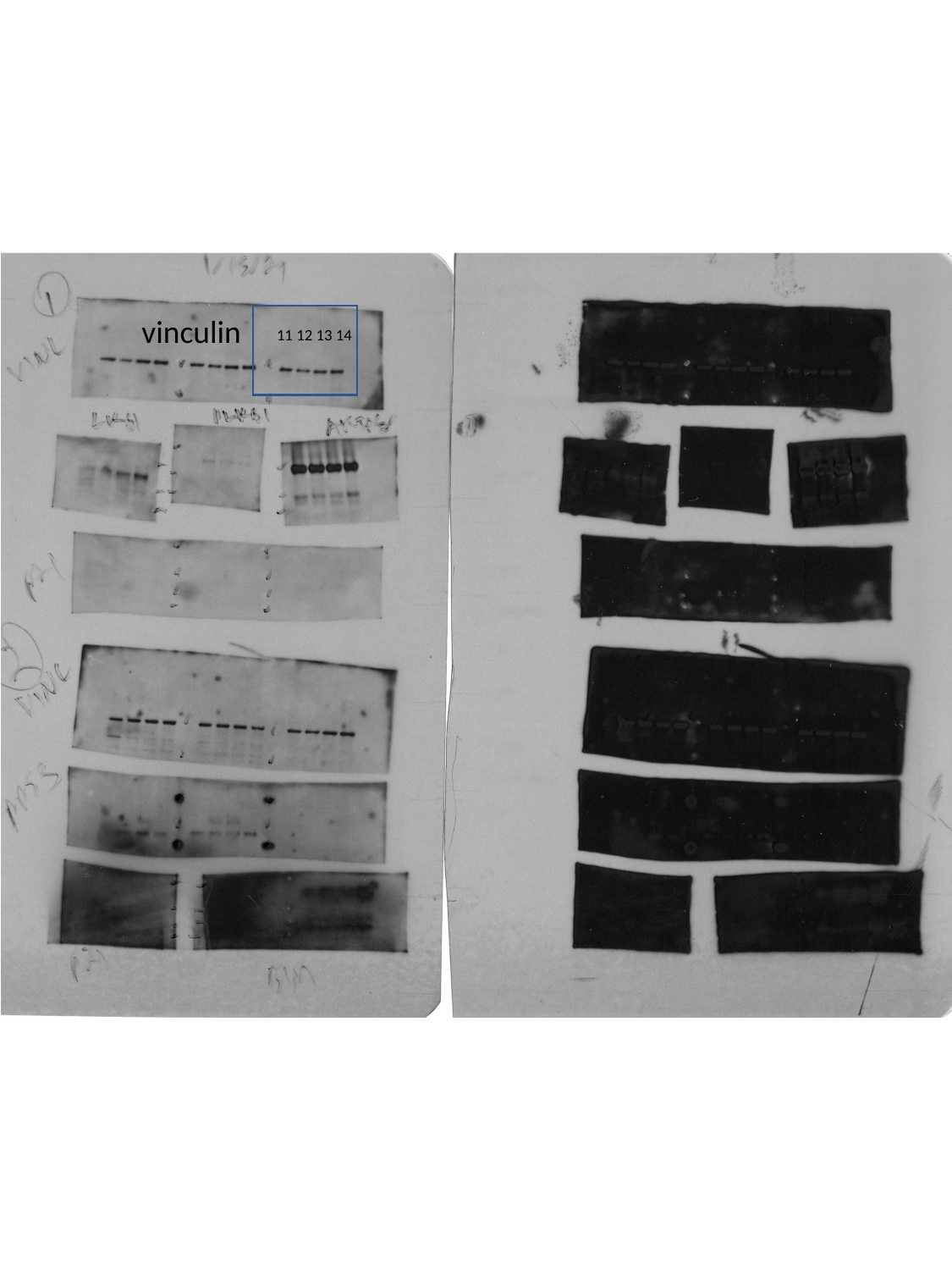

vinculin
11 12 13 14

## Slide 10
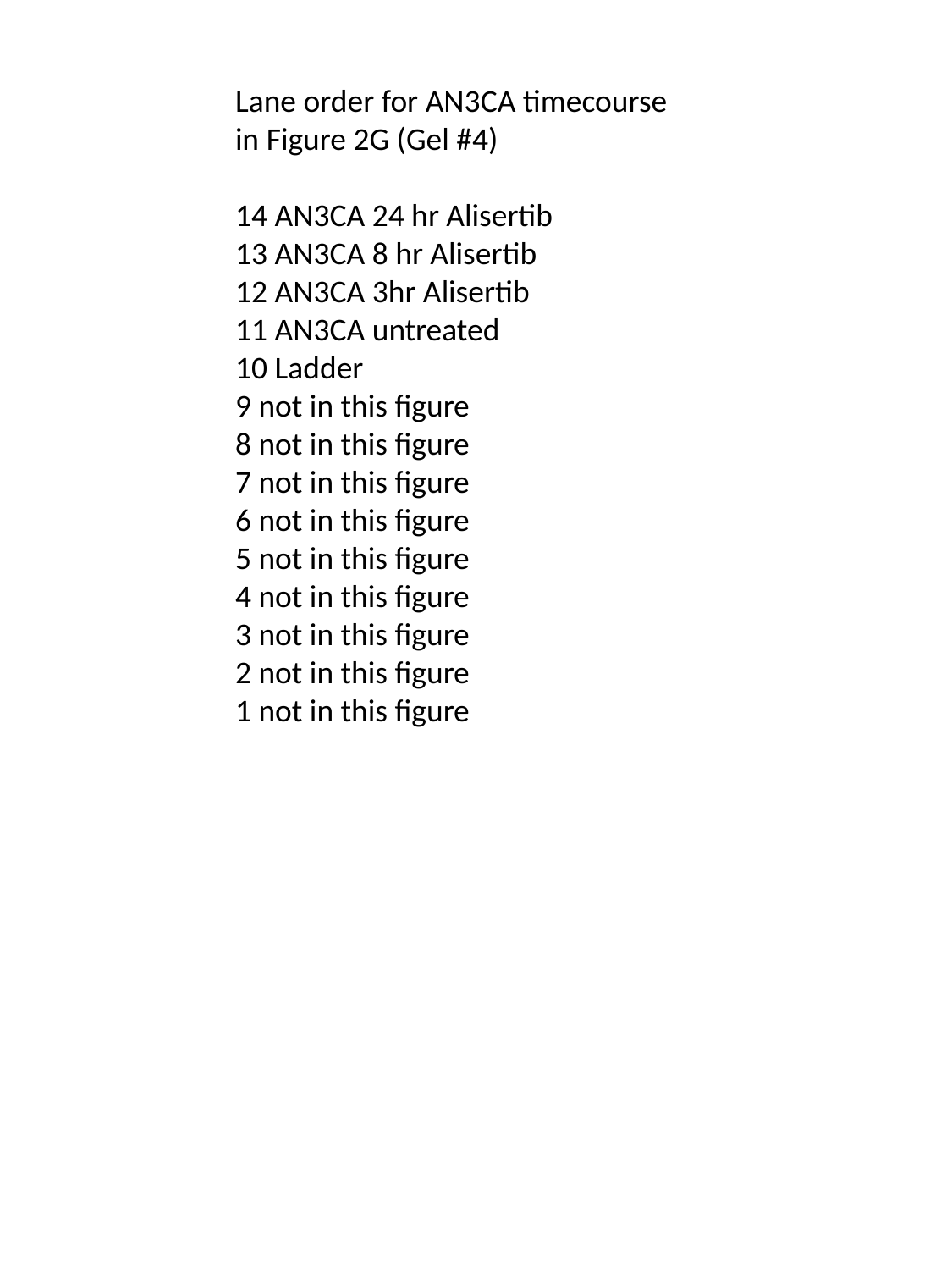

Lane order for AN3CA timecourse in Figure 2G (Gel #4)
14 AN3CA 24 hr Alisertib
13 AN3CA 8 hr Alisertib
12 AN3CA 3hr Alisertib
11 AN3CA untreated
10 Ladder
9 not in this figure
8 not in this figure
7 not in this figure
6 not in this figure
5 not in this figure
4 not in this figure
3 not in this figure
2 not in this figure
1 not in this figure

## Slide 11
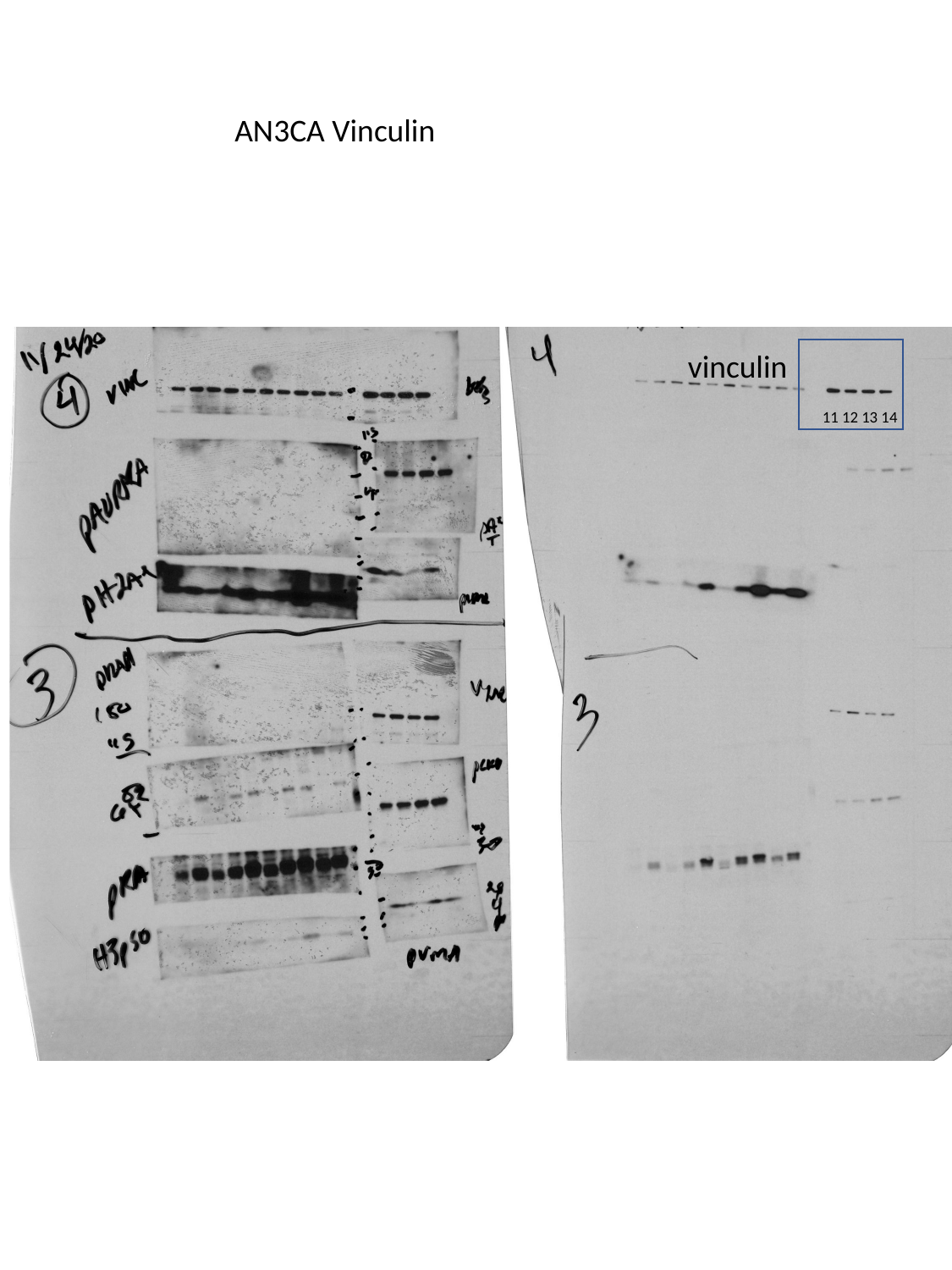

AN3CA Vinculin
vinculin
11 12 13 14

## Slide 12
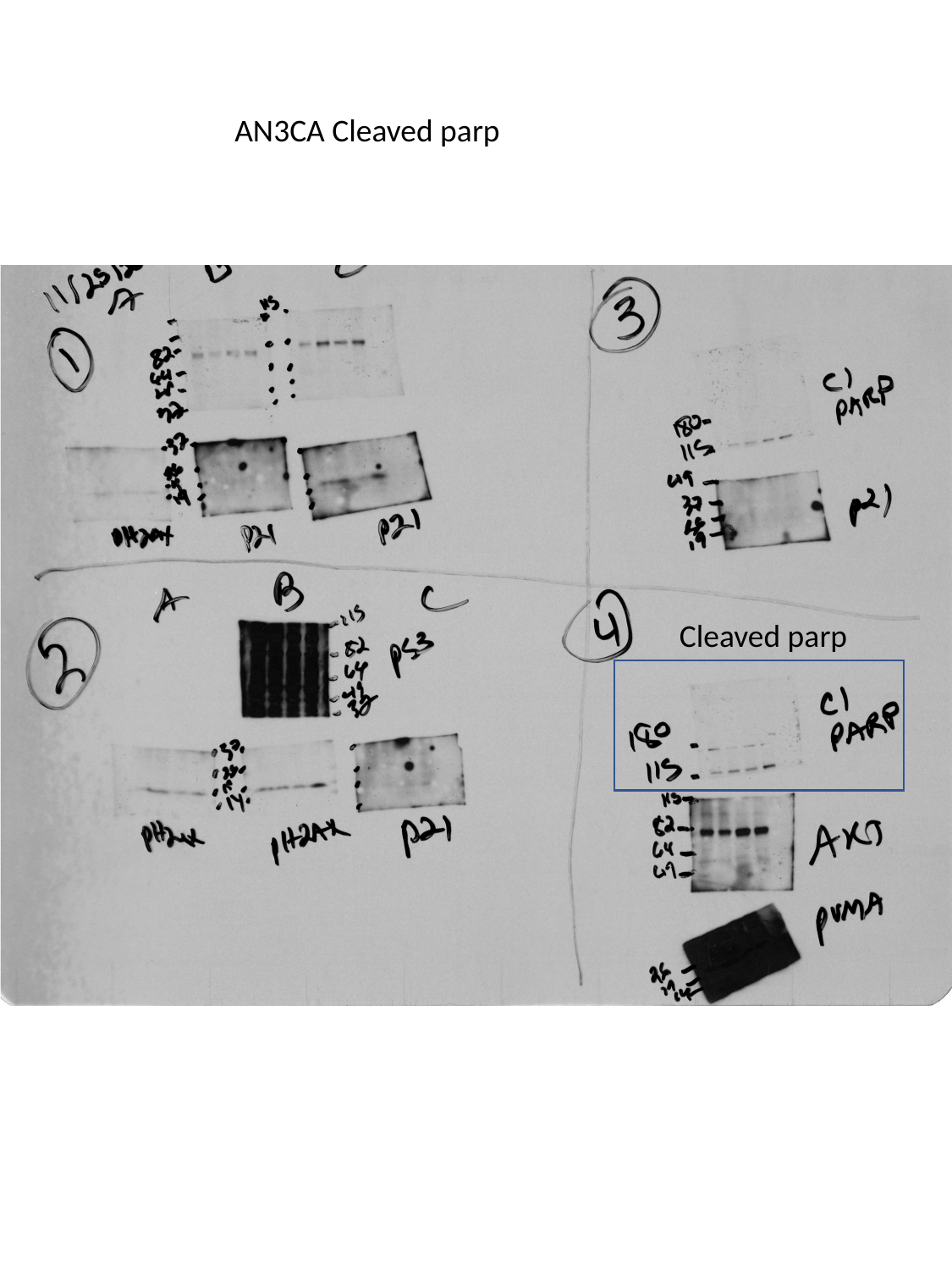

AN3CA Cleaved parp
Cleaved parp
