# Supplementary material for: Enhanced Efficacy of Aurora Kinase Inhibitors in G2/M Checkpoint Deficient TP53 Mutant Uterine Carcinomas Is Linked to the Summation of LKB1–AKT–p53 Interactions
Source: Cancers (Basel). 2021 May 3;13(9):2195. doi: 10.3390/cancers13092195 (PMC8125555; doi:10.3390/cancers13092195)
Supplement: Supplementary file 1 [file cancers-13-02195-s001.zip › Lynch and Hill Supplementary Matierals/original blot/Figure 3B.pptx]

## Slide 1
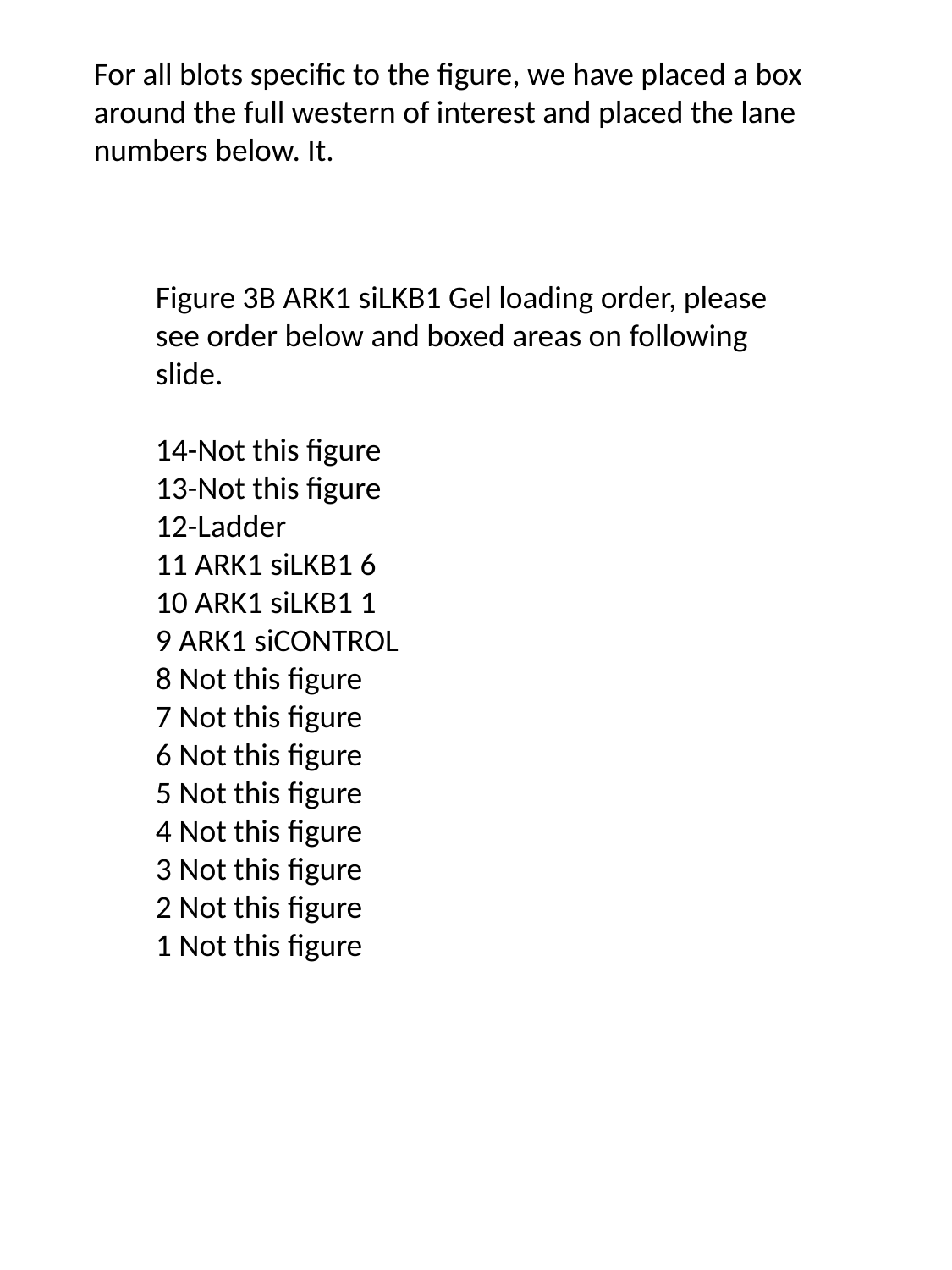

For all blots specific to the figure, we have placed a box around the full western of interest and placed the lane numbers below. It.
Figure 3B ARK1 siLKB1 Gel loading order, please see order below and boxed areas on following slide.
14-Not this figure
13-Not this figure
12-Ladder
11 ARK1 siLKB1 6
10 ARK1 siLKB1 1
9 ARK1 siCONTROL
8 Not this figure
7 Not this figure
6 Not this figure
5 Not this figure
4 Not this figure
3 Not this figure
2 Not this figure
1 Not this figure

## Slide 2
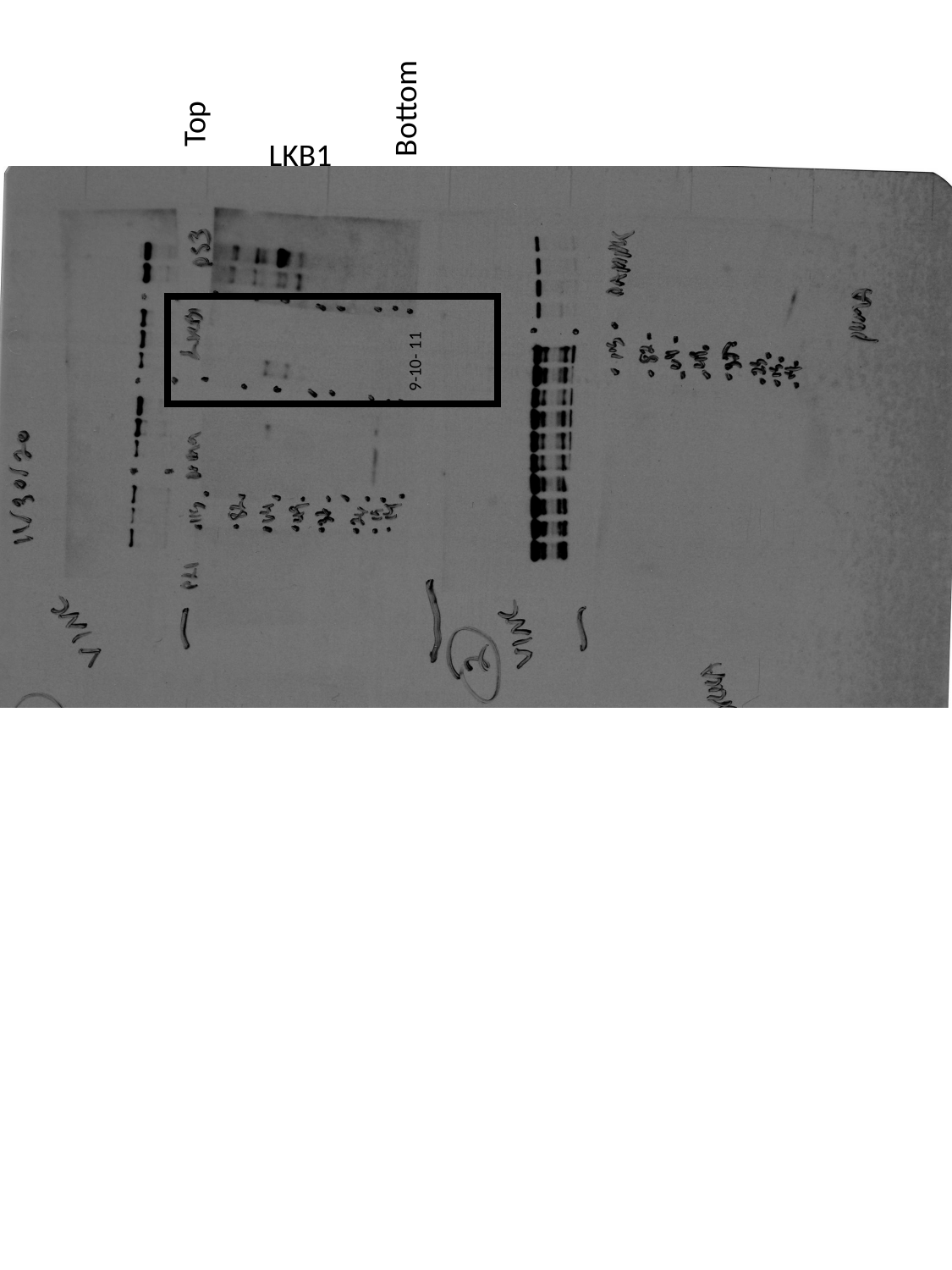

Bottom
Top
LKB1
9-10- 11

## Slide 3
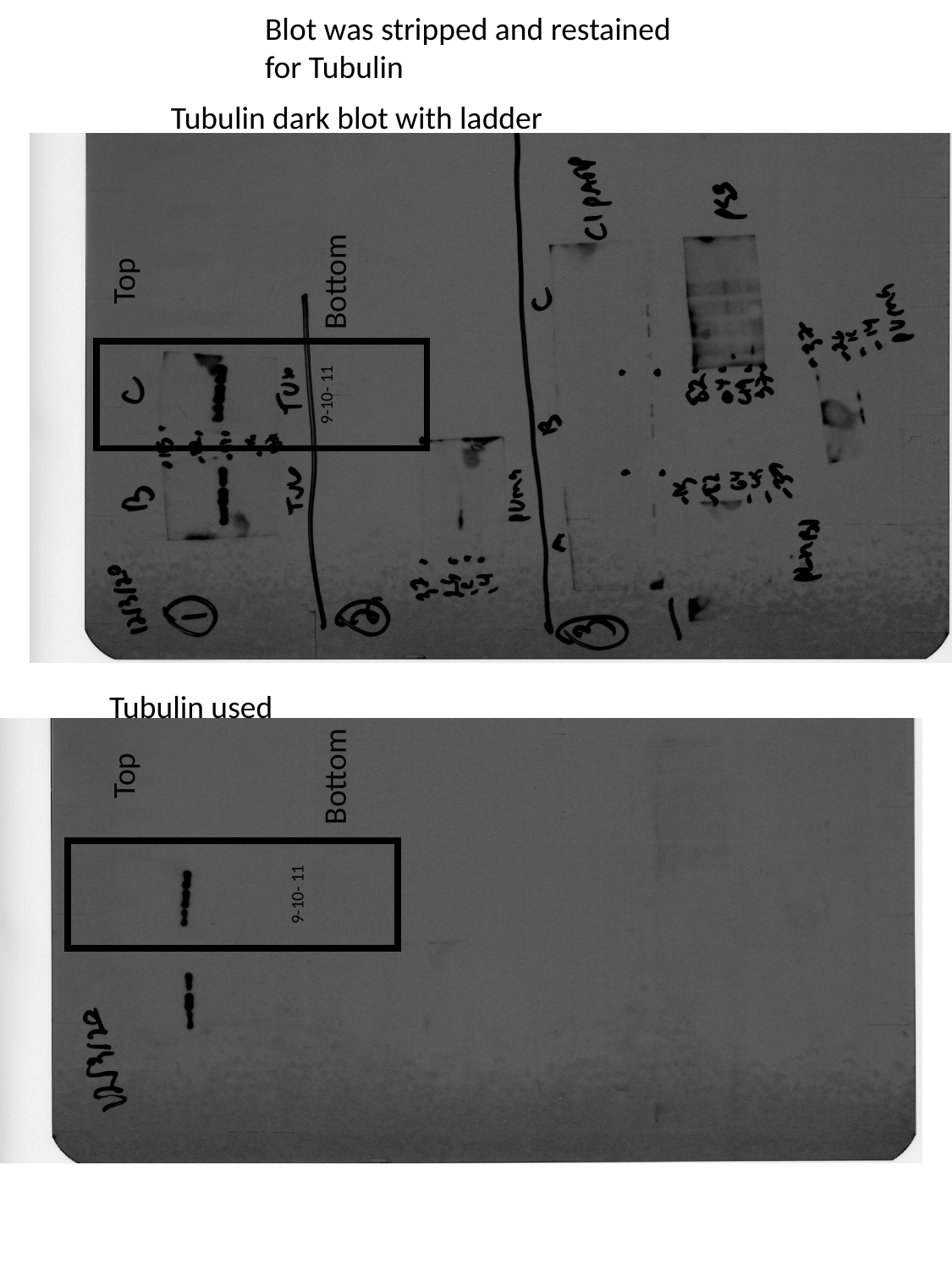

Blot was stripped and restained for Tubulin
Tubulin dark blot with ladder
Bottom
Top
9-10- 11
Tubulin used
Bottom
Top
9-10- 11

## Slide 4
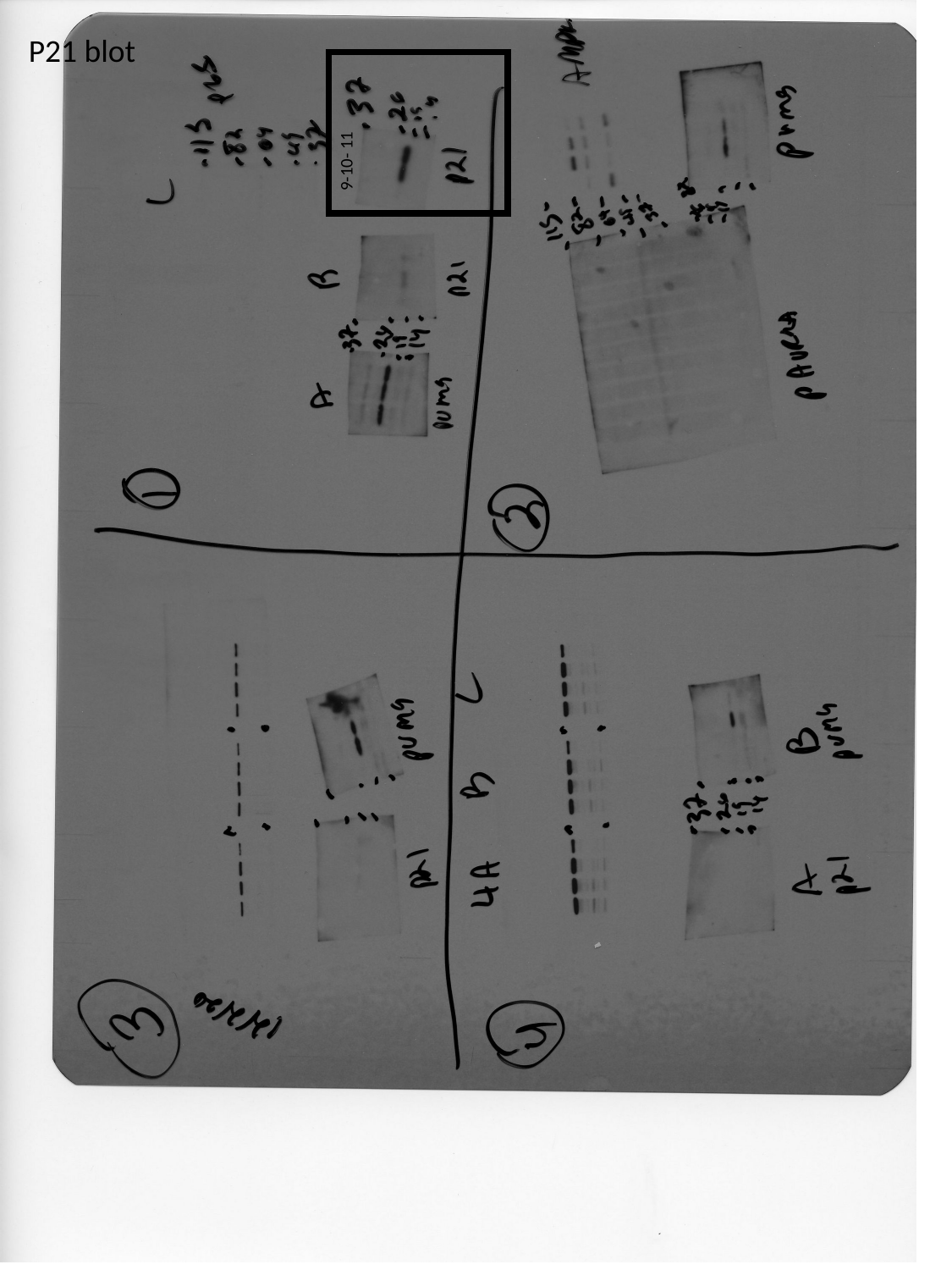

P21 blot
9-10- 11

## Slide 5
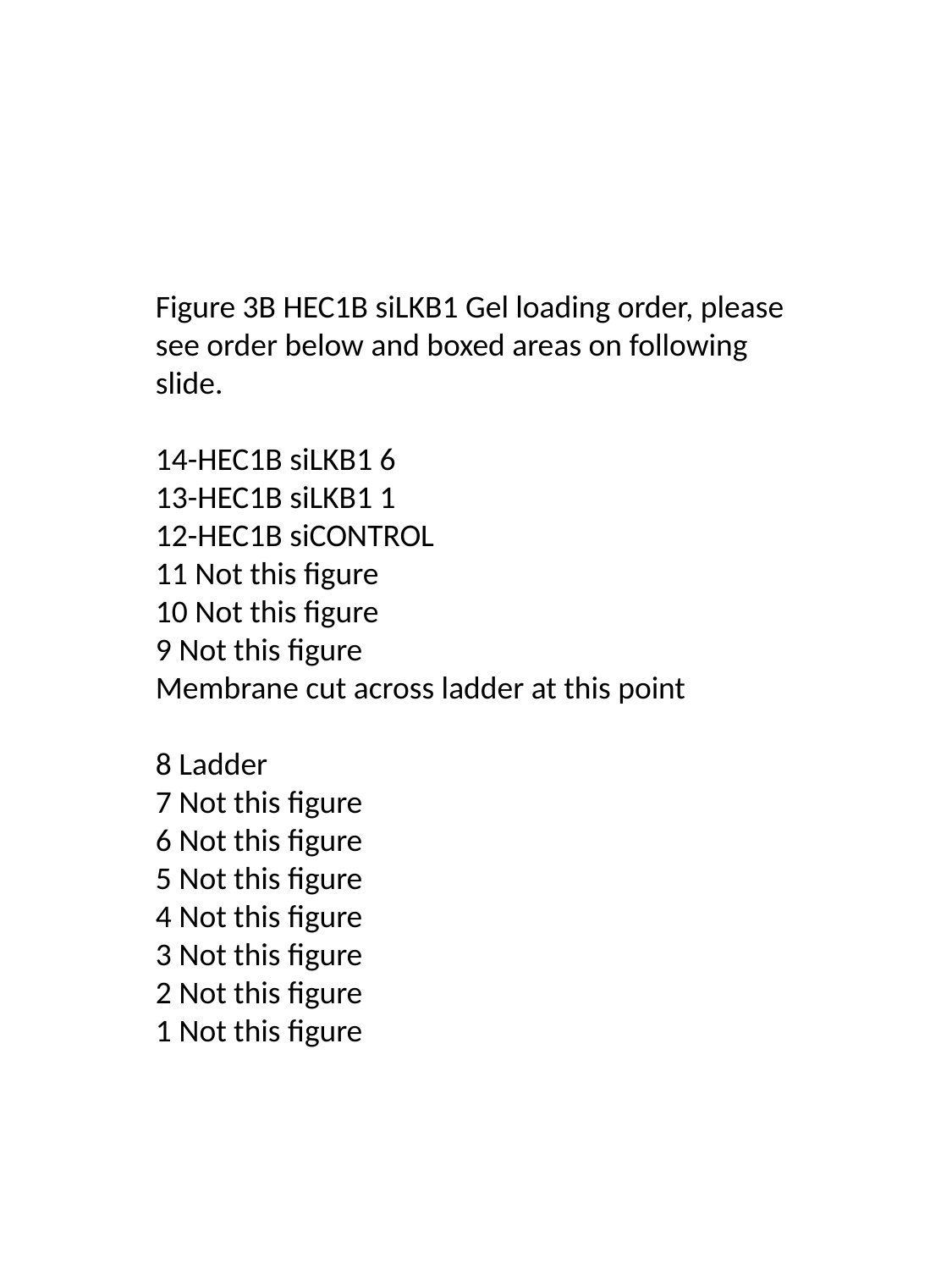

Figure 3B HEC1B siLKB1 Gel loading order, please see order below and boxed areas on following slide.
14-HEC1B siLKB1 6
13-HEC1B siLKB1 1
12-HEC1B siCONTROL
11 Not this figure
10 Not this figure
9 Not this figure
Membrane cut across ladder at this point
8 Ladder
7 Not this figure
6 Not this figure
5 Not this figure
4 Not this figure
3 Not this figure
2 Not this figure
1 Not this figure

## Slide 6
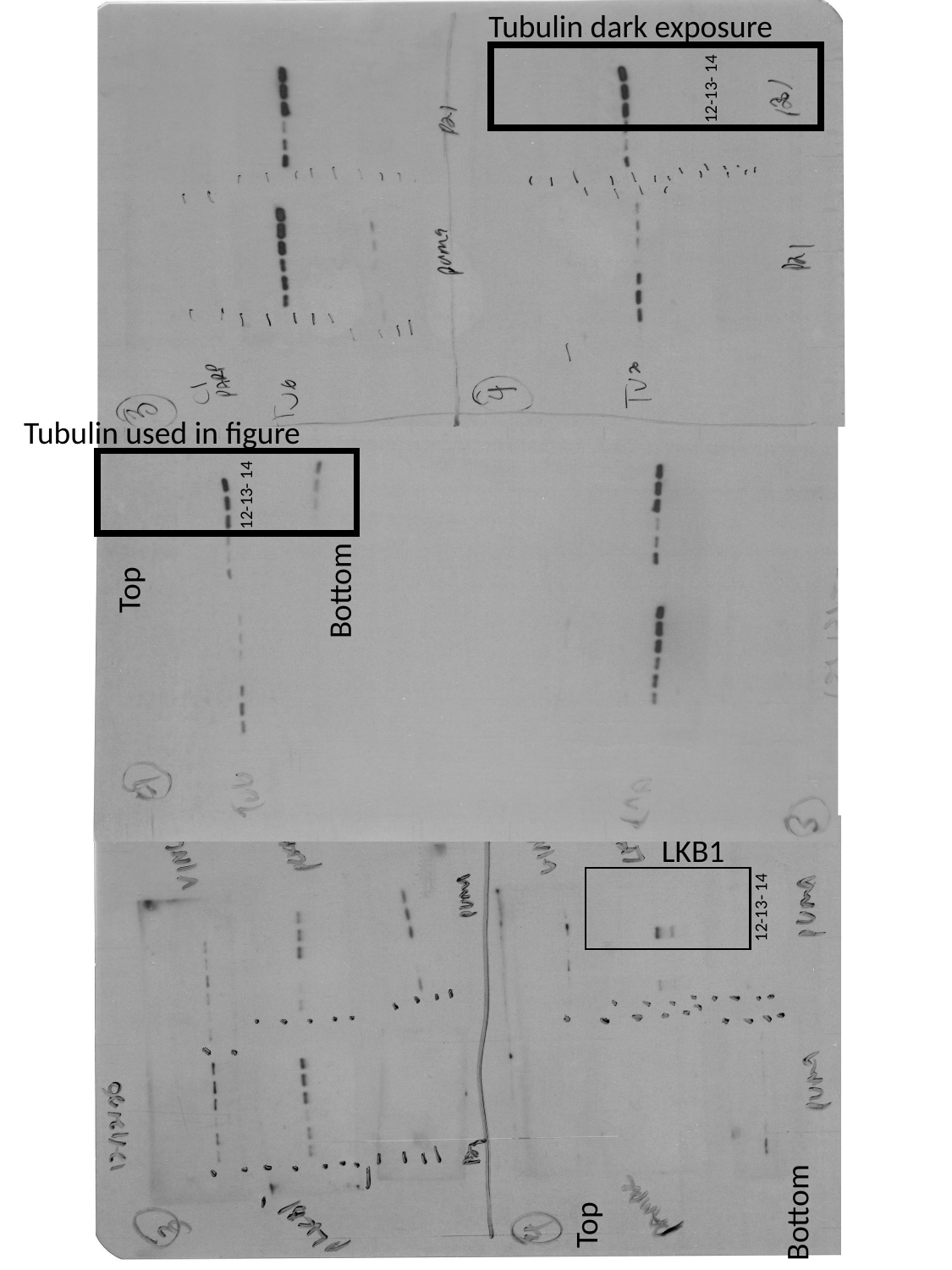

Tubulin dark exposure
12-13- 14
Tubulin used in figure
12-13- 14
Bottom
Top
LKB1
12-13- 14
Bottom
Top
