# Supplementary material for: Enhanced Efficacy of Aurora Kinase Inhibitors in G2/M Checkpoint Deficient TP53 Mutant Uterine Carcinomas Is Linked to the Summation of LKB1–AKT–p53 Interactions
Source: Cancers (Basel). 2021 May 3;13(9):2195. doi: 10.3390/cancers13092195 (PMC8125555; doi:10.3390/cancers13092195)
Supplement: Supplementary file 1 [file cancers-13-02195-s001.zip › Lynch and Hill Supplementary Matierals/original blot/Figure 3C.pptx]

## Slide 1
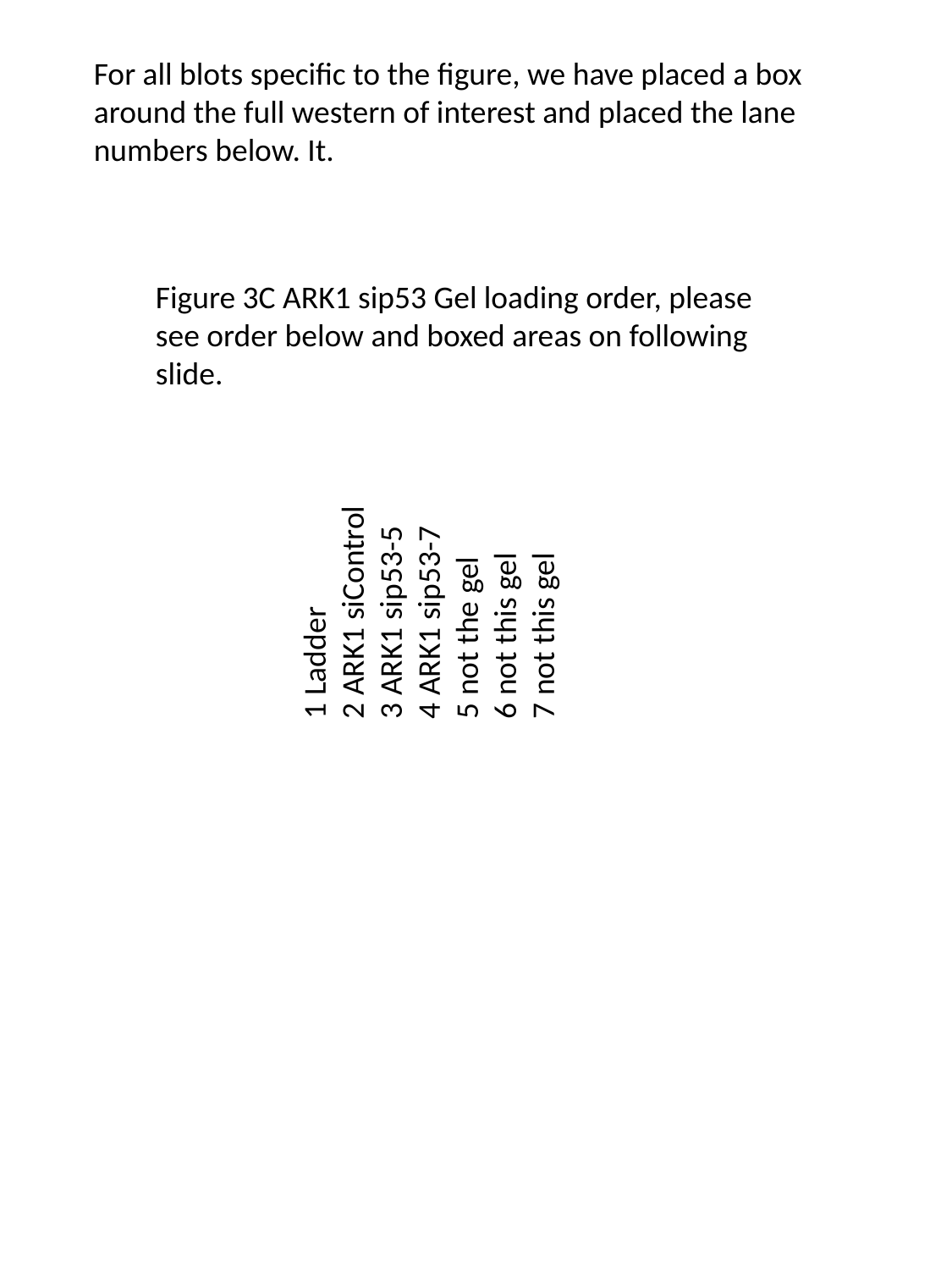

For all blots specific to the figure, we have placed a box around the full western of interest and placed the lane numbers below. It.
1 Ladder
2 ARK1 siControl
3 ARK1 sip53-5
4 ARK1 sip53-7
5 not the gel
6 not this gel
7 not this gel
Figure 3C ARK1 sip53 Gel loading order, please see order below and boxed areas on following slide.

## Slide 2
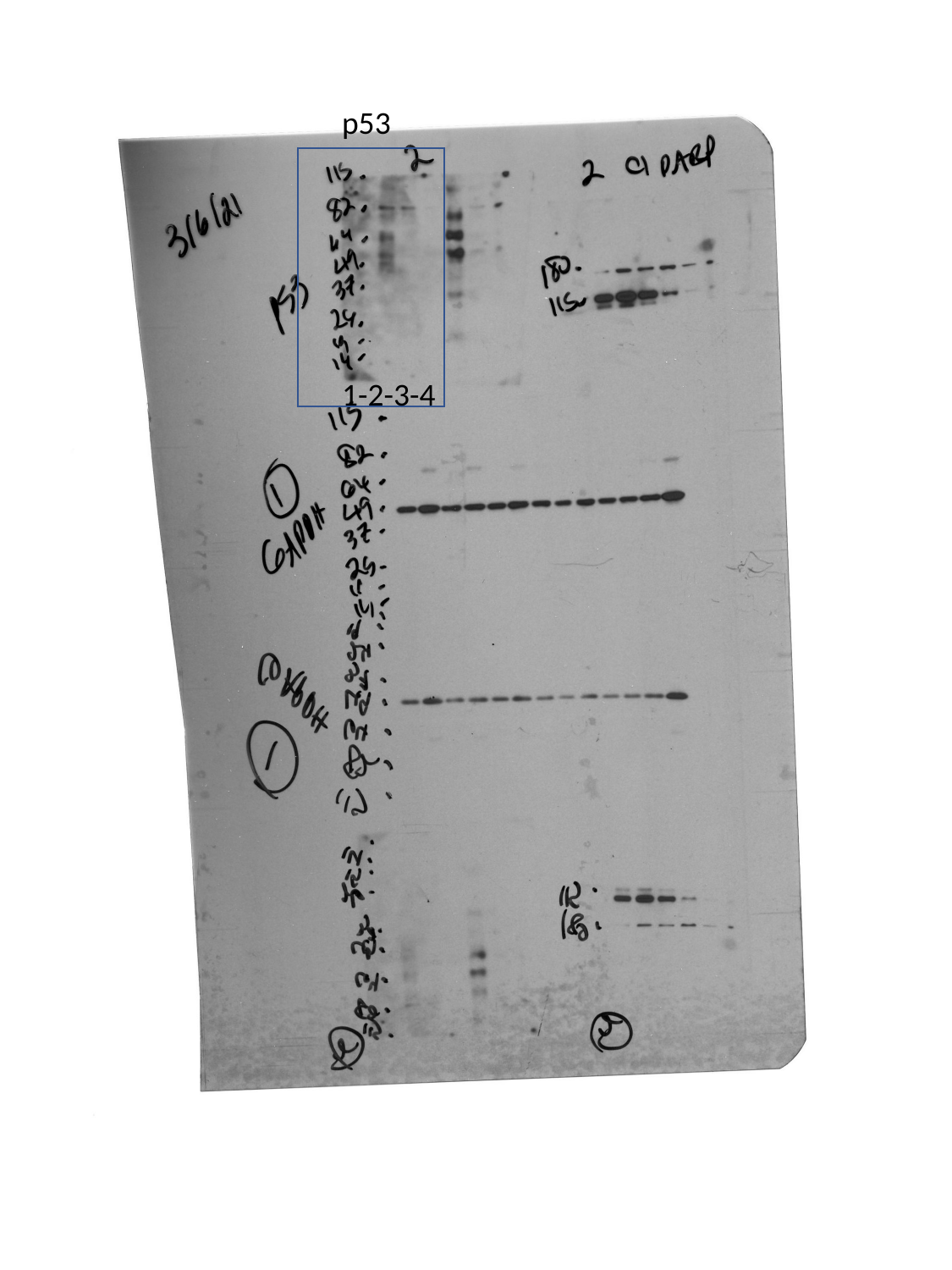

p53
1-2-3-4

## Slide 3
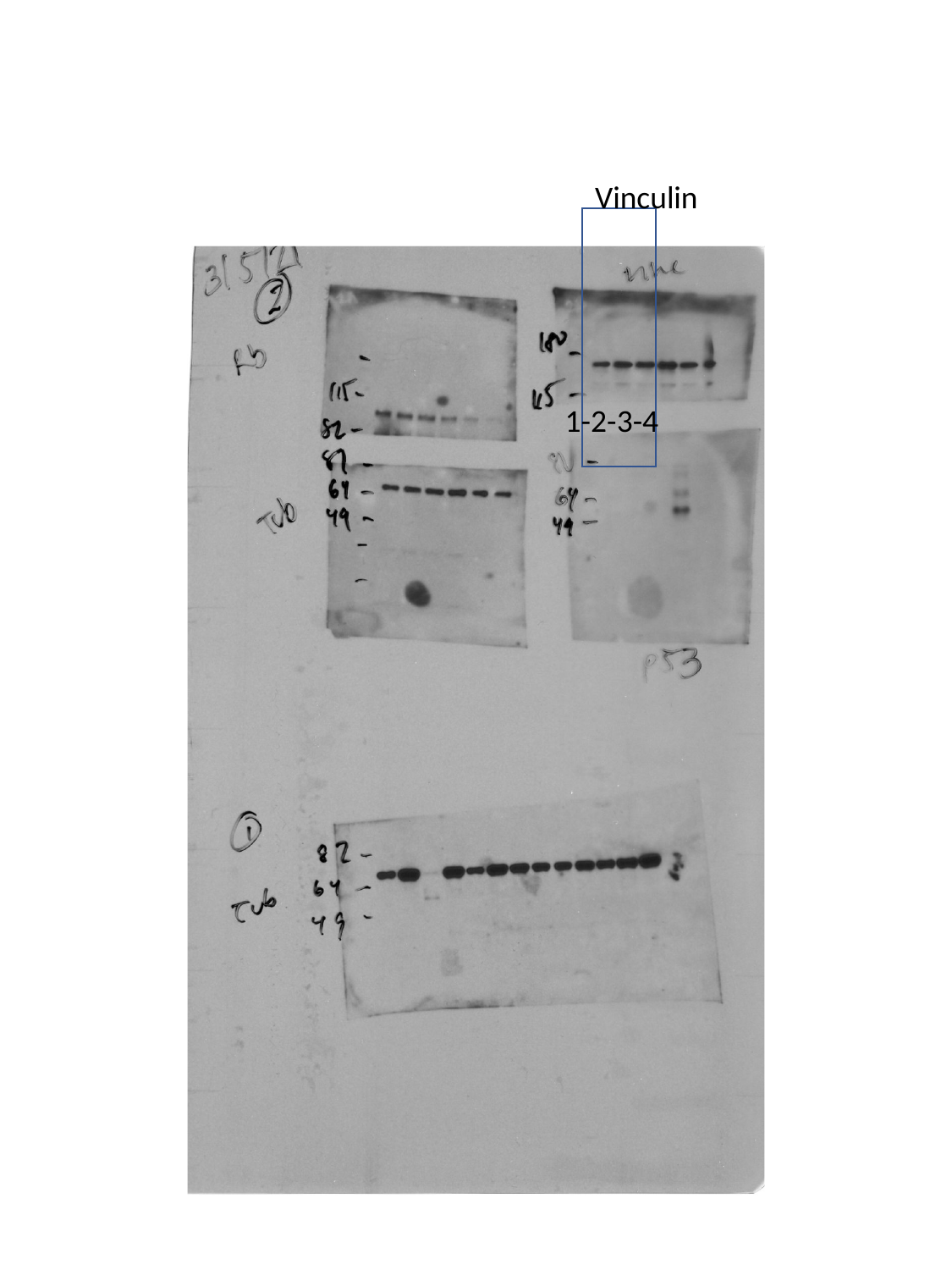

Vinculin
1-2-3-4

## Slide 4
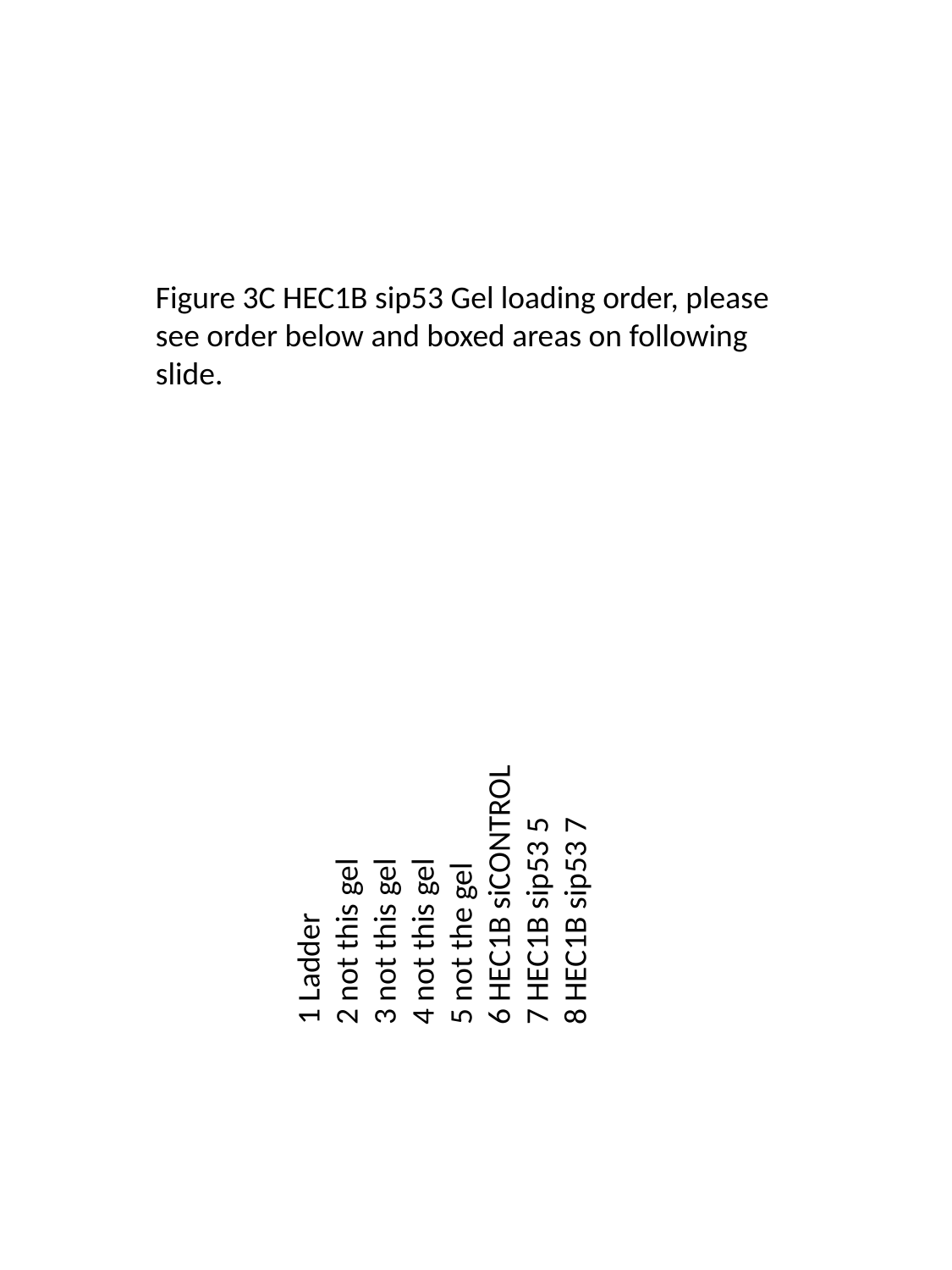

Figure 3C HEC1B sip53 Gel loading order, please see order below and boxed areas on following slide.
1 Ladder
2 not this gel
3 not this gel
4 not this gel
5 not the gel
6 HEC1B siCONTROL
7 HEC1B sip53 5
8 HEC1B sip53 7

## Slide 5
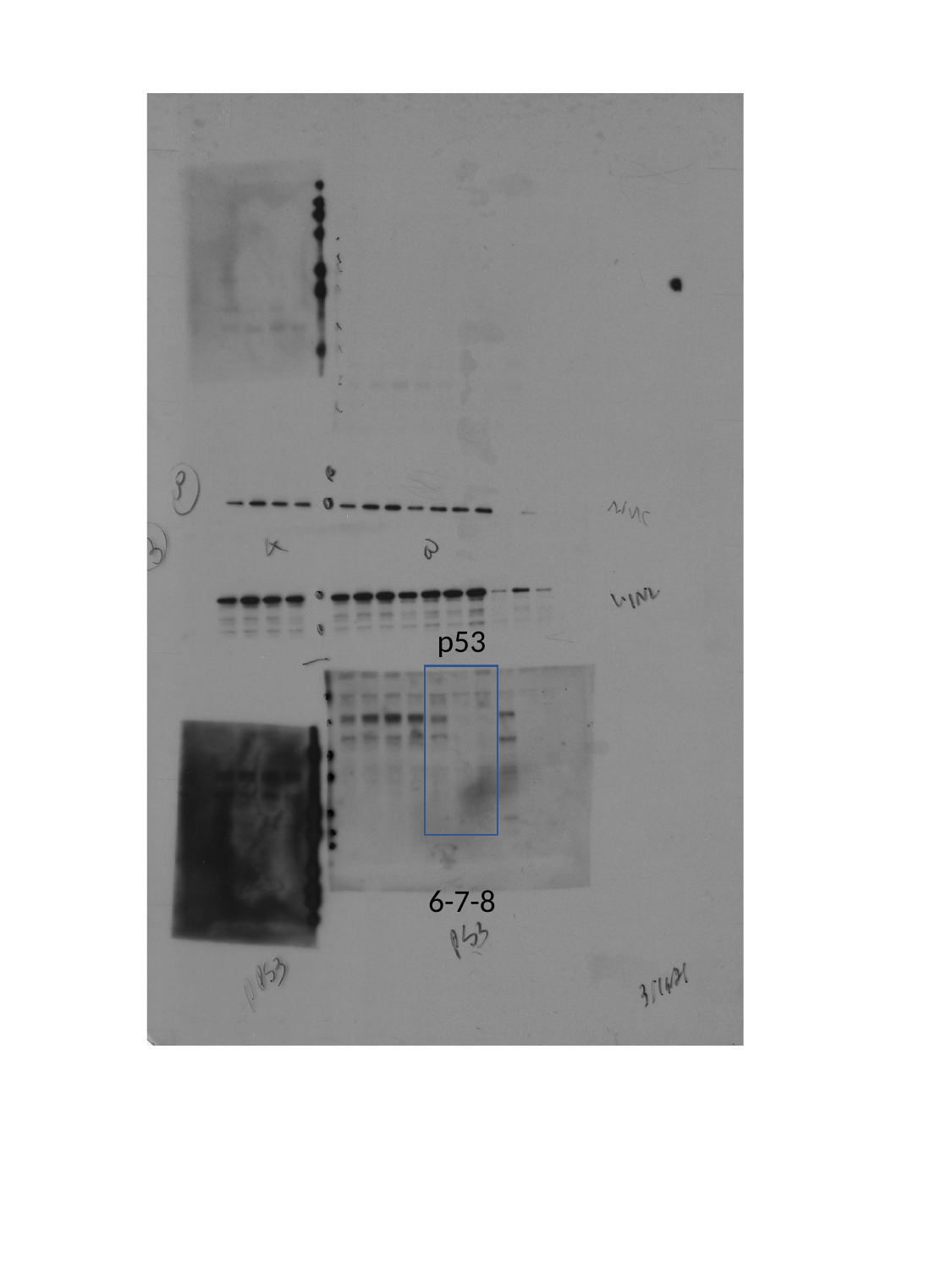

p53
6-7-8

## Slide 6
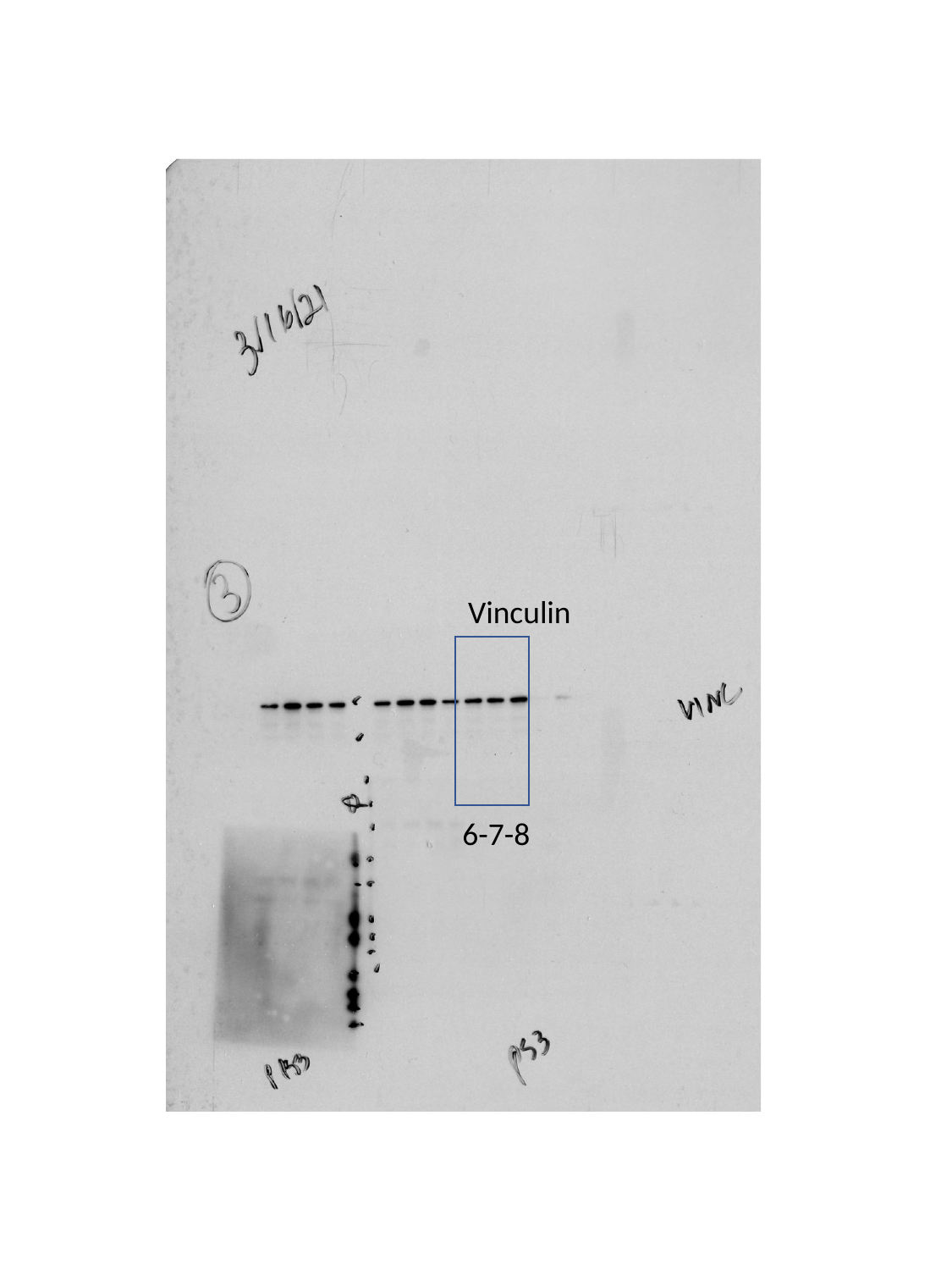

Vinculin
6-7-8
