# Supplementary material for: Enhanced Efficacy of Aurora Kinase Inhibitors in G2/M Checkpoint Deficient TP53 Mutant Uterine Carcinomas Is Linked to the Summation of LKB1–AKT–p53 Interactions
Source: Cancers (Basel). 2021 May 3;13(9):2195. doi: 10.3390/cancers13092195 (PMC8125555; doi:10.3390/cancers13092195)
Supplement: Supplementary file 1 [file cancers-13-02195-s001.zip › Lynch and Hill Supplementary Matierals/original blot/Figure 3F.pptx]

## Slide 1
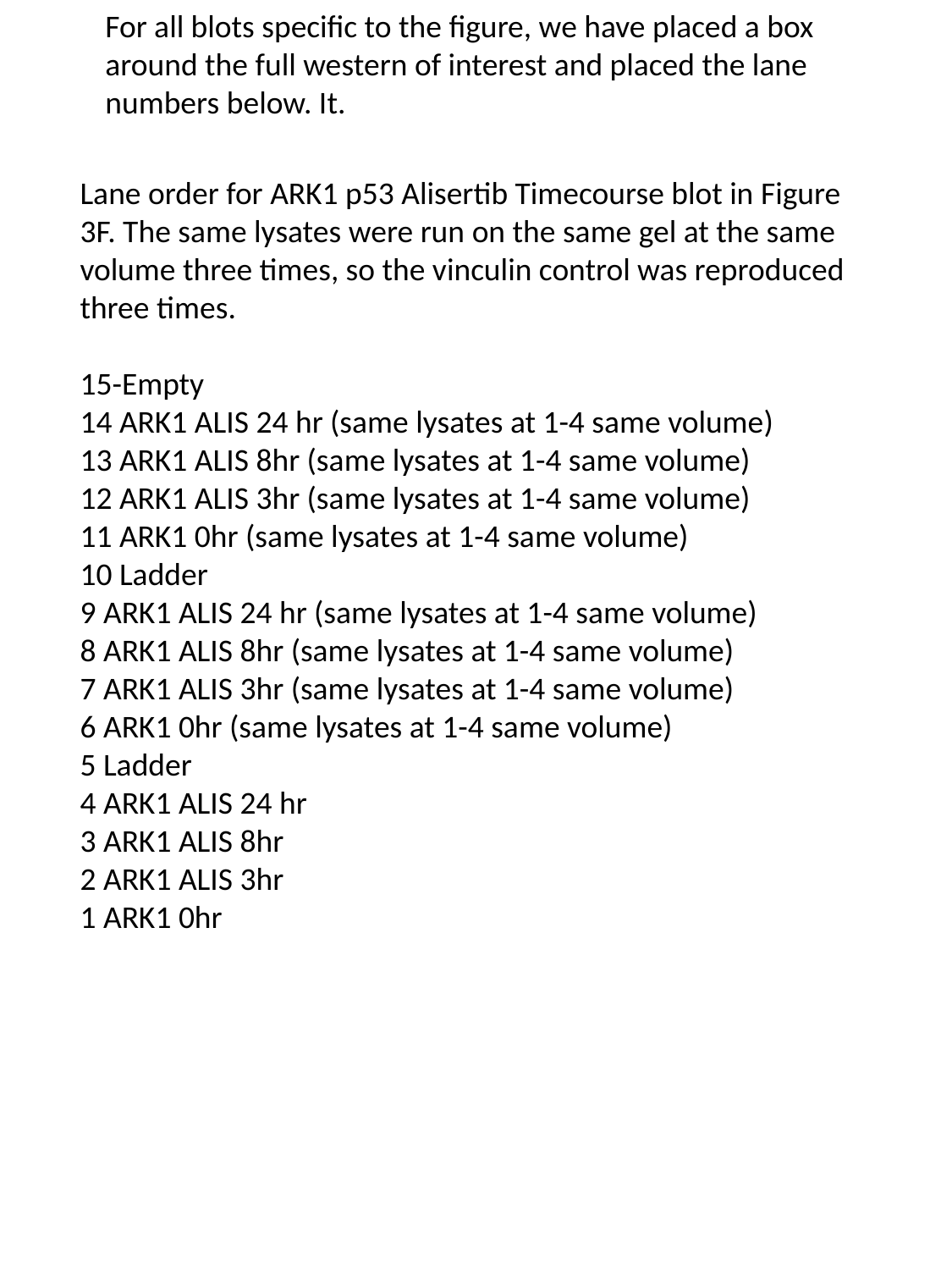

For all blots specific to the figure, we have placed a box around the full western of interest and placed the lane numbers below. It.
Lane order for ARK1 p53 Alisertib Timecourse blot in Figure 3F. The same lysates were run on the same gel at the same volume three times, so the vinculin control was reproduced three times.
15-Empty
14 ARK1 ALIS 24 hr (same lysates at 1-4 same volume)
13 ARK1 ALIS 8hr (same lysates at 1-4 same volume)
12 ARK1 ALIS 3hr (same lysates at 1-4 same volume)
11 ARK1 0hr (same lysates at 1-4 same volume)
10 Ladder
9 ARK1 ALIS 24 hr (same lysates at 1-4 same volume)
8 ARK1 ALIS 8hr (same lysates at 1-4 same volume)
7 ARK1 ALIS 3hr (same lysates at 1-4 same volume)
6 ARK1 0hr (same lysates at 1-4 same volume)
5 Ladder
4 ARK1 ALIS 24 hr
3 ARK1 ALIS 8hr
2 ARK1 ALIS 3hr
1 ARK1 0hr

## Slide 2
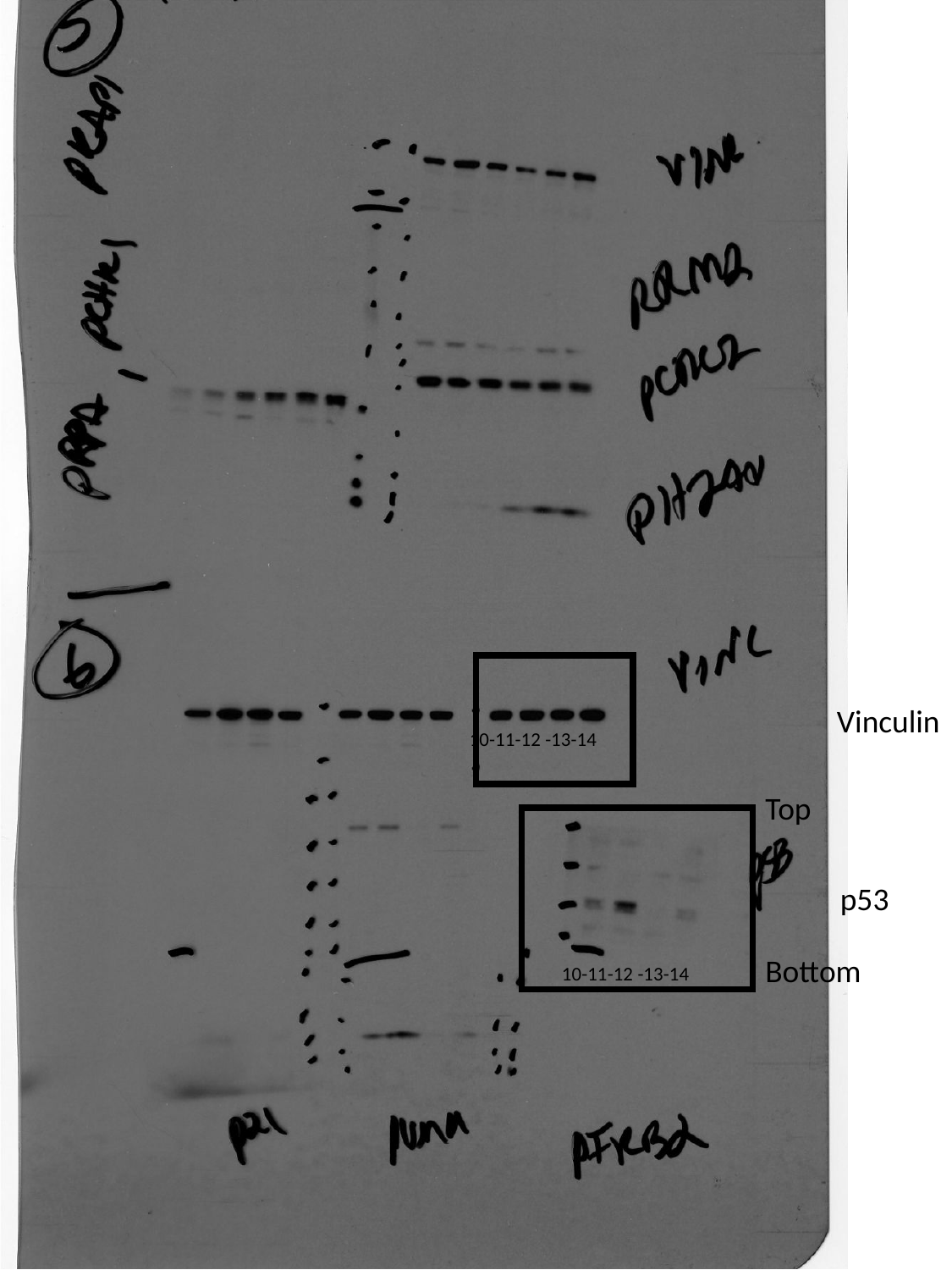

Vinculin
10-11-12 -13-14
Top
p53
Bottom
10-11-12 -13-14

## Slide 3
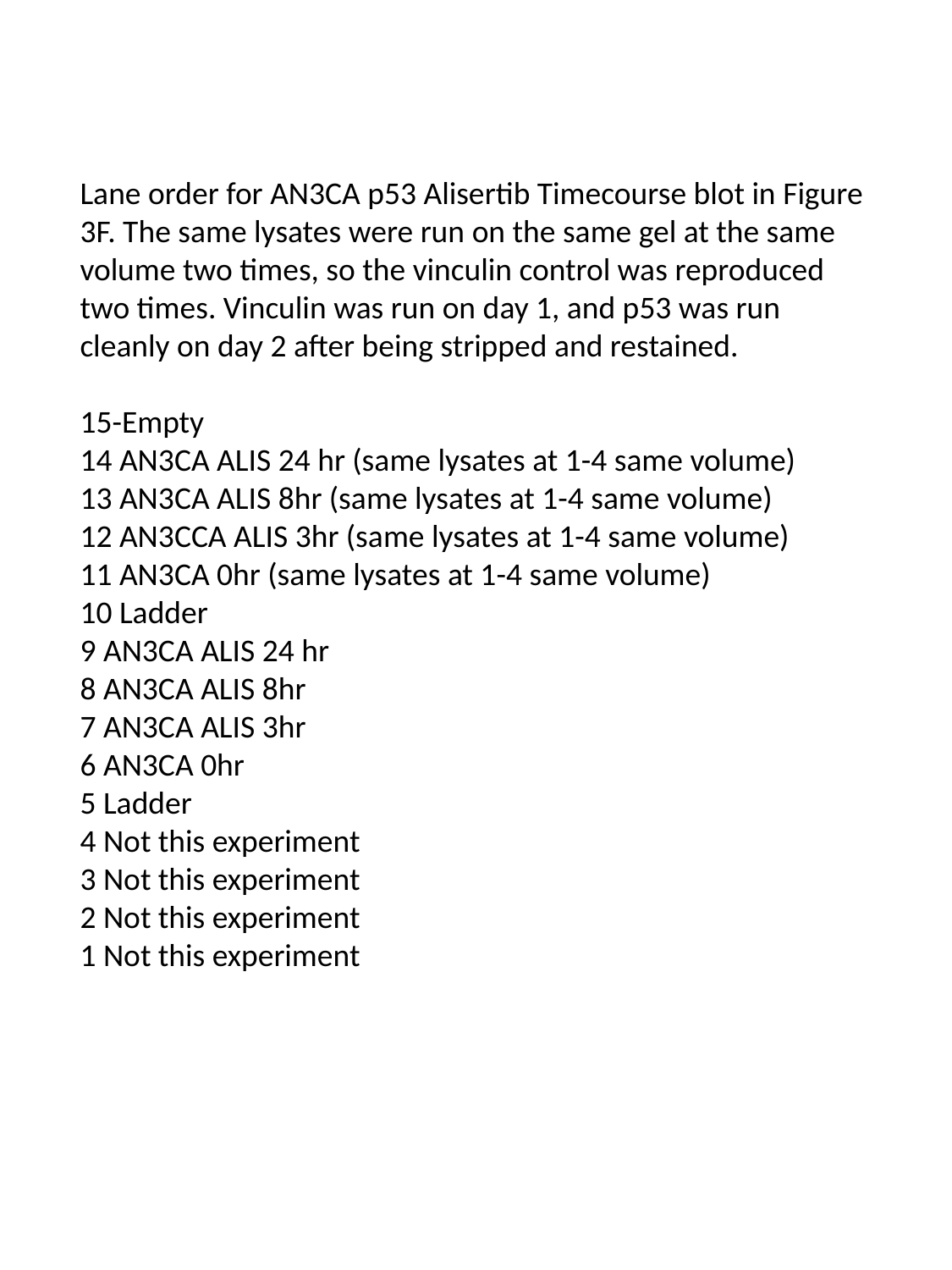

Lane order for AN3CA p53 Alisertib Timecourse blot in Figure 3F. The same lysates were run on the same gel at the same volume two times, so the vinculin control was reproduced two times. Vinculin was run on day 1, and p53 was run cleanly on day 2 after being stripped and restained.
15-Empty
14 AN3CA ALIS 24 hr (same lysates at 1-4 same volume)
13 AN3CA ALIS 8hr (same lysates at 1-4 same volume)
12 AN3CCA ALIS 3hr (same lysates at 1-4 same volume)
11 AN3CA 0hr (same lysates at 1-4 same volume)
10 Ladder
9 AN3CA ALIS 24 hr
8 AN3CA ALIS 8hr
7 AN3CA ALIS 3hr
6 AN3CA 0hr
5 Ladder
4 Not this experiment
3 Not this experiment
2 Not this experiment
1 Not this experiment

## Slide 4
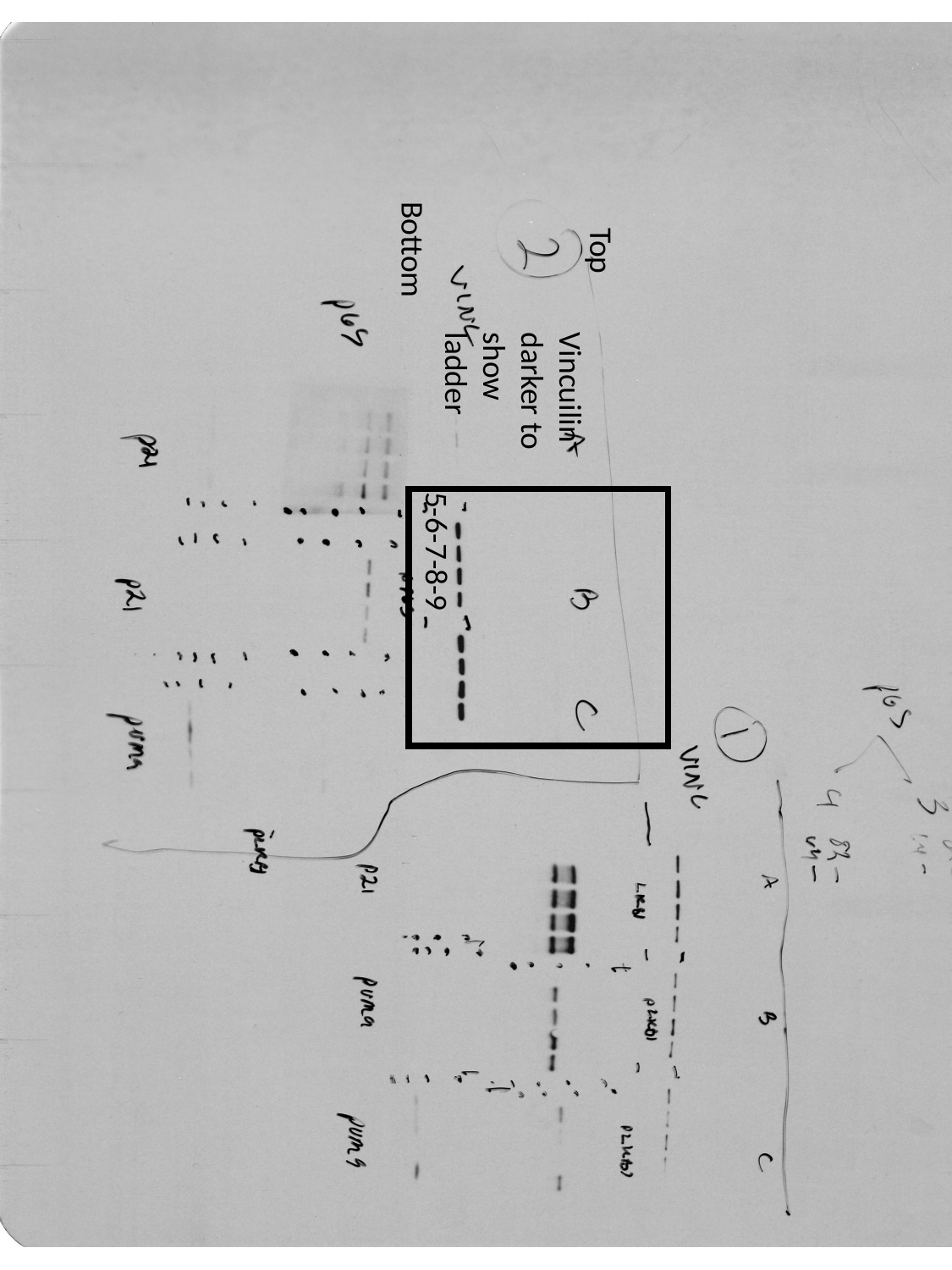

Bottom
Top
Vincuilin darker to show ladder
5-6-7-8-9

## Slide 5
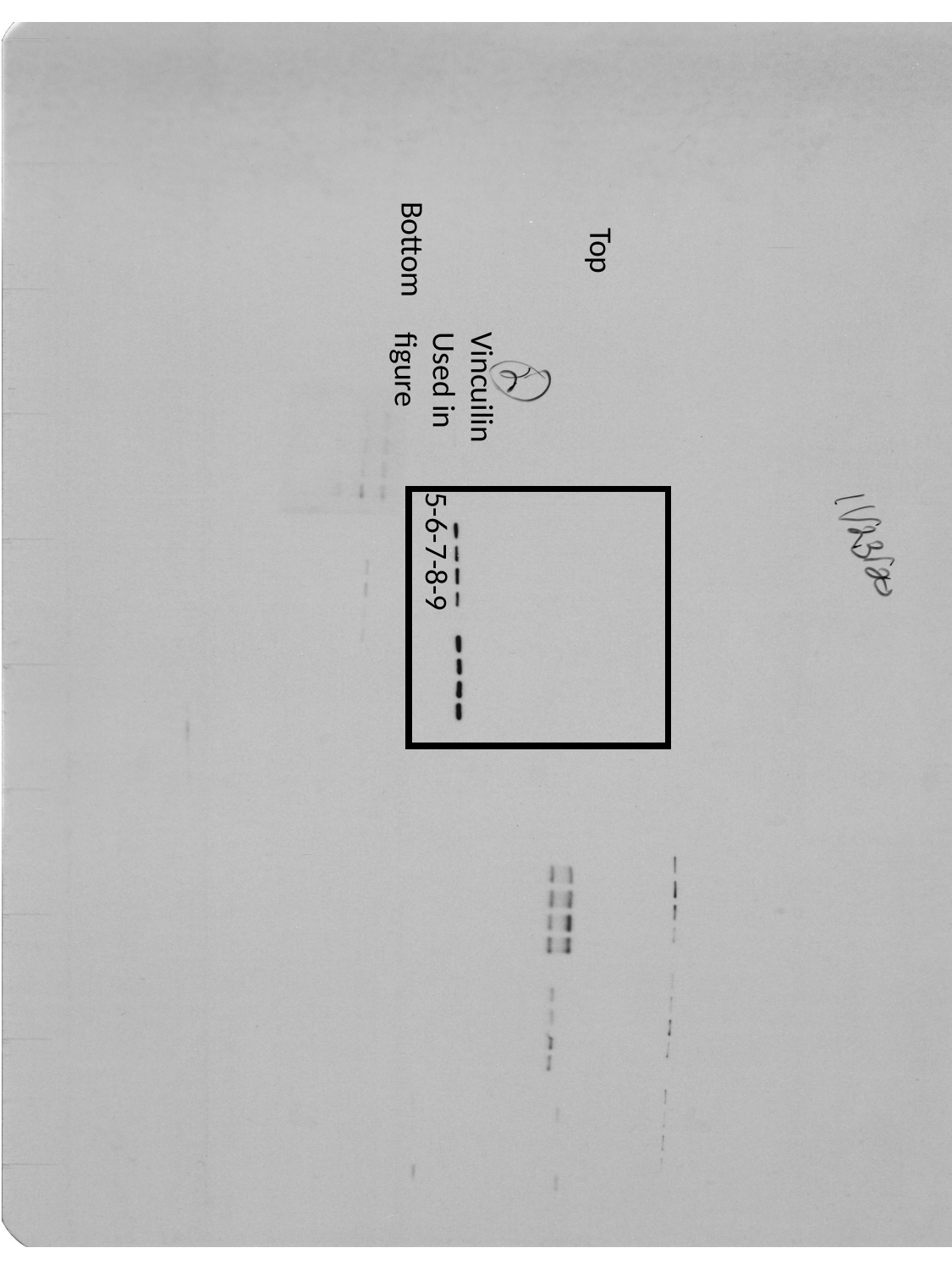

Bottom
Top
Vincuilin
Used in figure
5-6-7-8-9

## Slide 6
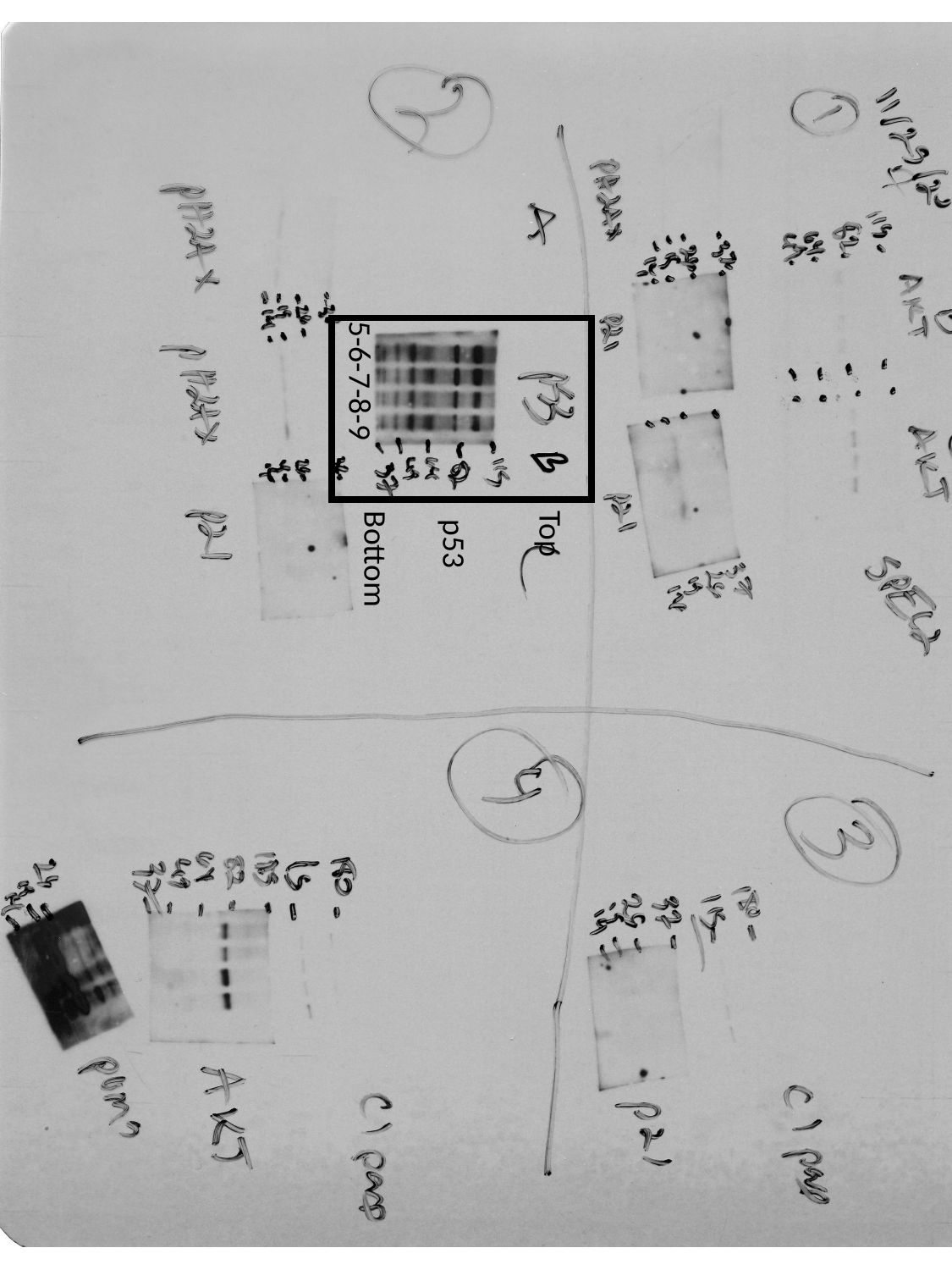

5-6-7-8-9
Top
Bottom
p53

## Slide 7
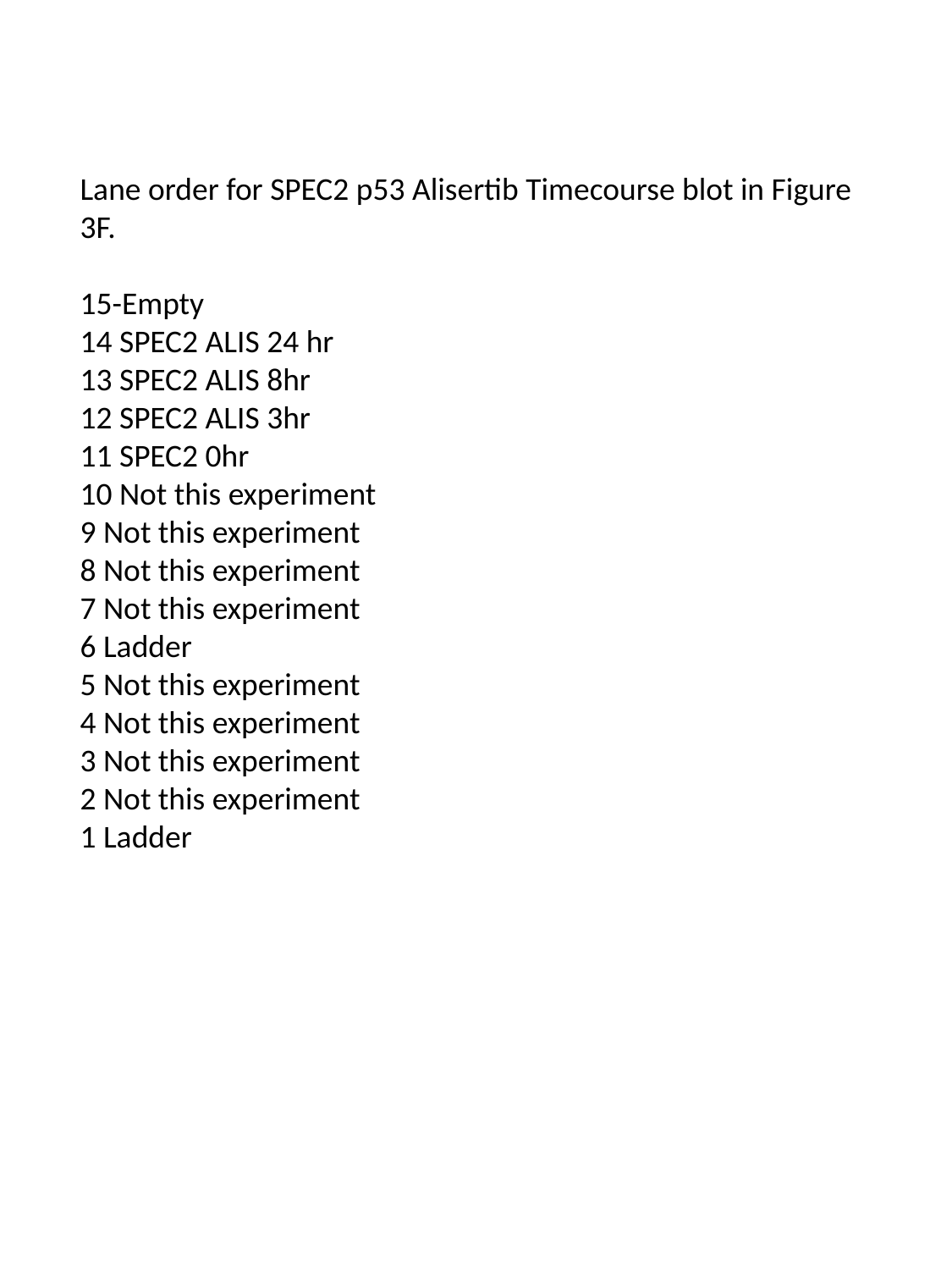

Lane order for SPEC2 p53 Alisertib Timecourse blot in Figure 3F.
15-Empty
14 SPEC2 ALIS 24 hr
13 SPEC2 ALIS 8hr
12 SPEC2 ALIS 3hr
11 SPEC2 0hr
10 Not this experiment
9 Not this experiment
8 Not this experiment
7 Not this experiment
6 Ladder
5 Not this experiment
4 Not this experiment
3 Not this experiment
2 Not this experiment
1 Ladder

## Slide 8
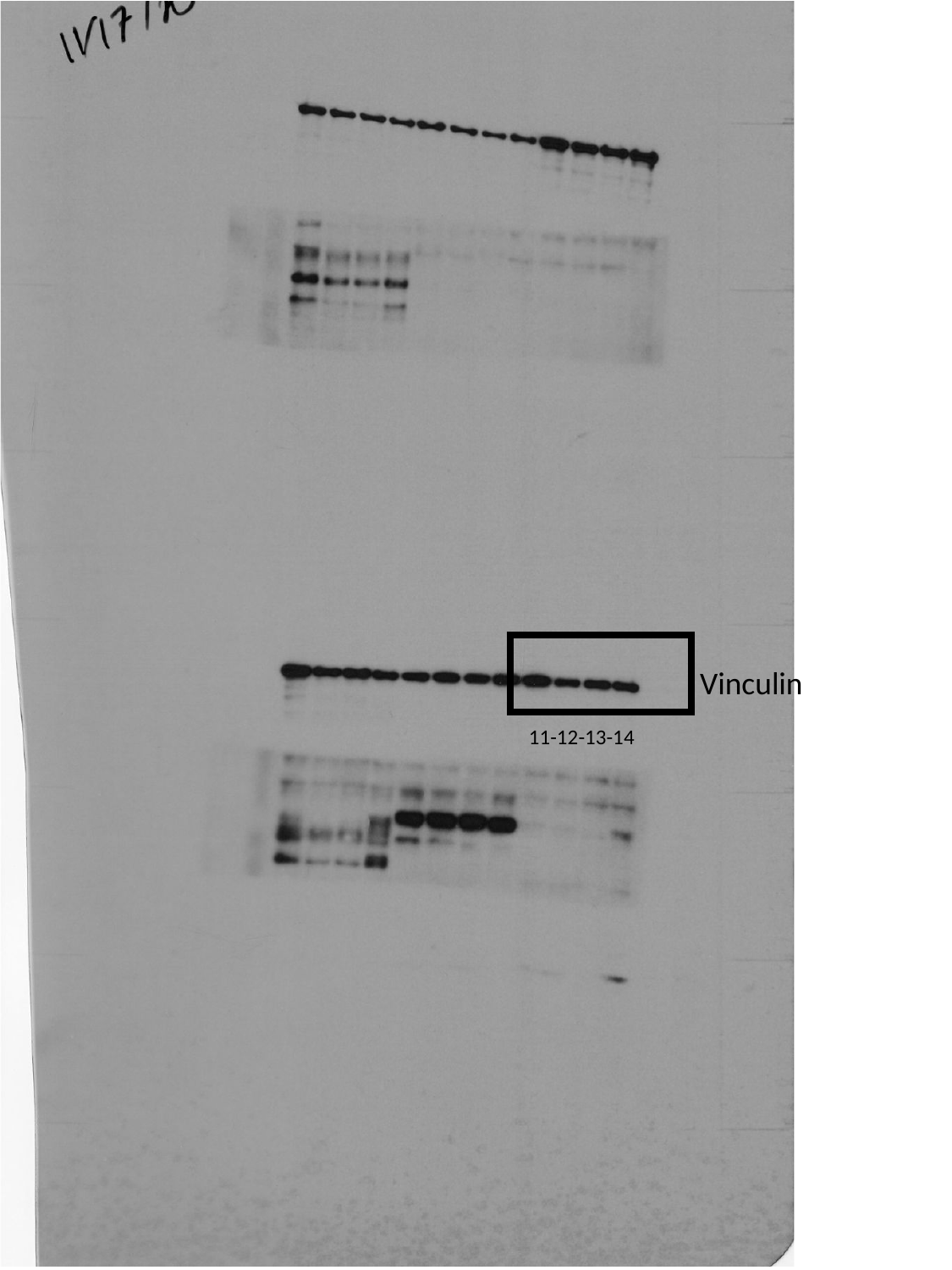

Vinculin
11-12-13-14

## Slide 9
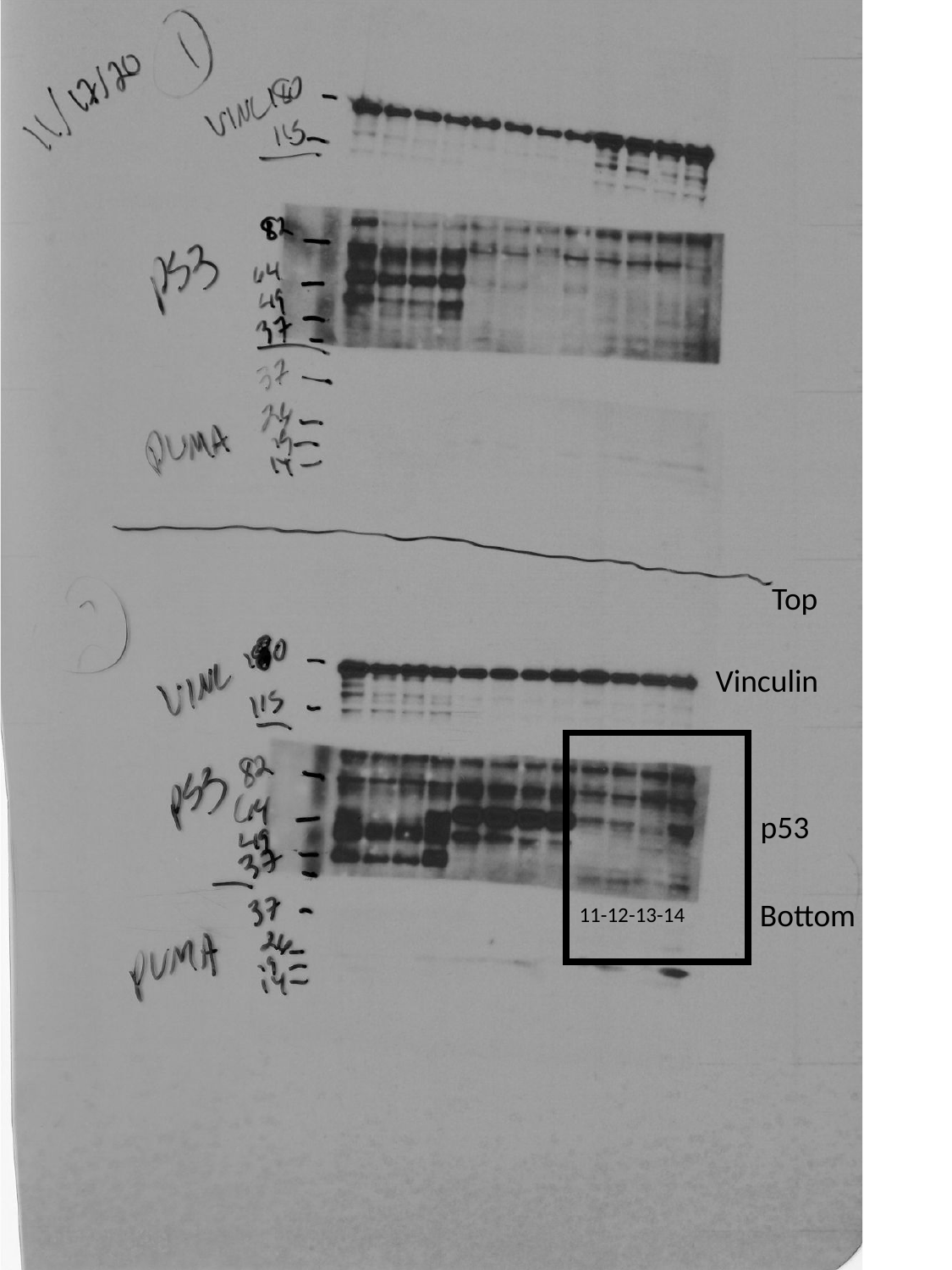

Top
Vinculin
p53
Bottom
11-12-13-14

## Slide 10
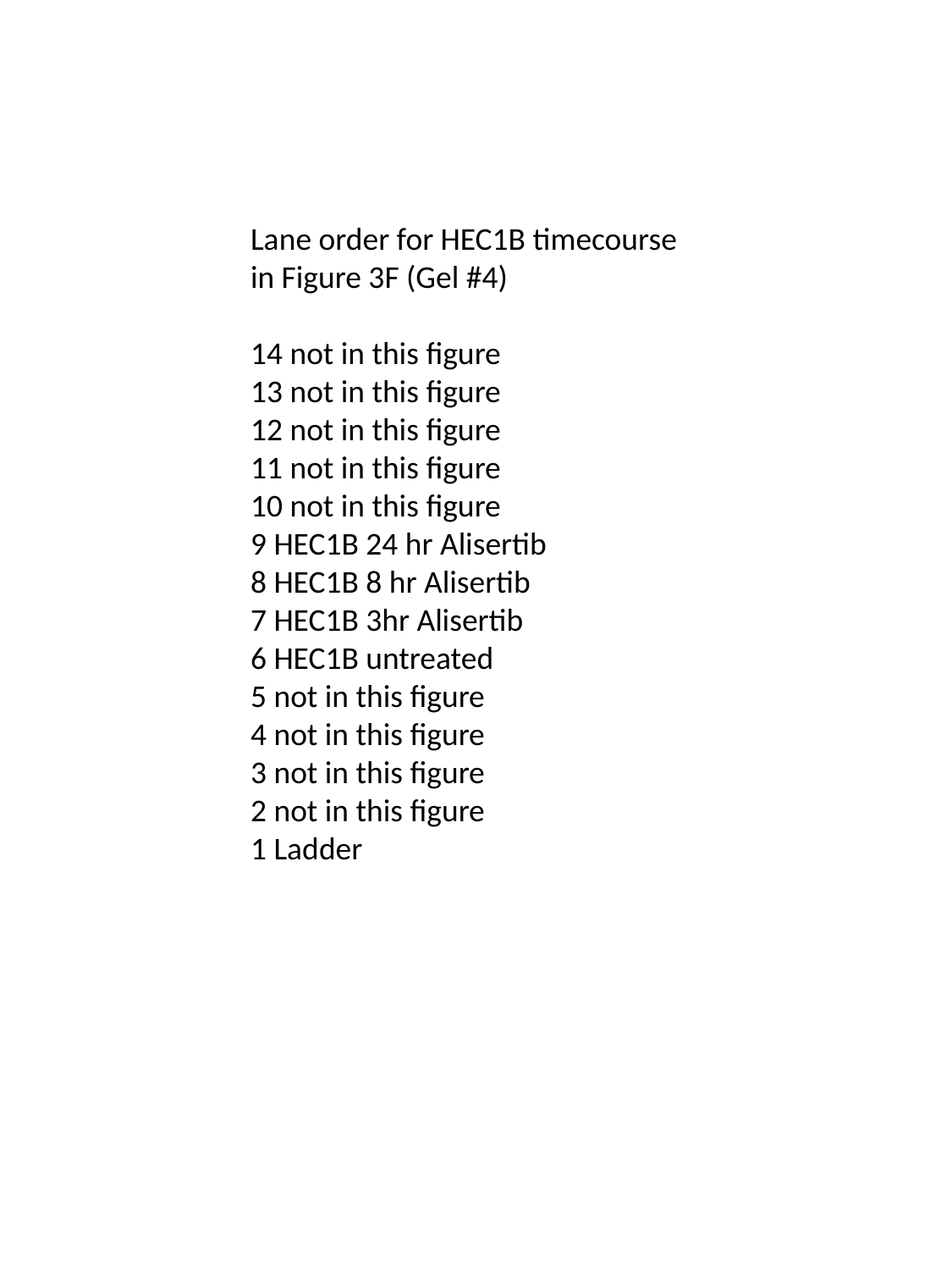

Lane order for HEC1B timecourse in Figure 3F (Gel #4)
14 not in this figure
13 not in this figure
12 not in this figure
11 not in this figure
10 not in this figure
9 HEC1B 24 hr Alisertib
8 HEC1B 8 hr Alisertib
7 HEC1B 3hr Alisertib
6 HEC1B untreated
5 not in this figure
4 not in this figure
3 not in this figure
2 not in this figure
1 Ladder

## Slide 11
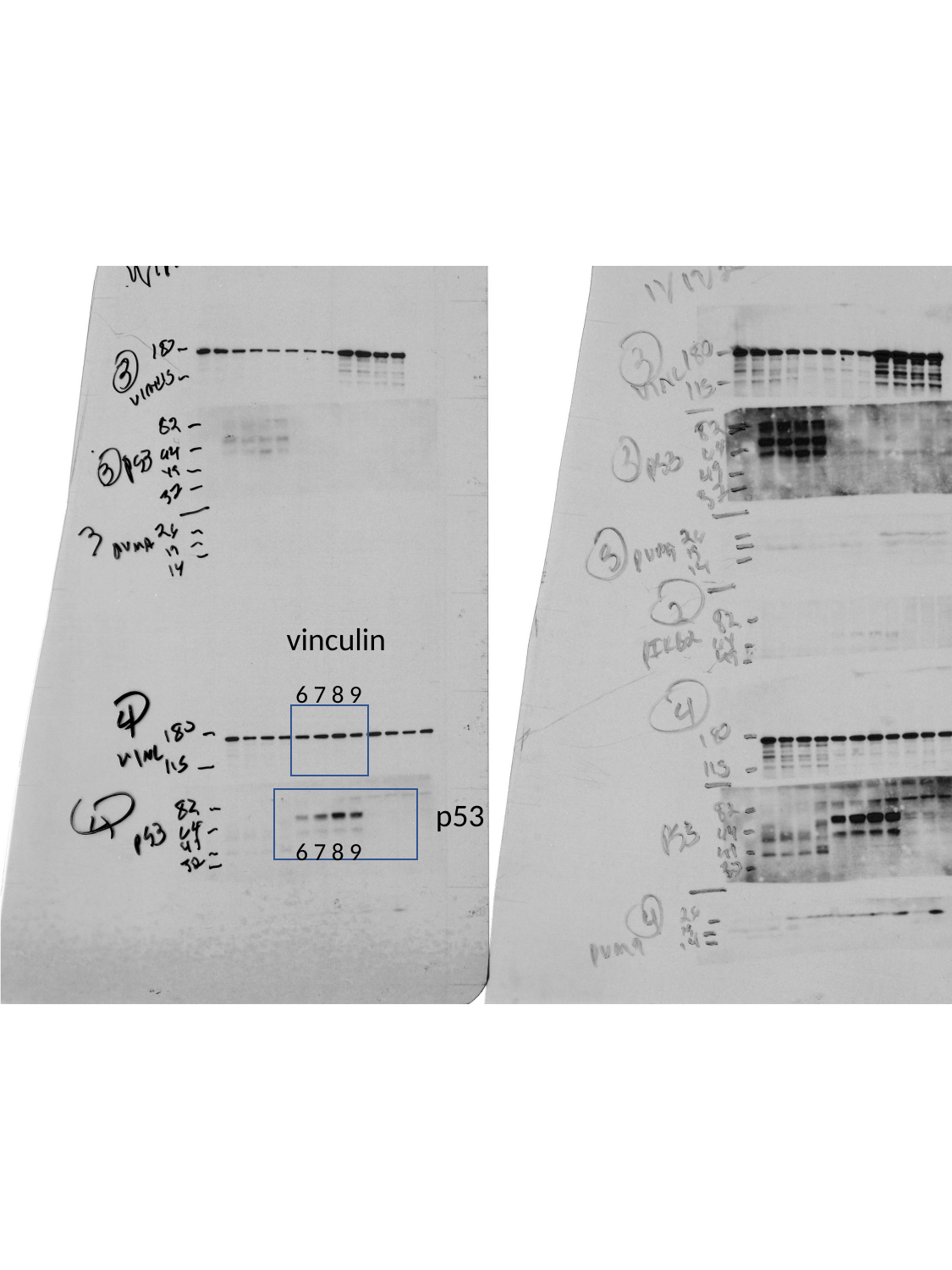

vinculin
6 7 8 9
p53
6 7 8 9
