# Supplementary material for: Enhanced Efficacy of Aurora Kinase Inhibitors in G2/M Checkpoint Deficient TP53 Mutant Uterine Carcinomas Is Linked to the Summation of LKB1–AKT–p53 Interactions
Source: Cancers (Basel). 2021 May 3;13(9):2195. doi: 10.3390/cancers13092195 (PMC8125555; doi:10.3390/cancers13092195)
Supplement: Supplementary file 1 [file cancers-13-02195-s001.zip › Lynch and Hill Supplementary Matierals/original blot/Figure 3G.pptx]

## Slide 1
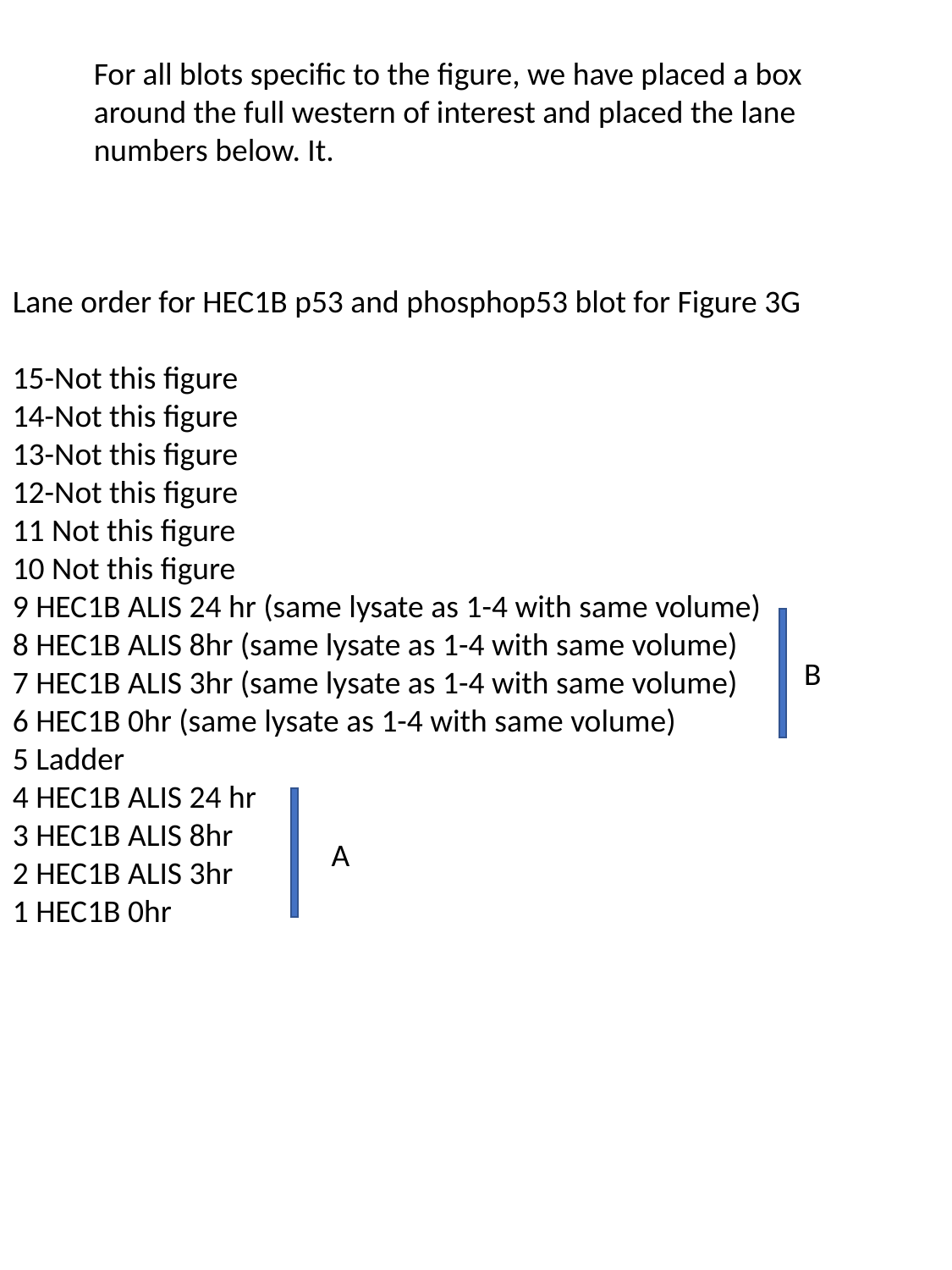

For all blots specific to the figure, we have placed a box around the full western of interest and placed the lane numbers below. It.
Lane order for HEC1B p53 and phosphop53 blot for Figure 3G
15-Not this figure
14-Not this figure
13-Not this figure
12-Not this figure
11 Not this figure
10 Not this figure
9 HEC1B ALIS 24 hr (same lysate as 1-4 with same volume)
8 HEC1B ALIS 8hr (same lysate as 1-4 with same volume)
7 HEC1B ALIS 3hr (same lysate as 1-4 with same volume)
6 HEC1B 0hr (same lysate as 1-4 with same volume)
5 Ladder
4 HEC1B ALIS 24 hr
3 HEC1B ALIS 8hr
2 HEC1B ALIS 3hr
1 HEC1B 0hr
B
A

## Slide 2
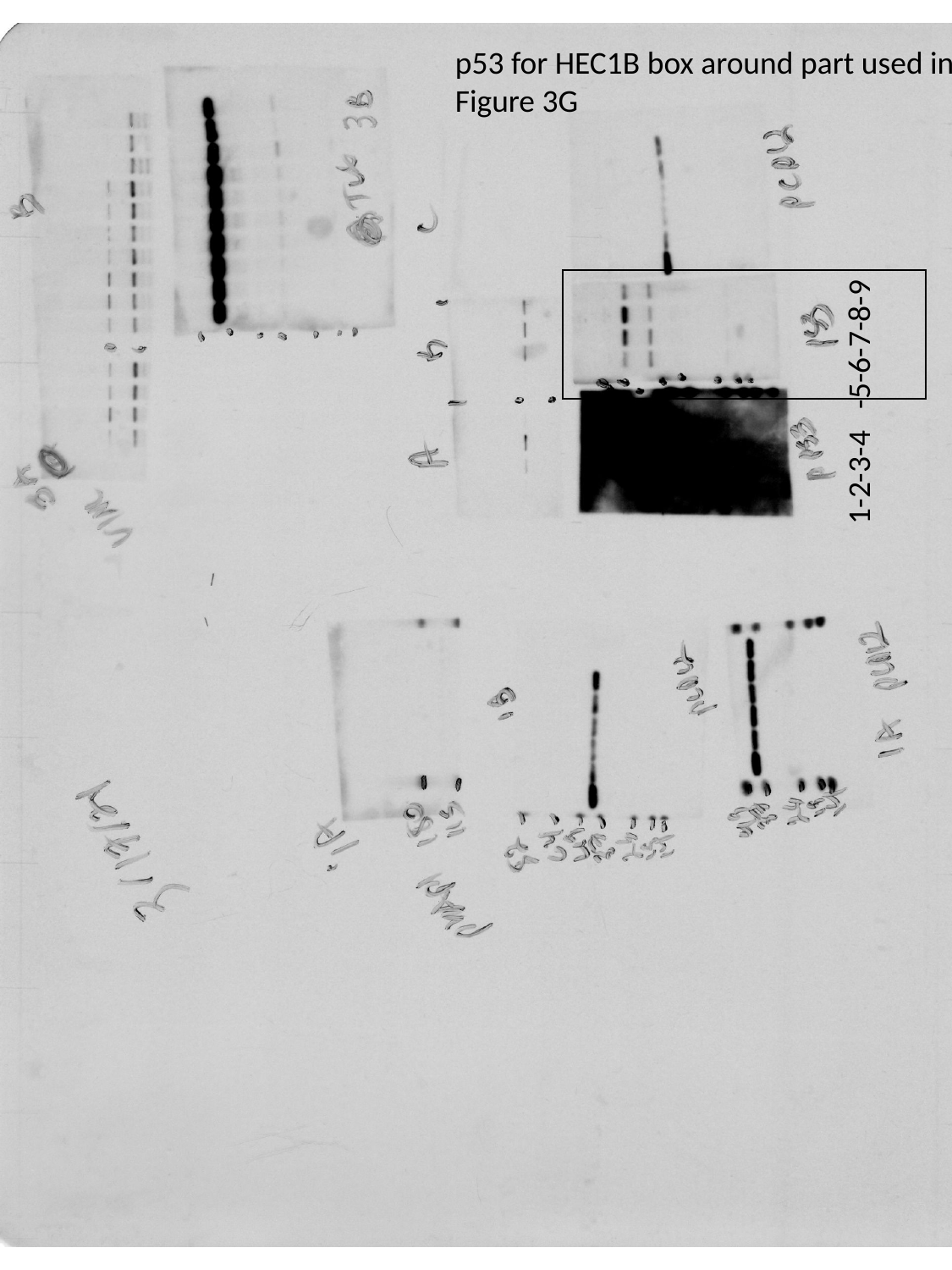

p53 for HEC1B box around part used in Figure 3G
1-2-3-4 -5-6-7-8-9

## Slide 3
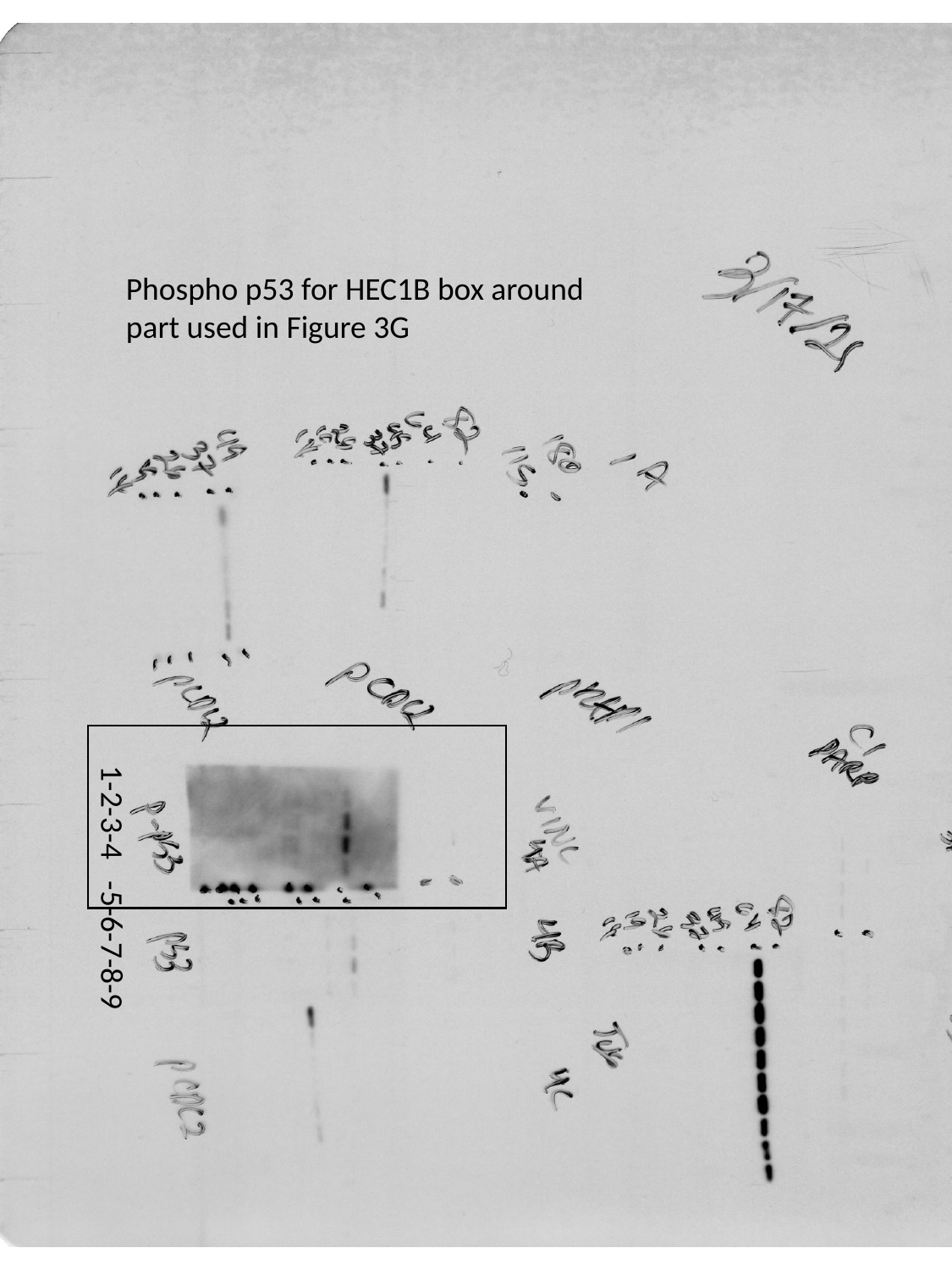

Phospho p53 for HEC1B box around part used in Figure 3G
1-2-3-4 -5-6-7-8-9

## Slide 4
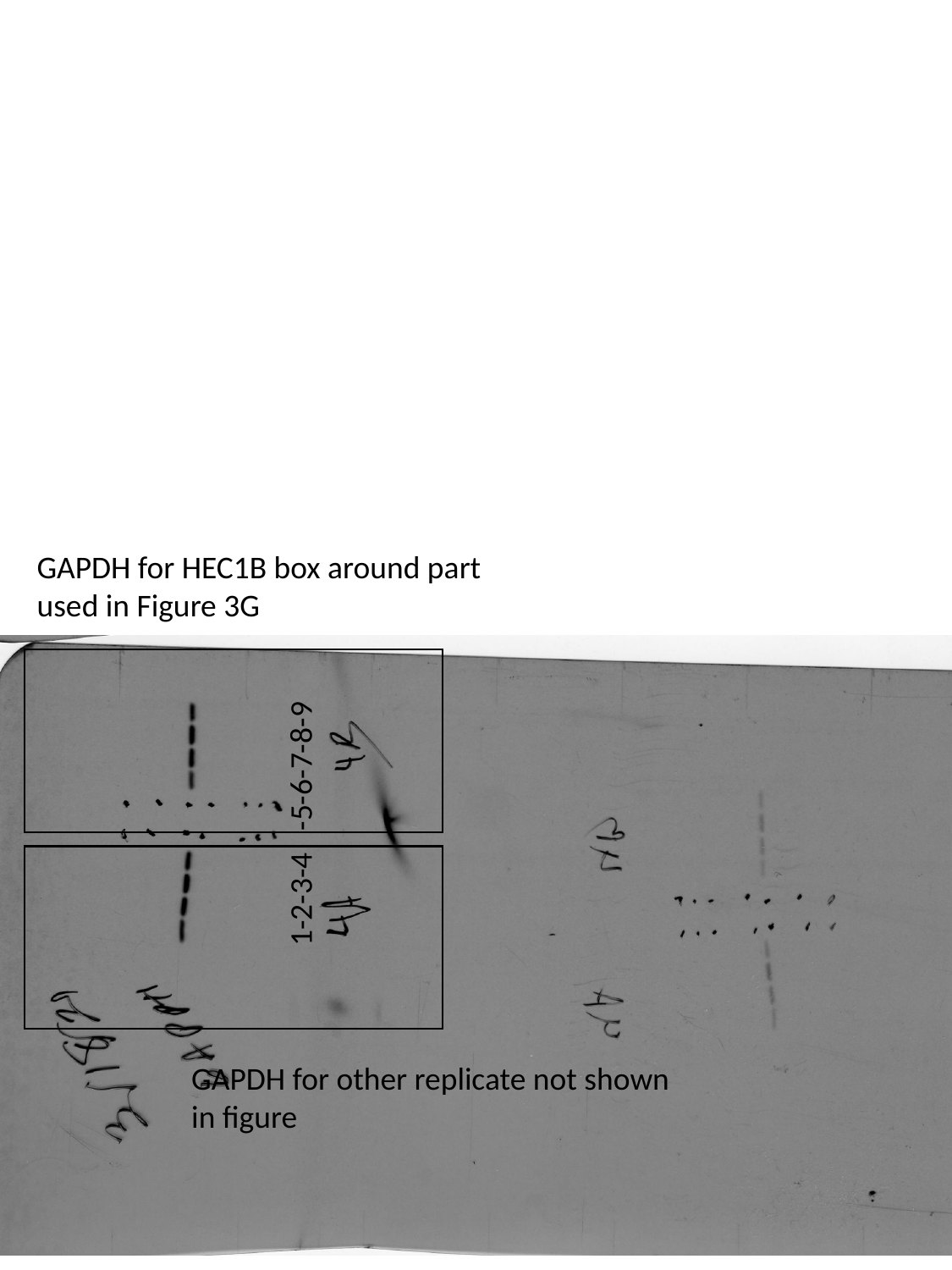

GAPDH for HEC1B box around part used in Figure 3G
1-2-3-4 -5-6-7-8-9
GAPDH for other replicate not shown in figure

## Slide 5
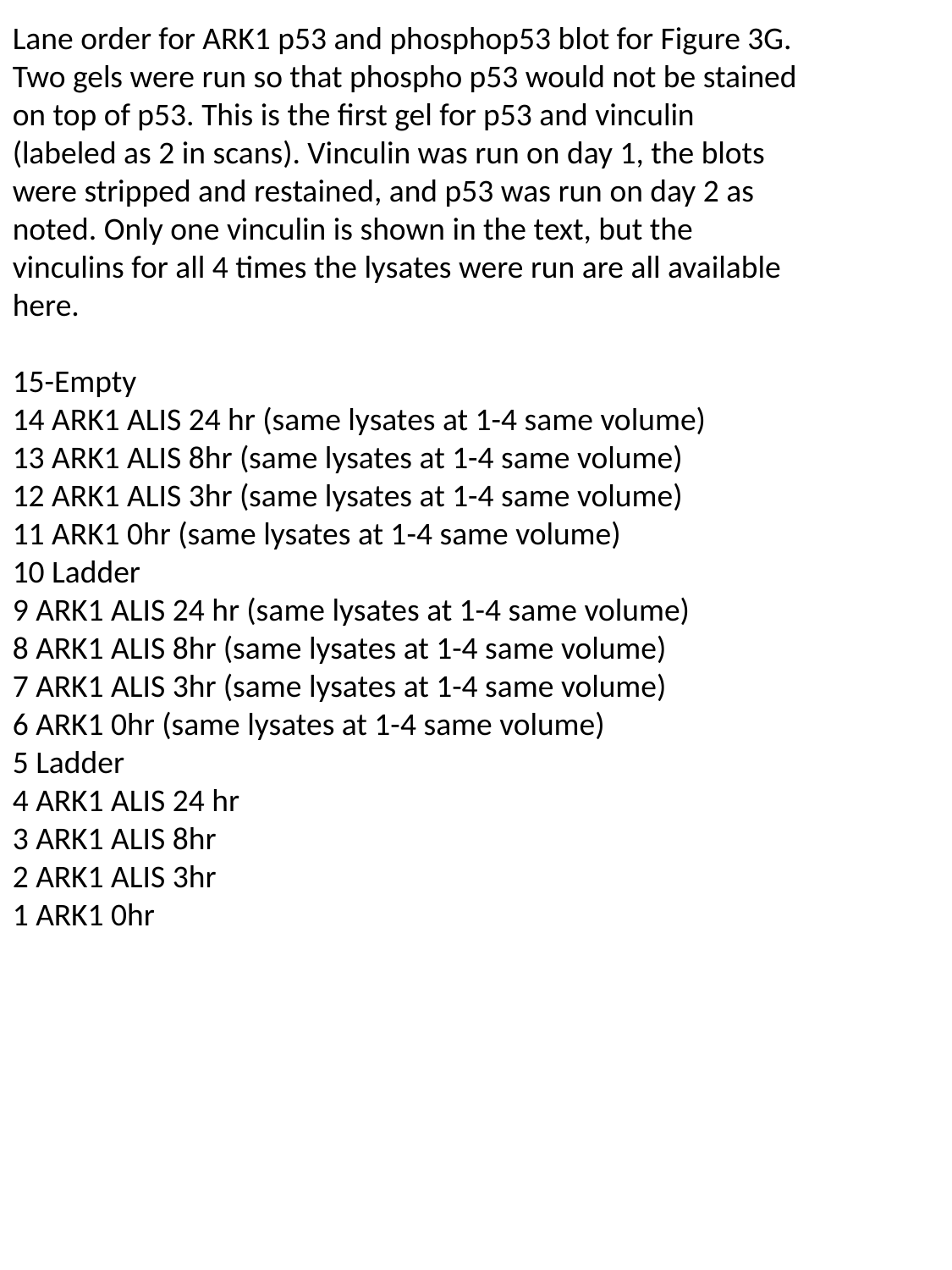

Lane order for ARK1 p53 and phosphop53 blot for Figure 3G. Two gels were run so that phospho p53 would not be stained on top of p53. This is the first gel for p53 and vinculin (labeled as 2 in scans). Vinculin was run on day 1, the blots were stripped and restained, and p53 was run on day 2 as noted. Only one vinculin is shown in the text, but the vinculins for all 4 times the lysates were run are all available here.
15-Empty
14 ARK1 ALIS 24 hr (same lysates at 1-4 same volume)
13 ARK1 ALIS 8hr (same lysates at 1-4 same volume)
12 ARK1 ALIS 3hr (same lysates at 1-4 same volume)
11 ARK1 0hr (same lysates at 1-4 same volume)
10 Ladder
9 ARK1 ALIS 24 hr (same lysates at 1-4 same volume)
8 ARK1 ALIS 8hr (same lysates at 1-4 same volume)
7 ARK1 ALIS 3hr (same lysates at 1-4 same volume)
6 ARK1 0hr (same lysates at 1-4 same volume)
5 Ladder
4 ARK1 ALIS 24 hr
3 ARK1 ALIS 8hr
2 ARK1 ALIS 3hr
1 ARK1 0hr

## Slide 6
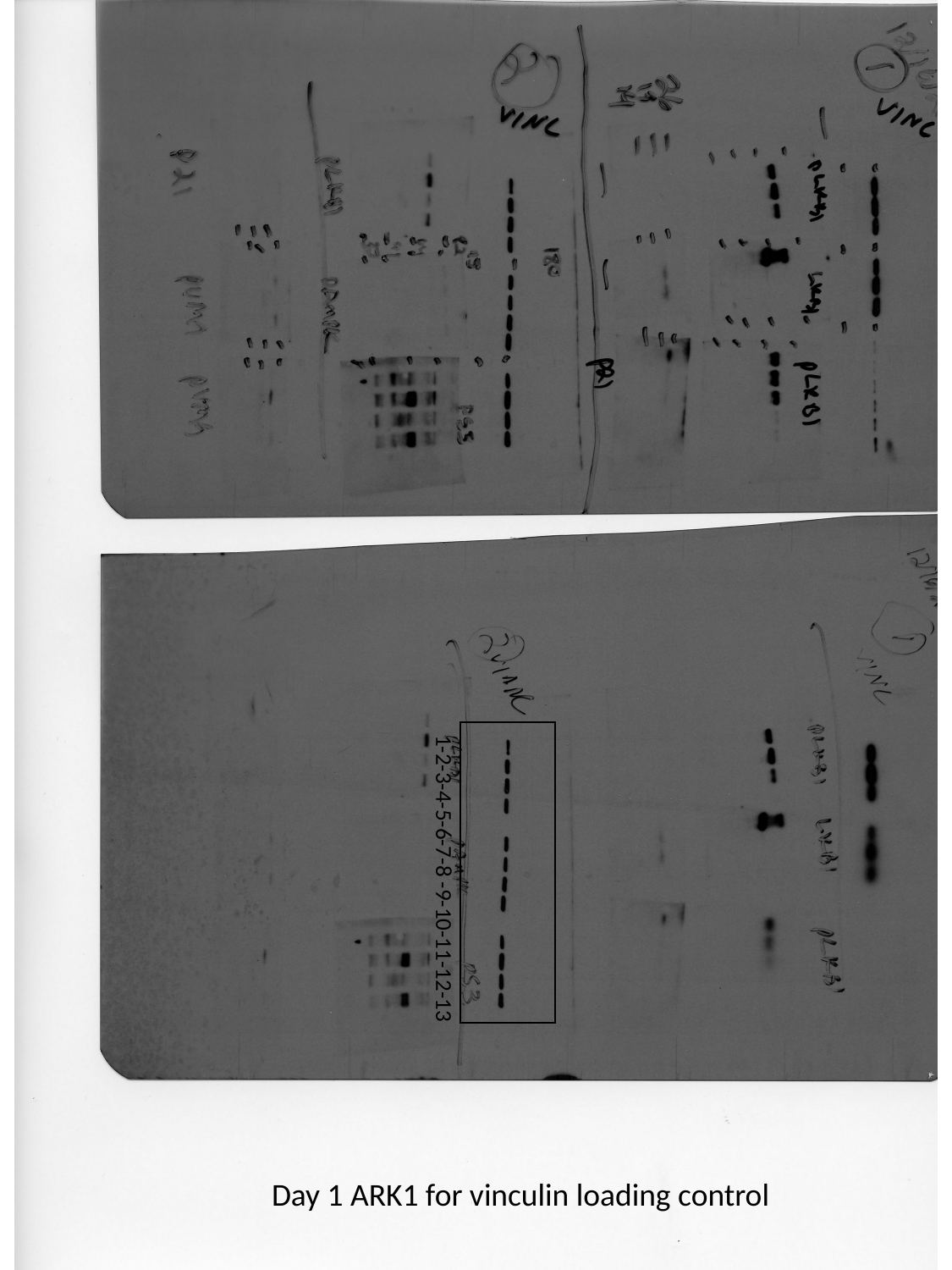

1-2-3-4-5-6-7-8 -9-10-11-12-13
Day 1 ARK1 for vinculin loading control

## Slide 7
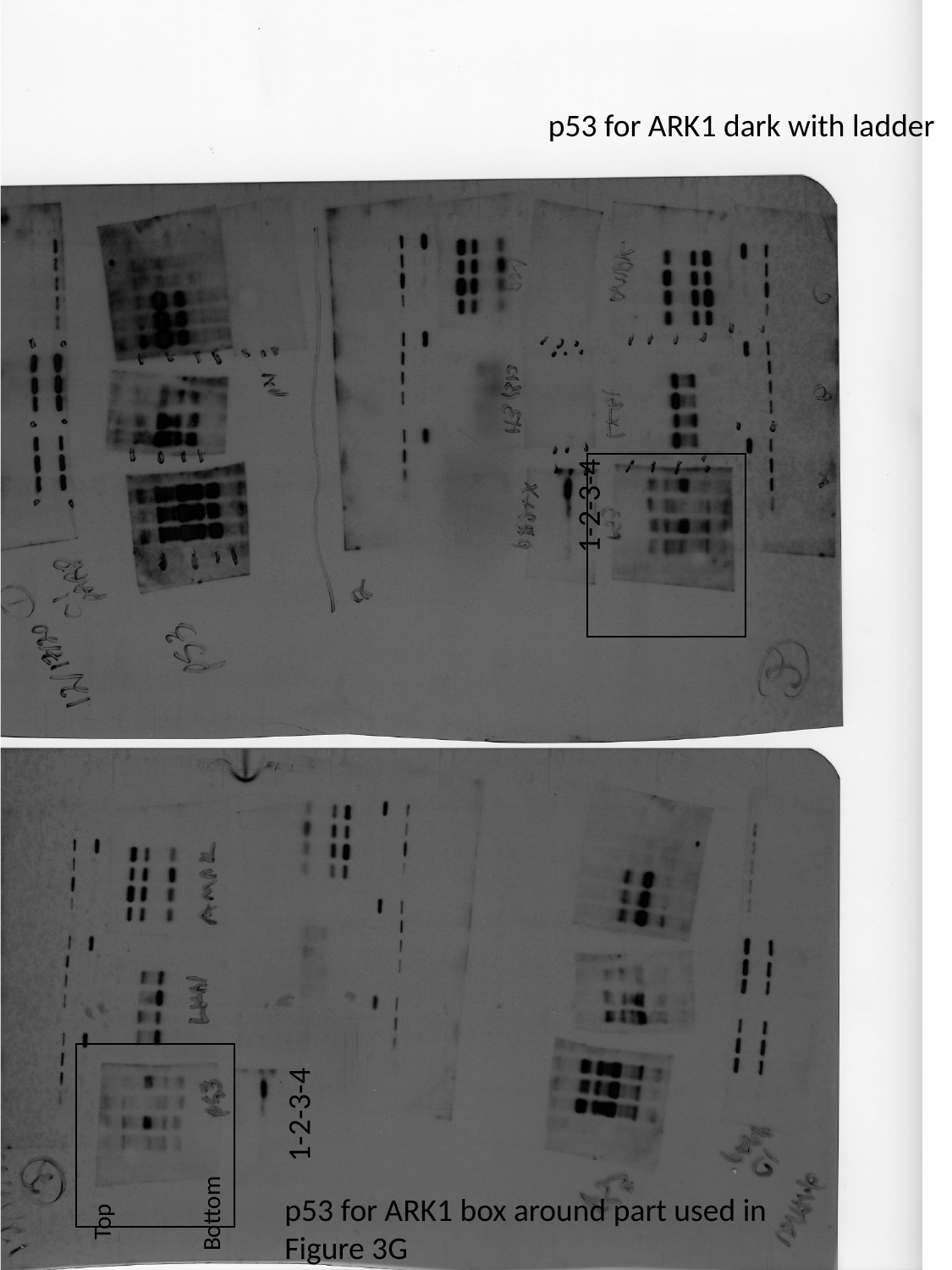

p53 for ARK1 dark with ladder
1-2-3-4
1-2-3-4
Bottom
p53 for ARK1 box around part used in Figure 3G
Top

## Slide 8
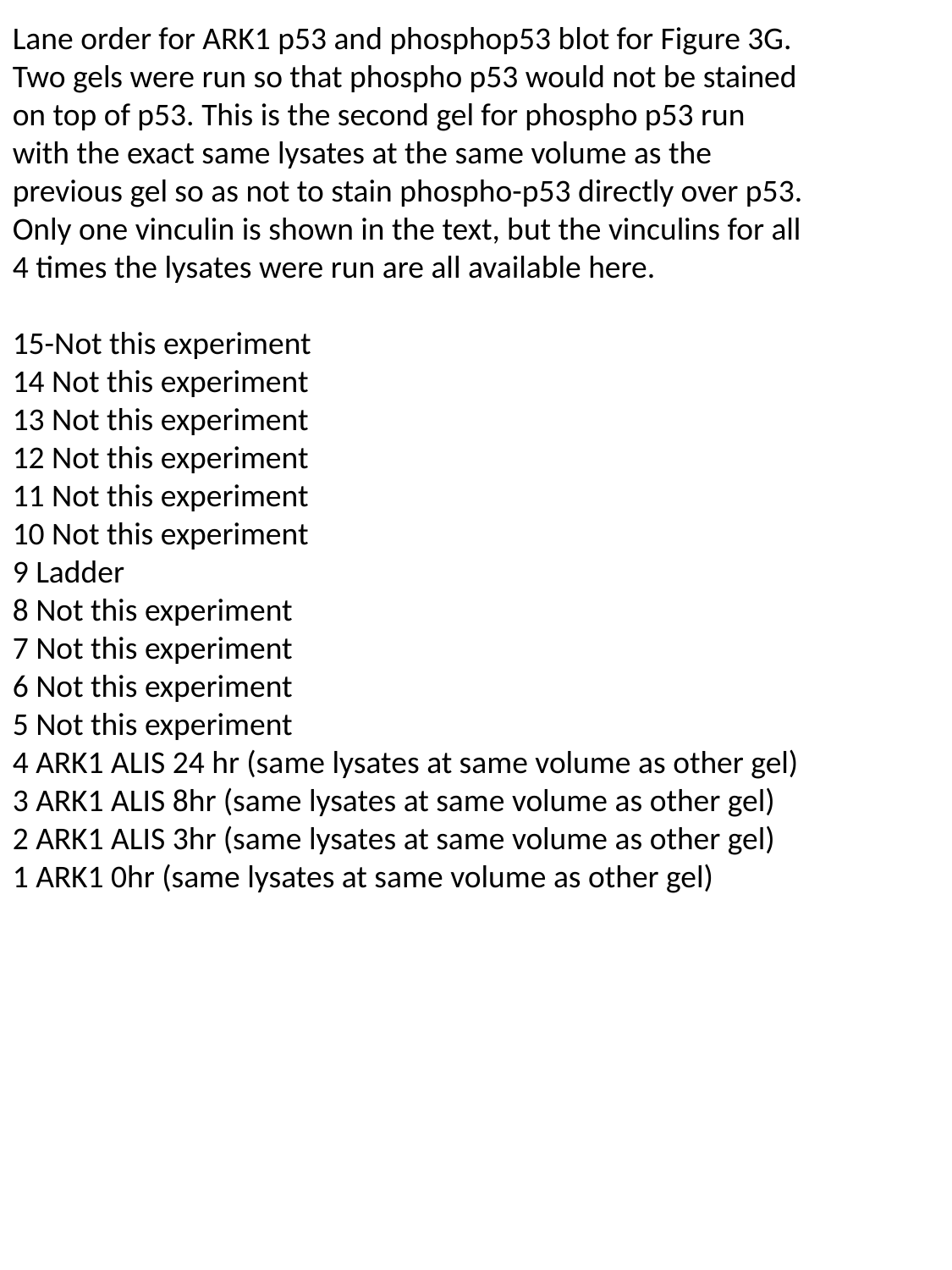

Lane order for ARK1 p53 and phosphop53 blot for Figure 3G. Two gels were run so that phospho p53 would not be stained on top of p53. This is the second gel for phospho p53 run with the exact same lysates at the same volume as the previous gel so as not to stain phospho-p53 directly over p53. Only one vinculin is shown in the text, but the vinculins for all 4 times the lysates were run are all available here.
15-Not this experiment
14 Not this experiment
13 Not this experiment
12 Not this experiment
11 Not this experiment
10 Not this experiment
9 Ladder
8 Not this experiment
7 Not this experiment
6 Not this experiment
5 Not this experiment
4 ARK1 ALIS 24 hr (same lysates at same volume as other gel)
3 ARK1 ALIS 8hr (same lysates at same volume as other gel)
2 ARK1 ALIS 3hr (same lysates at same volume as other gel)
1 ARK1 0hr (same lysates at same volume as other gel)

## Slide 9
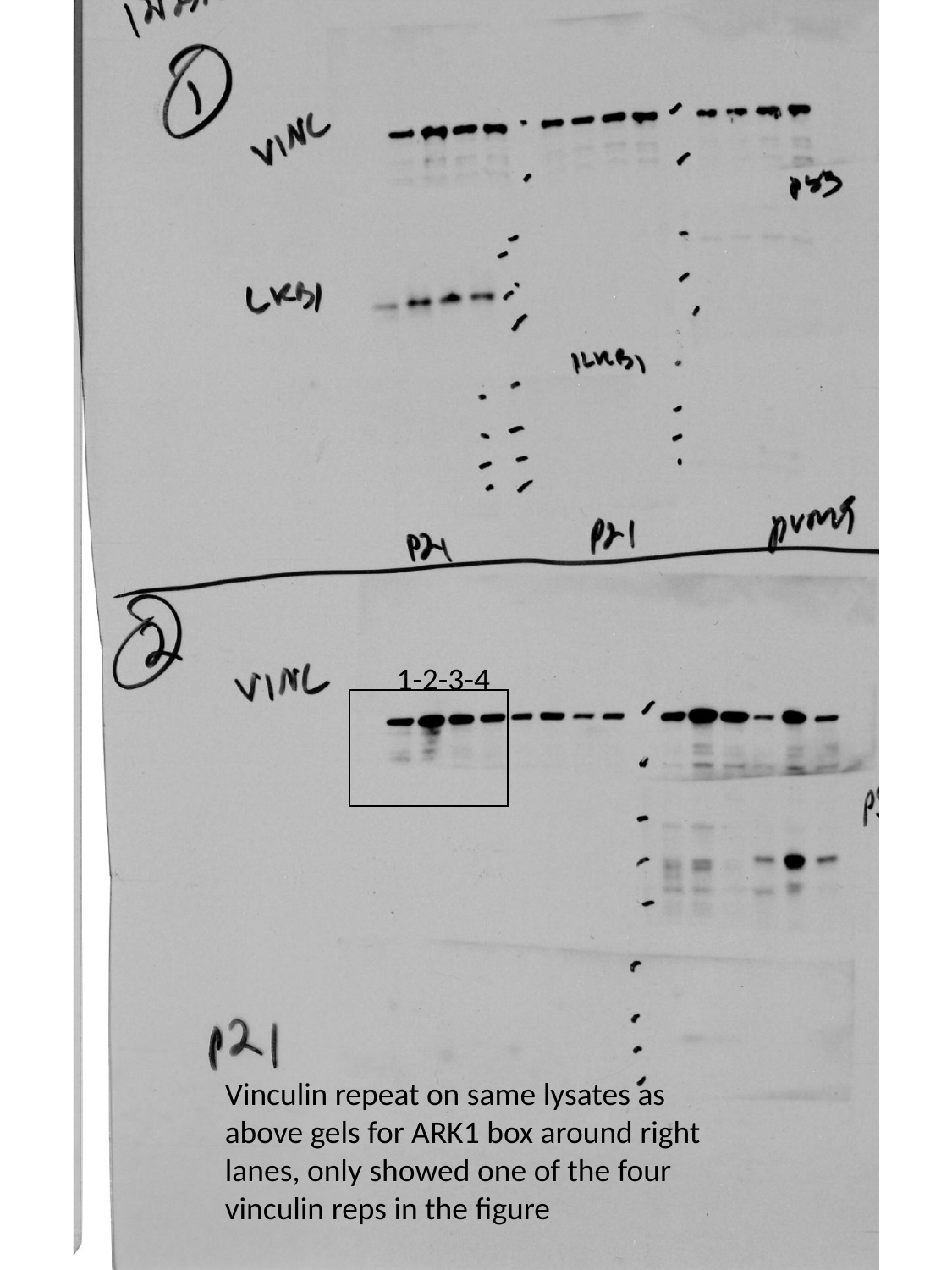

1-2-3-4
Vinculin repeat on same lysates as above gels for ARK1 box around right lanes, only showed one of the four vinculin reps in the figure

## Slide 10
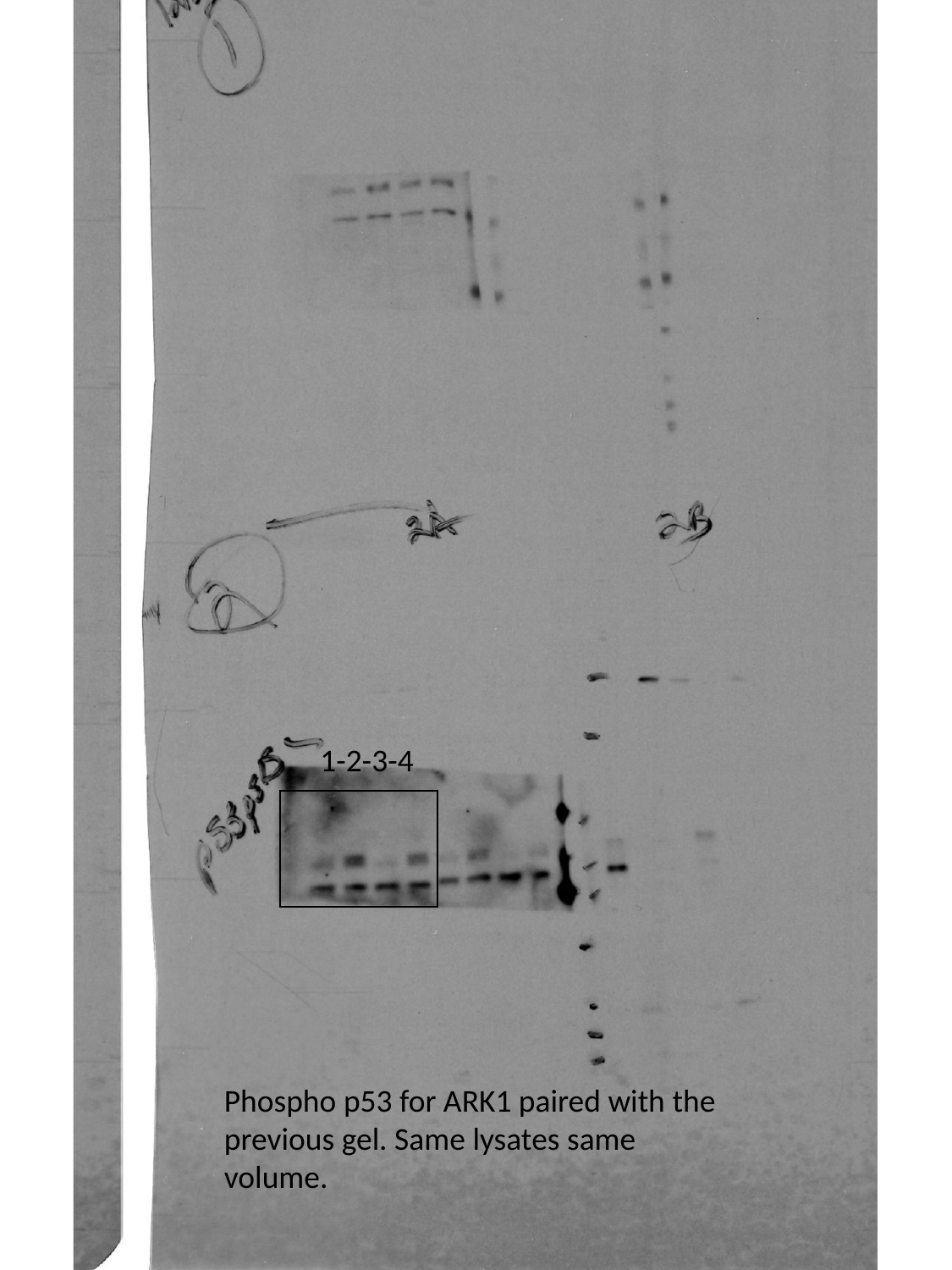

1-2-3-4
Phospho p53 for ARK1 paired with the previous gel. Same lysates same volume.
