# Supplementary material for: Enhanced Efficacy of Aurora Kinase Inhibitors in G2/M Checkpoint Deficient TP53 Mutant Uterine Carcinomas Is Linked to the Summation of LKB1–AKT–p53 Interactions
Source: Cancers (Basel). 2021 May 3;13(9):2195. doi: 10.3390/cancers13092195 (PMC8125555; doi:10.3390/cancers13092195)
Supplement: Supplementary file 1 [file cancers-13-02195-s001.zip › Lynch and Hill Supplementary Matierals/original blot/Figure 3H.pptx]

## Slide 1
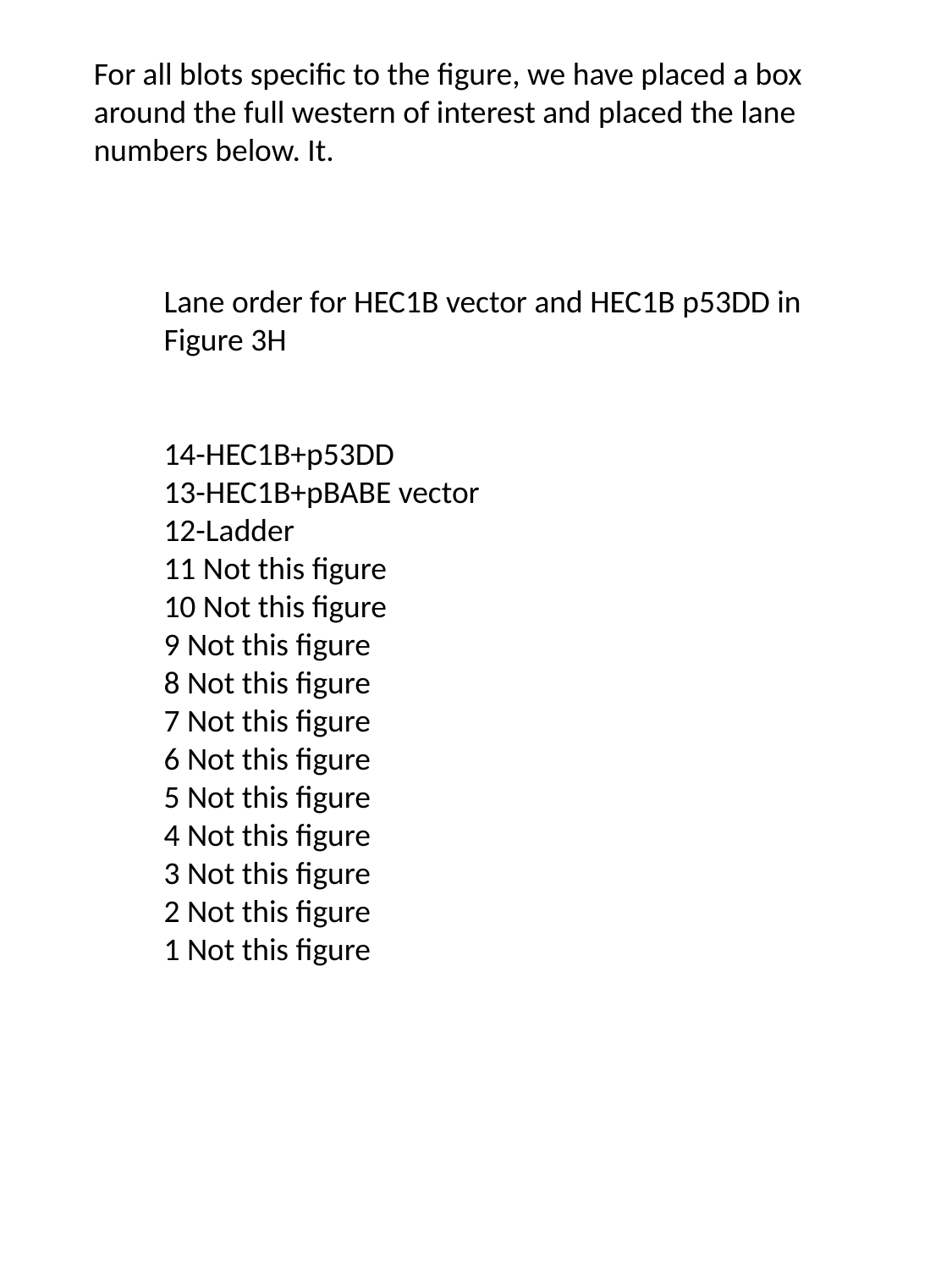

For all blots specific to the figure, we have placed a box around the full western of interest and placed the lane numbers below. It.
Lane order for HEC1B vector and HEC1B p53DD in Figure 3H
14-HEC1B+p53DD
13-HEC1B+pBABE vector
12-Ladder
11 Not this figure
10 Not this figure
9 Not this figure
8 Not this figure
7 Not this figure
6 Not this figure
5 Not this figure
4 Not this figure
3 Not this figure
2 Not this figure
1 Not this figure

## Slide 2
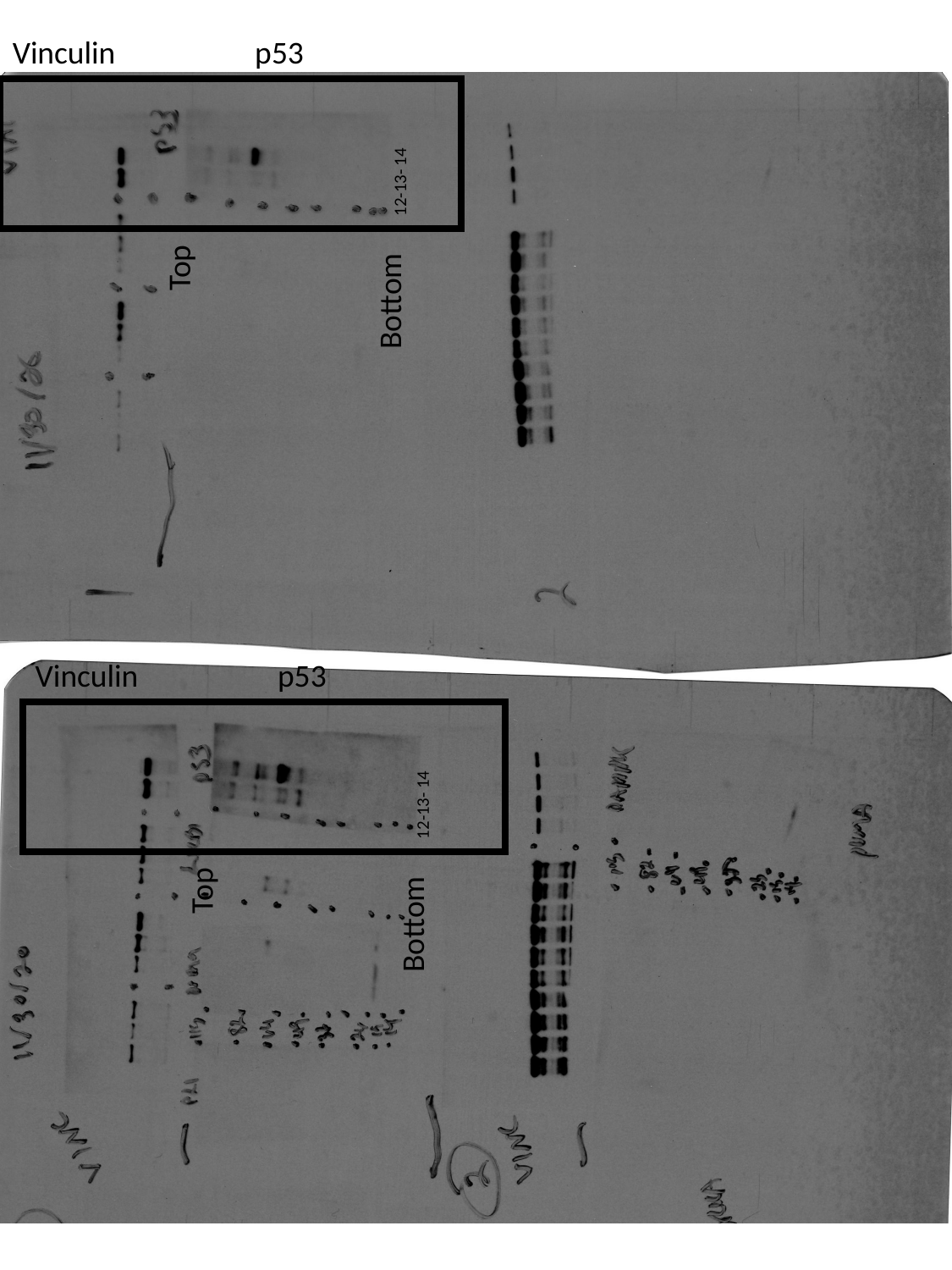

Vinculin
p53
12-13- 14
Top
Bottom
Vinculin
p53
12-13- 14
Top
Bottom
