# Supplementary material for: Enhanced Efficacy of Aurora Kinase Inhibitors in G2/M Checkpoint Deficient TP53 Mutant Uterine Carcinomas Is Linked to the Summation of LKB1–AKT–p53 Interactions
Source: Cancers (Basel). 2021 May 3;13(9):2195. doi: 10.3390/cancers13092195 (PMC8125555; doi:10.3390/cancers13092195)
Supplement: Supplementary file 1 [file cancers-13-02195-s001.zip › Lynch and Hill Supplementary Matierals/original blot/Figure 3J.pptx]

## Slide 1
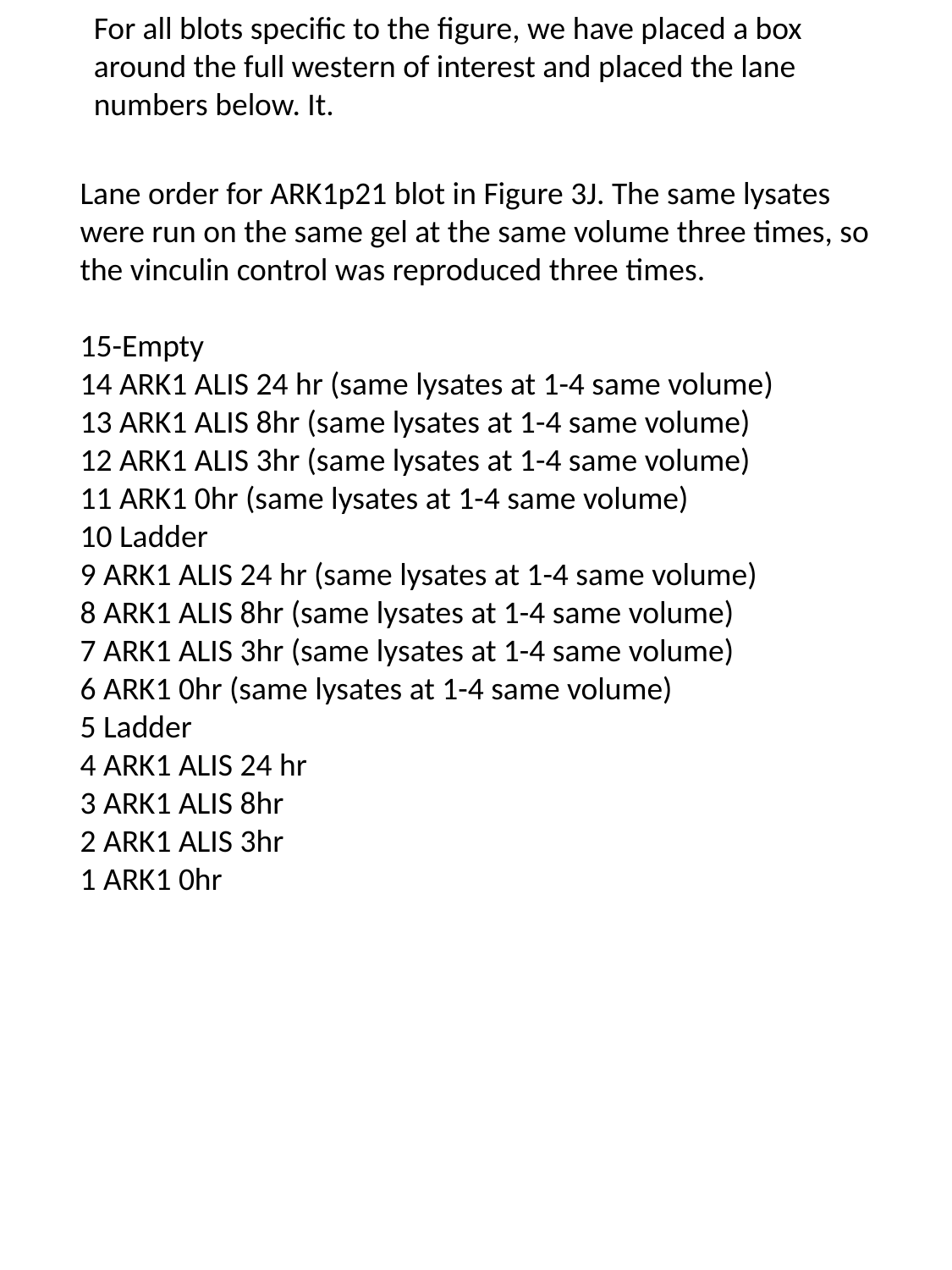

For all blots specific to the figure, we have placed a box around the full western of interest and placed the lane numbers below. It.
Lane order for ARK1p21 blot in Figure 3J. The same lysates were run on the same gel at the same volume three times, so the vinculin control was reproduced three times.
15-Empty
14 ARK1 ALIS 24 hr (same lysates at 1-4 same volume)
13 ARK1 ALIS 8hr (same lysates at 1-4 same volume)
12 ARK1 ALIS 3hr (same lysates at 1-4 same volume)
11 ARK1 0hr (same lysates at 1-4 same volume)
10 Ladder
9 ARK1 ALIS 24 hr (same lysates at 1-4 same volume)
8 ARK1 ALIS 8hr (same lysates at 1-4 same volume)
7 ARK1 ALIS 3hr (same lysates at 1-4 same volume)
6 ARK1 0hr (same lysates at 1-4 same volume)
5 Ladder
4 ARK1 ALIS 24 hr
3 ARK1 ALIS 8hr
2 ARK1 ALIS 3hr
1 ARK1 0hr

## Slide 2
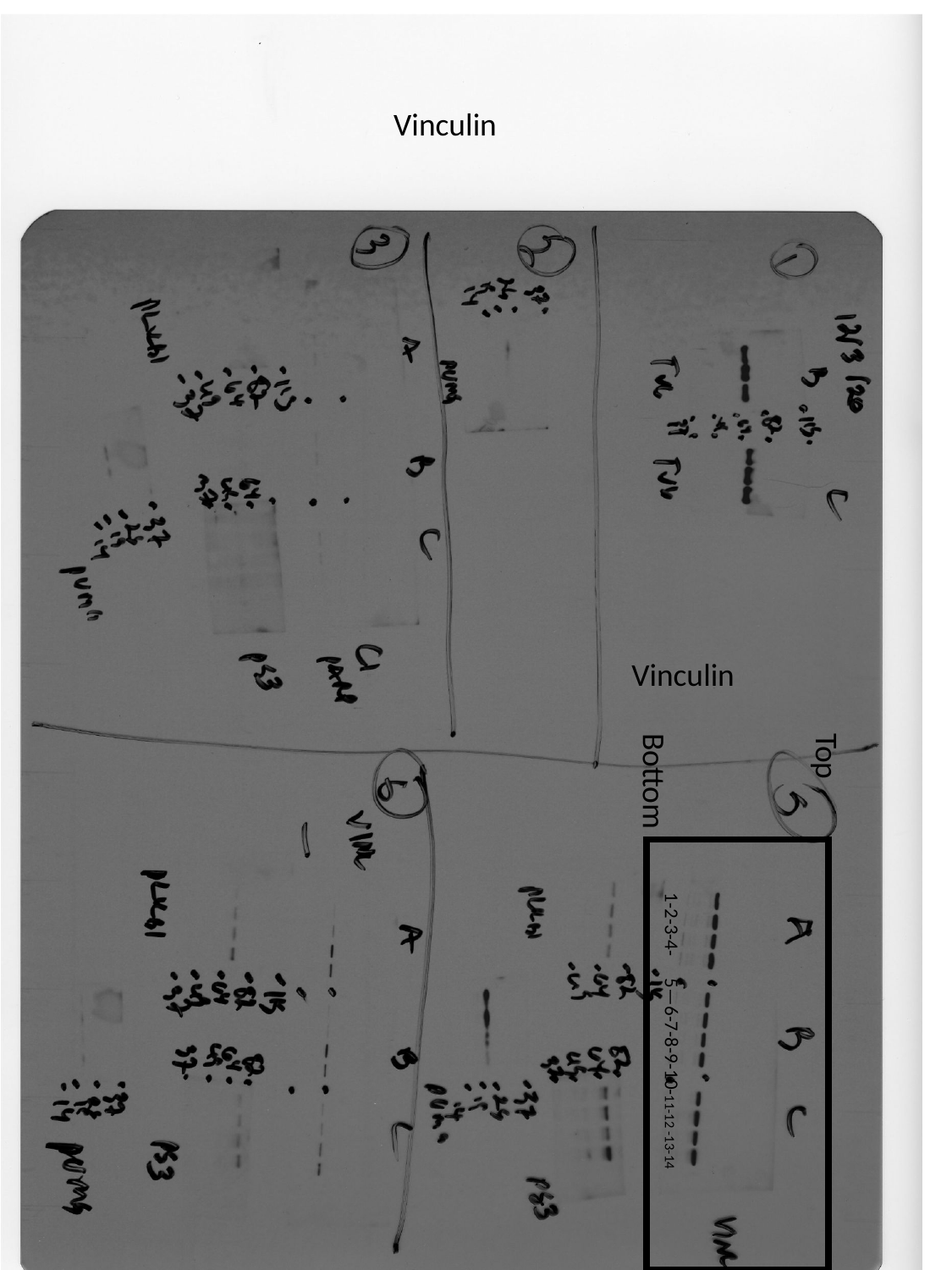

Vinculin
Vinculin
Top
Bottom
1-2-3-4- 5—6-7-8-9-10-11-12 -13-14

## Slide 3
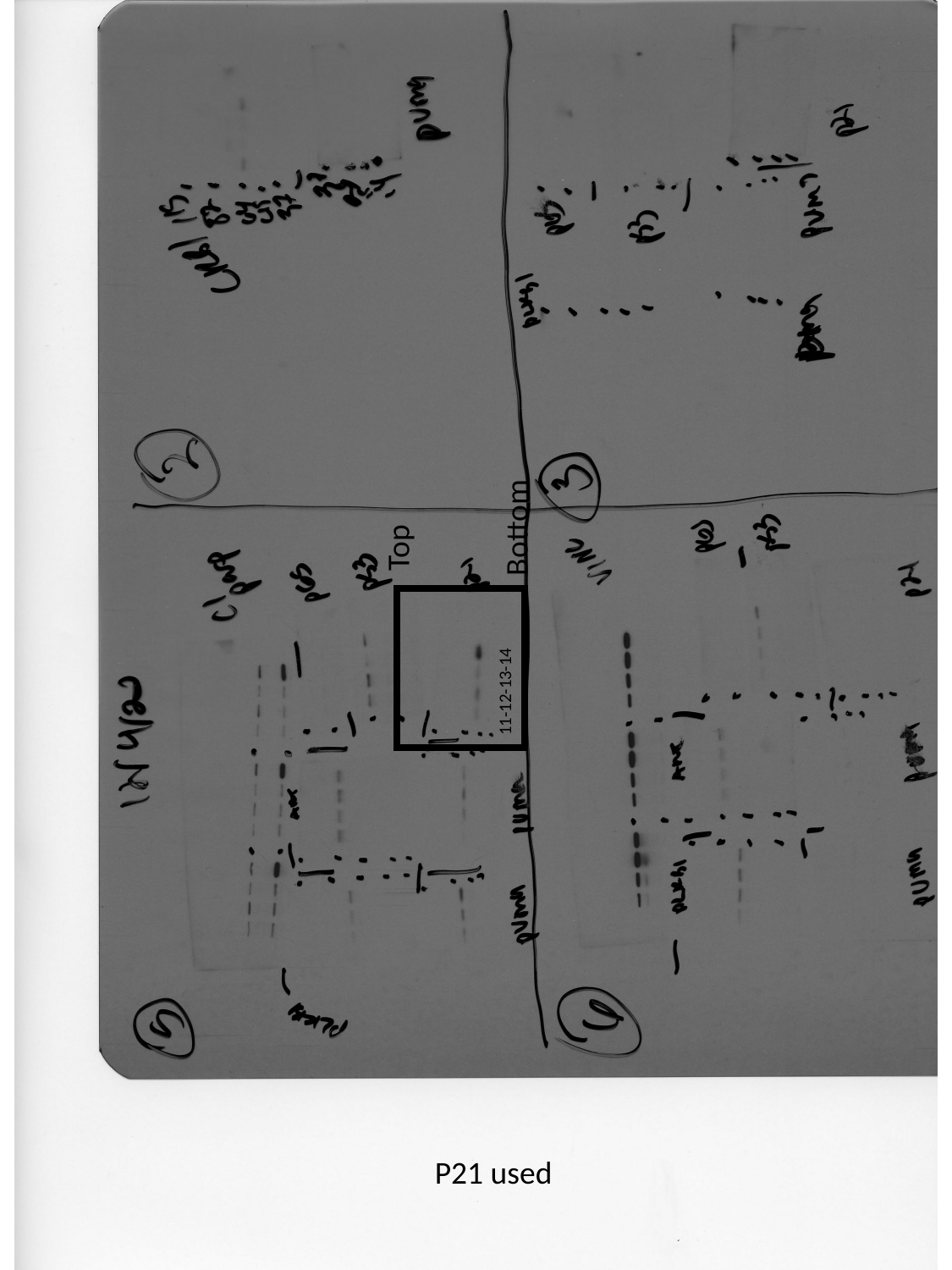

Bottom
Top
11-12-13-14
P21 used
