# Supplementary material for: Enhanced Efficacy of Aurora Kinase Inhibitors in G2/M Checkpoint Deficient TP53 Mutant Uterine Carcinomas Is Linked to the Summation of LKB1–AKT–p53 Interactions
Source: Cancers (Basel). 2021 May 3;13(9):2195. doi: 10.3390/cancers13092195 (PMC8125555; doi:10.3390/cancers13092195)
Supplement: Supplementary file 1 [file cancers-13-02195-s001.zip › Lynch and Hill Supplementary Matierals/original blot/Figure 3K.pptx]

## Slide 1
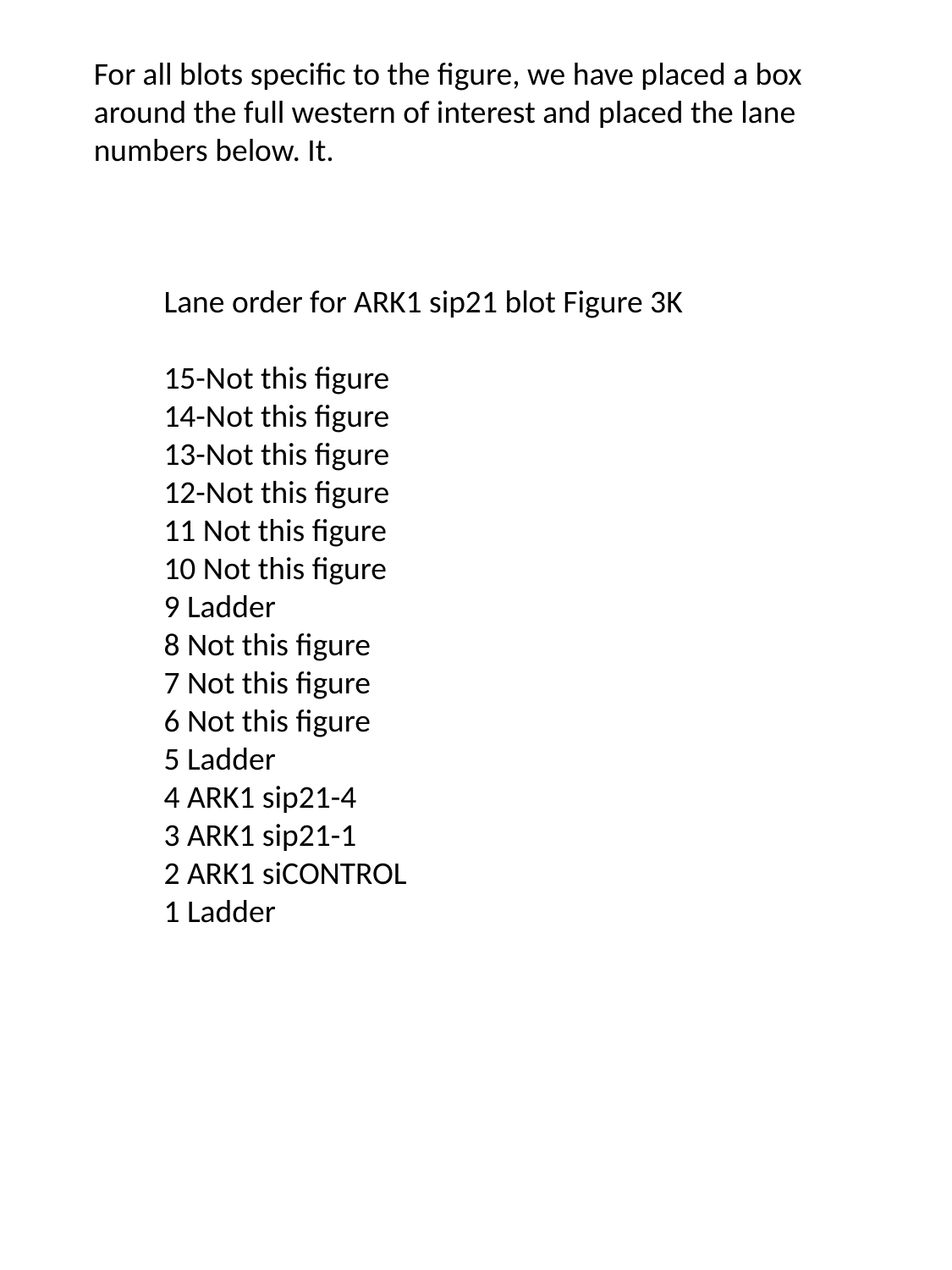

For all blots specific to the figure, we have placed a box around the full western of interest and placed the lane numbers below. It.
Lane order for ARK1 sip21 blot Figure 3K
15-Not this figure
14-Not this figure
13-Not this figure
12-Not this figure
11 Not this figure
10 Not this figure
9 Ladder
8 Not this figure
7 Not this figure
6 Not this figure
5 Ladder
4 ARK1 sip21-4
3 ARK1 sip21-1
2 ARK1 siCONTROL
1 Ladder

## Slide 2
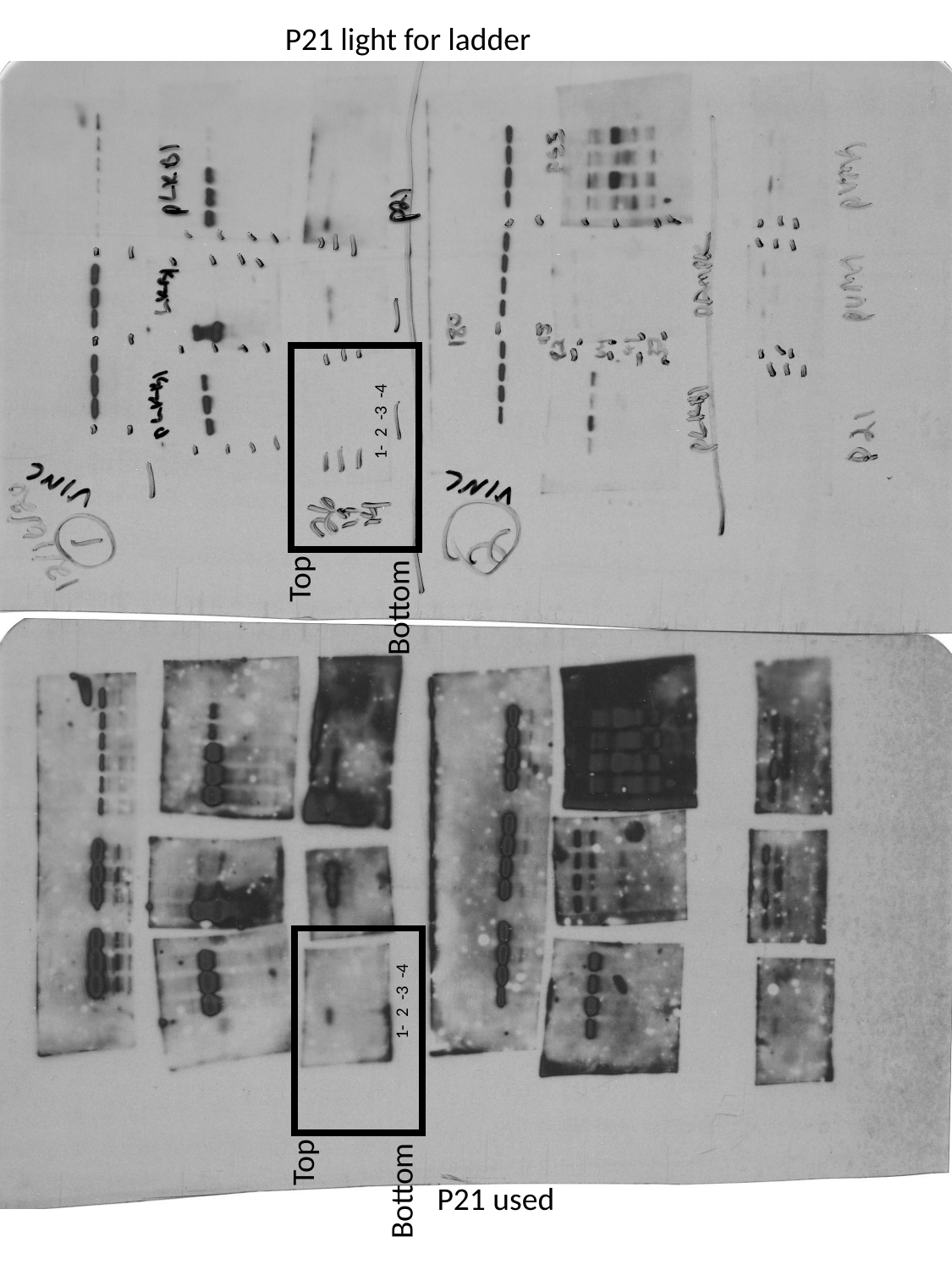

P21 light for ladder
1- 2 -3 -4
Top
Bottom
1- 2 -3 -4
Top
Bottom
P21 used

## Slide 3
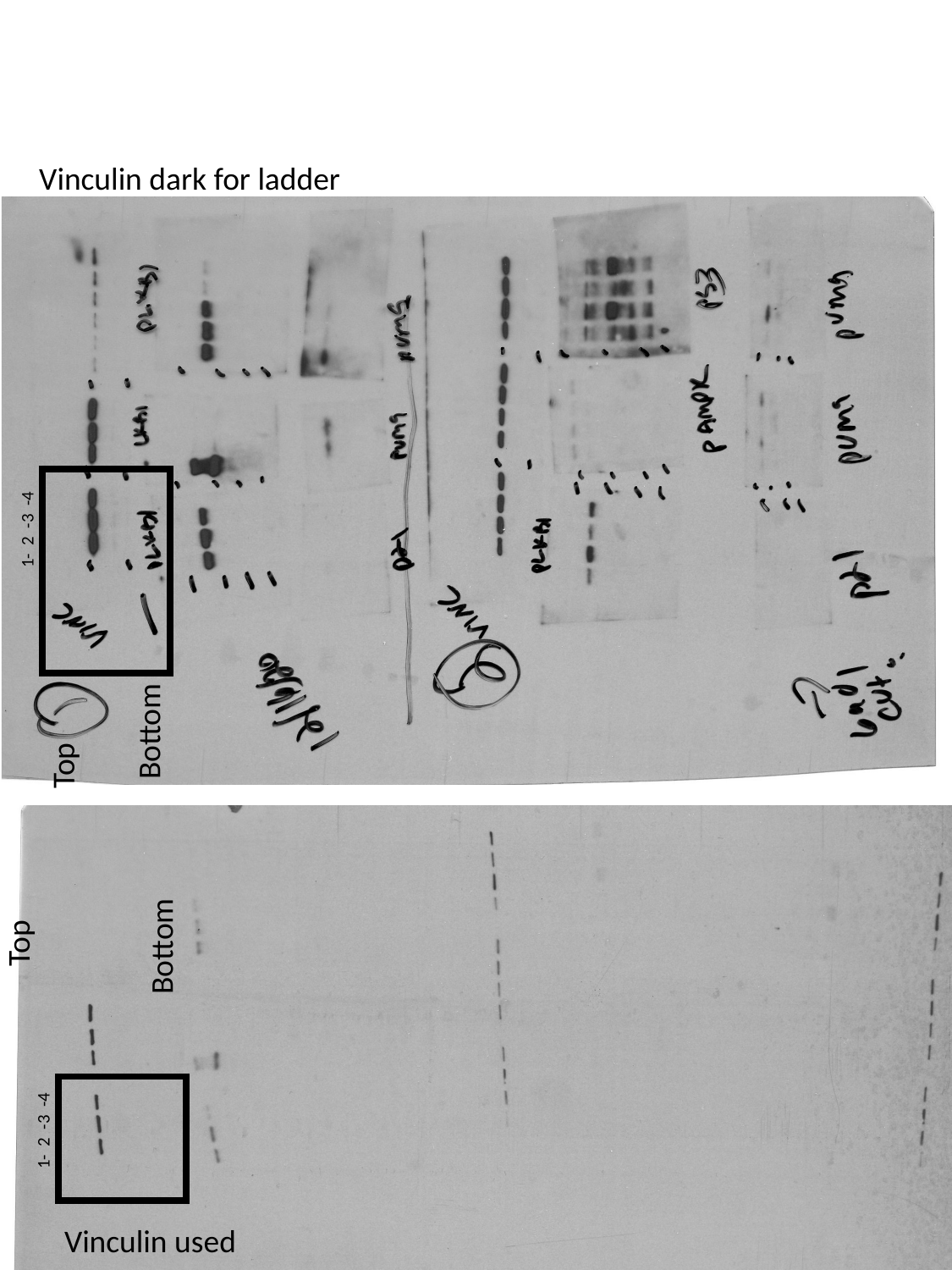

Vinculin dark for ladder
1- 2 -3 -4
Bottom
Top
Top
Bottom
1- 2 -3 -4
Vinculin used
