# Supplementary material for: Enhanced Efficacy of Aurora Kinase Inhibitors in G2/M Checkpoint Deficient TP53 Mutant Uterine Carcinomas Is Linked to the Summation of LKB1–AKT–p53 Interactions
Source: Cancers (Basel). 2021 May 3;13(9):2195. doi: 10.3390/cancers13092195 (PMC8125555; doi:10.3390/cancers13092195)
Supplement: Supplementary file 1 [file cancers-13-02195-s001.zip › Lynch and Hill Supplementary Matierals/original blot/Figure 4A.pptx]

## Slide 1
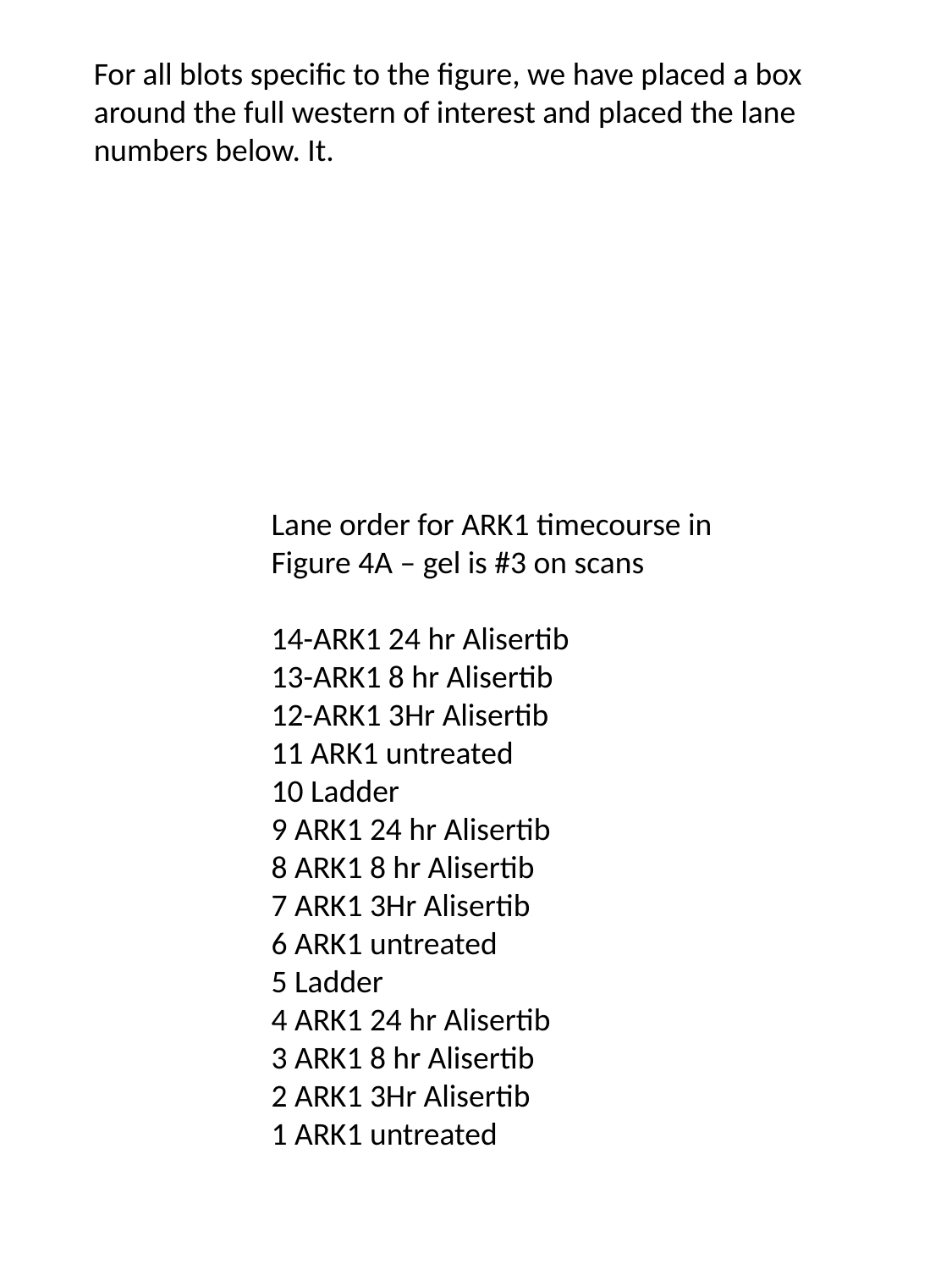

For all blots specific to the figure, we have placed a box around the full western of interest and placed the lane numbers below. It.
Lane order for ARK1 timecourse in Figure 4A – gel is #3 on scans
14-ARK1 24 hr Alisertib
13-ARK1 8 hr Alisertib
12-ARK1 3Hr Alisertib
11 ARK1 untreated
10 Ladder
9 ARK1 24 hr Alisertib
8 ARK1 8 hr Alisertib
7 ARK1 3Hr Alisertib
6 ARK1 untreated
5 Ladder
4 ARK1 24 hr Alisertib
3 ARK1 8 hr Alisertib
2 ARK1 3Hr Alisertib
1 ARK1 untreated

## Slide 2
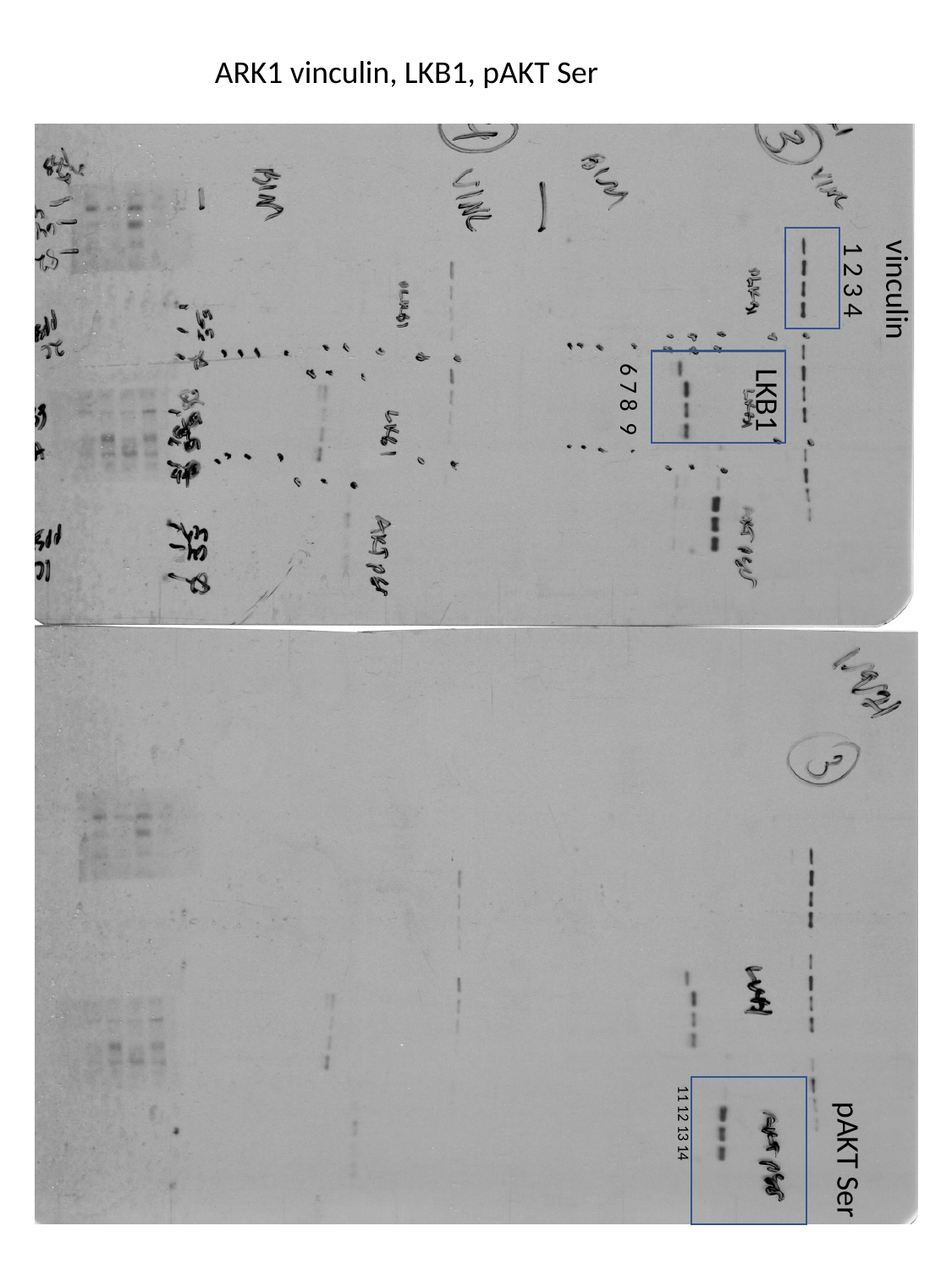

ARK1 vinculin, LKB1, pAKT Ser
1 2 3 4
vinculin
6 7 8 9
LKB1
11 12 13 14
pAKT Ser

## Slide 3
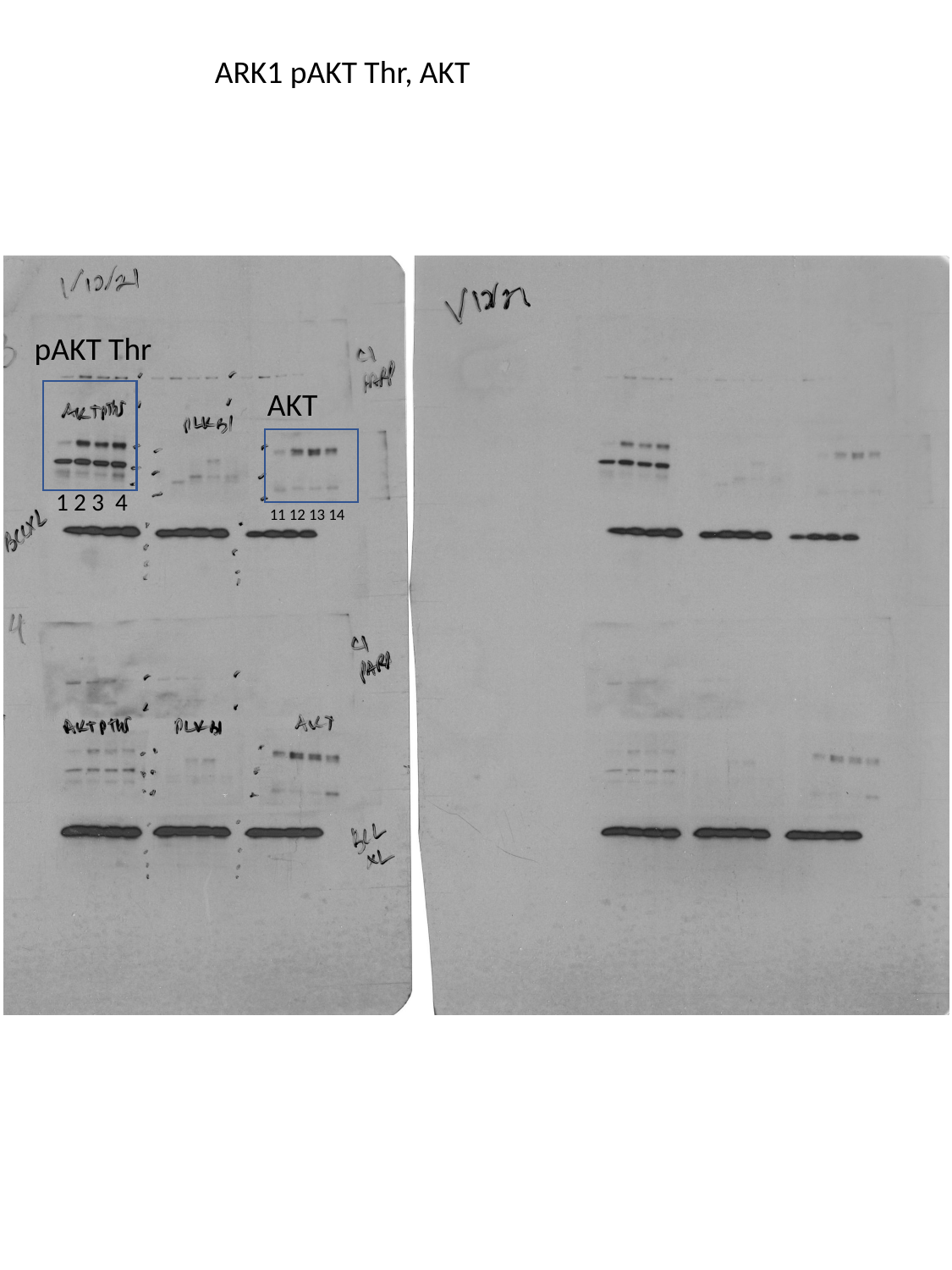

ARK1 pAKT Thr, AKT
pAKT Thr
AKT
1 2 3 4
11 12 13 14

## Slide 4
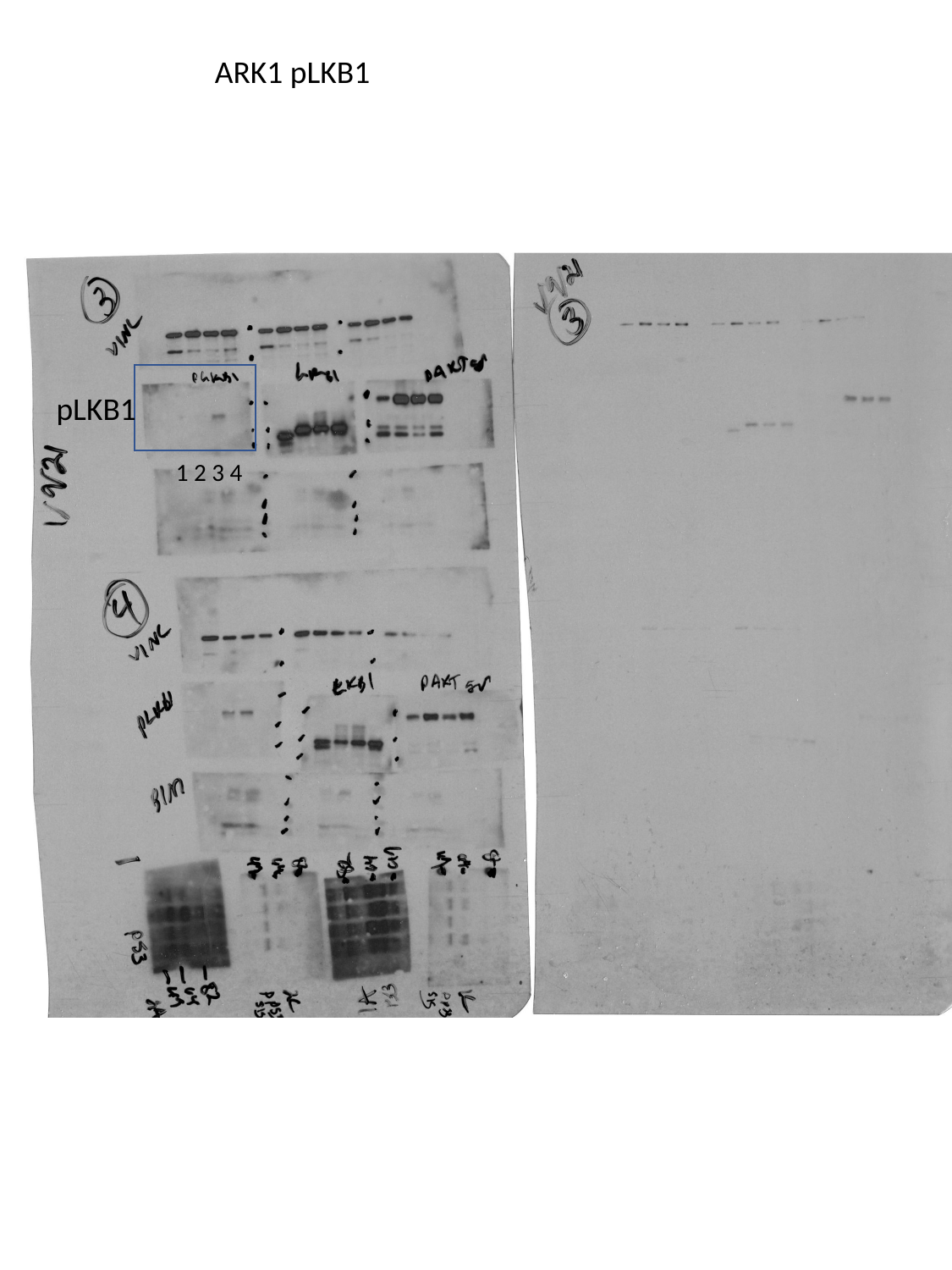

ARK1 pLKB1
pLKB1
1 2 3 4

## Slide 5
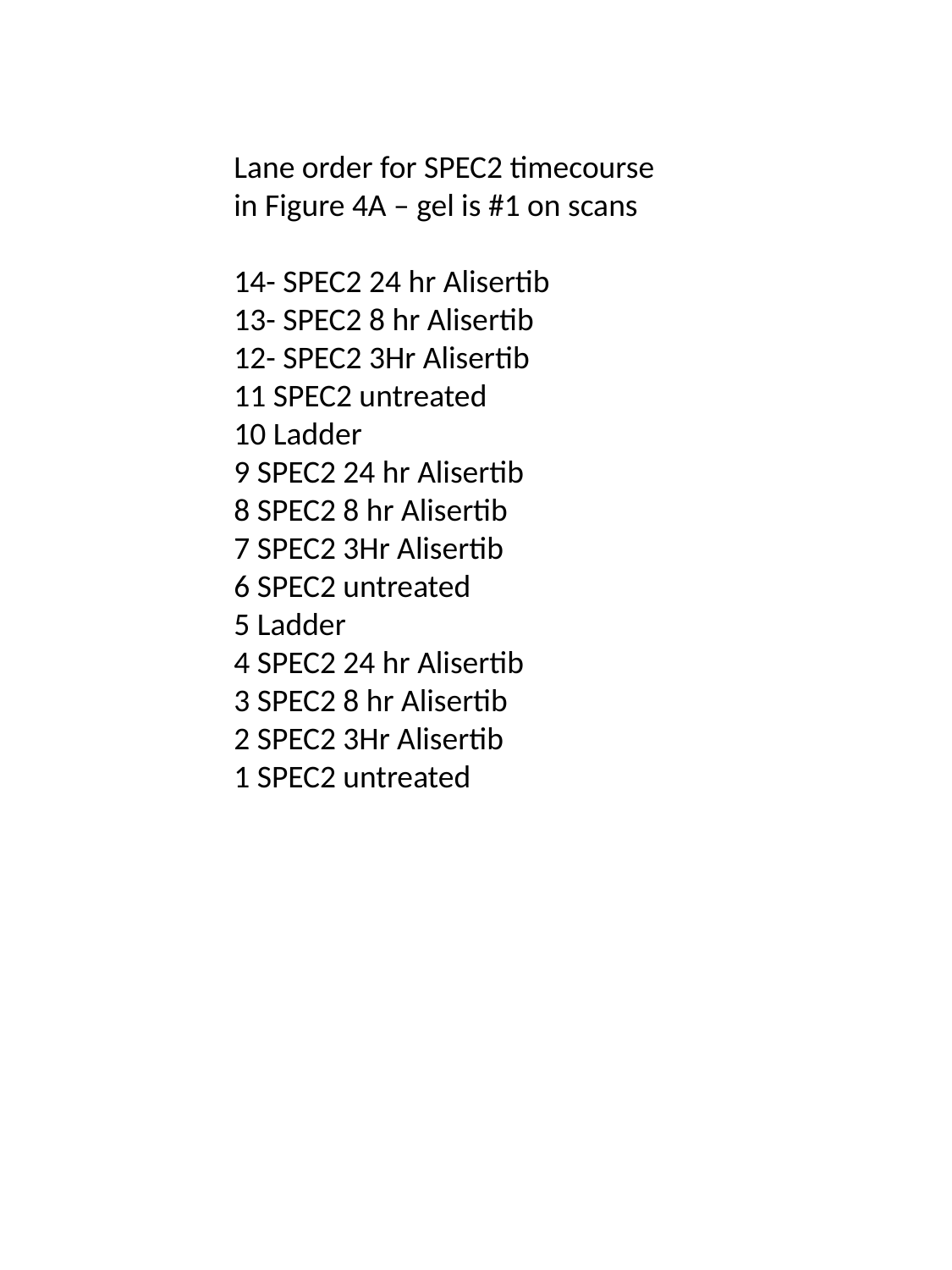

Lane order for SPEC2 timecourse in Figure 4A – gel is #1 on scans
14- SPEC2 24 hr Alisertib
13- SPEC2 8 hr Alisertib
12- SPEC2 3Hr Alisertib
11 SPEC2 untreated
10 Ladder
9 SPEC2 24 hr Alisertib
8 SPEC2 8 hr Alisertib
7 SPEC2 3Hr Alisertib
6 SPEC2 untreated
5 Ladder
4 SPEC2 24 hr Alisertib
3 SPEC2 8 hr Alisertib
2 SPEC2 3Hr Alisertib
1 SPEC2 untreated

## Slide 6
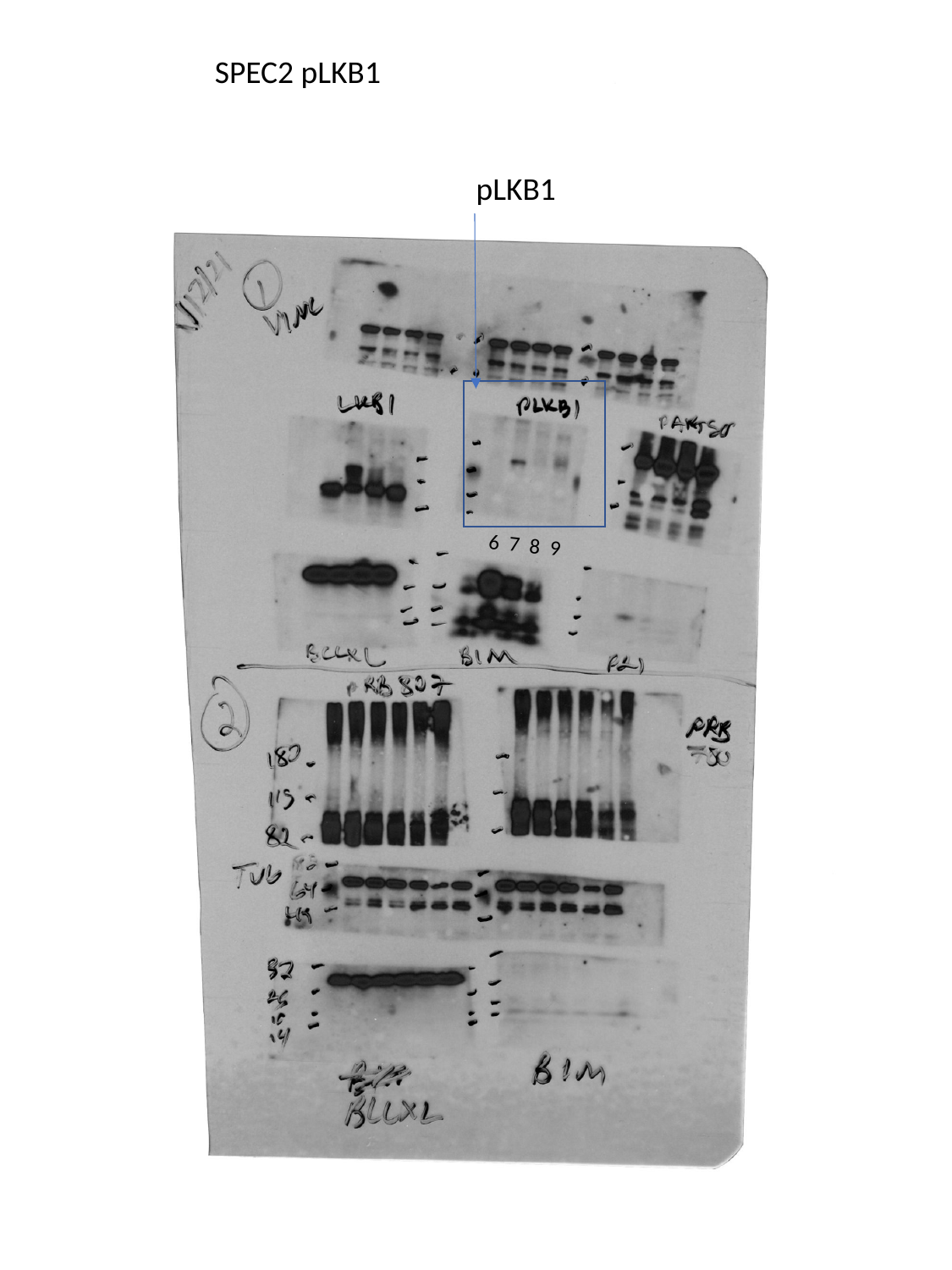

SPEC2 pLKB1
pLKB1
6 7 8 9

## Slide 7
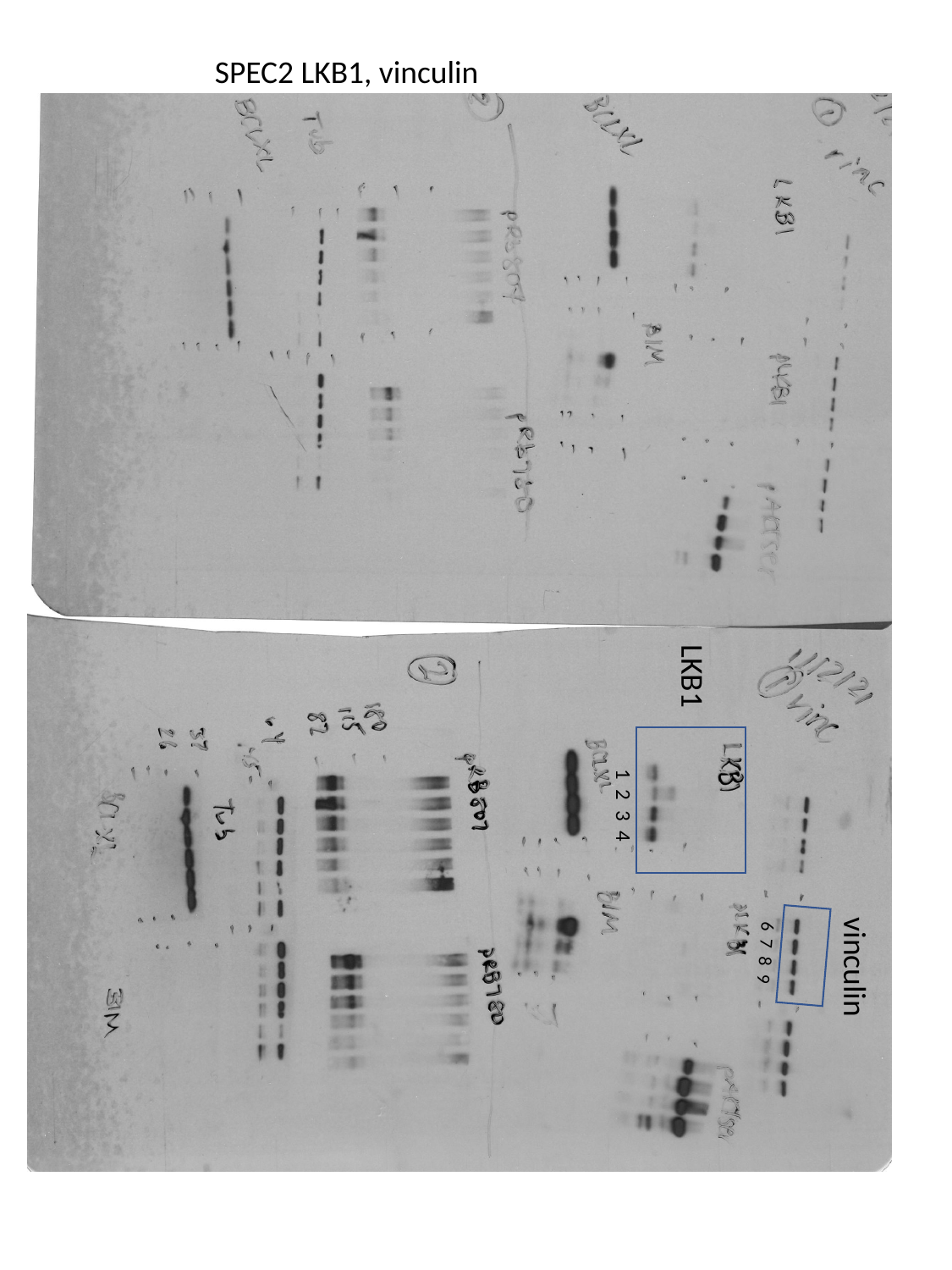

SPEC2 LKB1, vinculin
LKB1
1 2 3 4
6 7 8 9
vinculin

## Slide 8
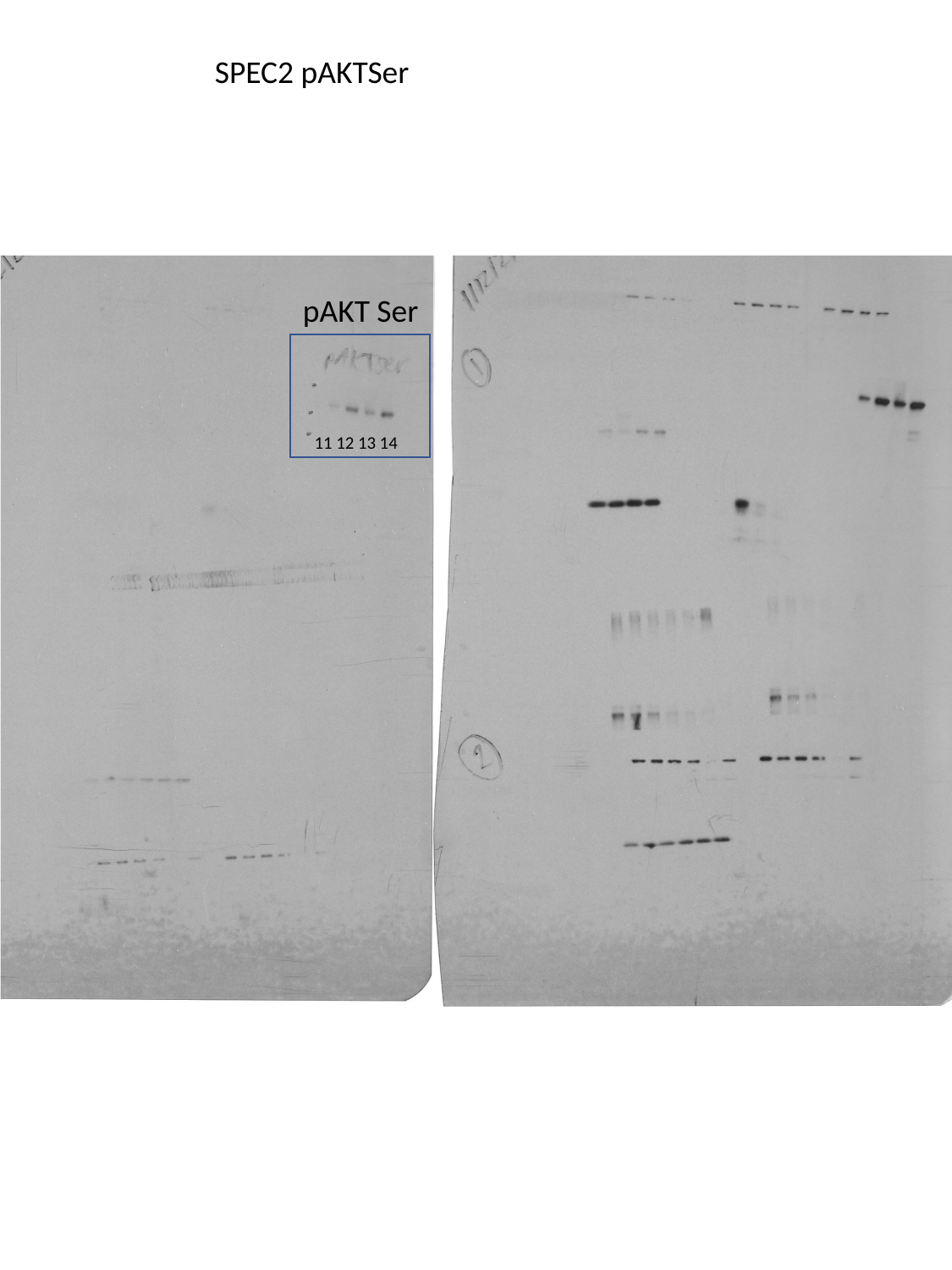

SPEC2 pAKTSer
pAKT Ser
11 12 13 14

## Slide 9
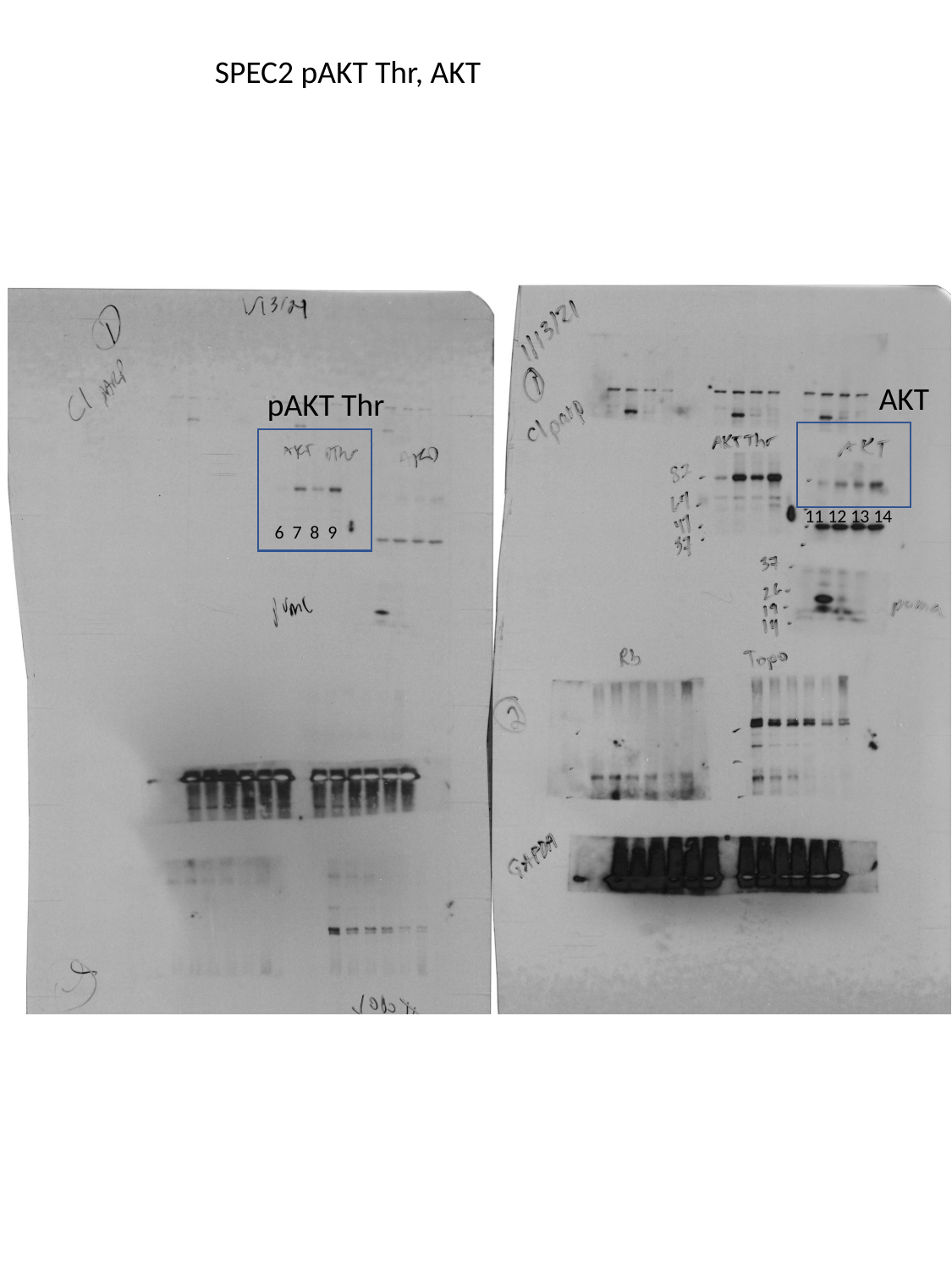

SPEC2 pAKT Thr, AKT
AKT
pAKT Thr
11 12 13 14
6 7 8 9

## Slide 10
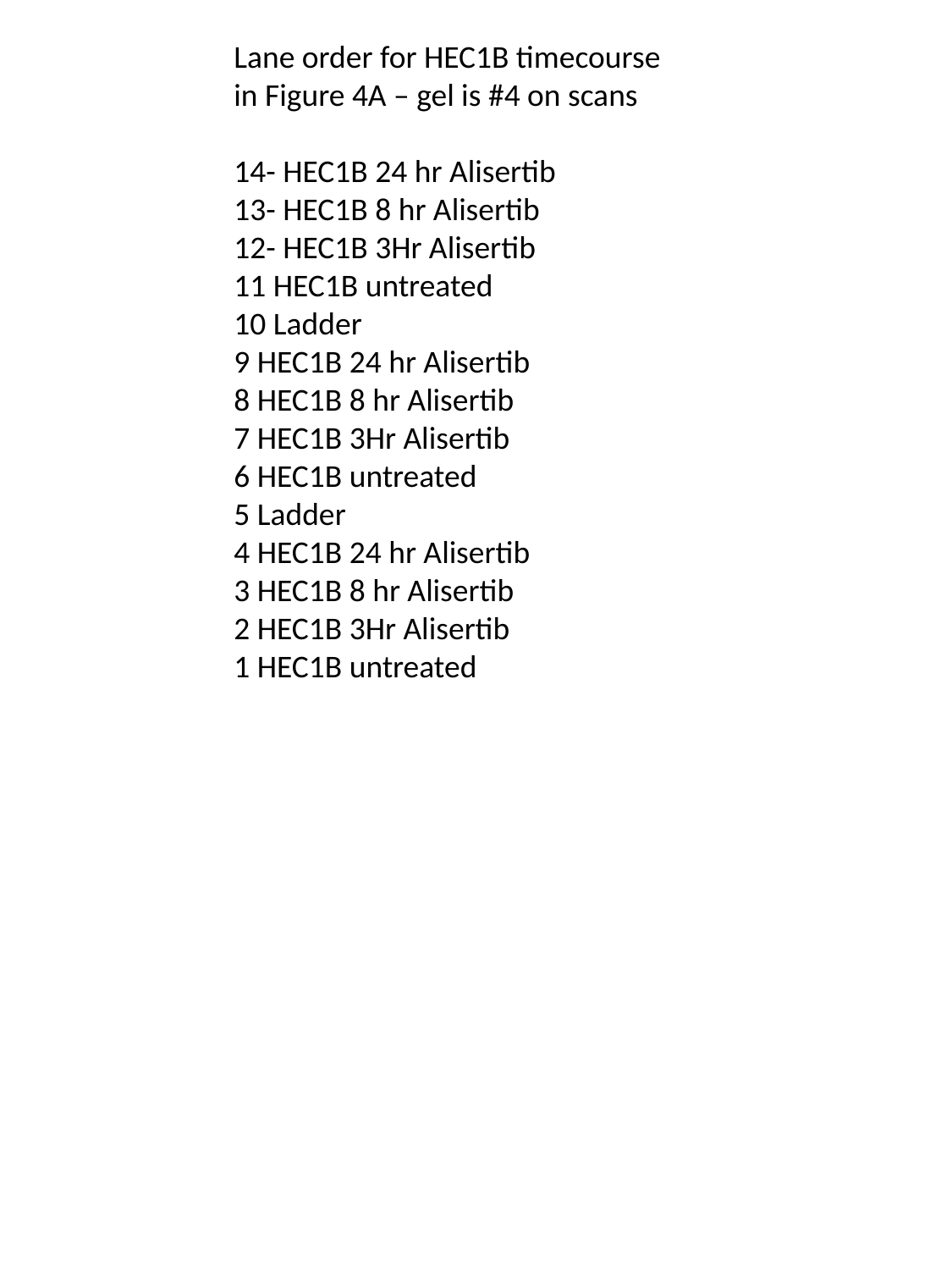

Lane order for HEC1B timecourse in Figure 4A – gel is #4 on scans
14- HEC1B 24 hr Alisertib
13- HEC1B 8 hr Alisertib
12- HEC1B 3Hr Alisertib
11 HEC1B untreated
10 Ladder
9 HEC1B 24 hr Alisertib
8 HEC1B 8 hr Alisertib
7 HEC1B 3Hr Alisertib
6 HEC1B untreated
5 Ladder
4 HEC1B 24 hr Alisertib
3 HEC1B 8 hr Alisertib
2 HEC1B 3Hr Alisertib
1 HEC1B untreated

## Slide 11
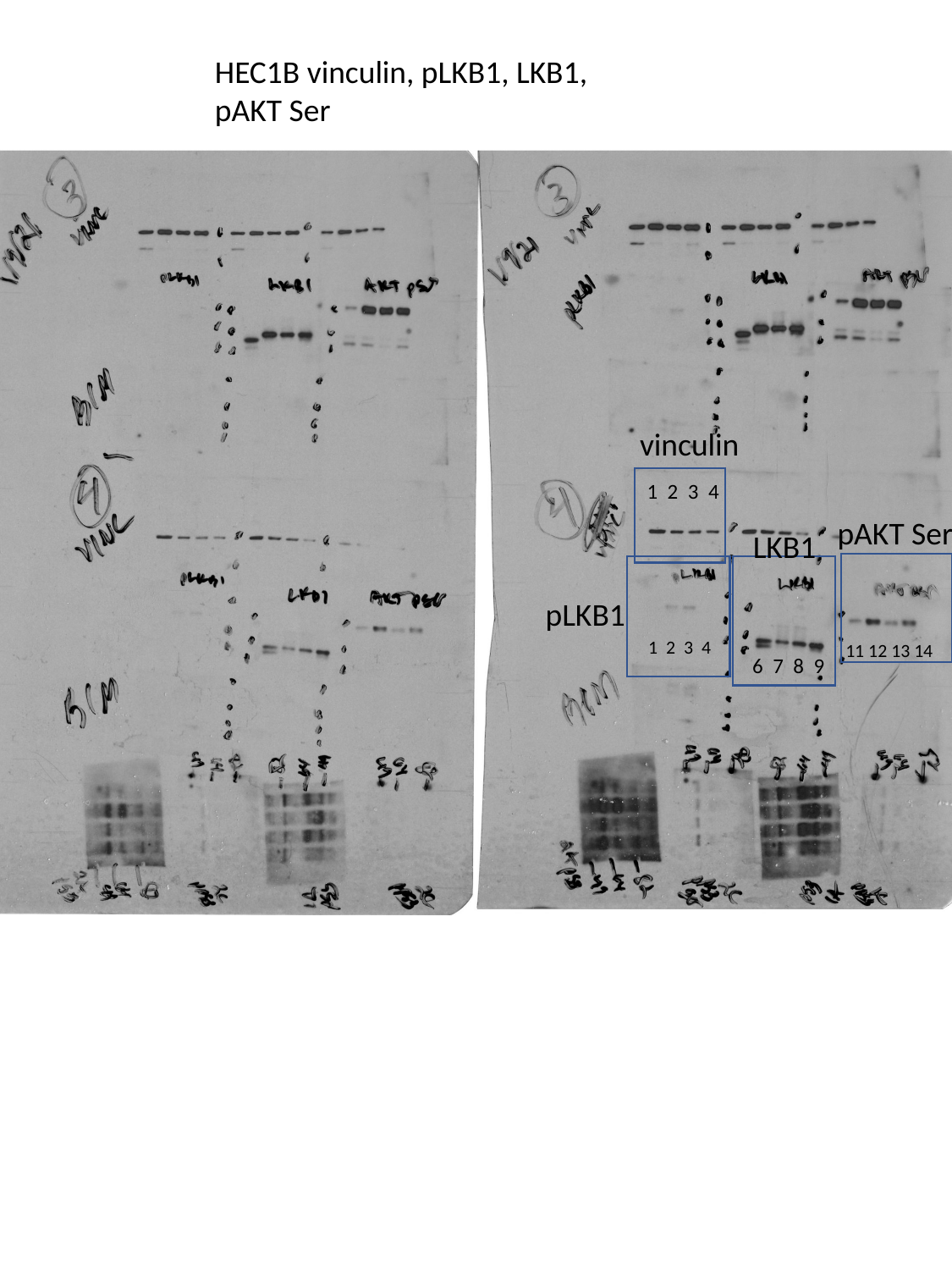

HEC1B vinculin, pLKB1, LKB1, pAKT Ser
vinculin
1 2 3 4
pAKT Ser
LKB1
pLKB1
1 2 3 4
11 12 13 14
6 7 8 9

## Slide 12
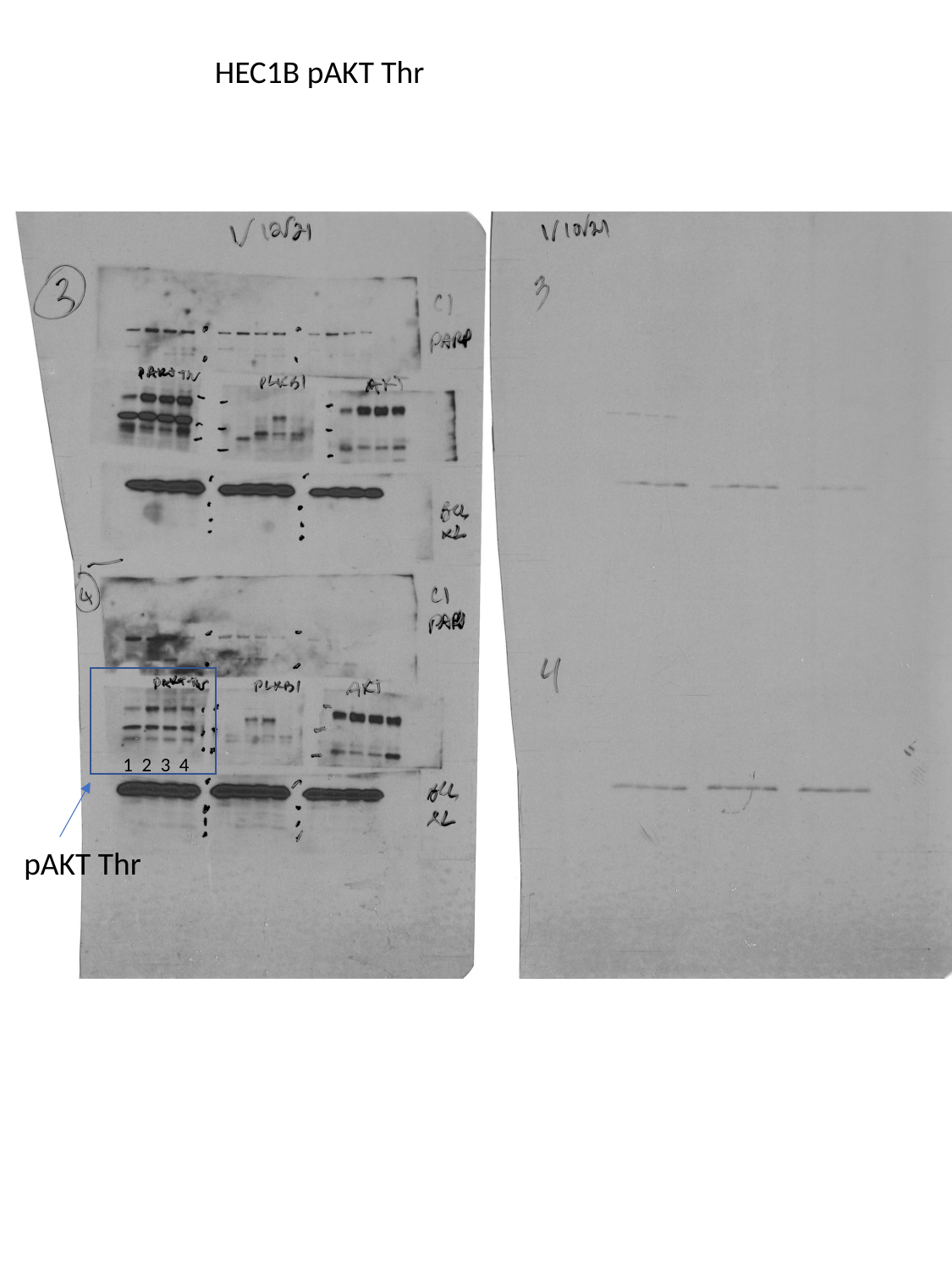

HEC1B pAKT Thr
1 2 3 4
pAKT Thr

## Slide 13
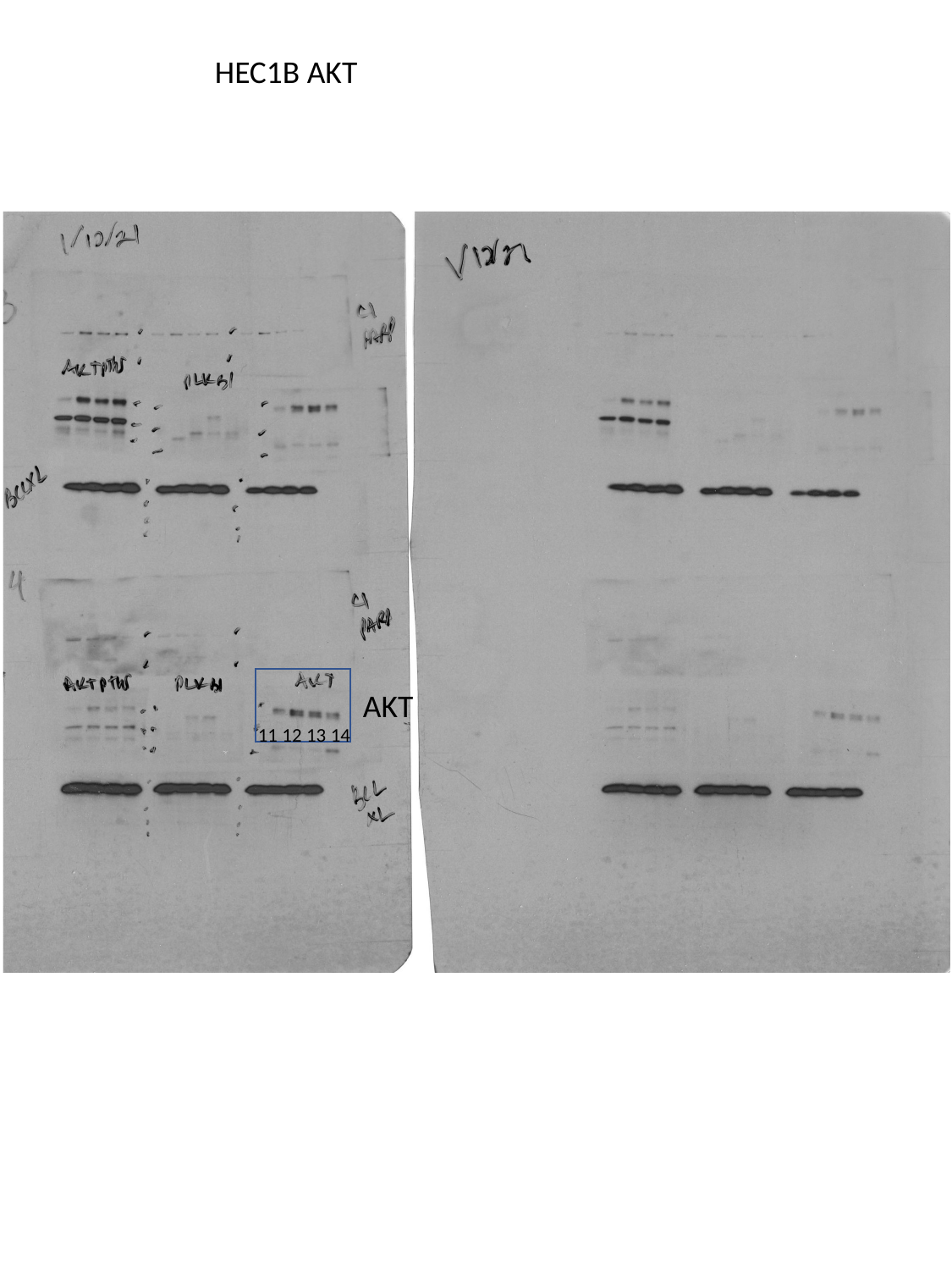

HEC1B AKT
AKT
11 12 13 14
