# Supplementary material for: Enhanced Efficacy of Aurora Kinase Inhibitors in G2/M Checkpoint Deficient TP53 Mutant Uterine Carcinomas Is Linked to the Summation of LKB1–AKT–p53 Interactions
Source: Cancers (Basel). 2021 May 3;13(9):2195. doi: 10.3390/cancers13092195 (PMC8125555; doi:10.3390/cancers13092195)
Supplement: Supplementary file 1 [file cancers-13-02195-s001.zip › Lynch and Hill Supplementary Matierals/original blot/Figure 5F.pptx]

## Slide 1
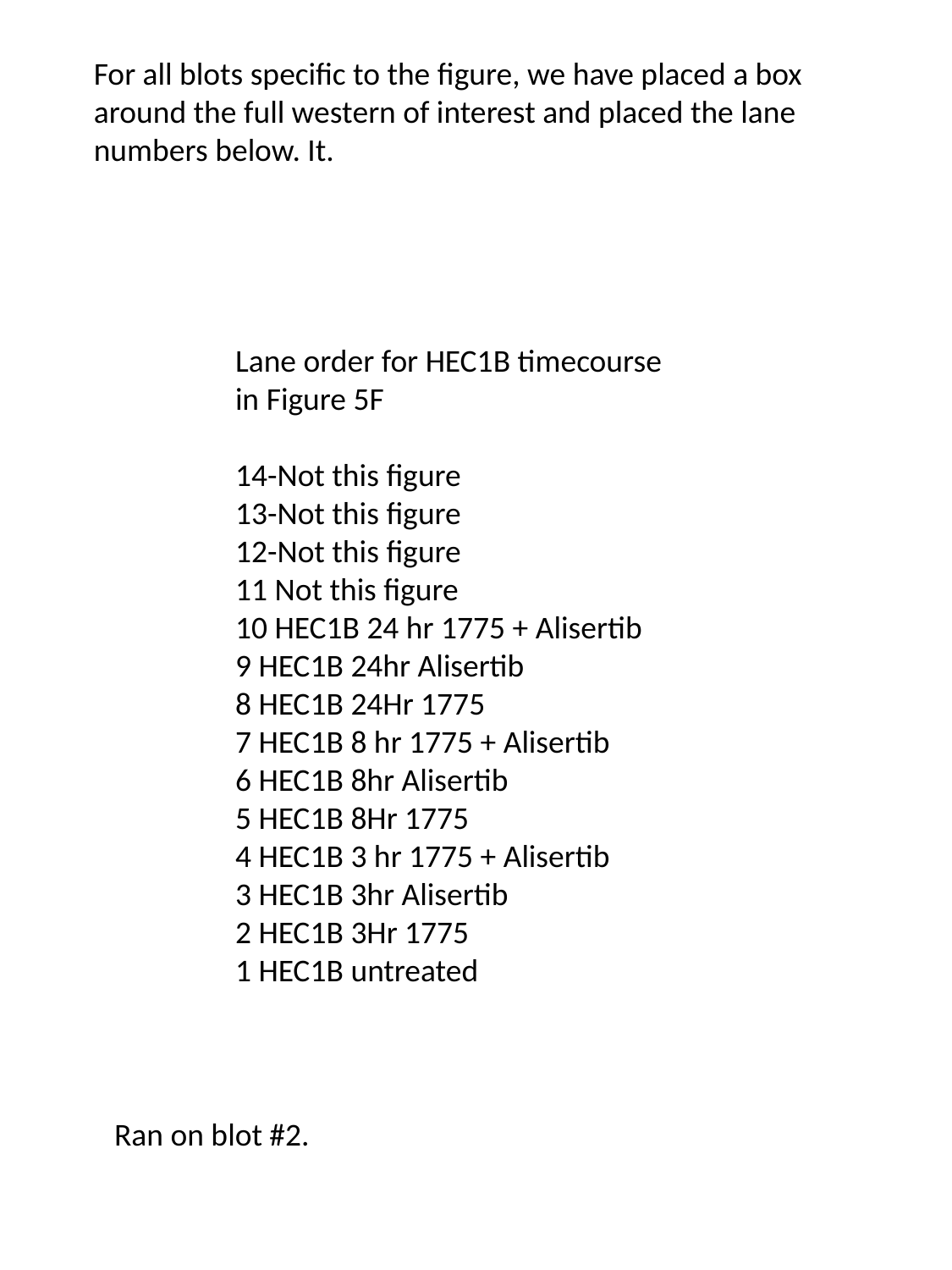

For all blots specific to the figure, we have placed a box around the full western of interest and placed the lane numbers below. It.
Lane order for HEC1B timecourse in Figure 5F
14-Not this figure
13-Not this figure
12-Not this figure
11 Not this figure
10 HEC1B 24 hr 1775 + Alisertib
9 HEC1B 24hr Alisertib
8 HEC1B 24Hr 1775
7 HEC1B 8 hr 1775 + Alisertib
6 HEC1B 8hr Alisertib
5 HEC1B 8Hr 1775
4 HEC1B 3 hr 1775 + Alisertib
3 HEC1B 3hr Alisertib
2 HEC1B 3Hr 1775
1 HEC1B untreated
Ran on blot #2.

## Slide 2
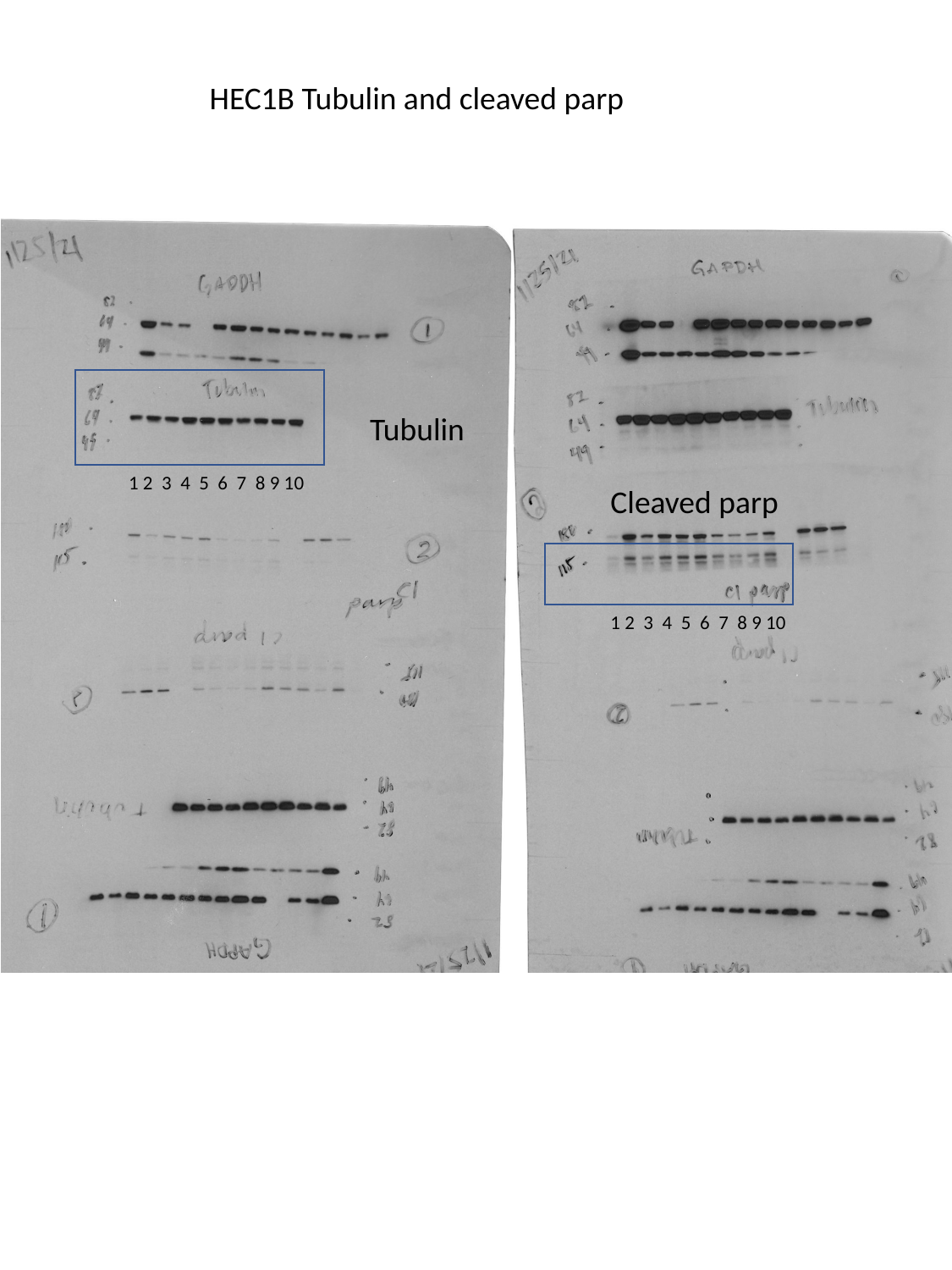

HEC1B Tubulin and cleaved parp
Tubulin
1 2 3 4 5 6 7 8 9 10
Cleaved parp
1 2 3 4 5 6 7 8 9 10
