# Supplementary material for: Enhanced Efficacy of Aurora Kinase Inhibitors in G2/M Checkpoint Deficient TP53 Mutant Uterine Carcinomas Is Linked to the Summation of LKB1–AKT–p53 Interactions
Source: Cancers (Basel). 2021 May 3;13(9):2195. doi: 10.3390/cancers13092195 (PMC8125555; doi:10.3390/cancers13092195)
Supplement: Supplementary file 1 [file cancers-13-02195-s001.zip › Lynch and Hill Supplementary Matierals/original blot/Figure S4A.pptx]

## Slide 1
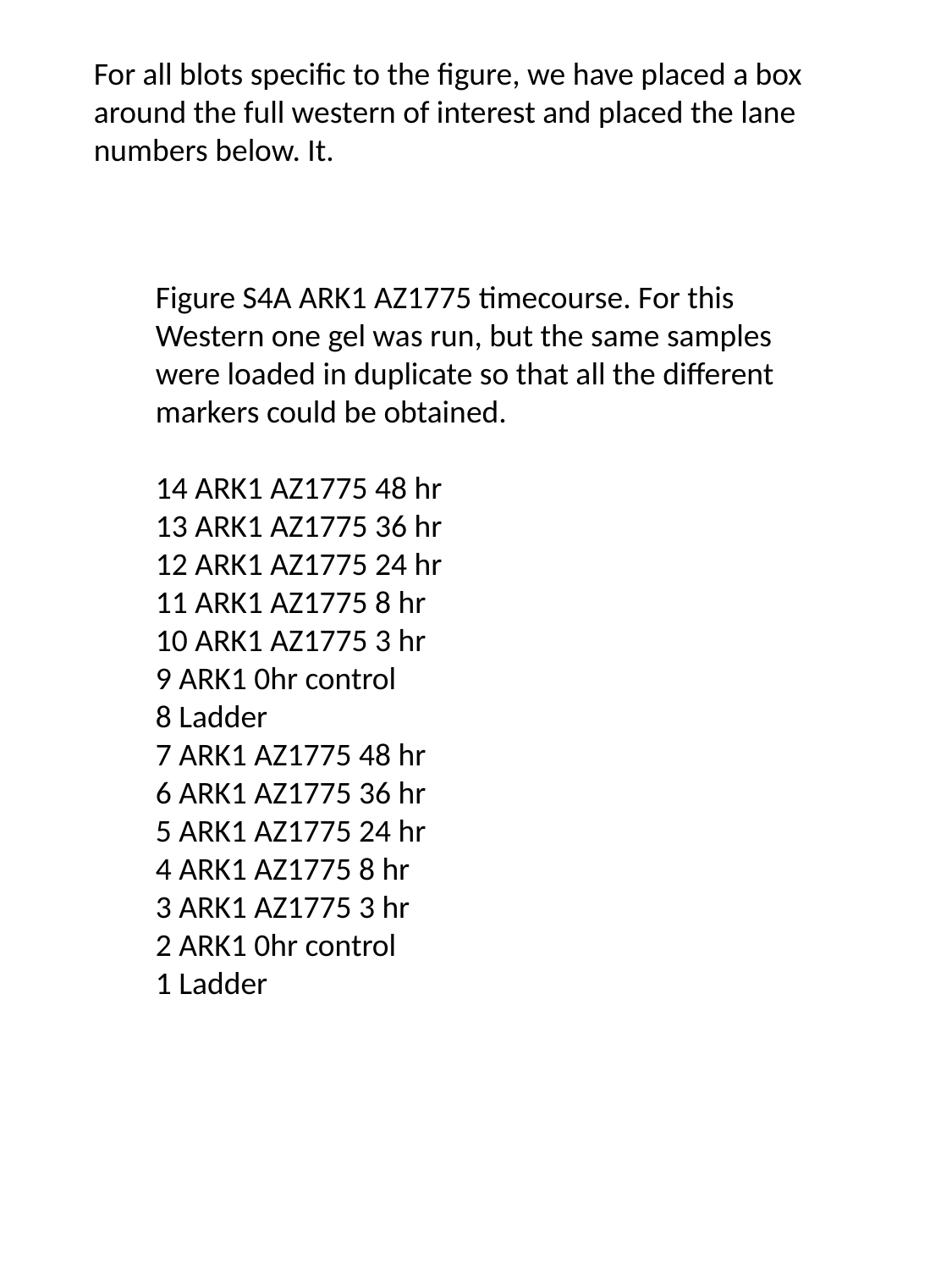

For all blots specific to the figure, we have placed a box around the full western of interest and placed the lane numbers below. It.
Figure S4A ARK1 AZ1775 timecourse. For this Western one gel was run, but the same samples were loaded in duplicate so that all the different markers could be obtained.
14 ARK1 AZ1775 48 hr
13 ARK1 AZ1775 36 hr
12 ARK1 AZ1775 24 hr
11 ARK1 AZ1775 8 hr
10 ARK1 AZ1775 3 hr
9 ARK1 0hr control
8 Ladder
7 ARK1 AZ1775 48 hr
6 ARK1 AZ1775 36 hr
5 ARK1 AZ1775 24 hr
4 ARK1 AZ1775 8 hr
3 ARK1 AZ1775 3 hr
2 ARK1 0hr control
1 Ladder

## Slide 2
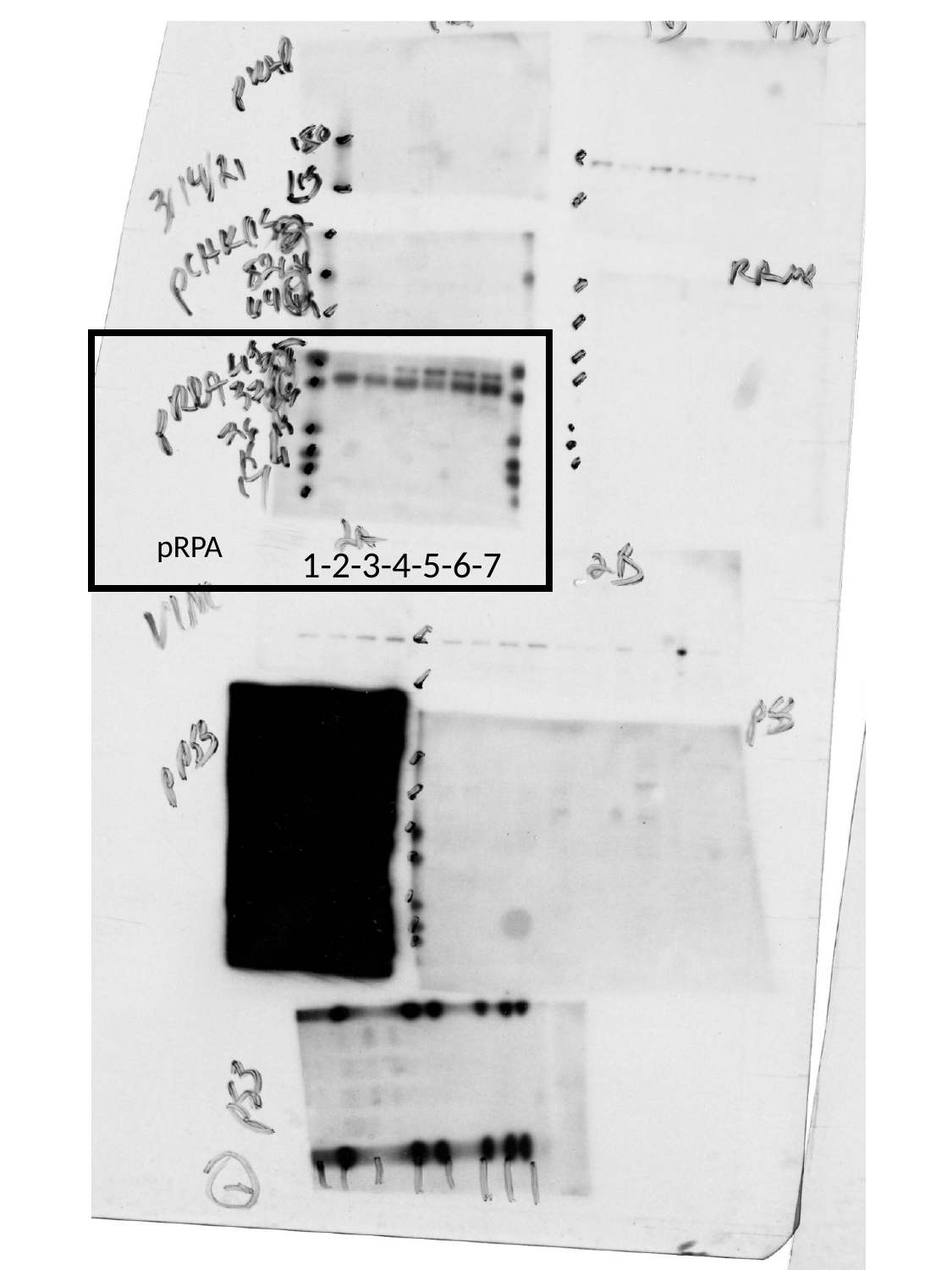

pRPA
1-2-3-4-5-6-7

## Slide 3
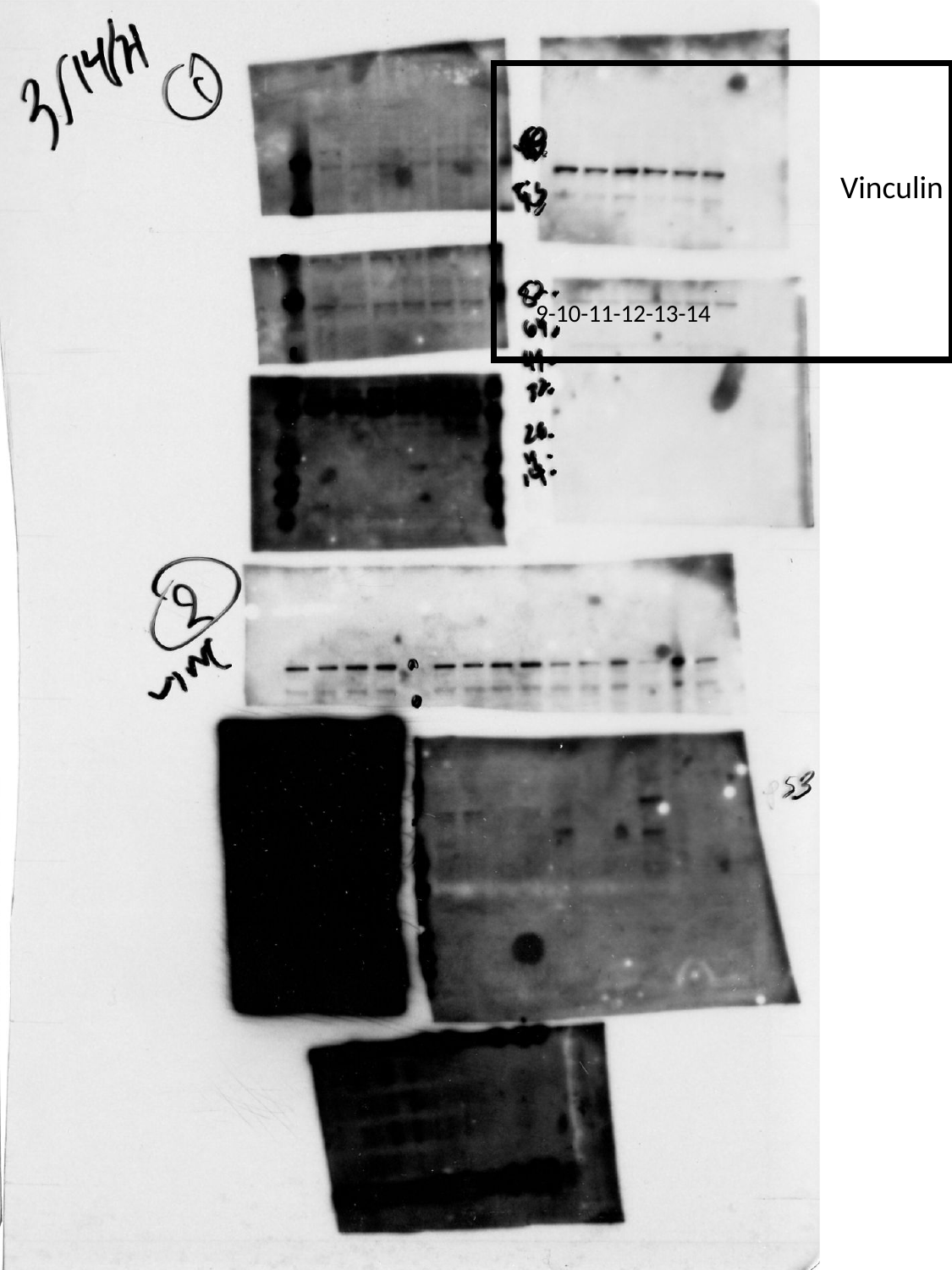

Vinculin
9-10-11-12-13-14

## Slide 4
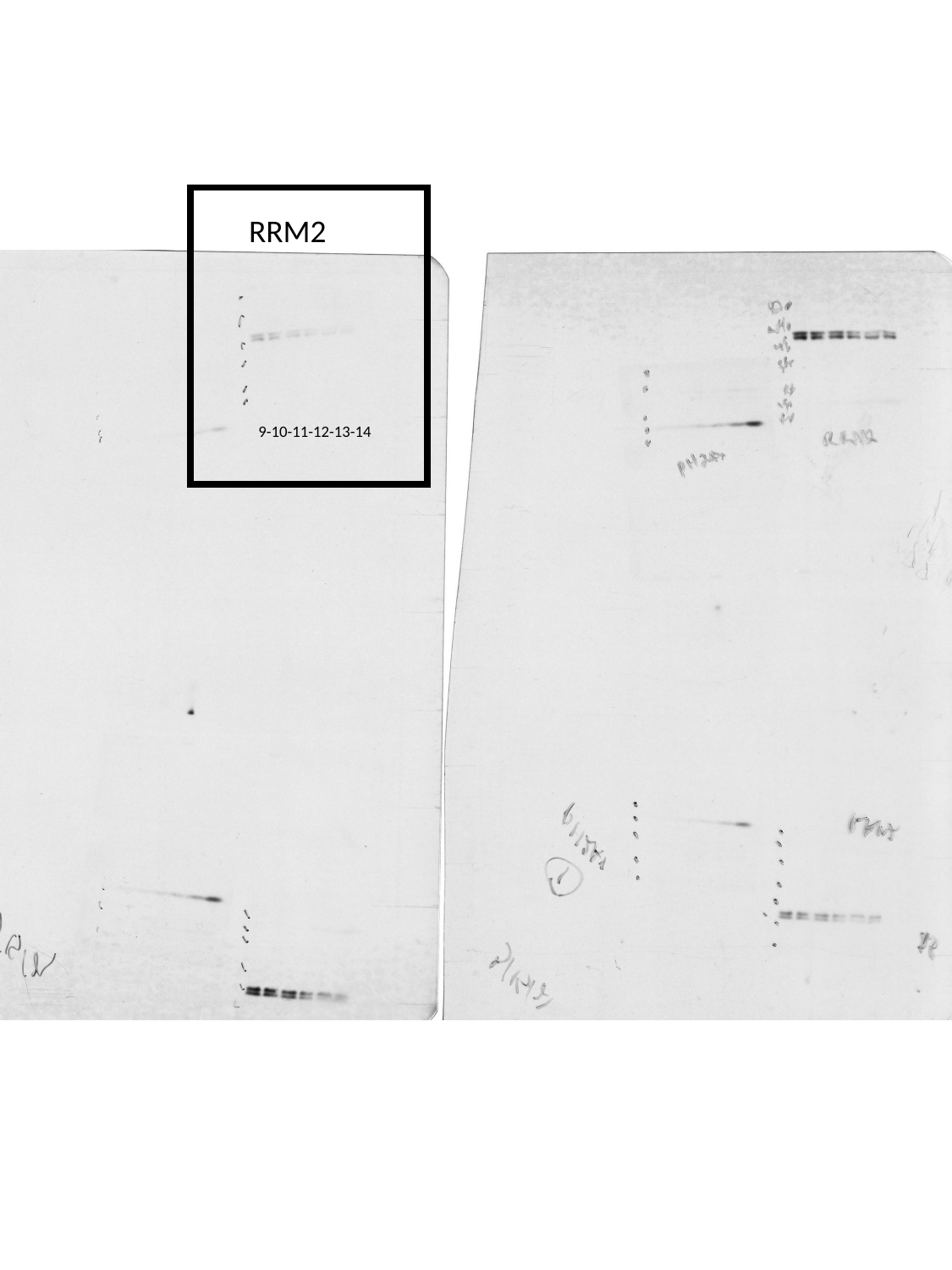

RRM2
9-10-11-12-13-14

## Slide 5
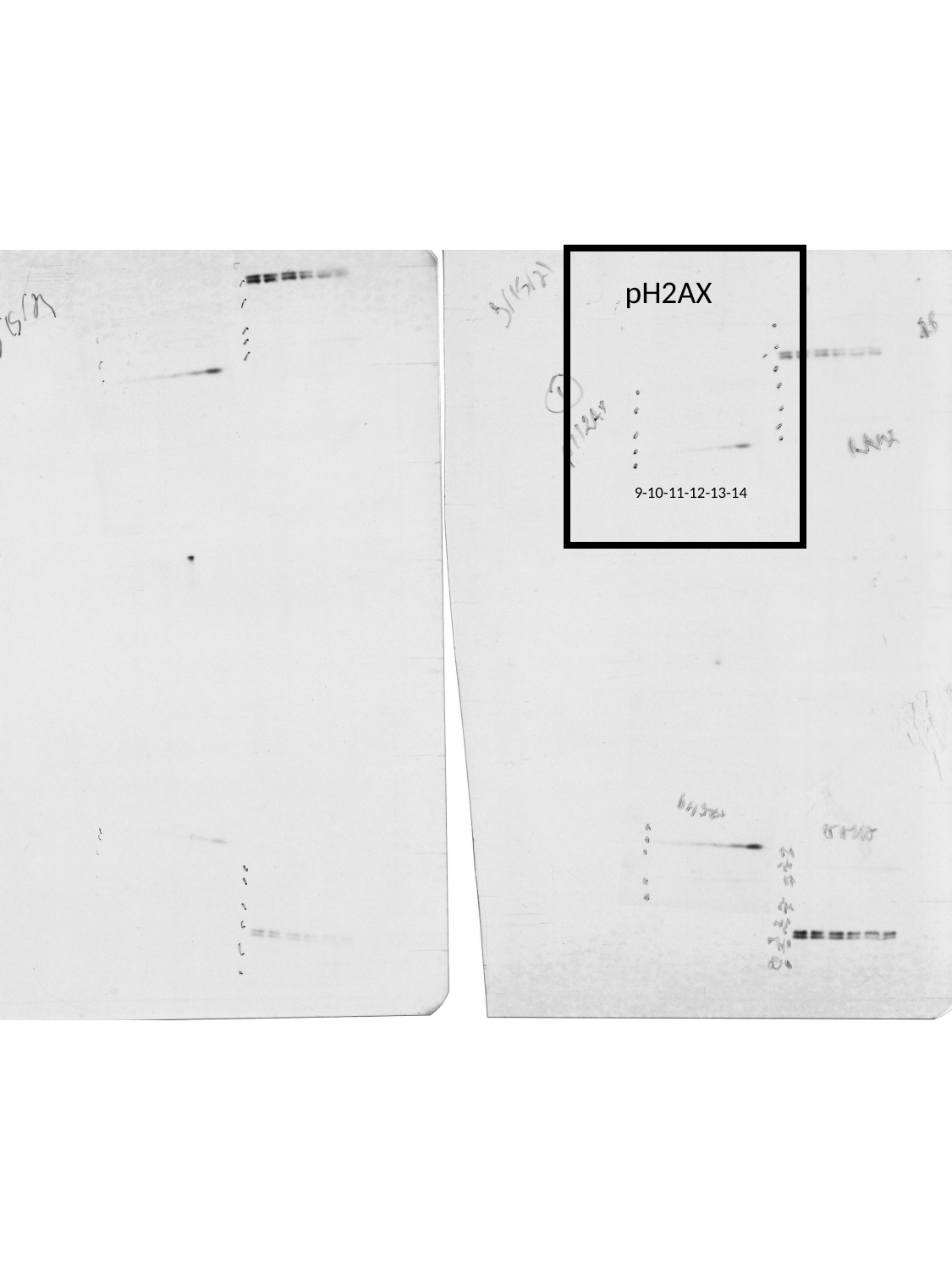

pH2AX
9-10-11-12-13-14

## Slide 6
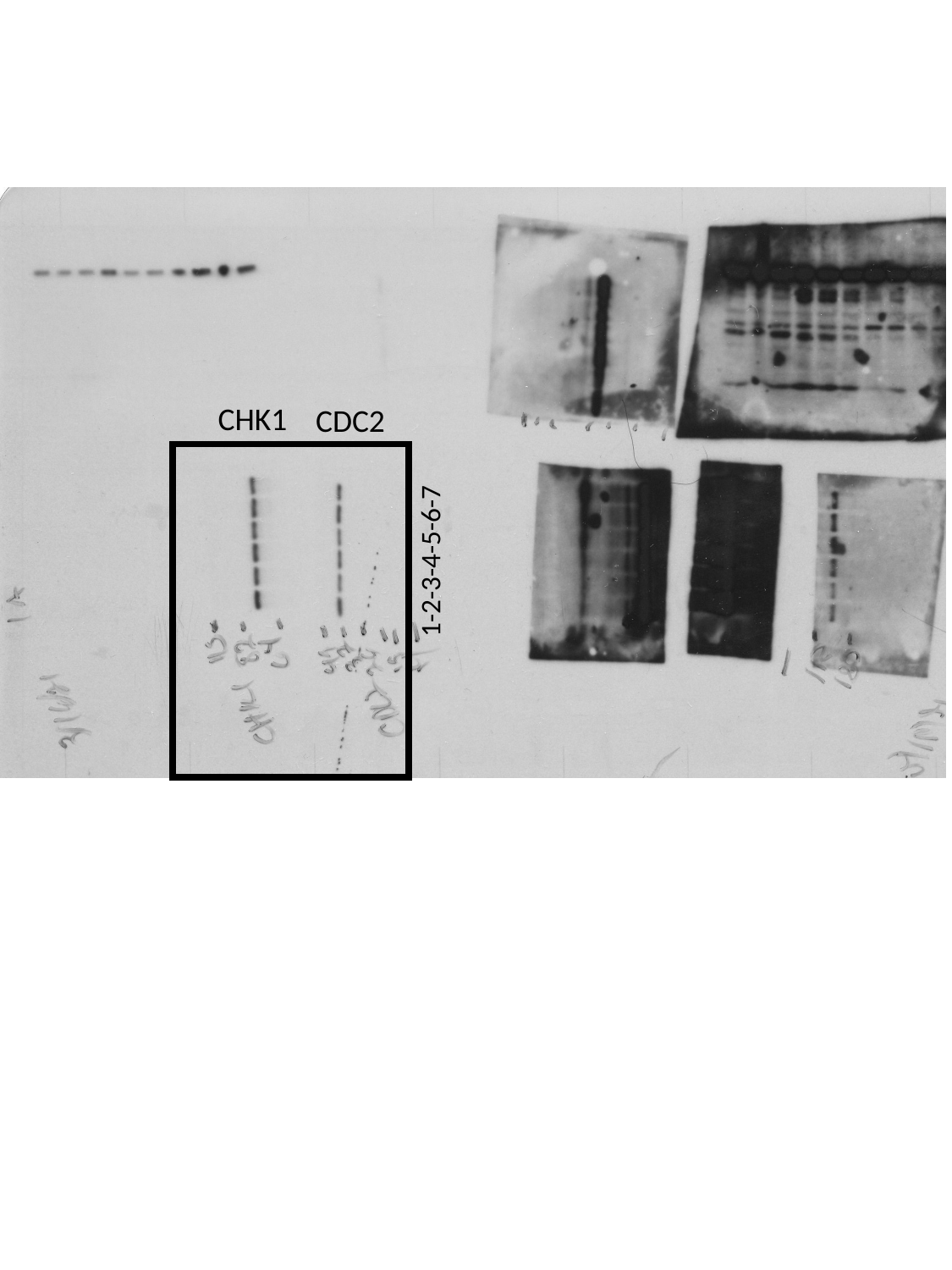

CHK1
CDC2
1-2-3-4-5-6-7

## Slide 7
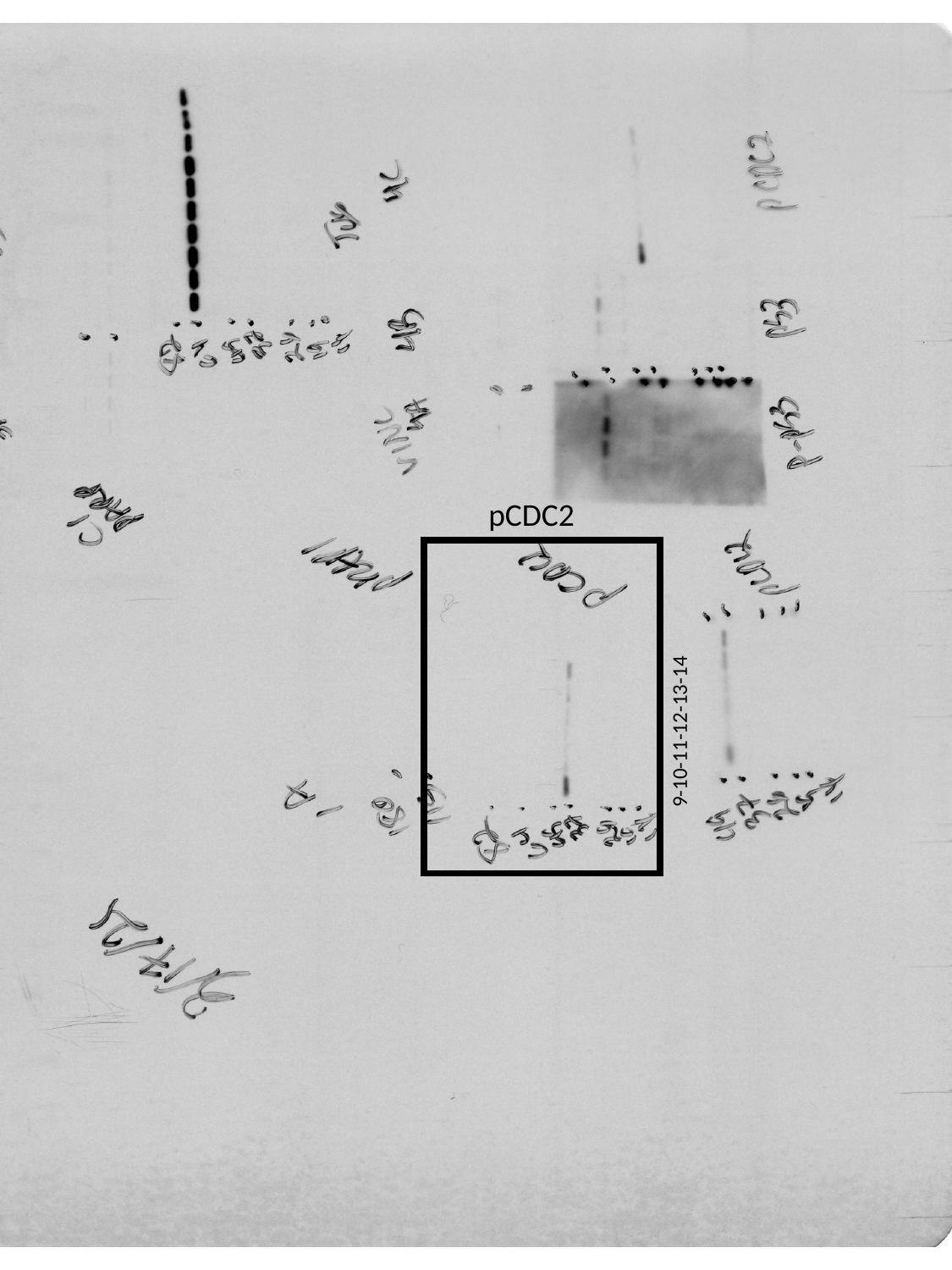

pCDC2
9-10-11-12-13-14

## Slide 8
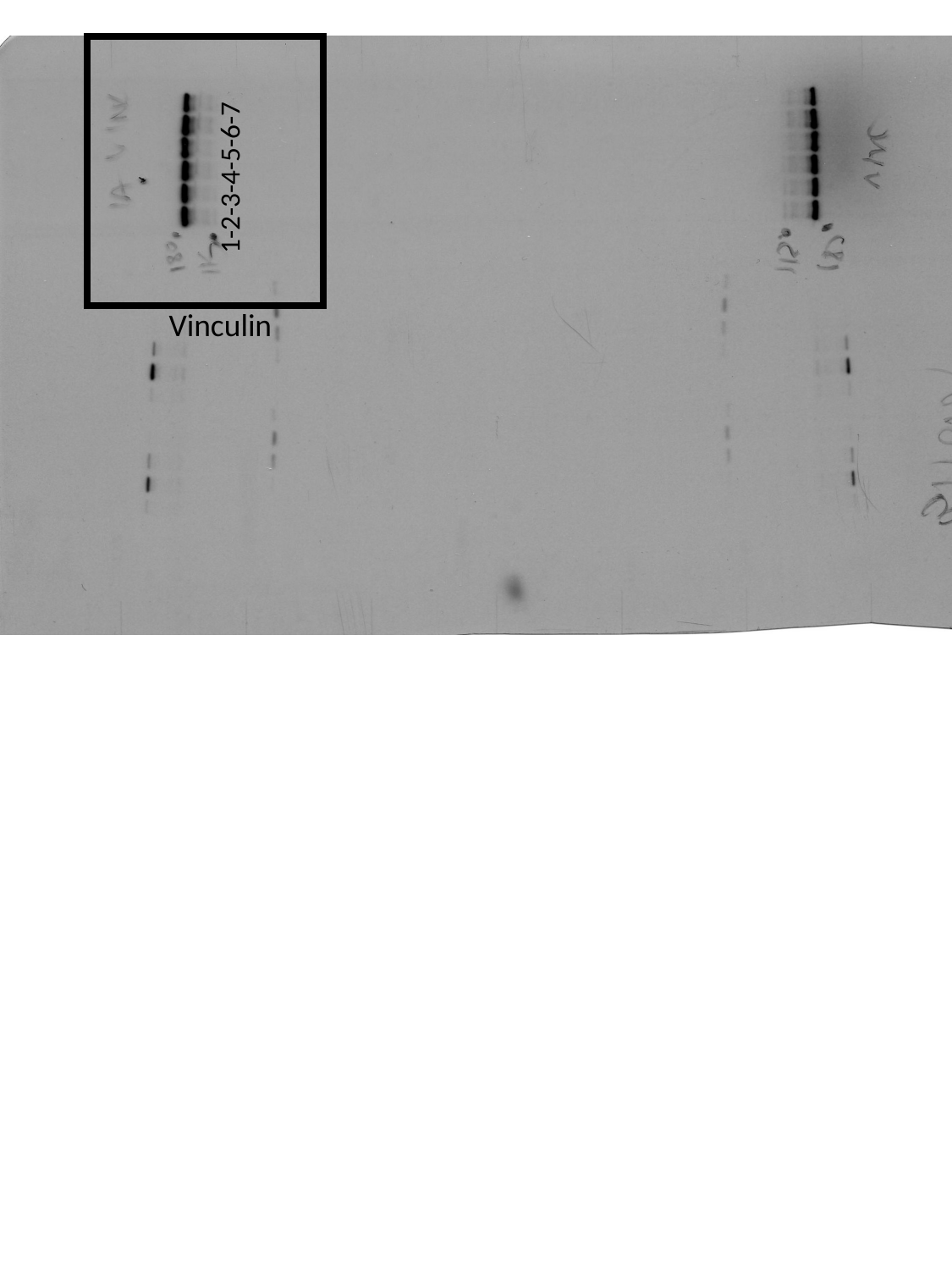

1-2-3-4-5-6-7
Vinculin

## Slide 9
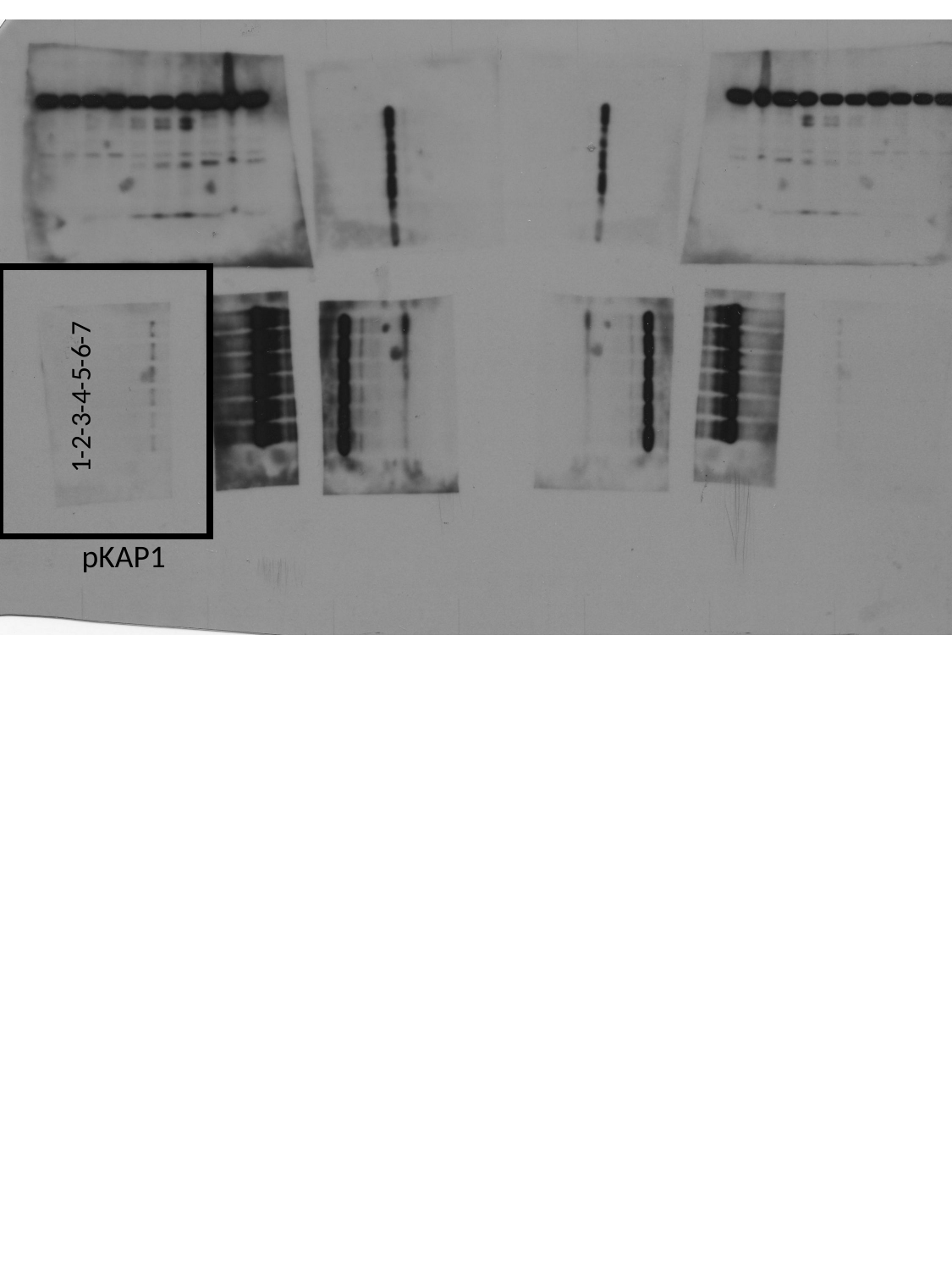

1-2-3-4-5-6-7
pKAP1

## Slide 10
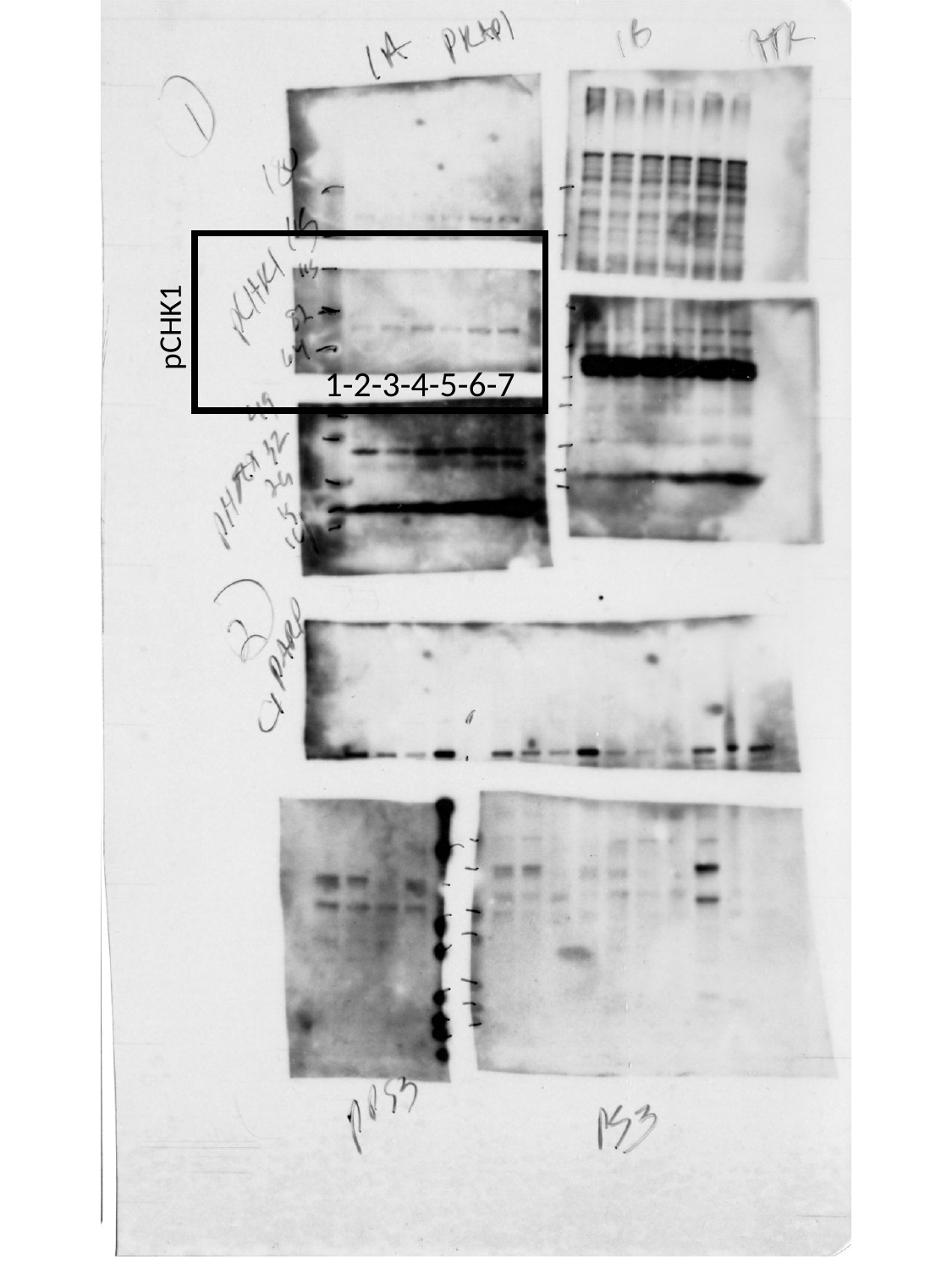

pCHK1
1-2-3-4-5-6-7

## Slide 11
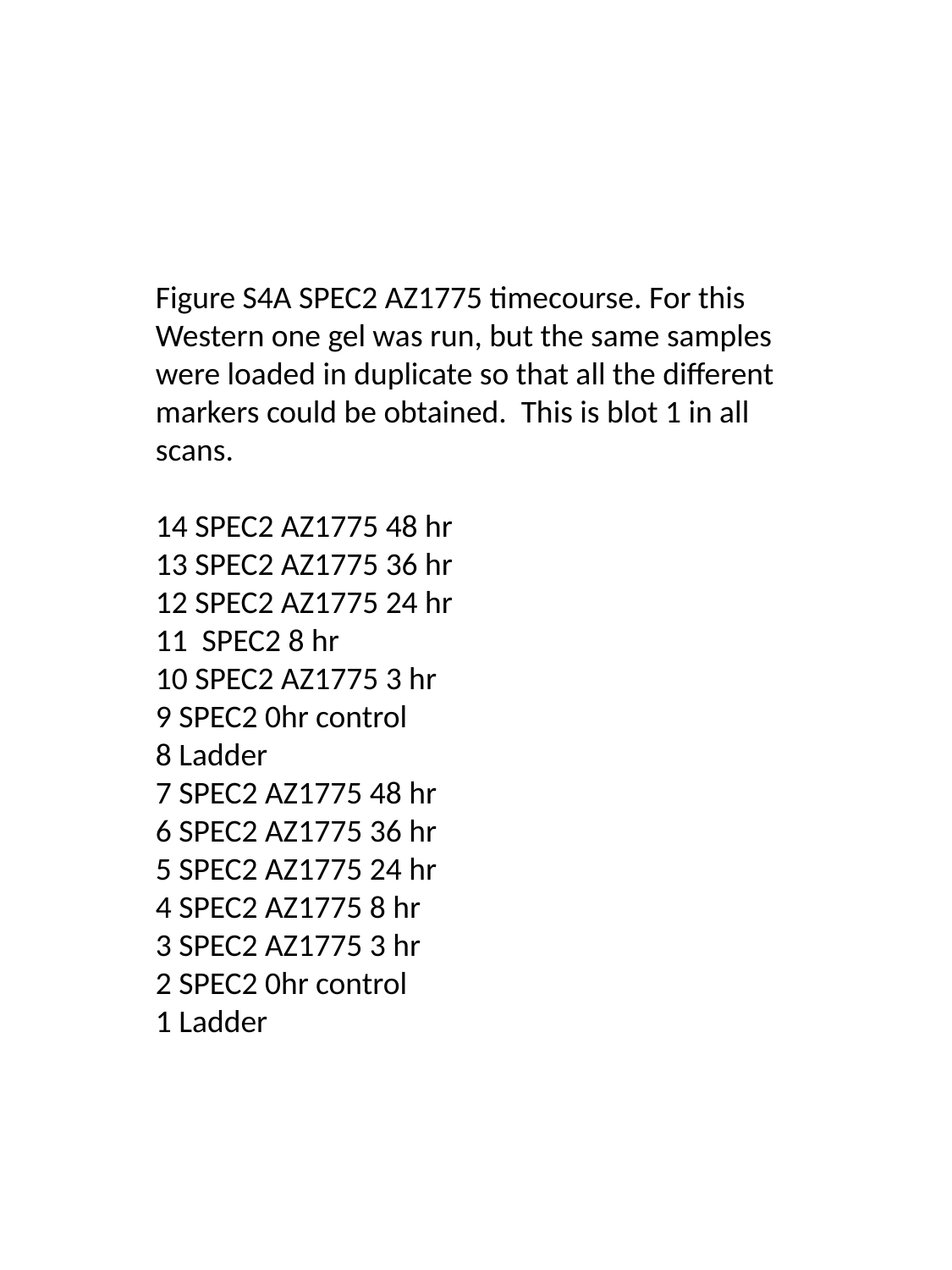

Figure S4A SPEC2 AZ1775 timecourse. For this Western one gel was run, but the same samples were loaded in duplicate so that all the different markers could be obtained. This is blot 1 in all scans.
14 SPEC2 AZ1775 48 hr
13 SPEC2 AZ1775 36 hr
12 SPEC2 AZ1775 24 hr
11 SPEC2 8 hr
10 SPEC2 AZ1775 3 hr
9 SPEC2 0hr control
8 Ladder
7 SPEC2 AZ1775 48 hr
6 SPEC2 AZ1775 36 hr
5 SPEC2 AZ1775 24 hr
4 SPEC2 AZ1775 8 hr
3 SPEC2 AZ1775 3 hr
2 SPEC2 0hr control
1 Ladder

## Slide 12
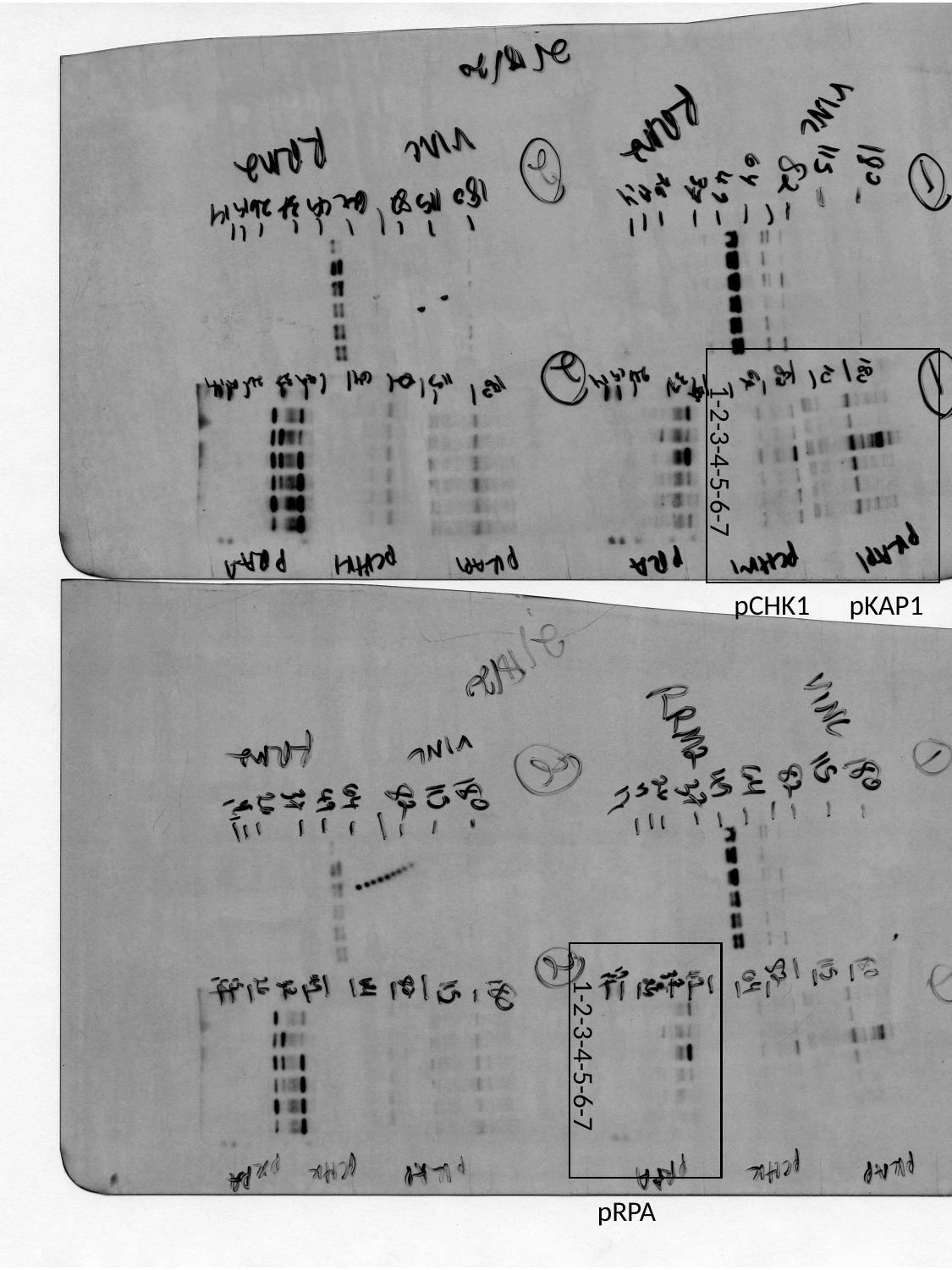

1-2-3-4-5-6-7
pCHK1
pKAP1
1-2-3-4-5-6-7
pRPA

## Slide 13
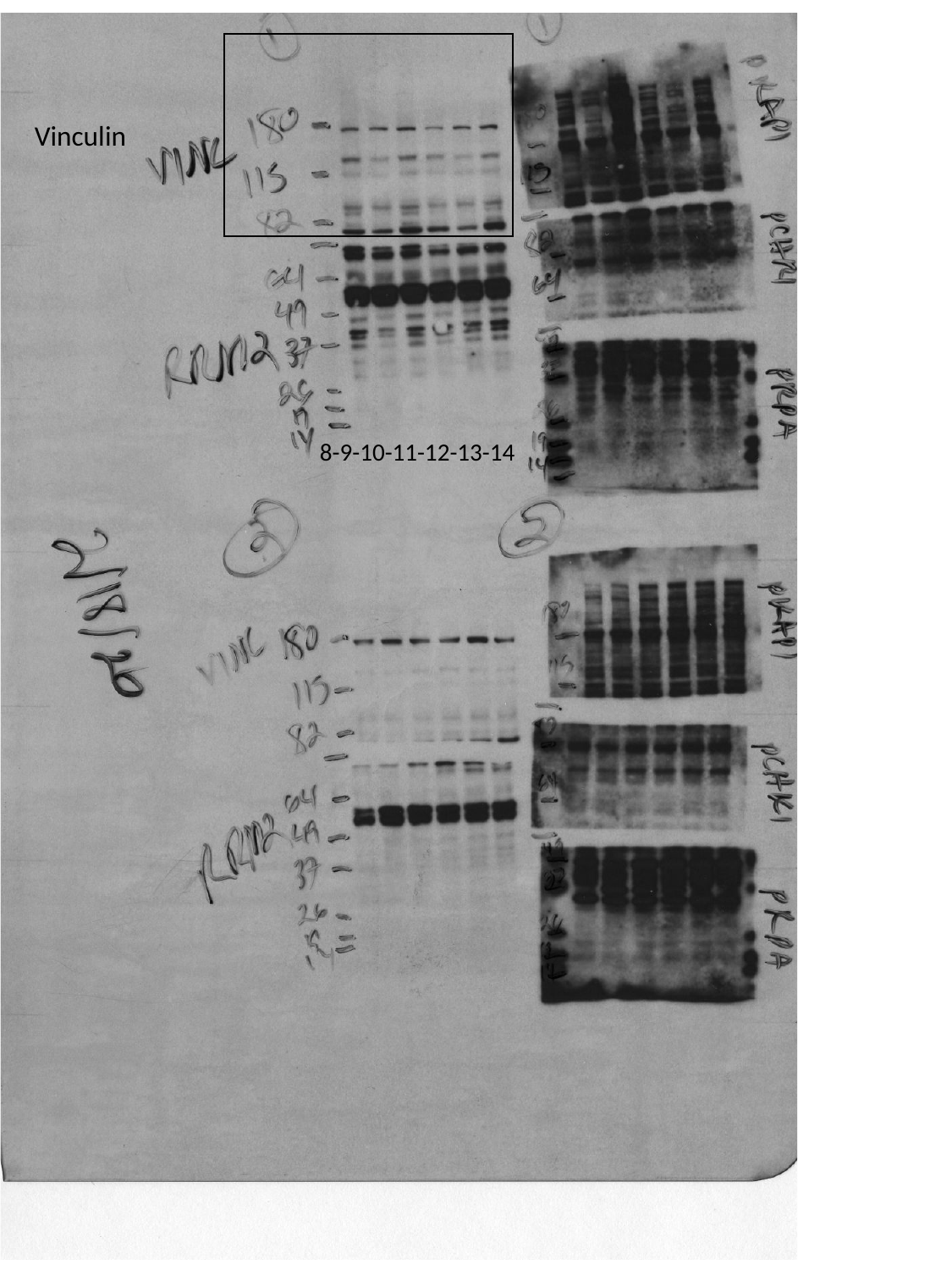

Vinculin
8-9-10-11-12-13-14

## Slide 14
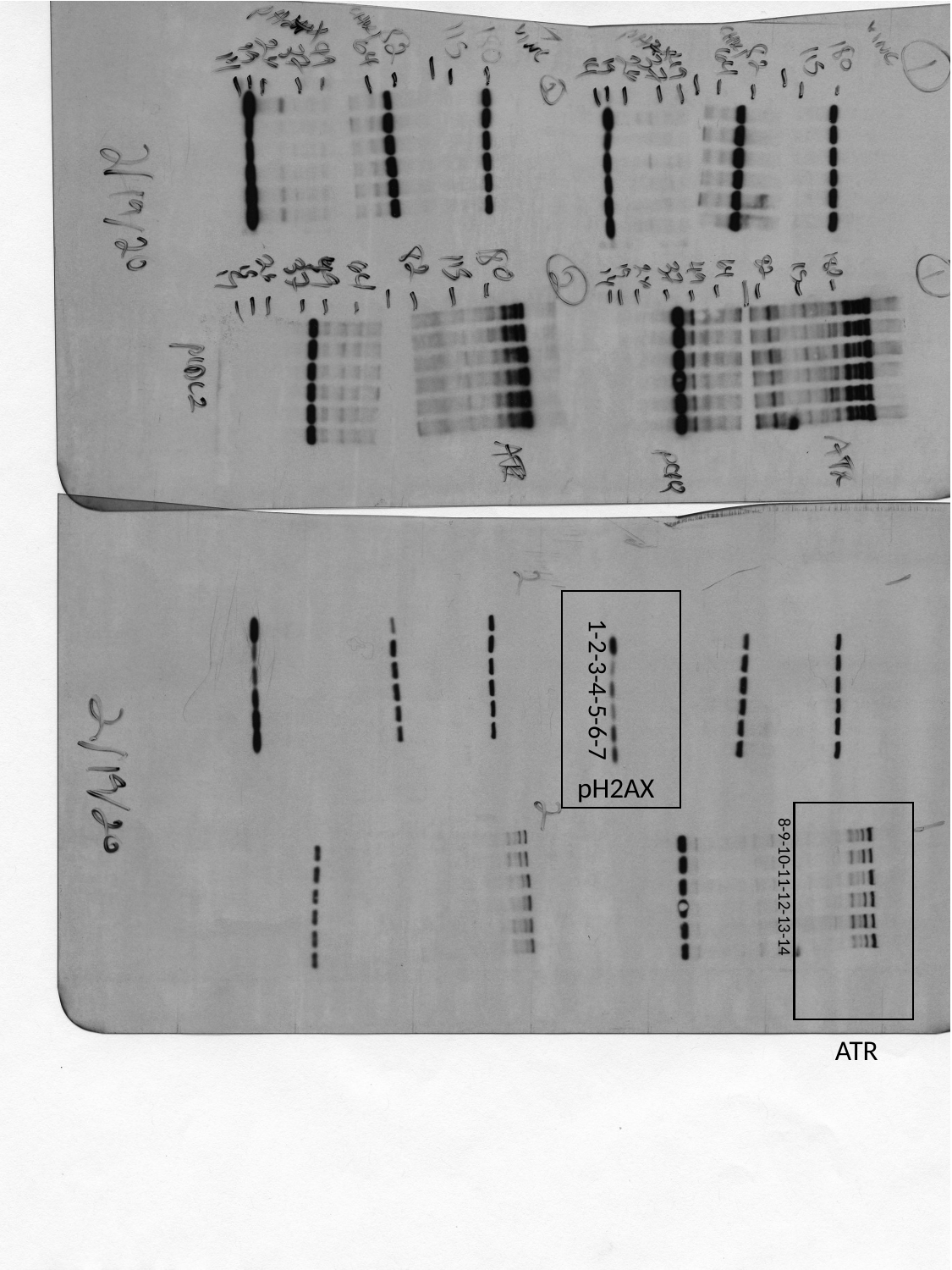

1-2-3-4-5-6-7
pH2AX
8-9-10-11-12-13-14
ATR

## Slide 15
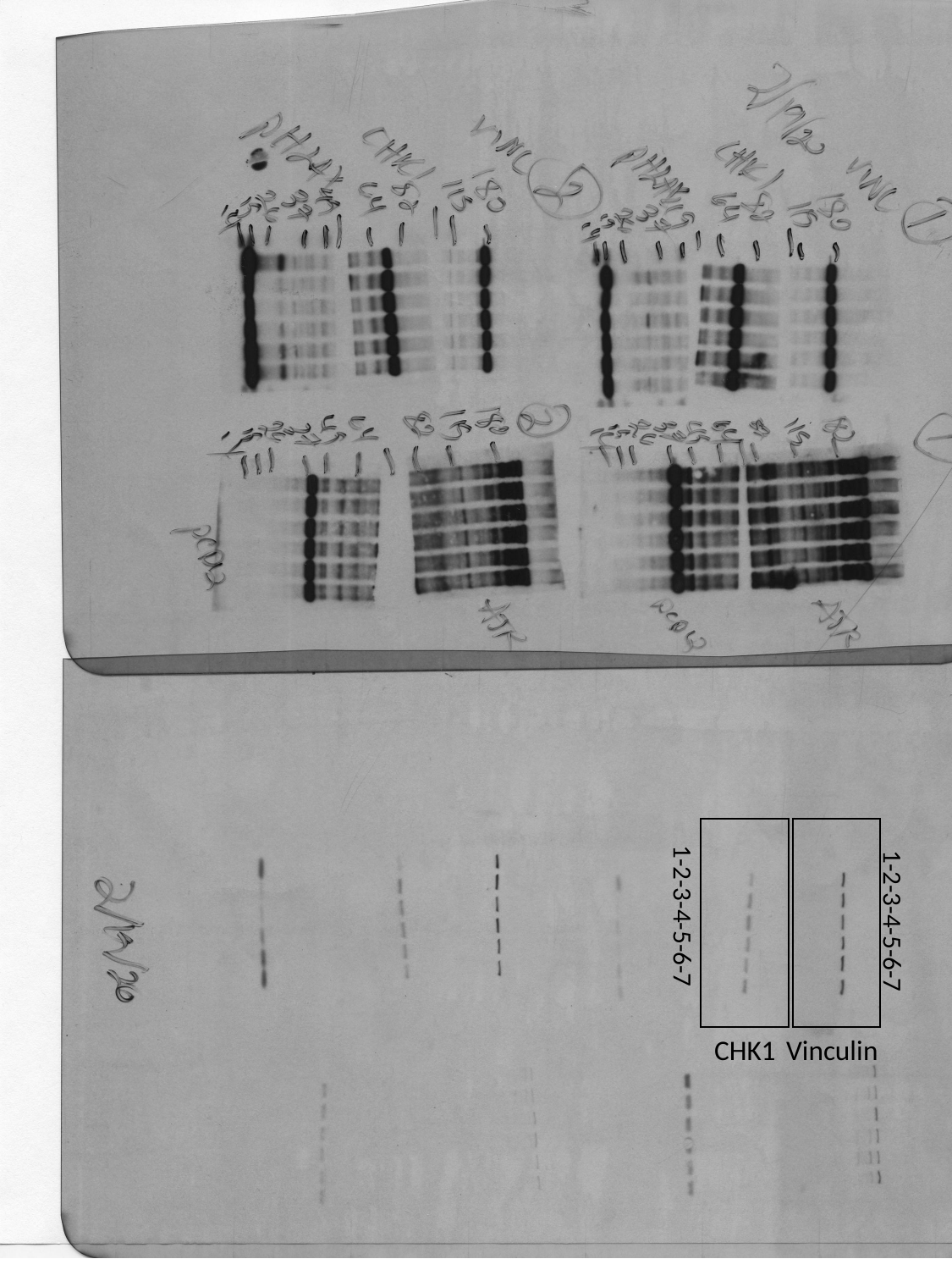

1-2-3-4-5-6-7
1-2-3-4-5-6-7
Vinculin
CHK1

## Slide 16
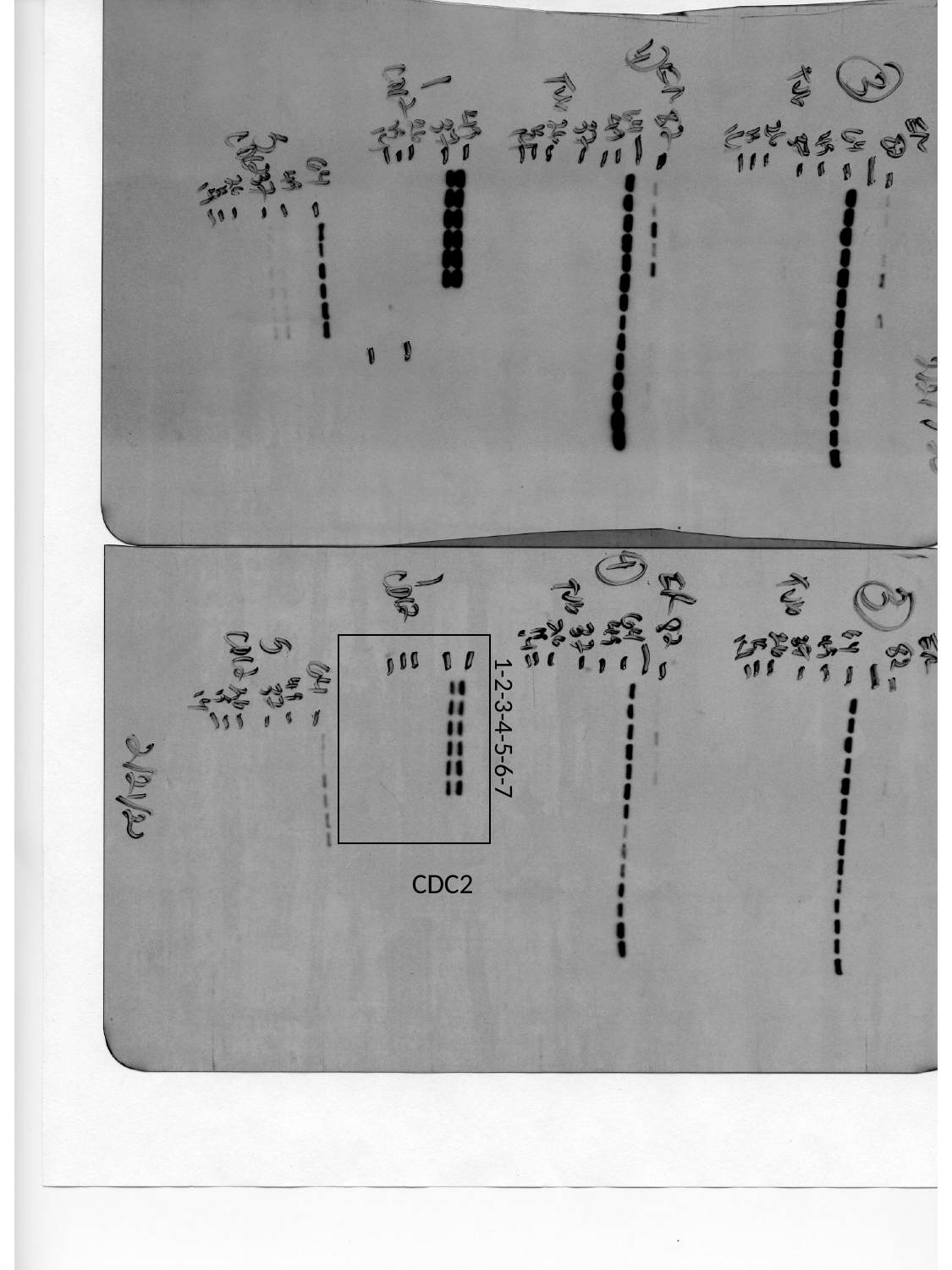

1-2-3-4-5-6-7
CDC2

## Slide 17
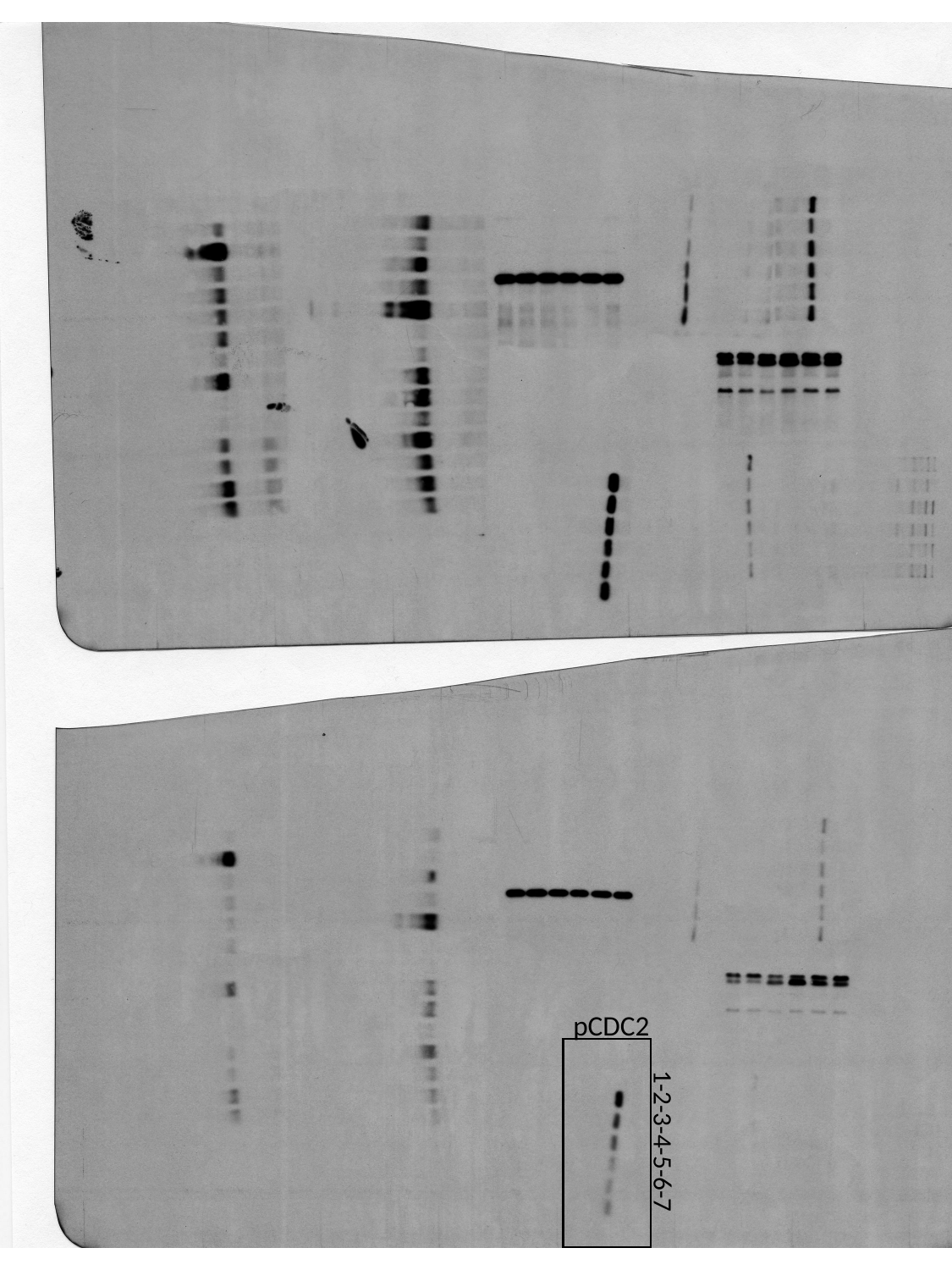

pCDC2
1-2-3-4-5-6-7

## Slide 18
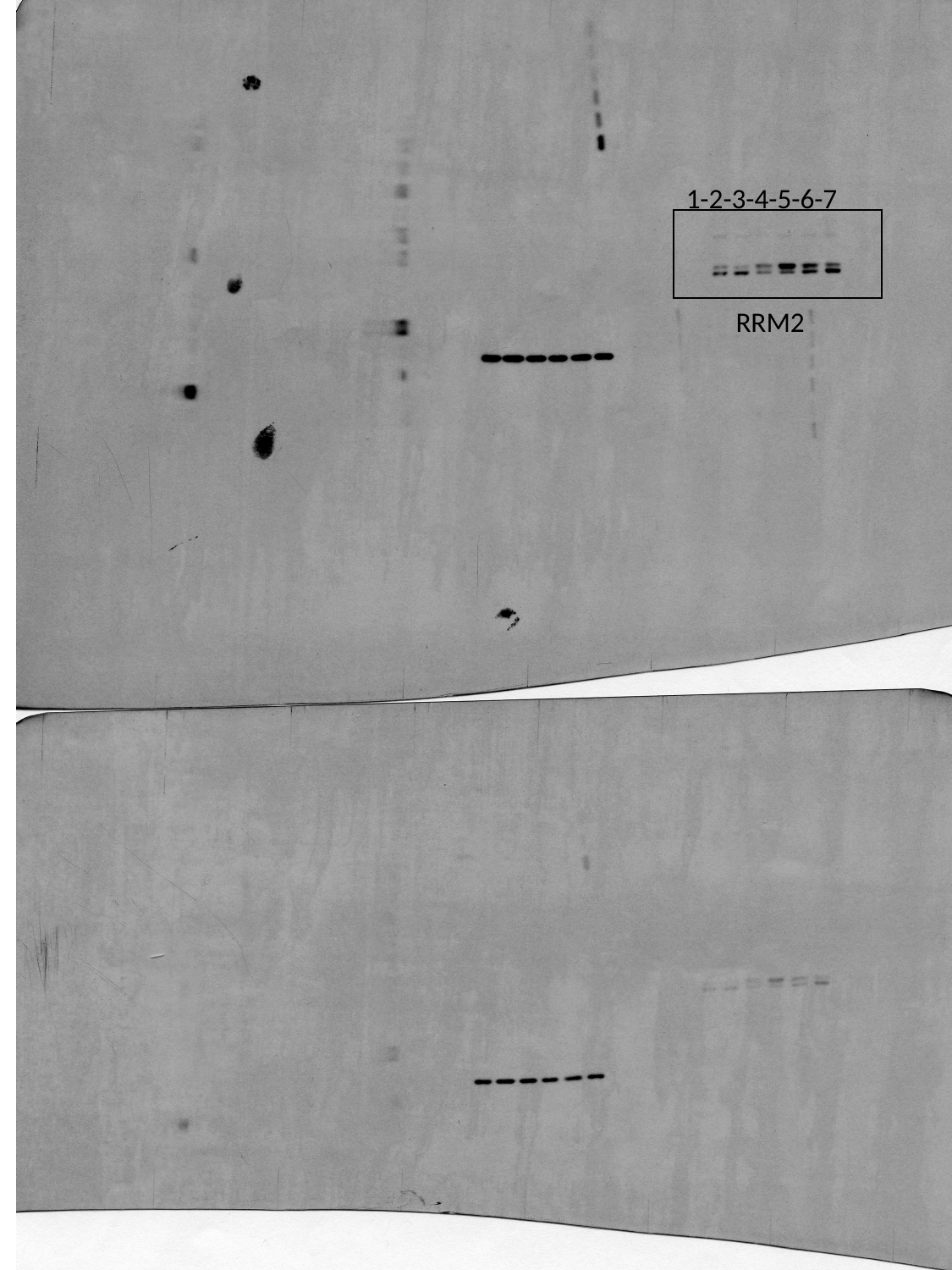

1-2-3-4-5-6-7
RRM2

## Slide 19
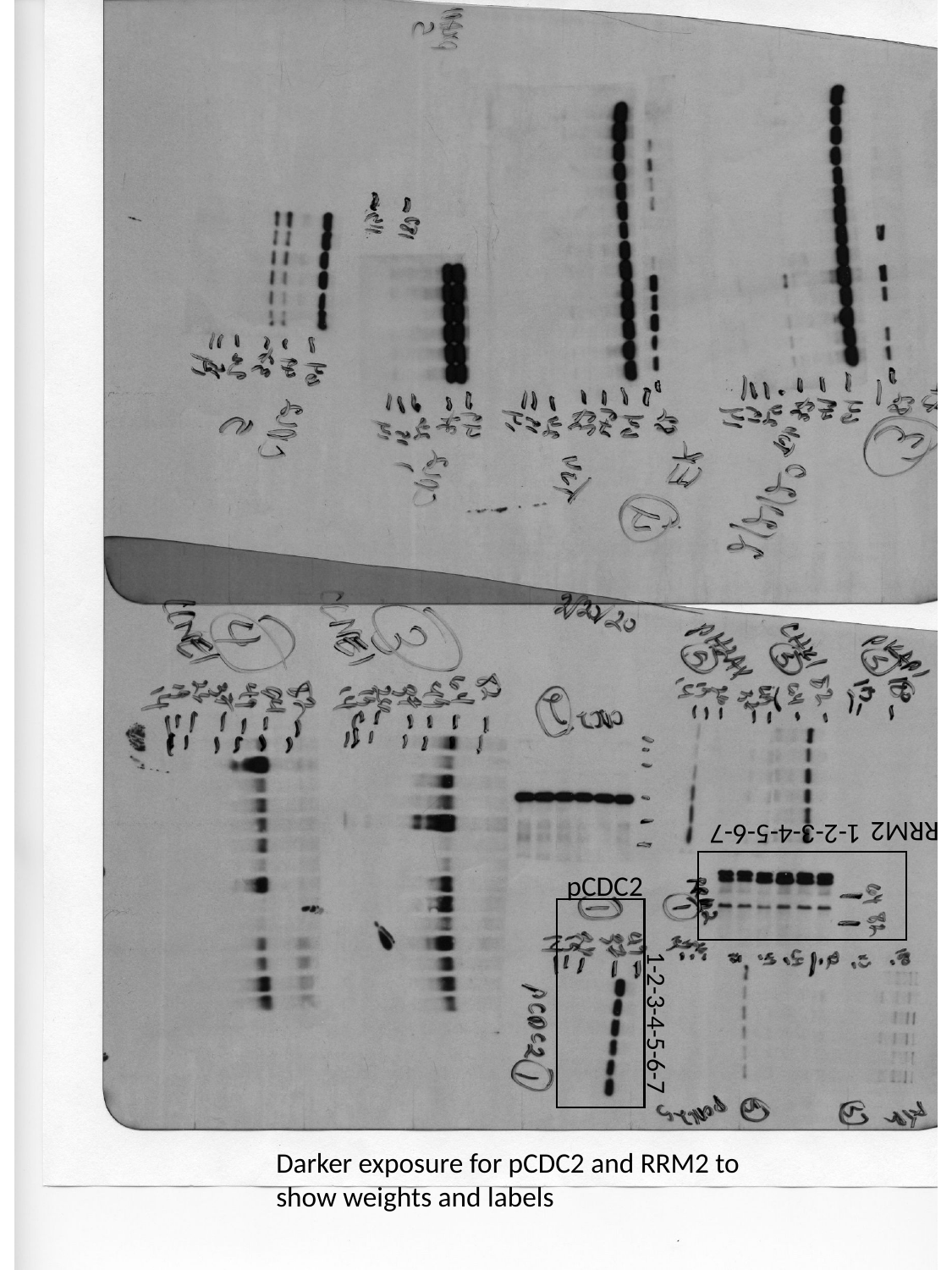

RRM2
1-2-3-4-5-6-7
pCDC2
1-2-3-4-5-6-7
Darker exposure for pCDC2 and RRM2 to show weights and labels

## Slide 20
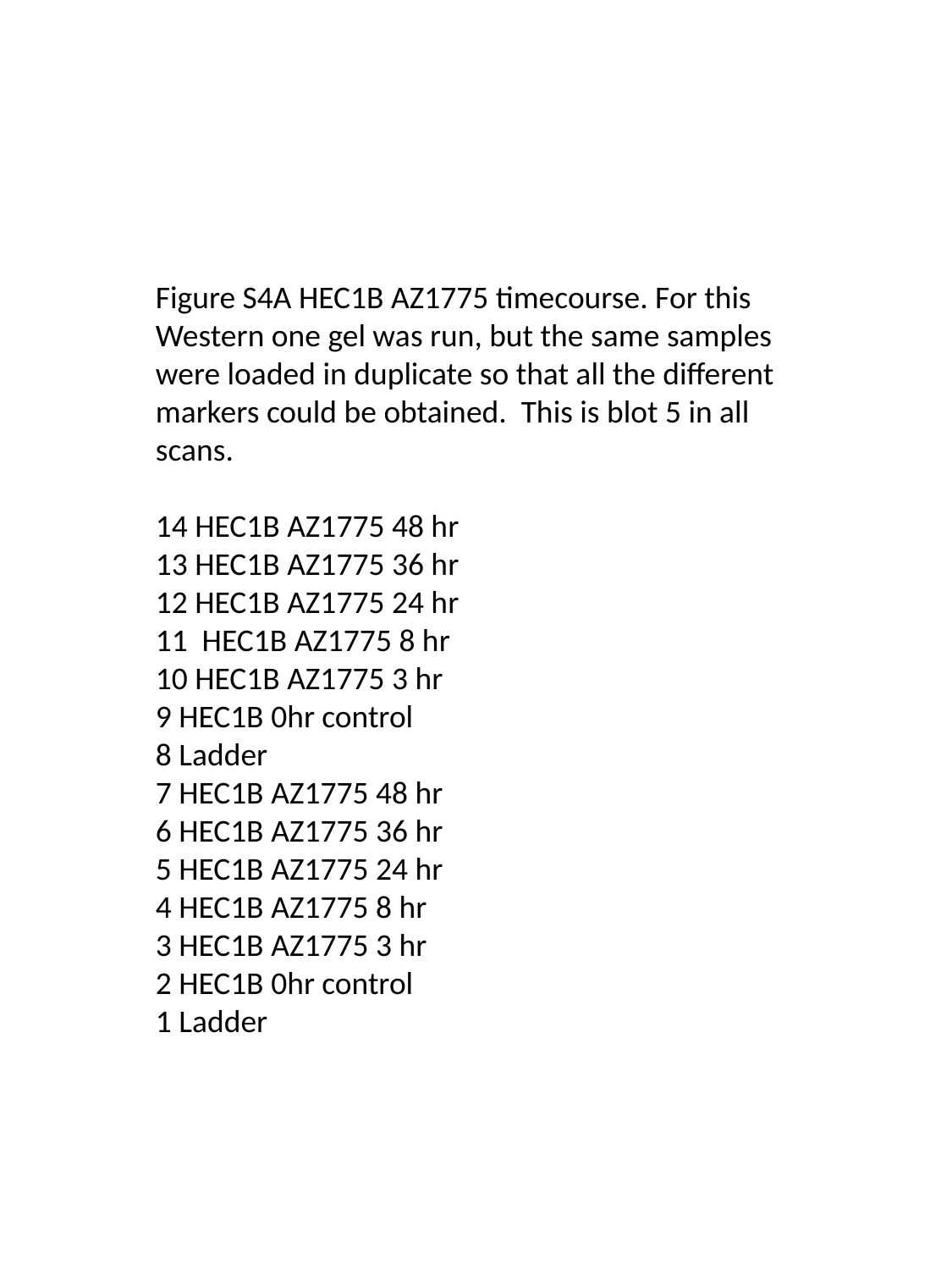

Figure S4A HEC1B AZ1775 timecourse. For this Western one gel was run, but the same samples were loaded in duplicate so that all the different markers could be obtained. This is blot 5 in all scans.
14 HEC1B AZ1775 48 hr
13 HEC1B AZ1775 36 hr
12 HEC1B AZ1775 24 hr
11 HEC1B AZ1775 8 hr
10 HEC1B AZ1775 3 hr
9 HEC1B 0hr control
8 Ladder
7 HEC1B AZ1775 48 hr
6 HEC1B AZ1775 36 hr
5 HEC1B AZ1775 24 hr
4 HEC1B AZ1775 8 hr
3 HEC1B AZ1775 3 hr
2 HEC1B 0hr control
1 Ladder

## Slide 21
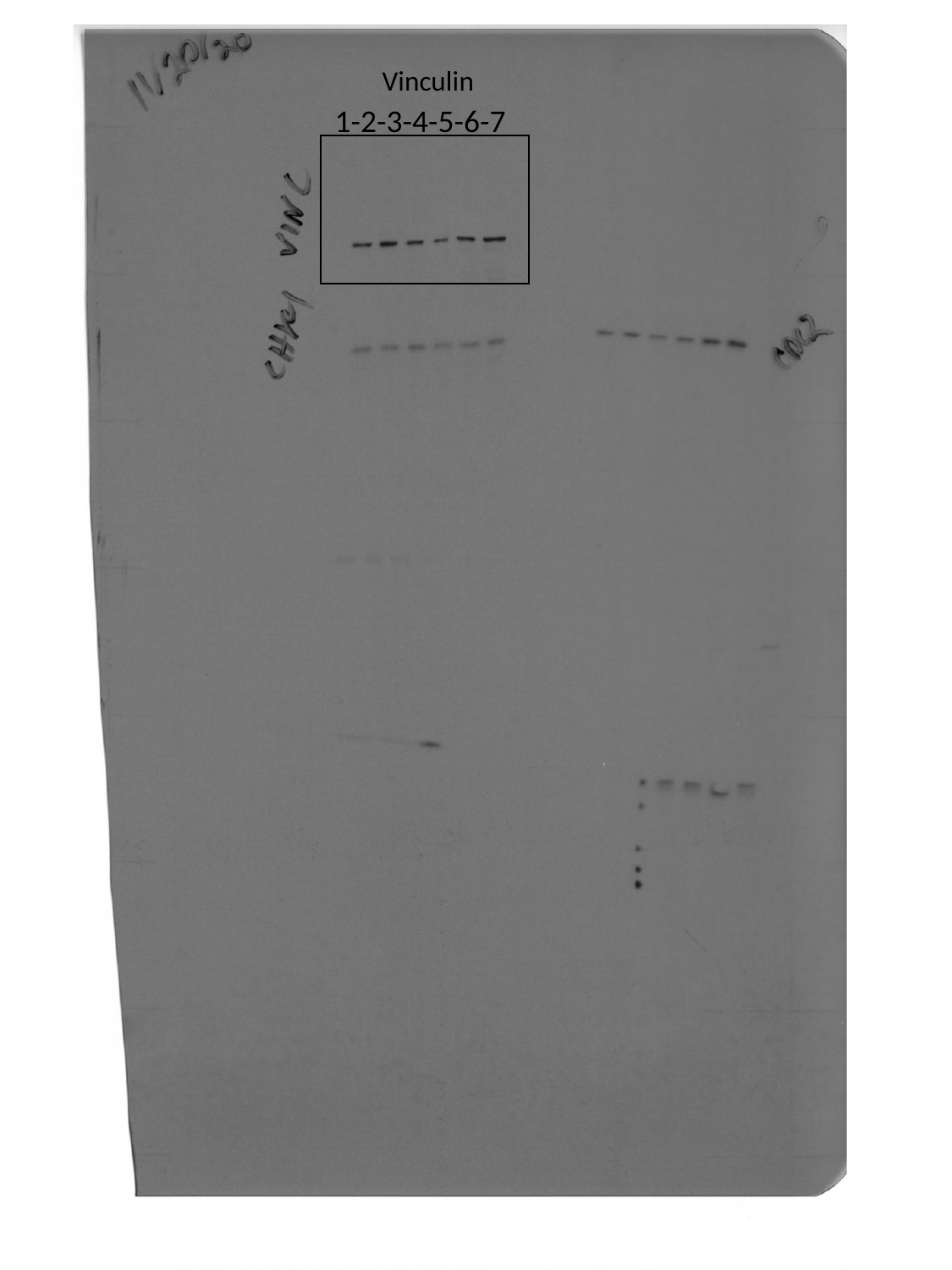

Vinculin
1-2-3-4-5-6-7

## Slide 22
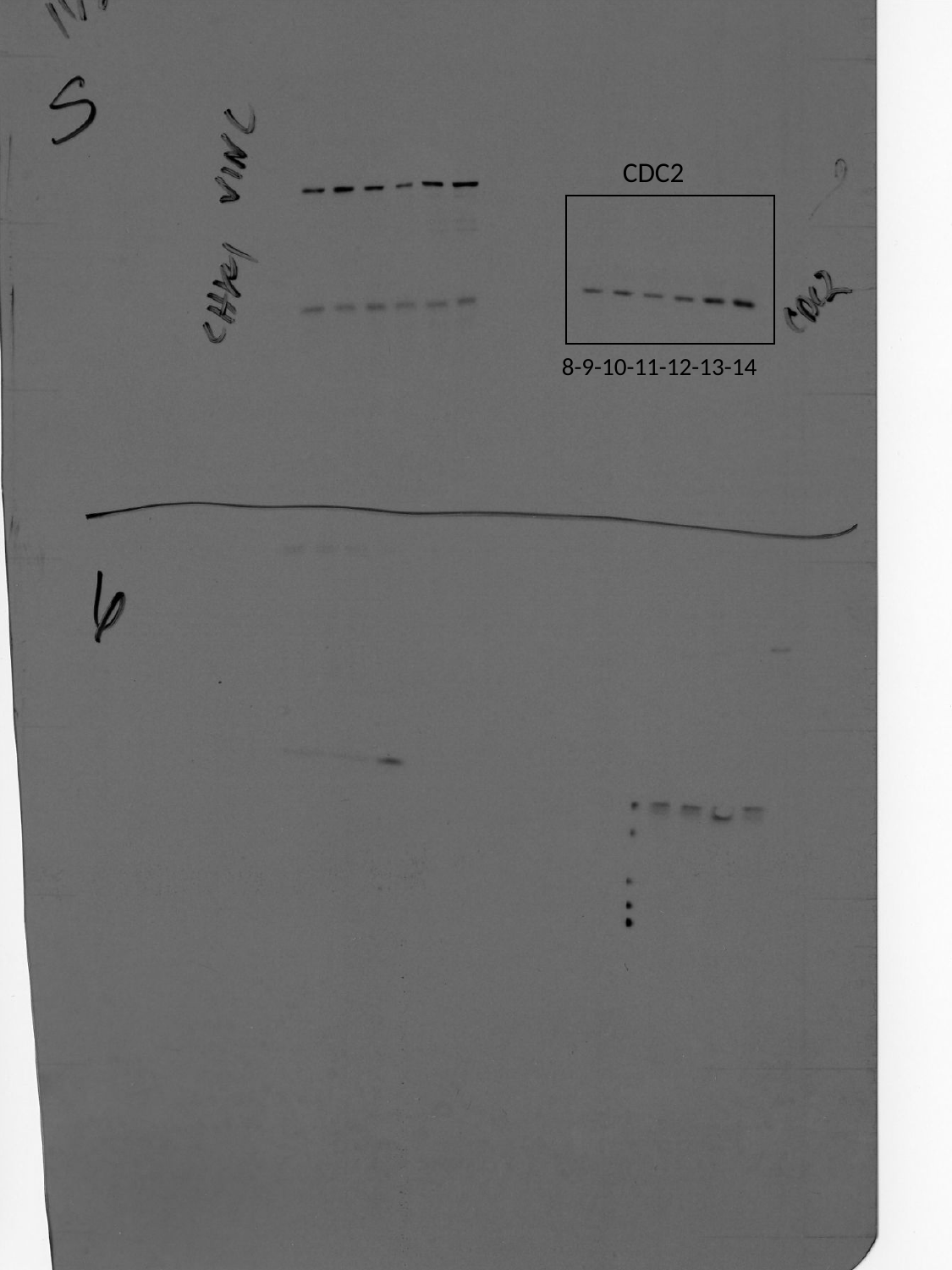

CDC2
8-9-10-11-12-13-14

## Slide 23
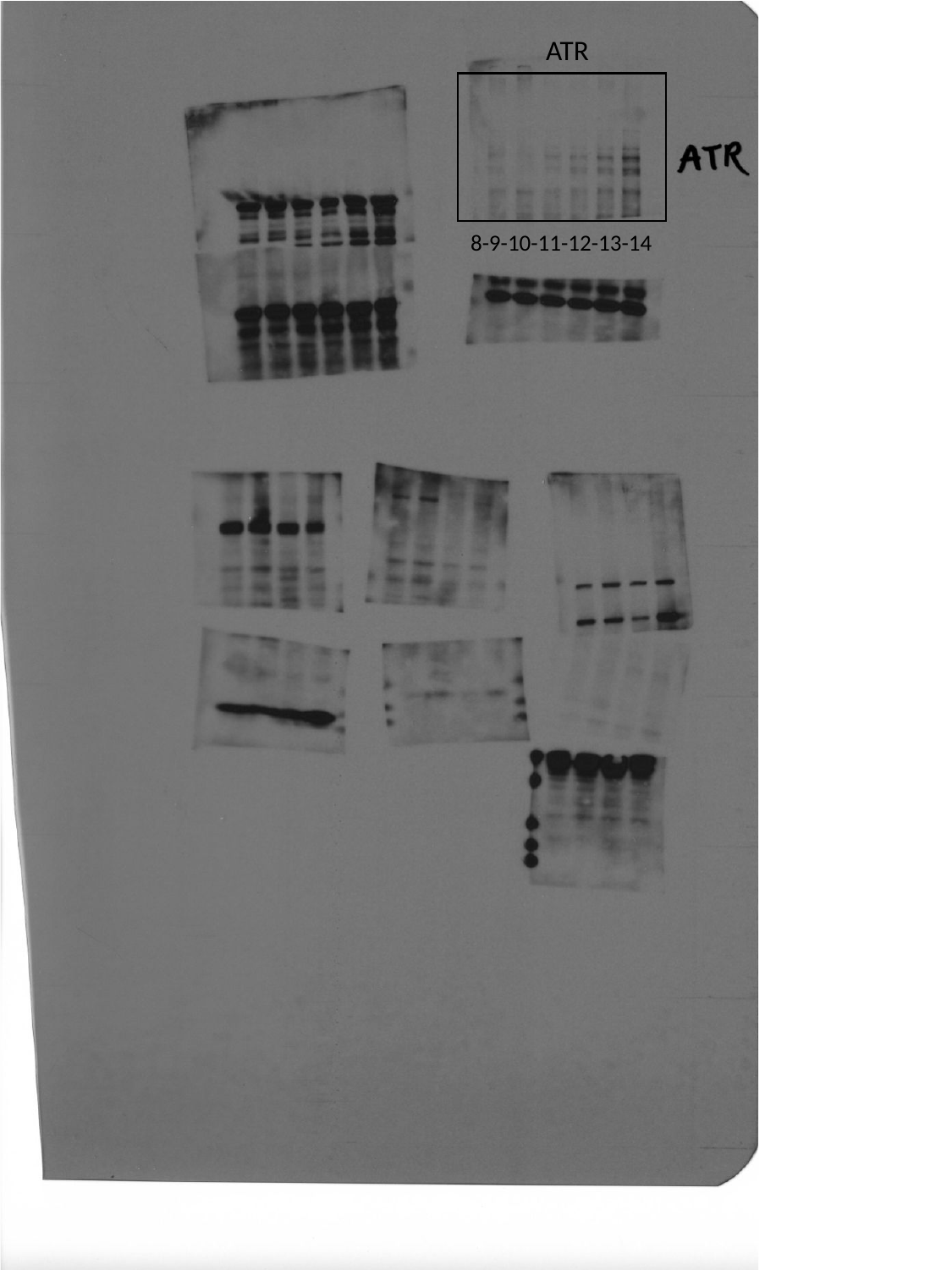

ATR
8-9-10-11-12-13-14

## Slide 24
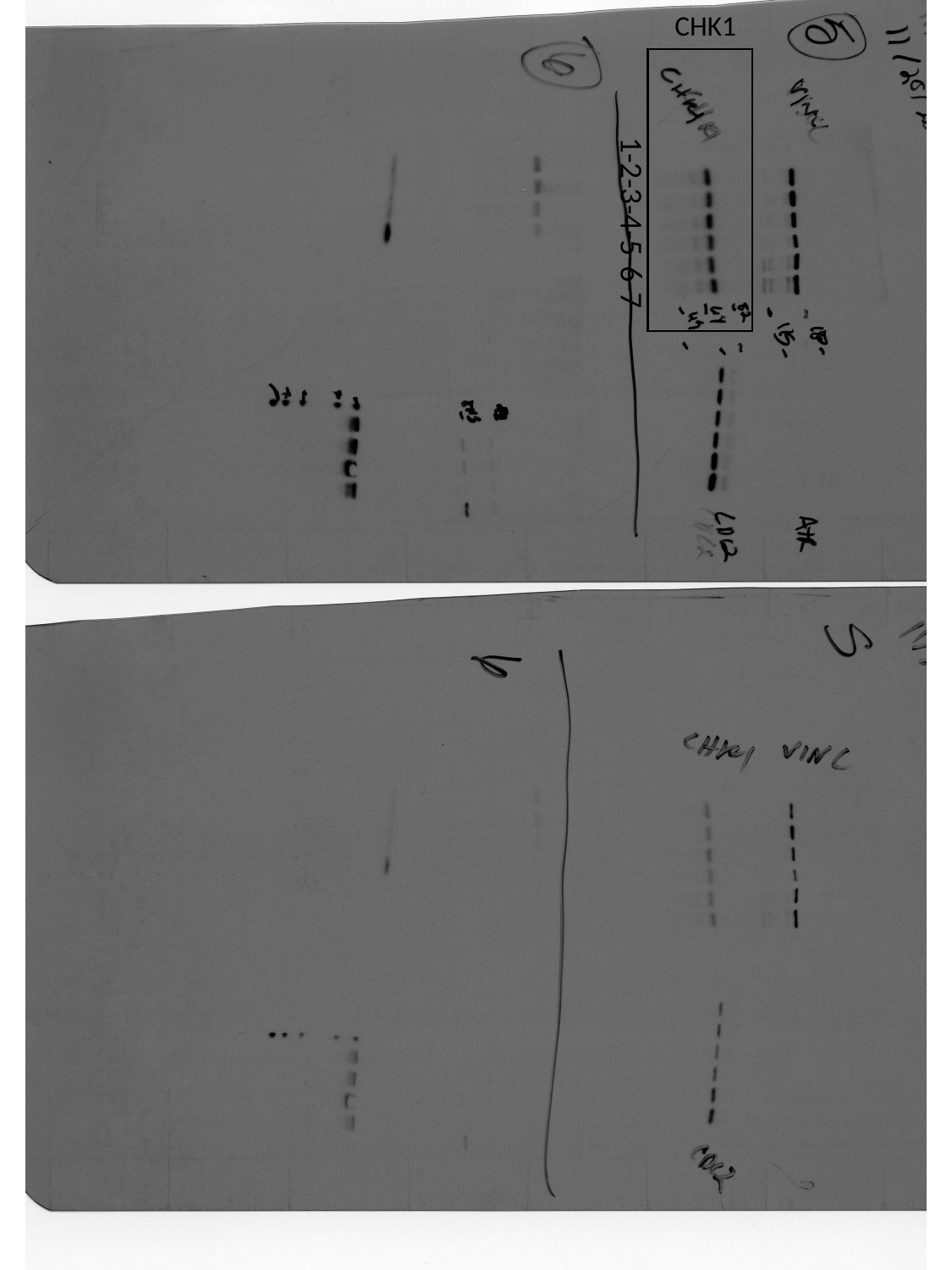

CHK1
1-2-3-4-5-6-7

## Slide 25
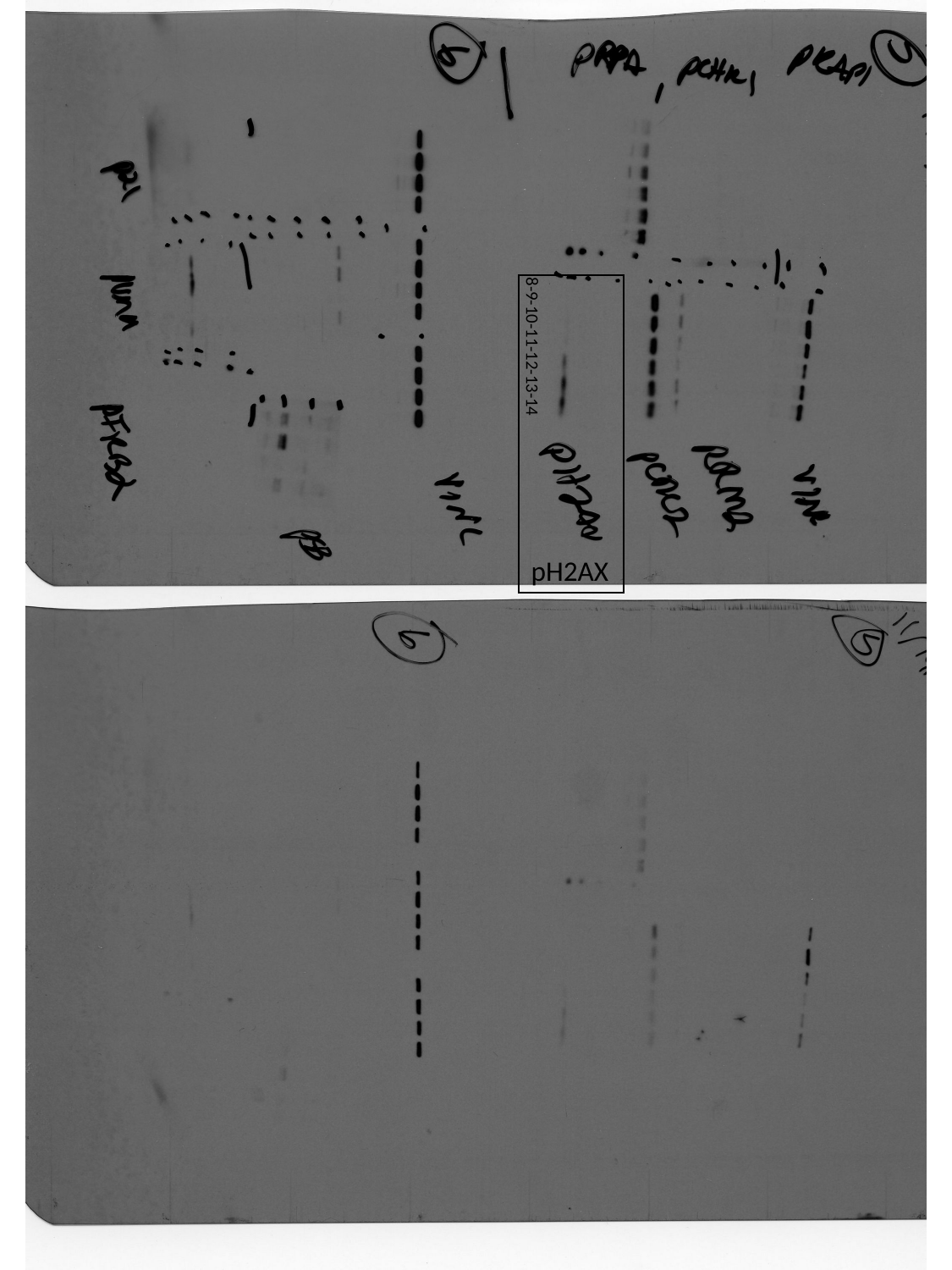

8-9-10-11-12-13-14
pH2AX

## Slide 26
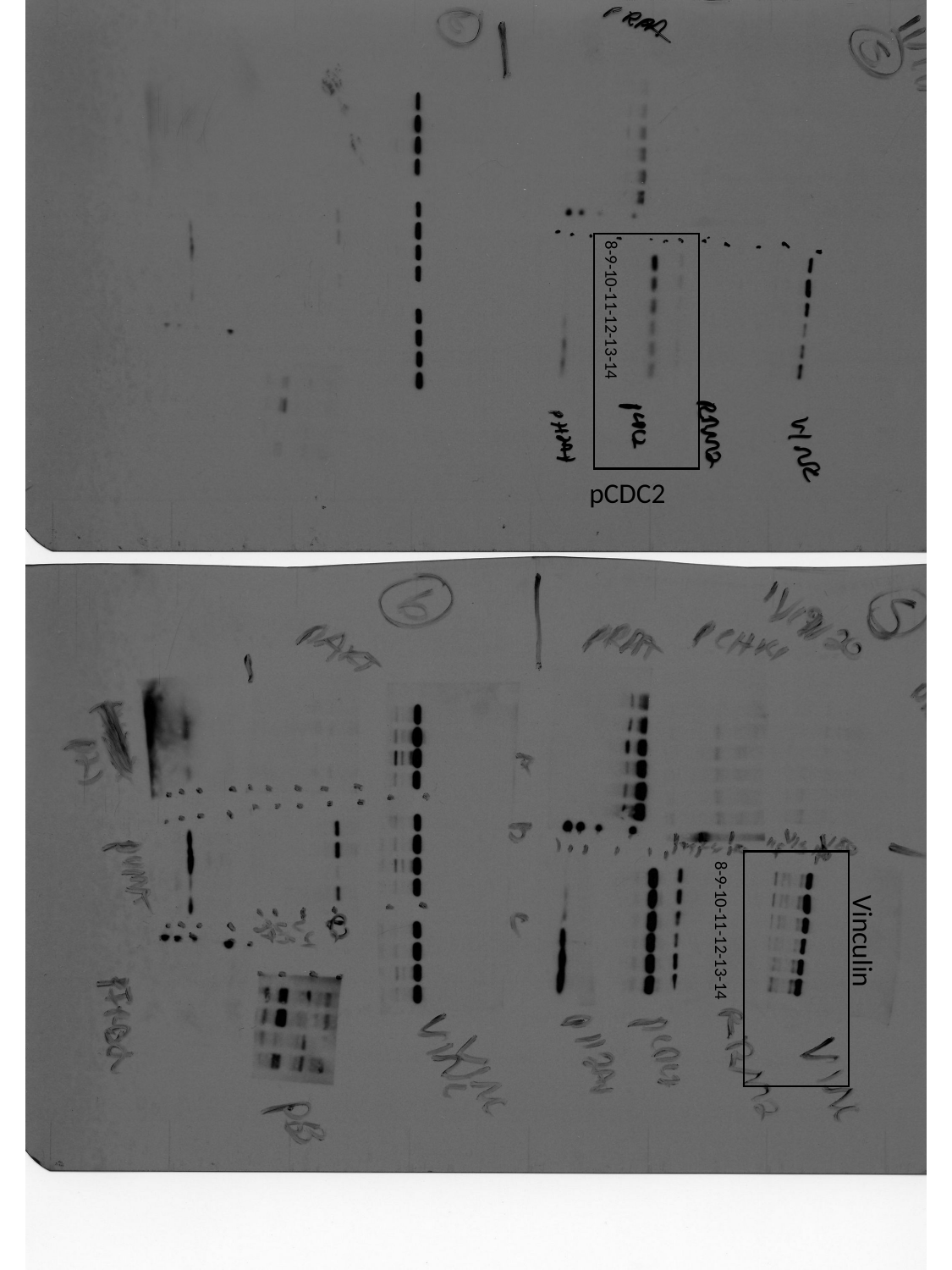

8-9-10-11-12-13-14
pCDC2
Vinculin
8-9-10-11-12-13-14

## Slide 27
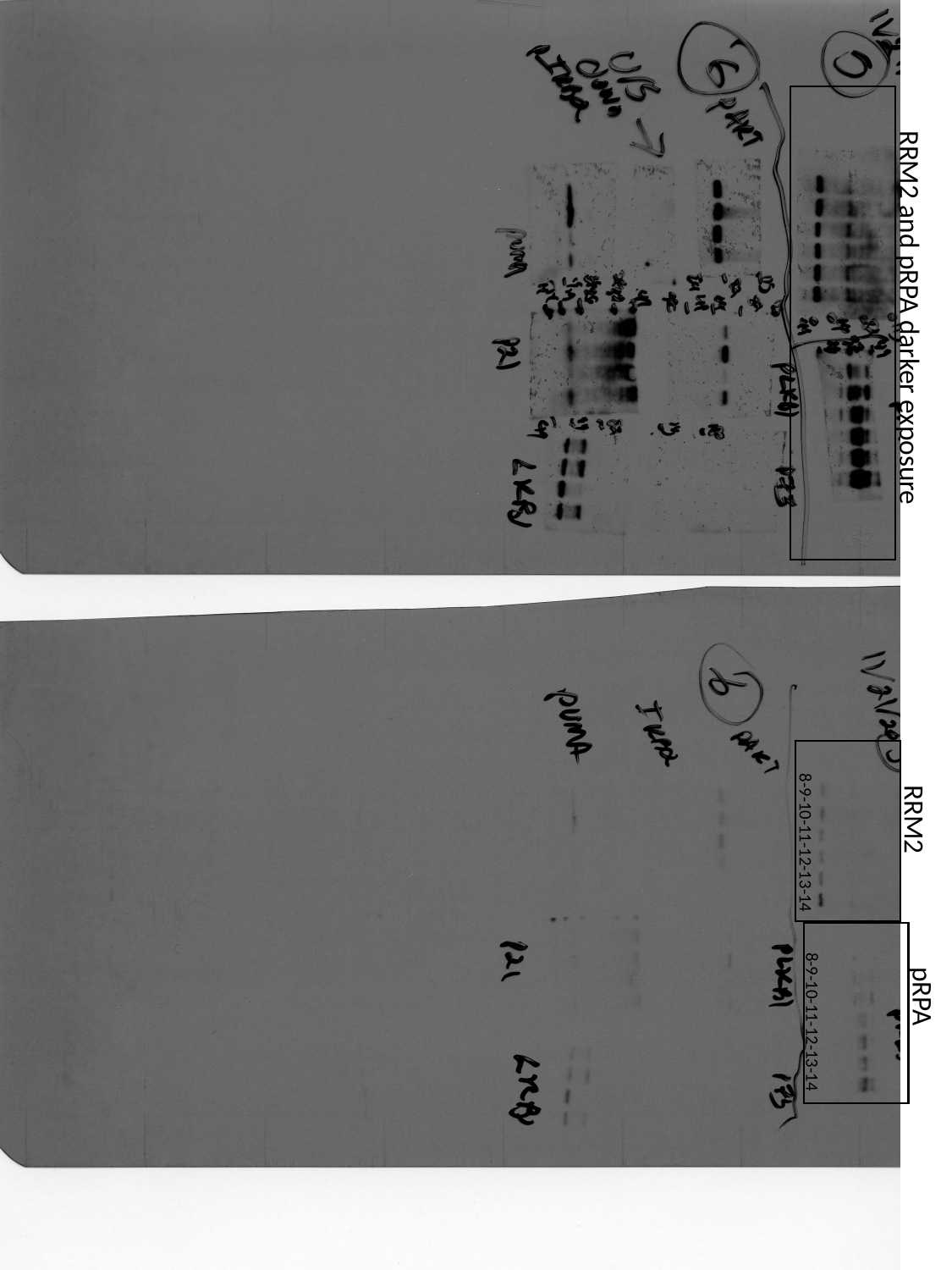

RRM2 and pRPA darker exposure
RRM2
8-9-10-11-12-13-14
8-9-10-11-12-13-14
pRPA

## Slide 28
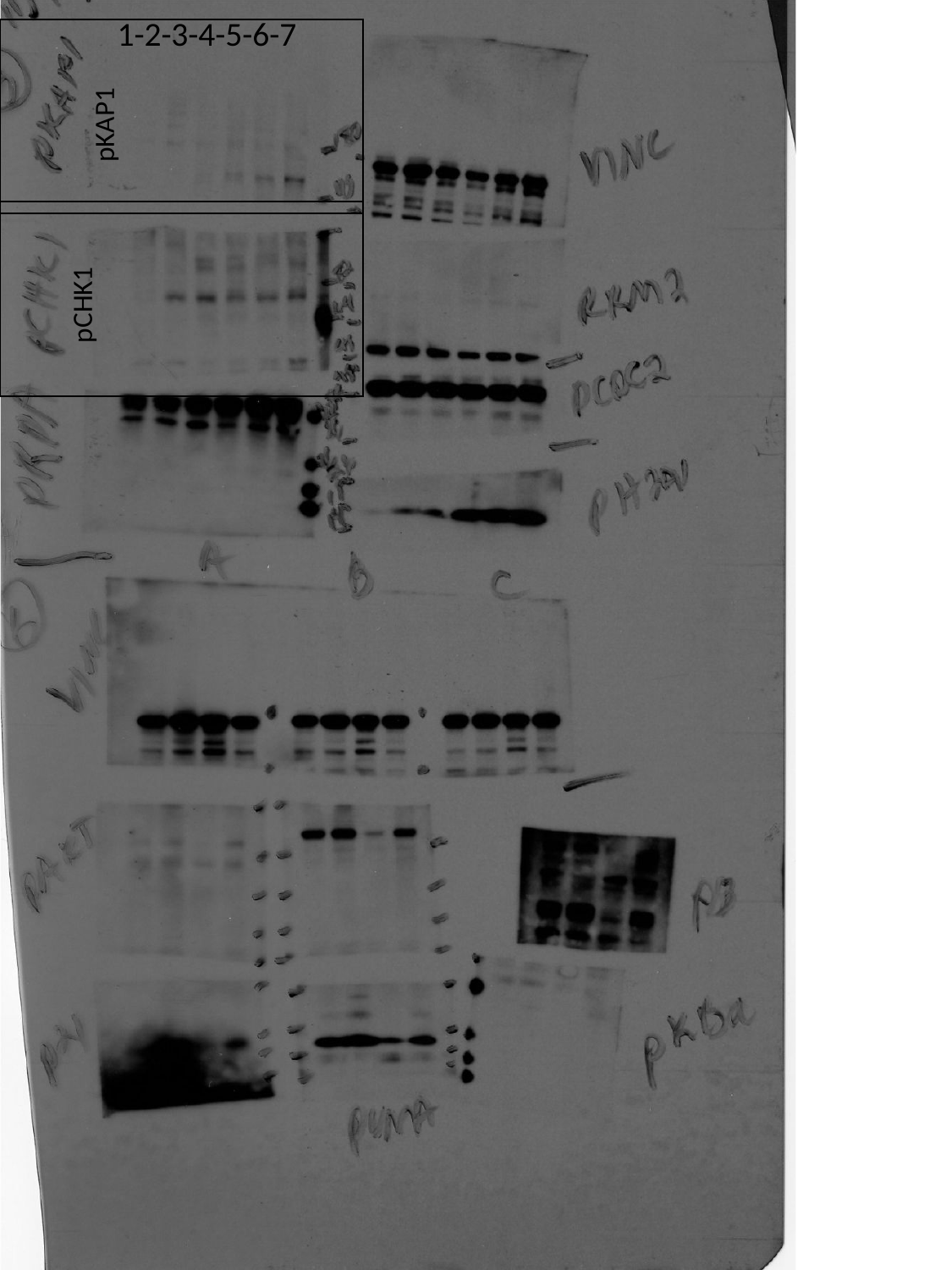

1-2-3-4-5-6-7
pKAP1
pCHK1
