# Supplementary material for: Enhanced Efficacy of Aurora Kinase Inhibitors in G2/M Checkpoint Deficient TP53 Mutant Uterine Carcinomas Is Linked to the Summation of LKB1–AKT–p53 Interactions
Source: Cancers (Basel). 2021 May 3;13(9):2195. doi: 10.3390/cancers13092195 (PMC8125555; doi:10.3390/cancers13092195)
Supplement: Supplementary file 1 [file cancers-13-02195-s001.zip › Lynch and Hill Supplementary Matierals/original blot/Figure S4C.pptx]

## Slide 1
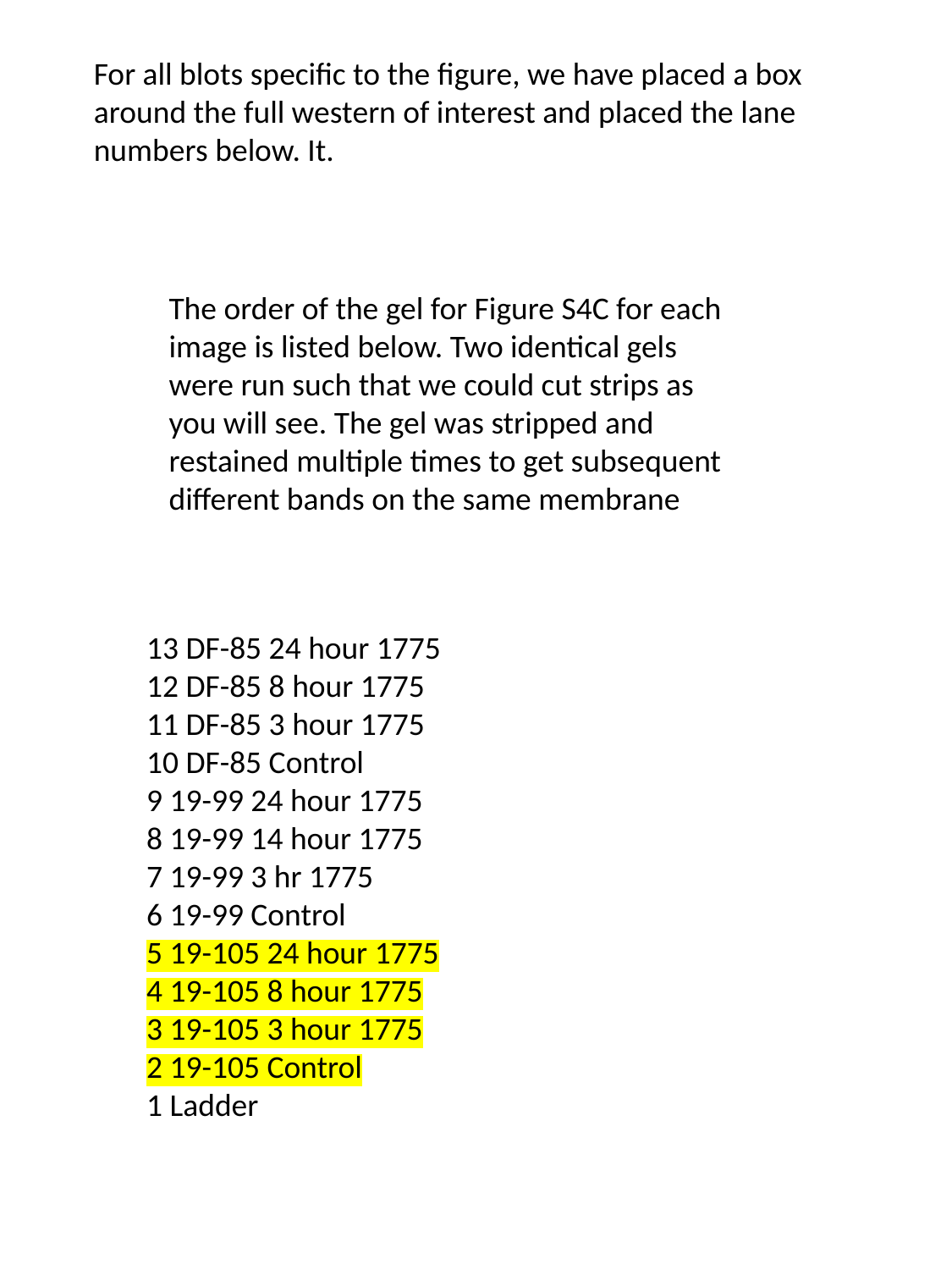

For all blots specific to the figure, we have placed a box around the full western of interest and placed the lane numbers below. It.
The order of the gel for Figure S4C for each image is listed below. Two identical gels were run such that we could cut strips as you will see. The gel was stripped and restained multiple times to get subsequent different bands on the same membrane
13 DF-85 24 hour 1775
12 DF-85 8 hour 1775
11 DF-85 3 hour 1775
10 DF-85 Control
9 19-99 24 hour 1775
8 19-99 14 hour 1775
7 19-99 3 hr 1775
6 19-99 Control
5 19-105 24 hour 1775
4 19-105 8 hour 1775
3 19-105 3 hour 1775
2 19-105 Control
1 Ladder

## Slide 2
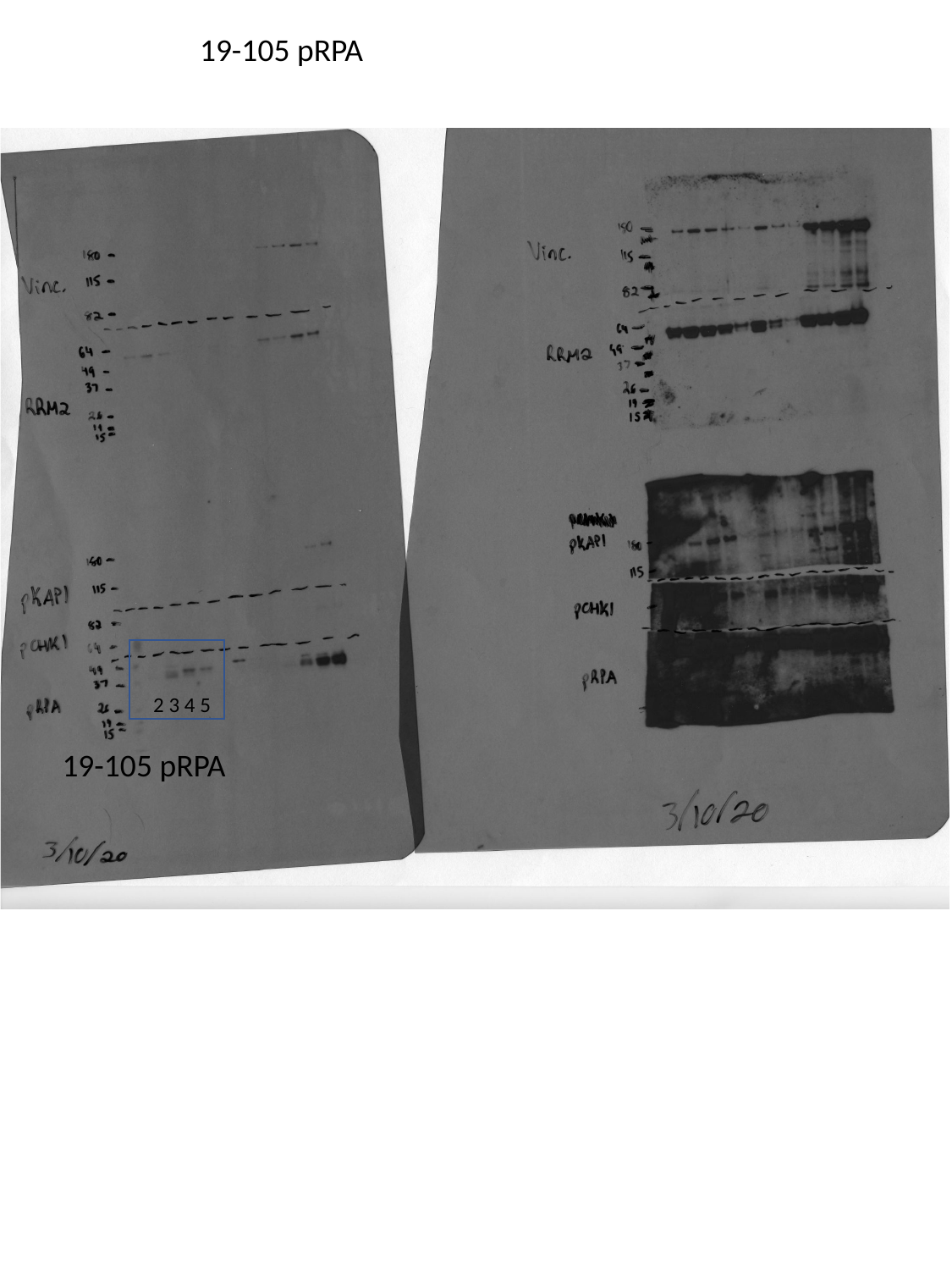

19-105 pRPA
2 3 4 5
19-105 pRPA

## Slide 3
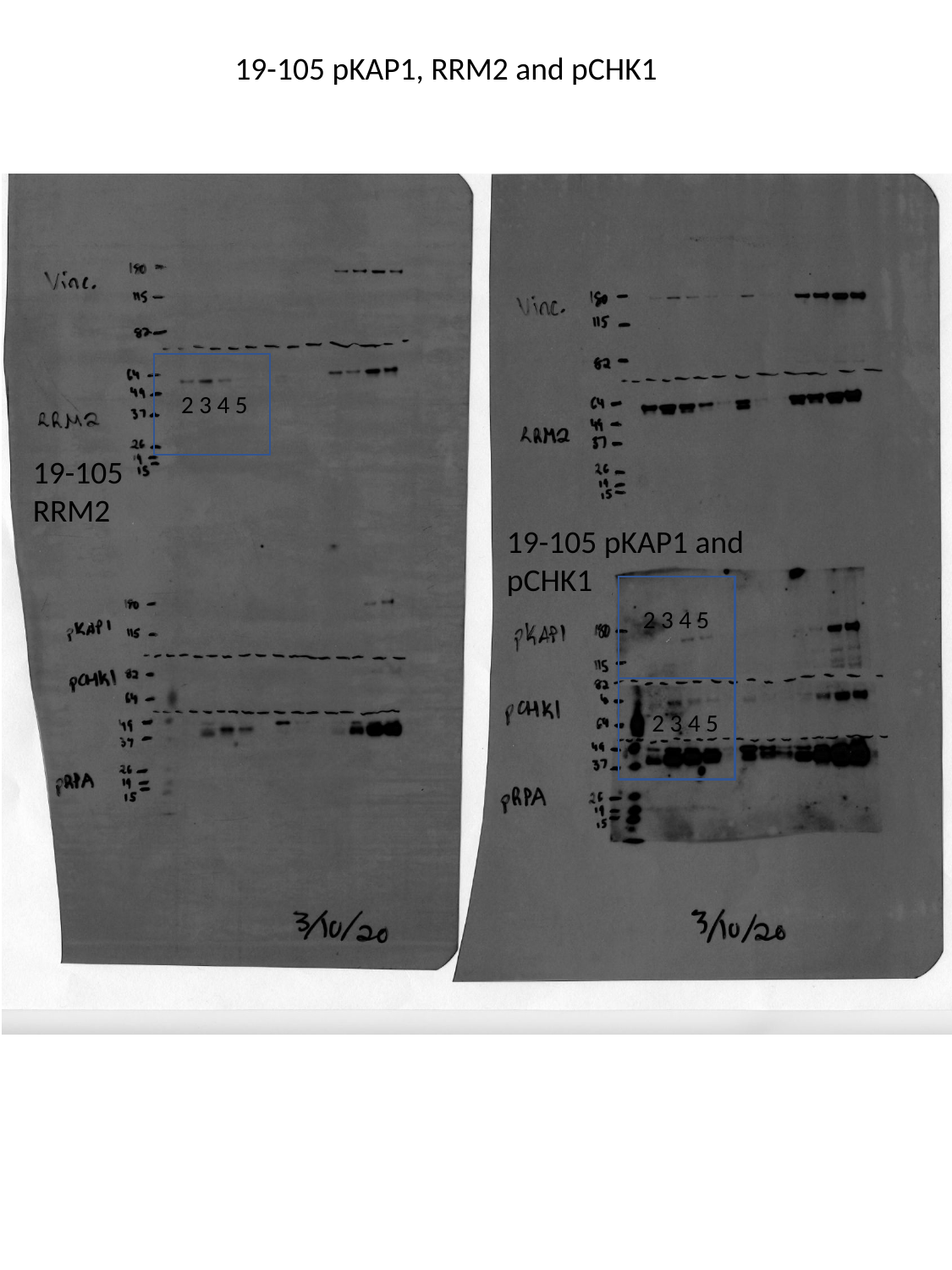

19-105 pKAP1, RRM2 and pCHK1
2 3 4 5
19-105 RRM2
19-105 pKAP1 and pCHK1
2 3 4 5
2 3 4 5

## Slide 4
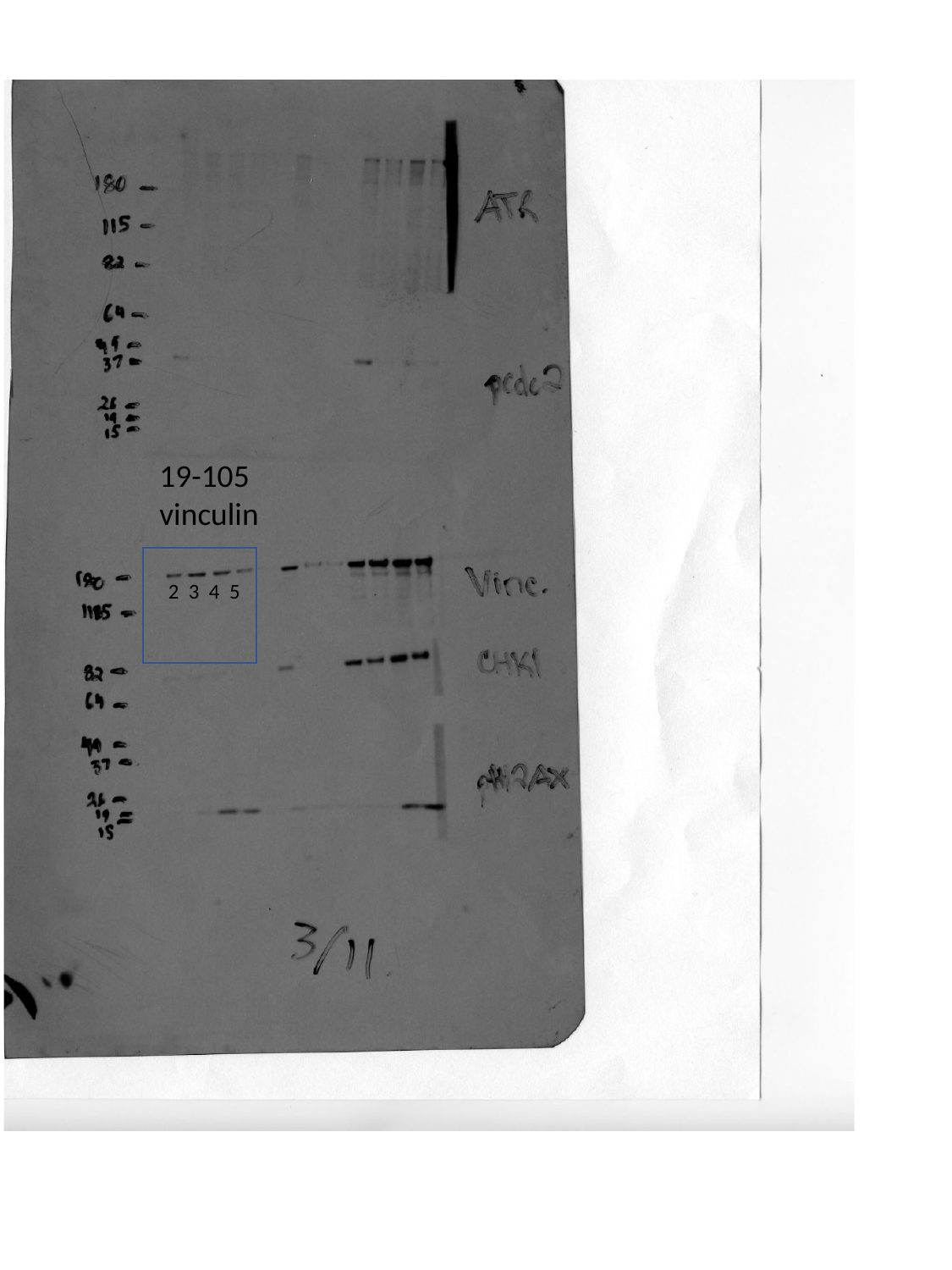

19-105 vinculin
2 3 4 5

## Slide 5
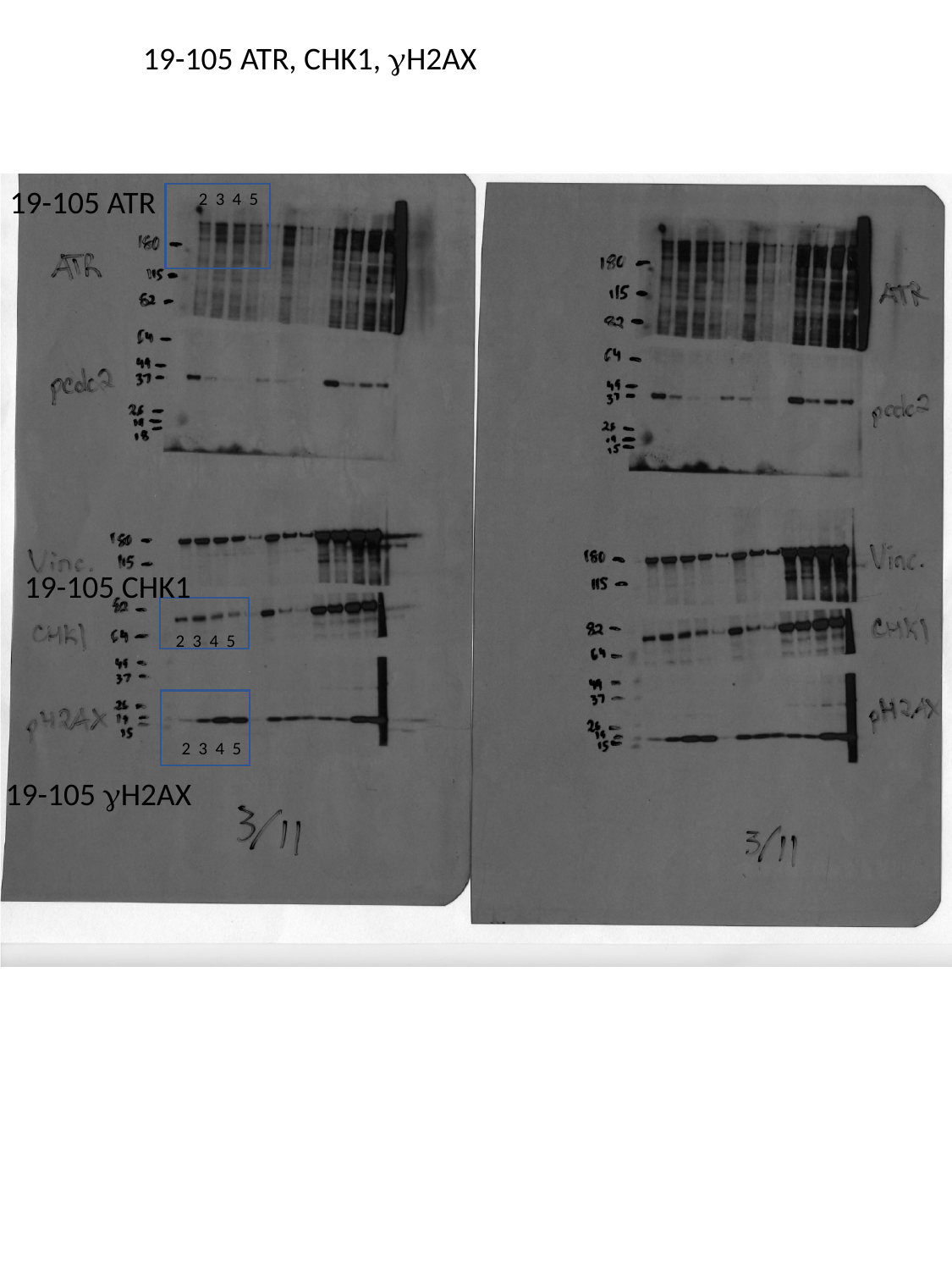

19-105 ATR, CHK1, γH2AX
19-105 ATR
2 3 4 5
19-105 CHK1
2 3 4 5
2 3 4 5
19-105 γH2AX

## Slide 6
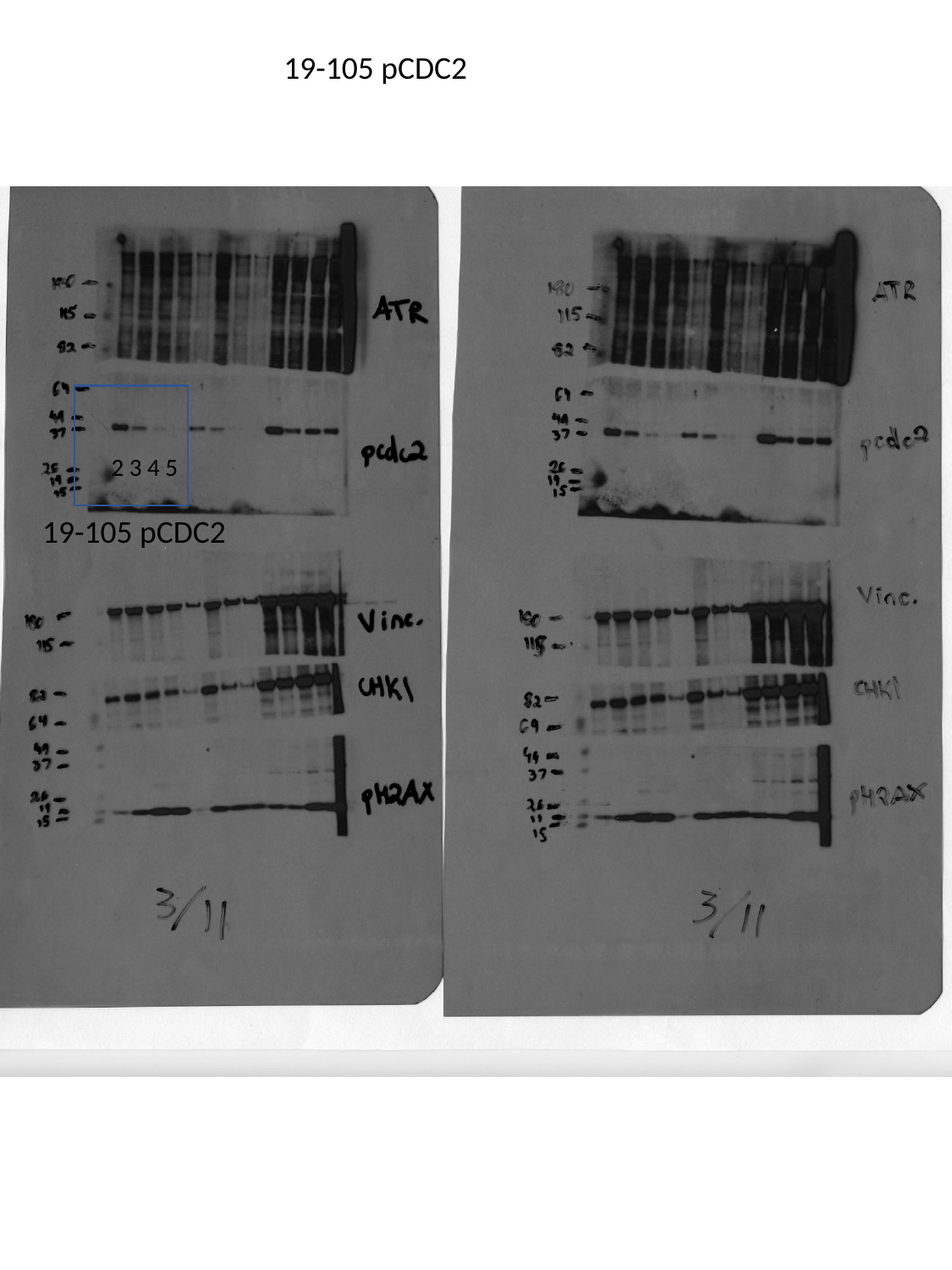

19-105 pCDC2
2 3 4 5
19-105 pCDC2

## Slide 7
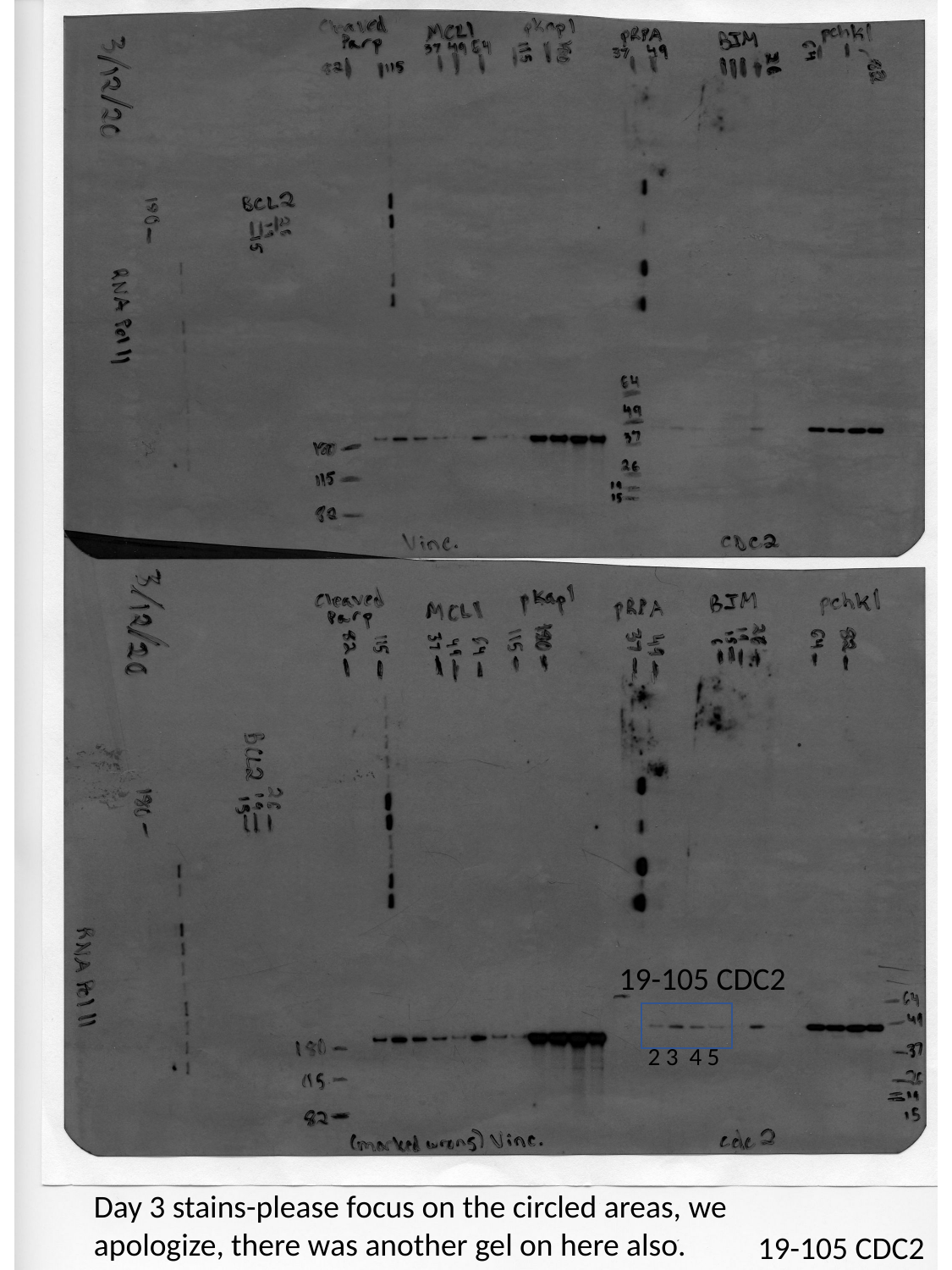

19-105 CDC2
2 3 4 5
Day 3 stains-please focus on the circled areas, we apologize, there was another gel on here also.
19-105 CDC2

## Slide 8
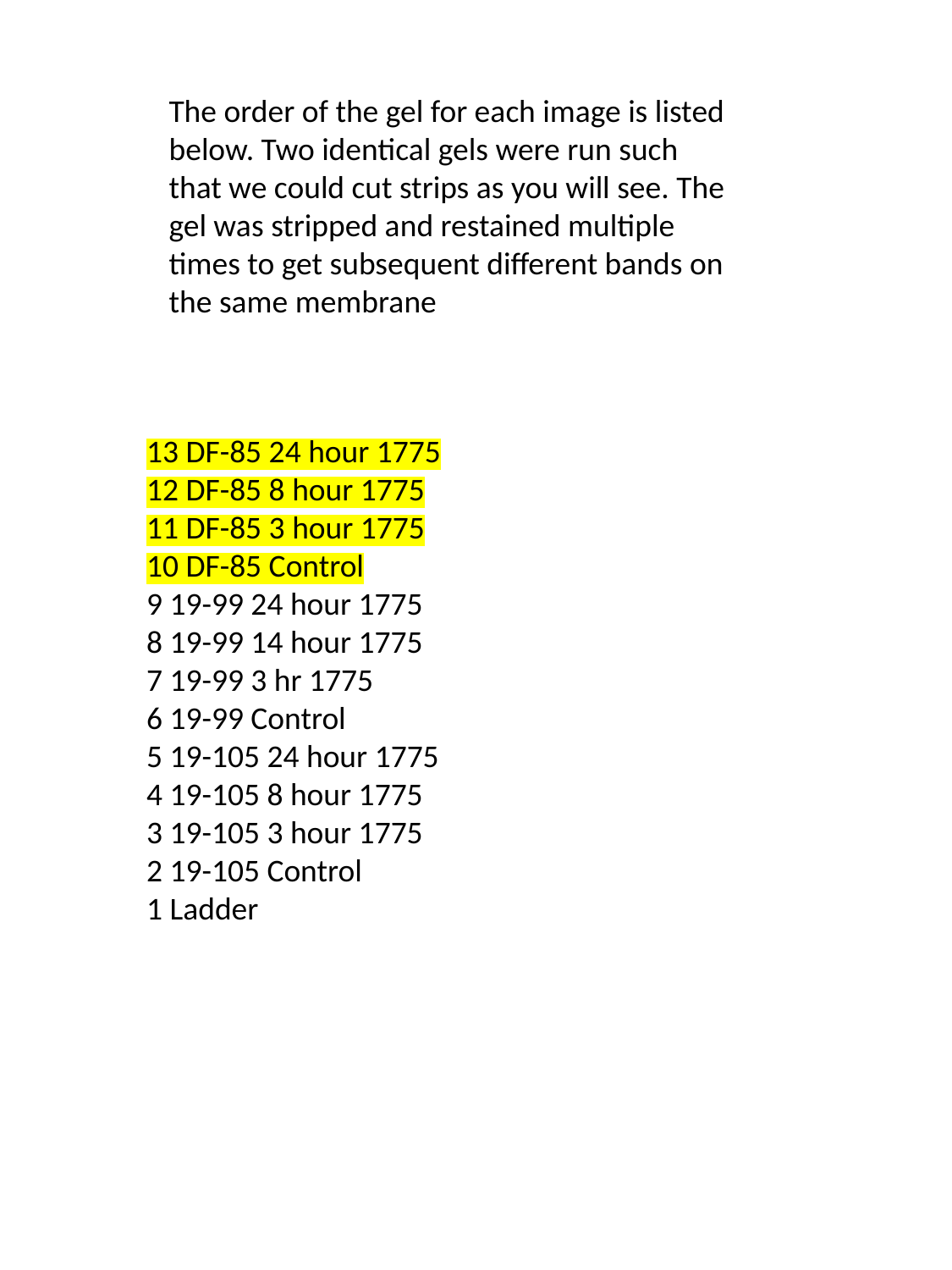

The order of the gel for each image is listed below. Two identical gels were run such that we could cut strips as you will see. The gel was stripped and restained multiple times to get subsequent different bands on the same membrane
13 DF-85 24 hour 1775
12 DF-85 8 hour 1775
11 DF-85 3 hour 1775
10 DF-85 Control
9 19-99 24 hour 1775
8 19-99 14 hour 1775
7 19-99 3 hr 1775
6 19-99 Control
5 19-105 24 hour 1775
4 19-105 8 hour 1775
3 19-105 3 hour 1775
2 19-105 Control
1 Ladder

## Slide 9
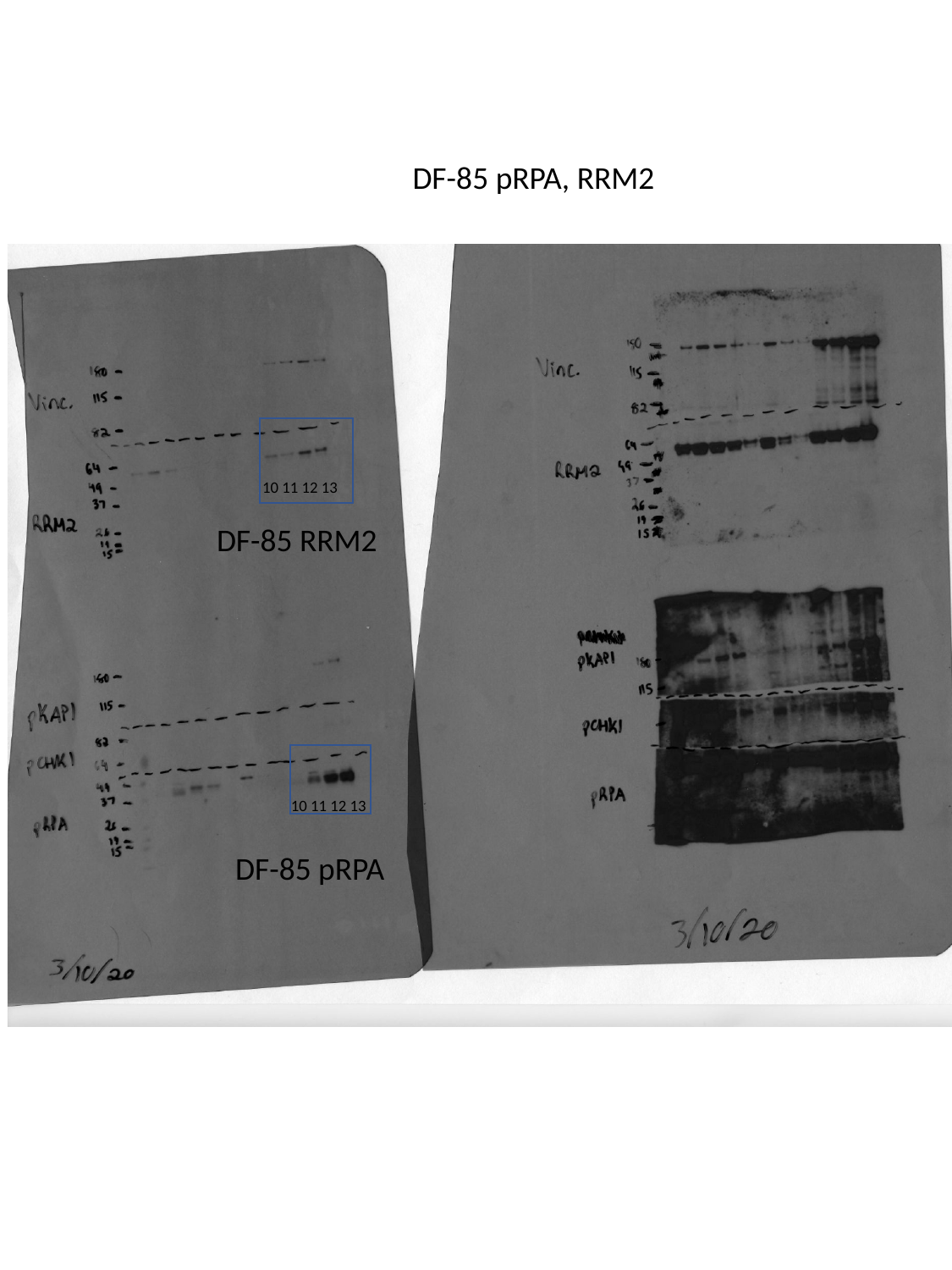

DF-85 pRPA, RRM2
10 11 12 13
DF-85 RRM2
10 11 12 13
DF-85 pRPA

## Slide 10
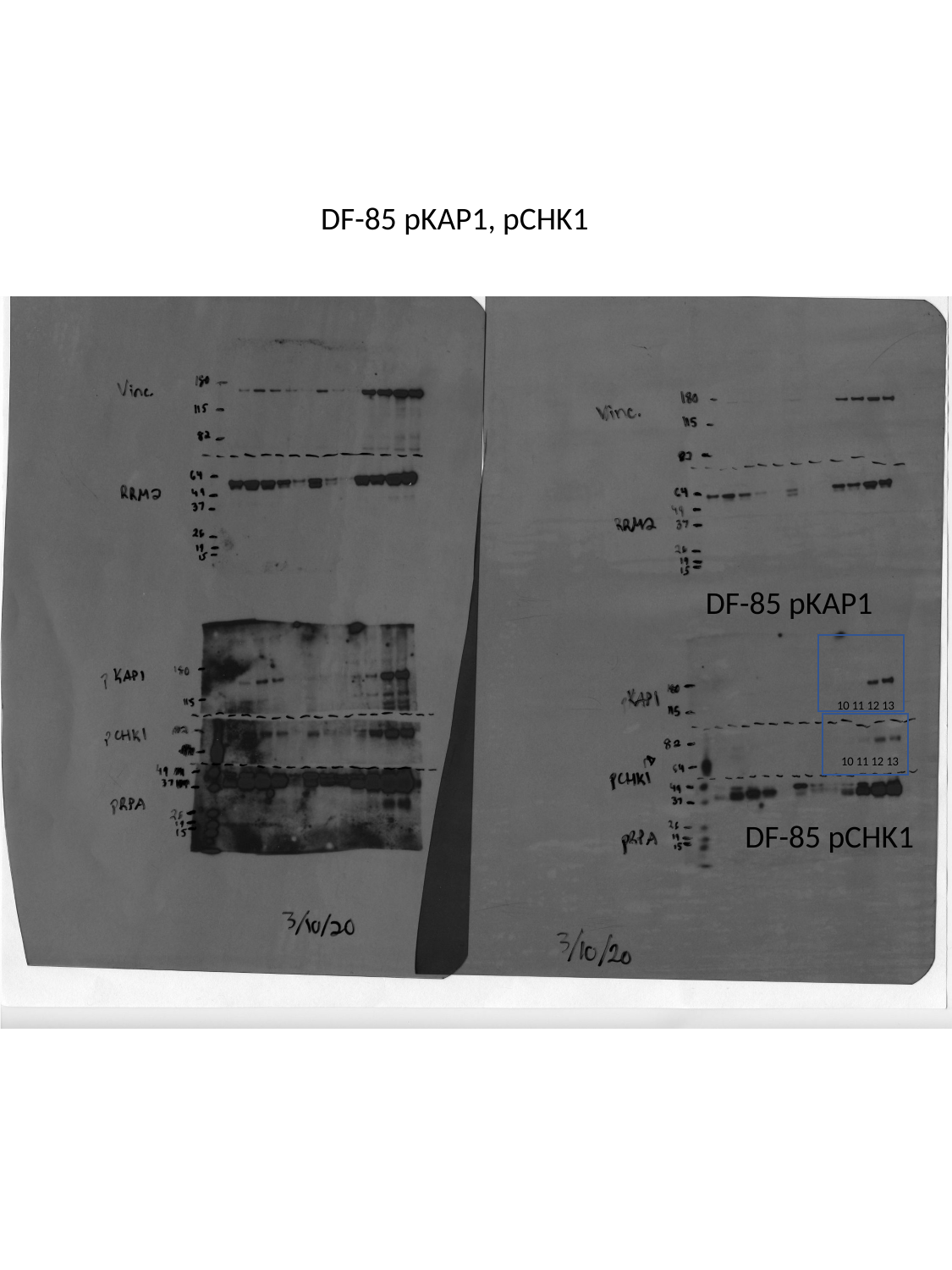

DF-85 pKAP1, pCHK1
DF-85 pKAP1
10 11 12 13
10 11 12 13
DF-85 pCHK1

## Slide 11
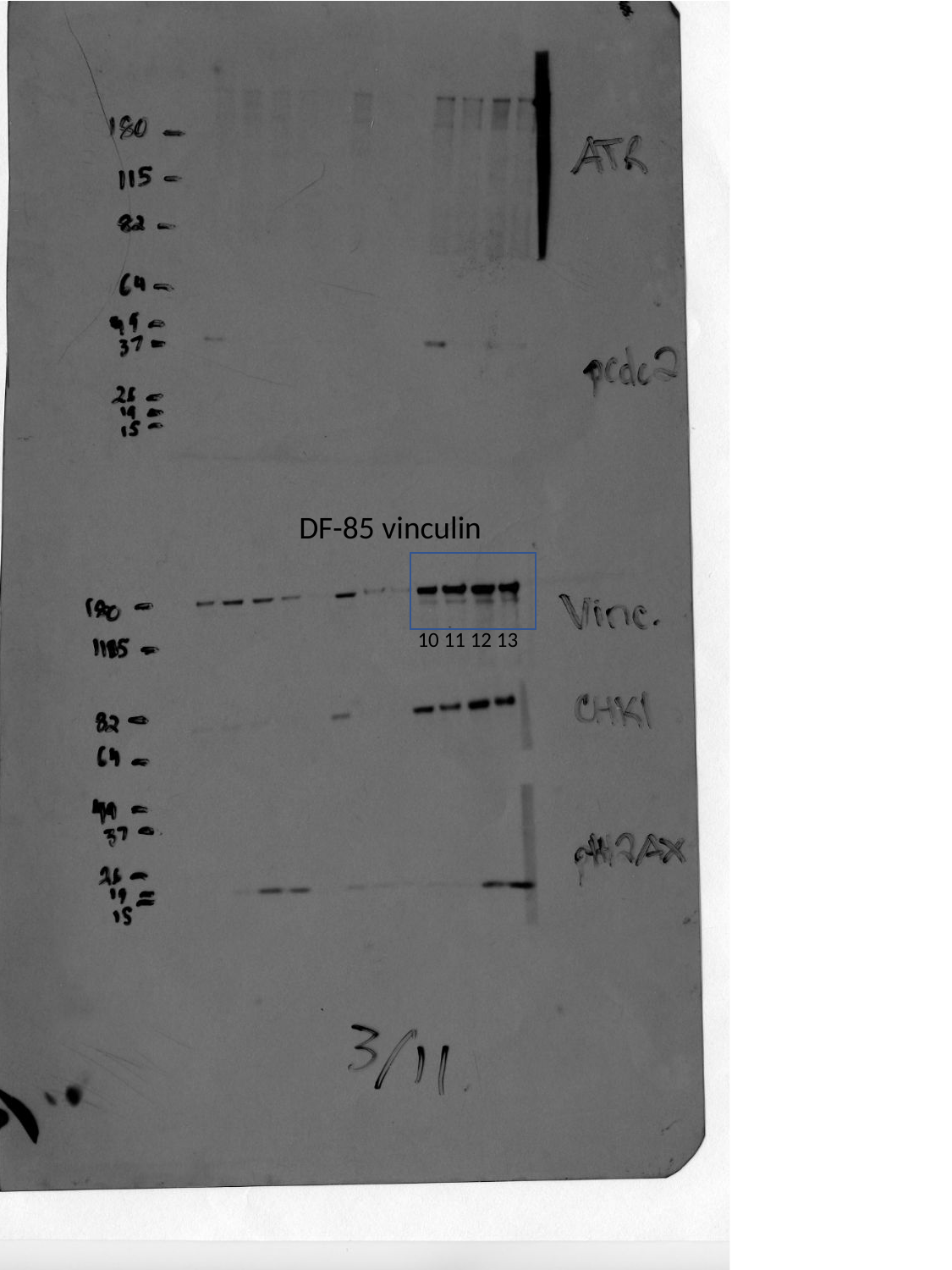

DF-85 vinculin
10 11 12 13

## Slide 12
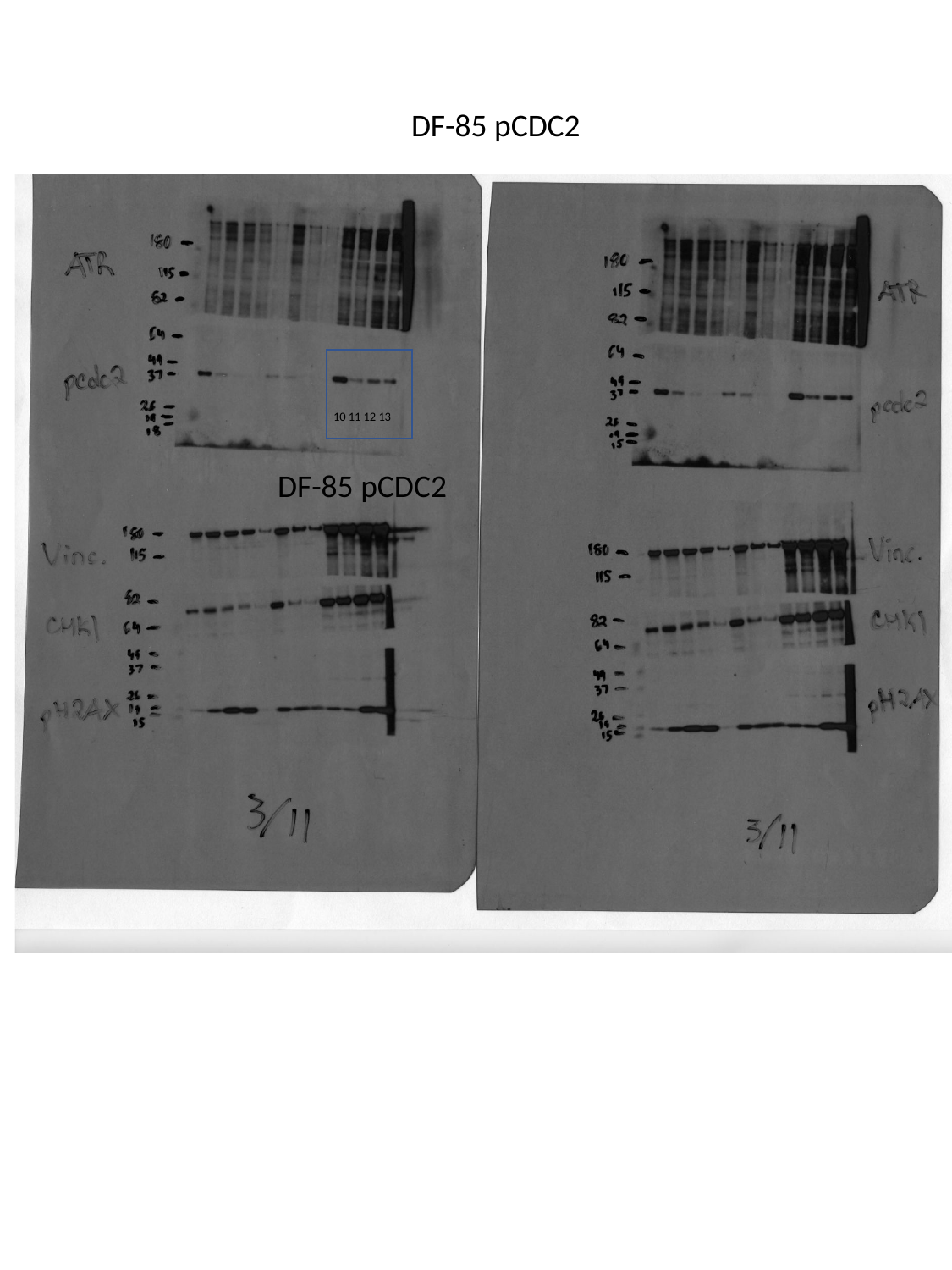

DF-85 pCDC2
10 11 12 13
DF-85 pCDC2

## Slide 13
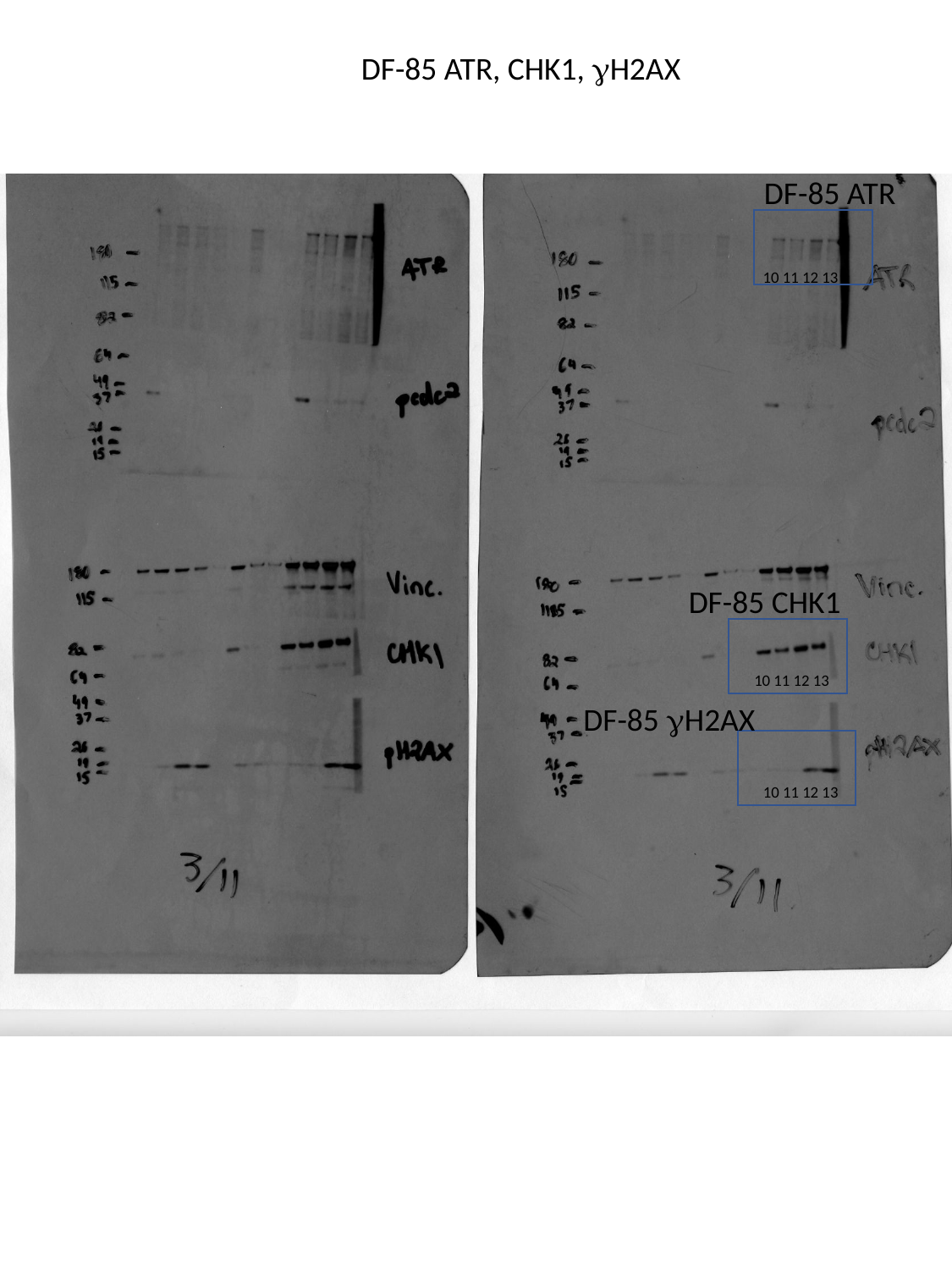

DF-85 ATR, CHK1, γH2AX
DF-85 ATR
10 11 12 13
DF-85 CHK1
10 11 12 13
DF-85 γH2AX
10 11 12 13

## Slide 14
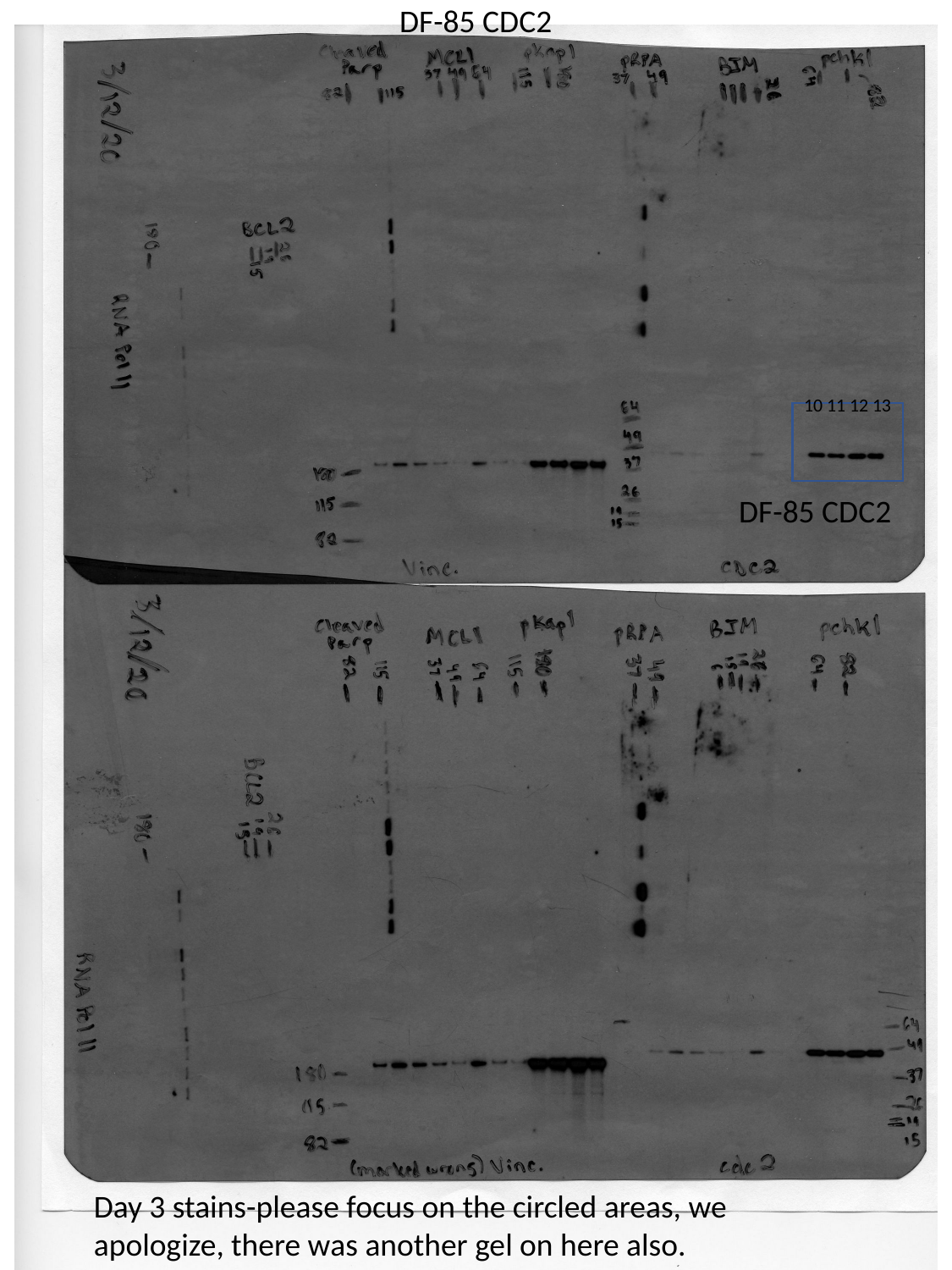

DF-85 CDC2
10 11 12 13
DF-85 CDC2
Day 3 stains-please focus on the circled areas, we apologize, there was another gel on here also.

## Slide 15
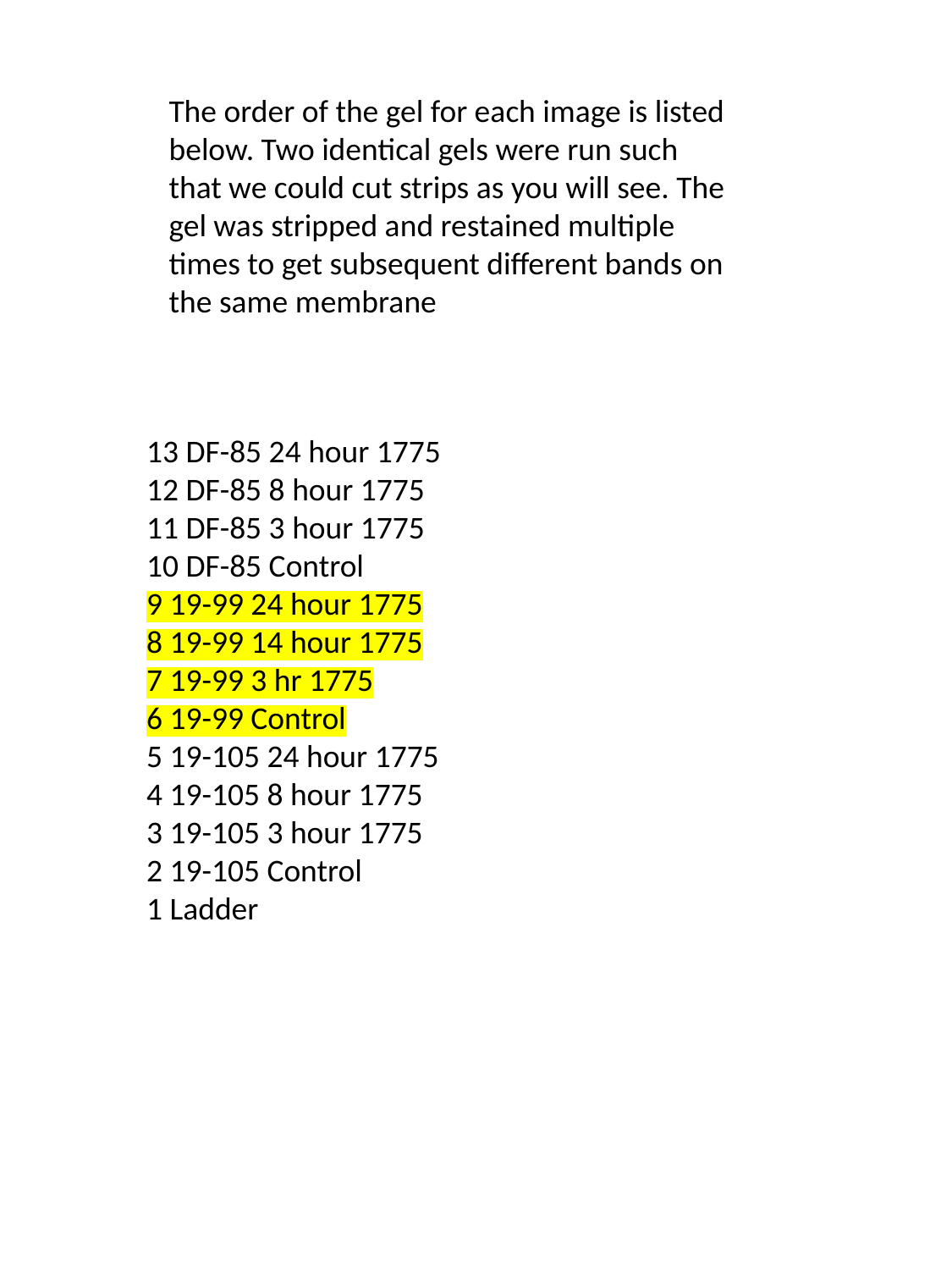

The order of the gel for each image is listed below. Two identical gels were run such that we could cut strips as you will see. The gel was stripped and restained multiple times to get subsequent different bands on the same membrane
13 DF-85 24 hour 1775
12 DF-85 8 hour 1775
11 DF-85 3 hour 1775
10 DF-85 Control
9 19-99 24 hour 1775
8 19-99 14 hour 1775
7 19-99 3 hr 1775
6 19-99 Control
5 19-105 24 hour 1775
4 19-105 8 hour 1775
3 19-105 3 hour 1775
2 19-105 Control
1 Ladder

## Slide 16
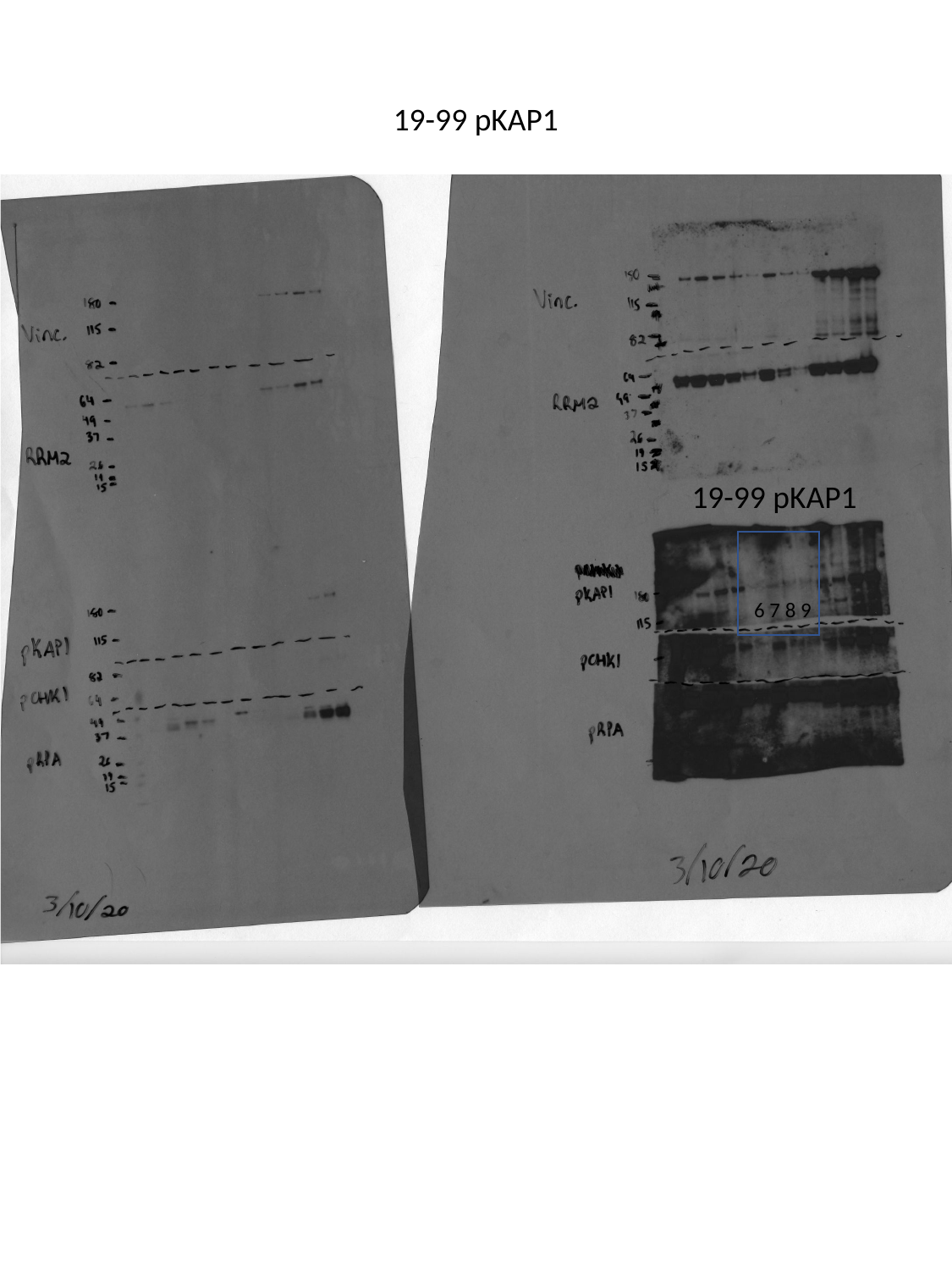

19-99 pKAP1
19-99 pKAP1
6 7 8 9

## Slide 17
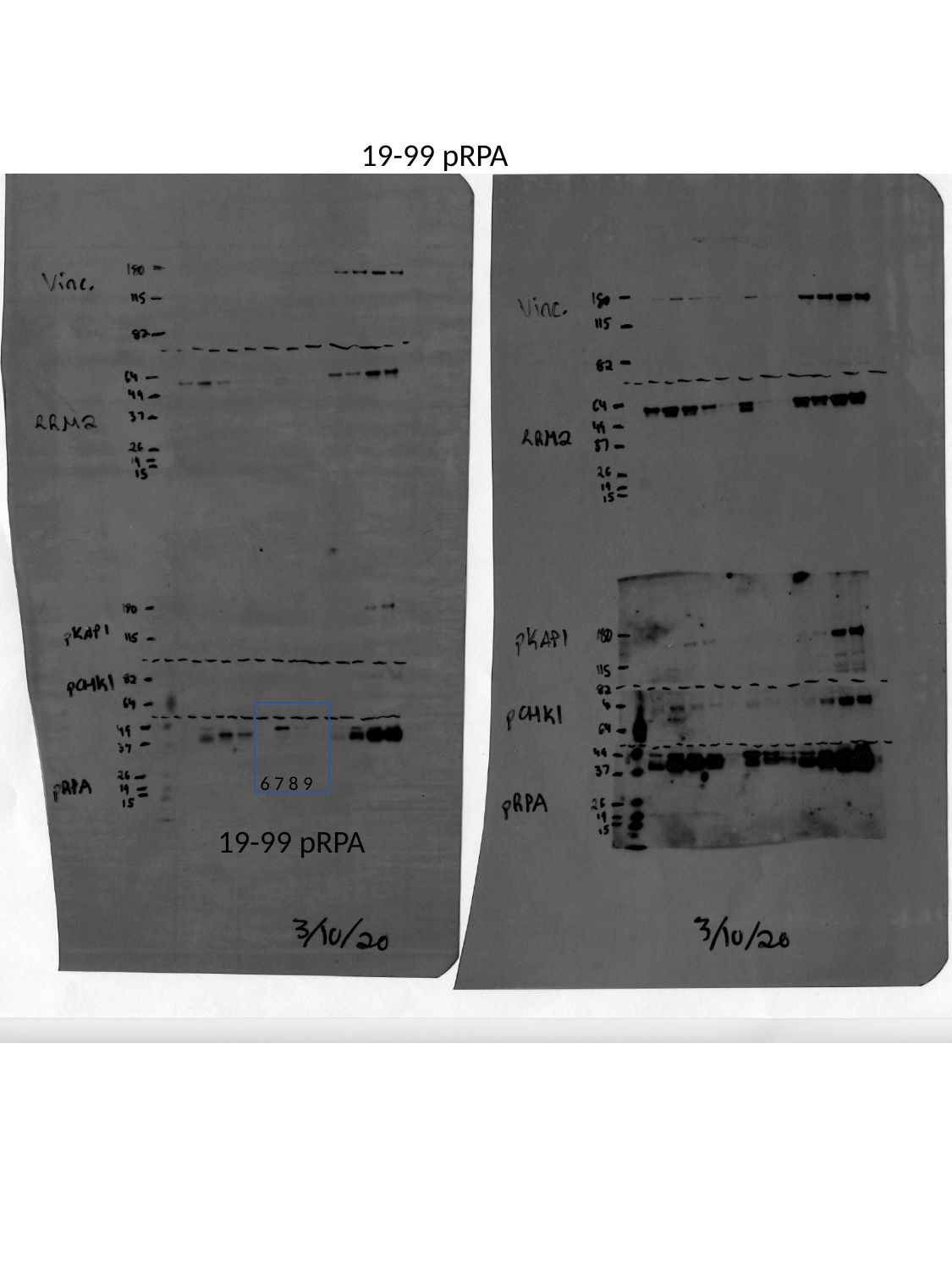

19-99 pRPA
6 7 8 9
19-99 pRPA

## Slide 18
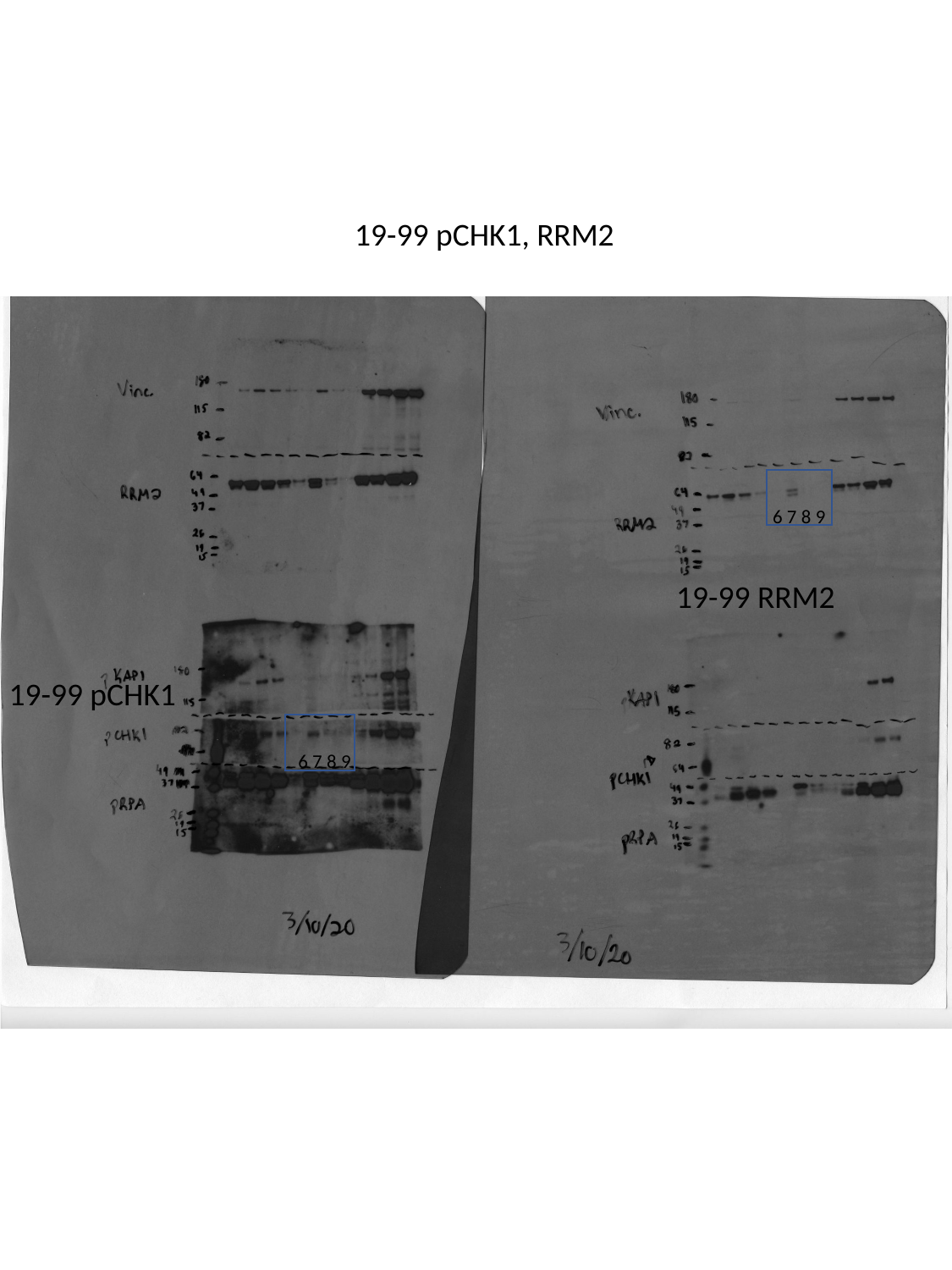

19-99 pCHK1, RRM2
6 7 8 9
19-99 RRM2
19-99 pCHK1
6 7 8 9

## Slide 19
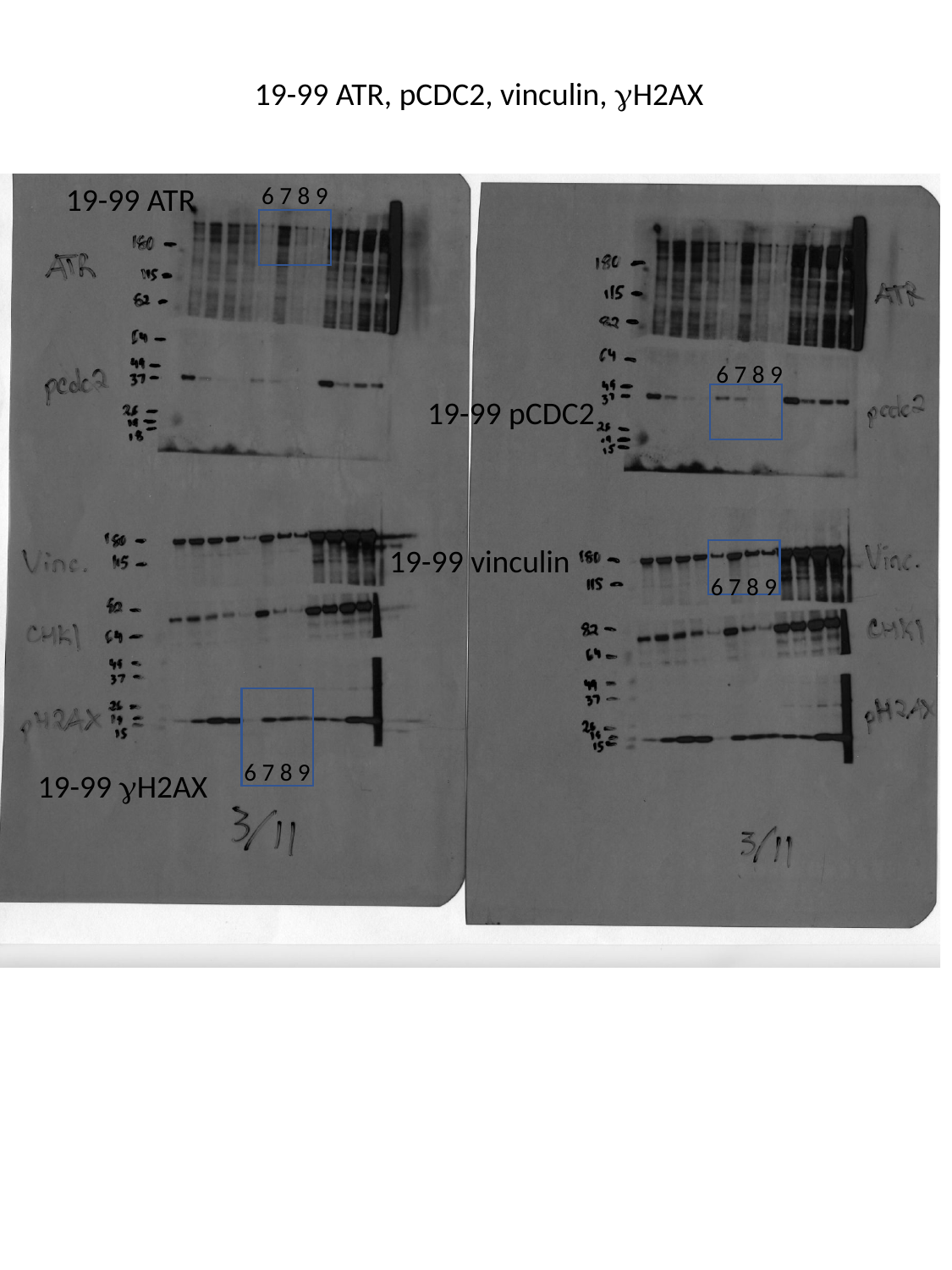

19-99 ATR, pCDC2, vinculin, γH2AX
19-99 ATR
6 7 8 9
6 7 8 9
19-99 pCDC2
19-99 vinculin
6 7 8 9
6 7 8 9
19-99 γH2AX

## Slide 20
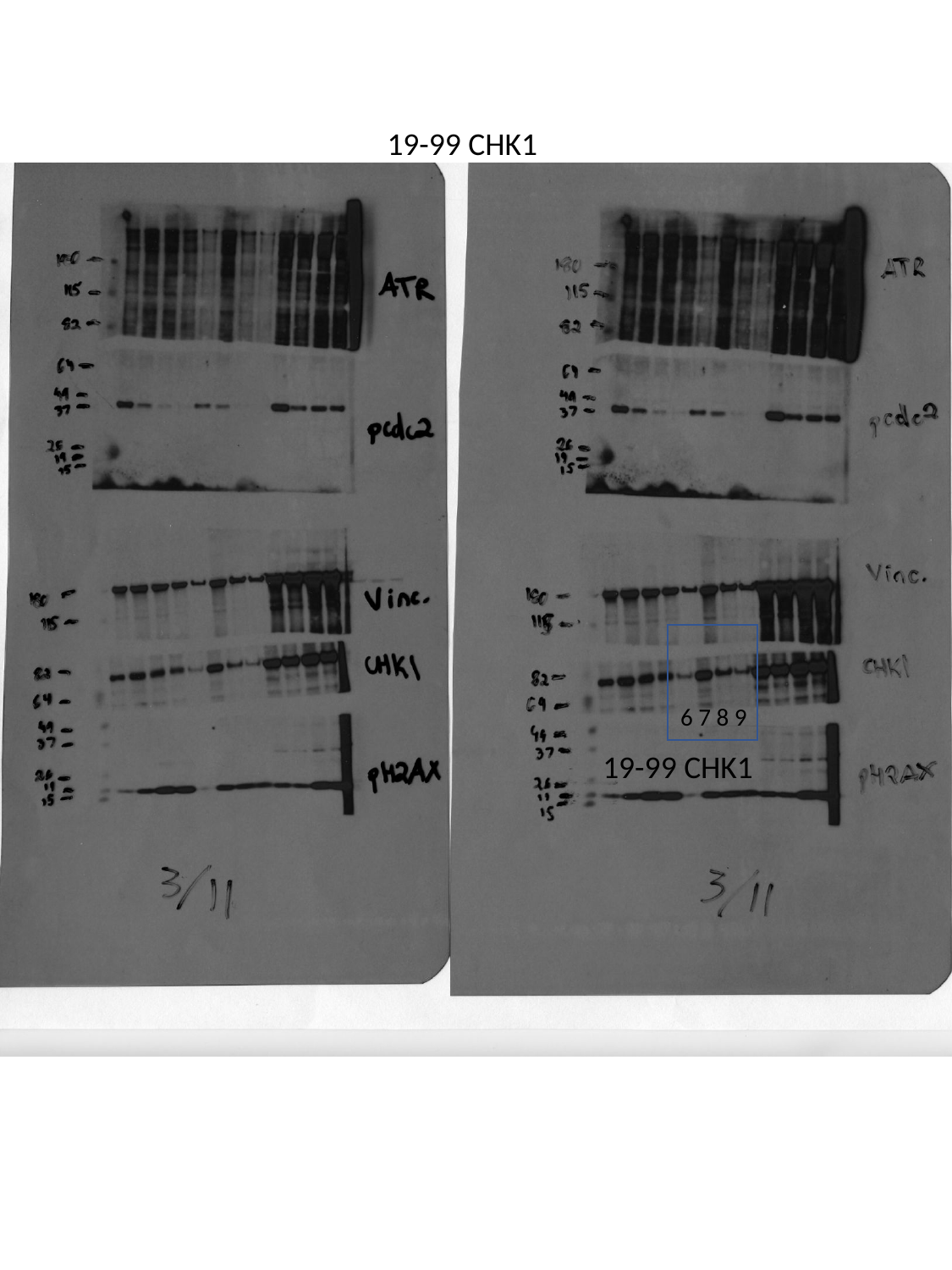

19-99 CHK1
6 7 8 9
19-99 CHK1

## Slide 21
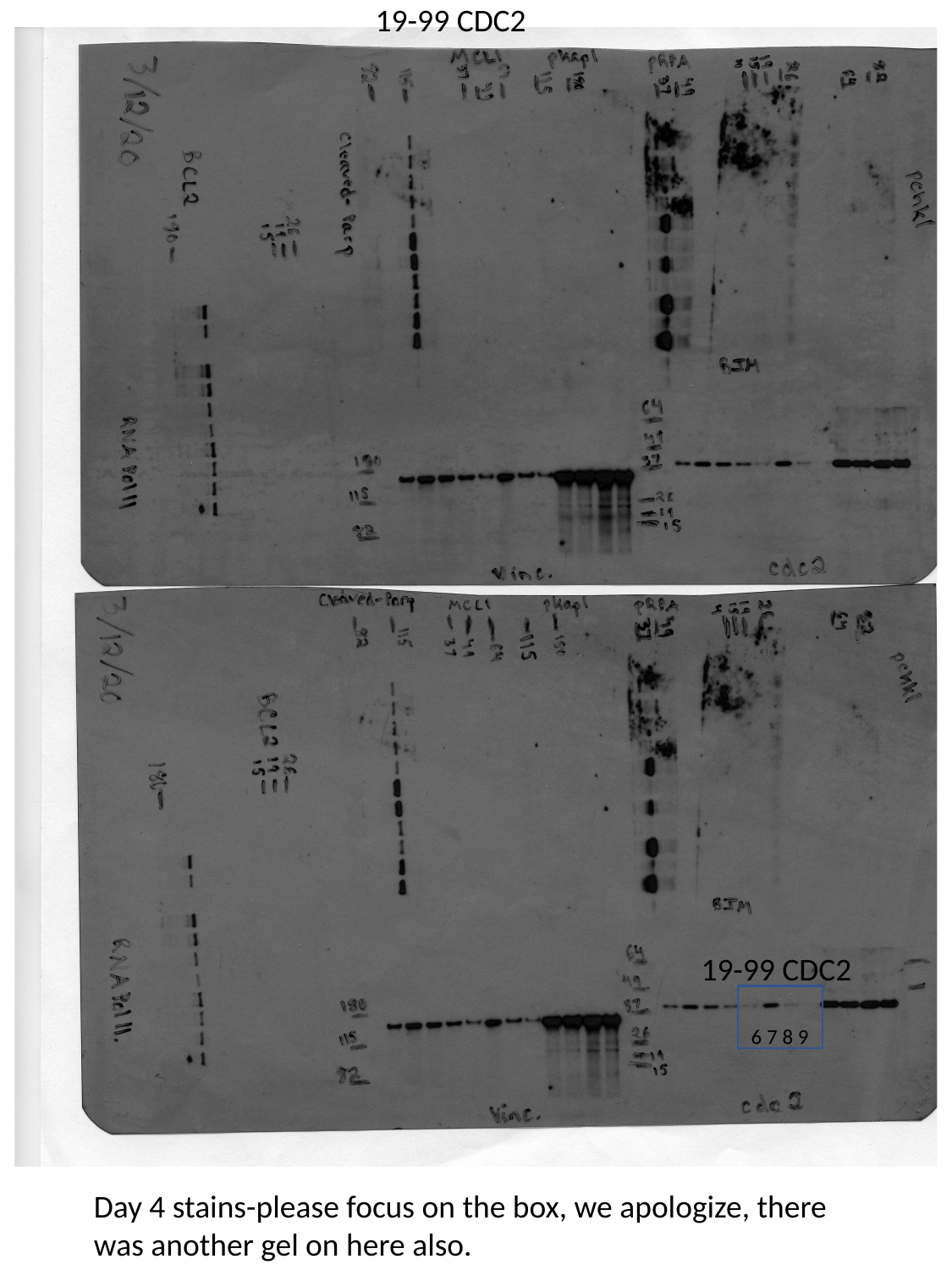

19-99 CDC2
19-99 CDC2
6 7 8 9
Day 4 stains-please focus on the box, we apologize, there was another gel on here also.
