# Supplementary material for: Enhanced Efficacy of Aurora Kinase Inhibitors in G2/M Checkpoint Deficient TP53 Mutant Uterine Carcinomas Is Linked to the Summation of LKB1–AKT–p53 Interactions
Source: Cancers (Basel). 2021 May 3;13(9):2195. doi: 10.3390/cancers13092195 (PMC8125555; doi:10.3390/cancers13092195)
Supplement: Supplementary file 1 [file cancers-13-02195-s001.zip › Lynch and Hill Supplementary Matierals/original blot/Figure S7A.pptx]

## Slide 1
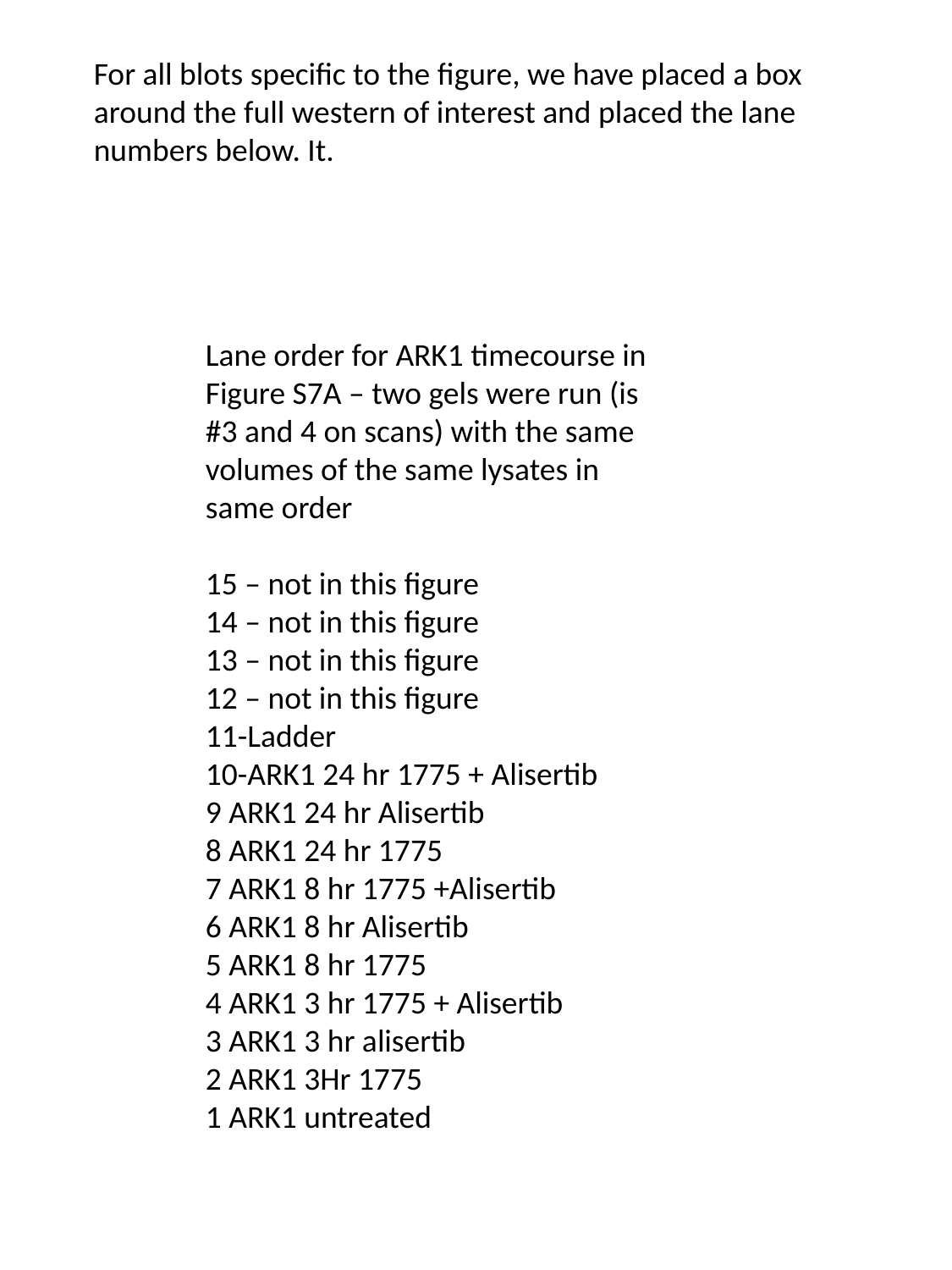

For all blots specific to the figure, we have placed a box around the full western of interest and placed the lane numbers below. It.
Lane order for ARK1 timecourse in Figure S7A – two gels were run (is #3 and 4 on scans) with the same volumes of the same lysates in same order
15 – not in this figure
14 – not in this figure
13 – not in this figure
12 – not in this figure
11-Ladder
10-ARK1 24 hr 1775 + Alisertib
9 ARK1 24 hr Alisertib
8 ARK1 24 hr 1775
7 ARK1 8 hr 1775 +Alisertib
6 ARK1 8 hr Alisertib
5 ARK1 8 hr 1775
4 ARK1 3 hr 1775 + Alisertib
3 ARK1 3 hr alisertib
2 ARK1 3Hr 1775
1 ARK1 untreated

## Slide 2
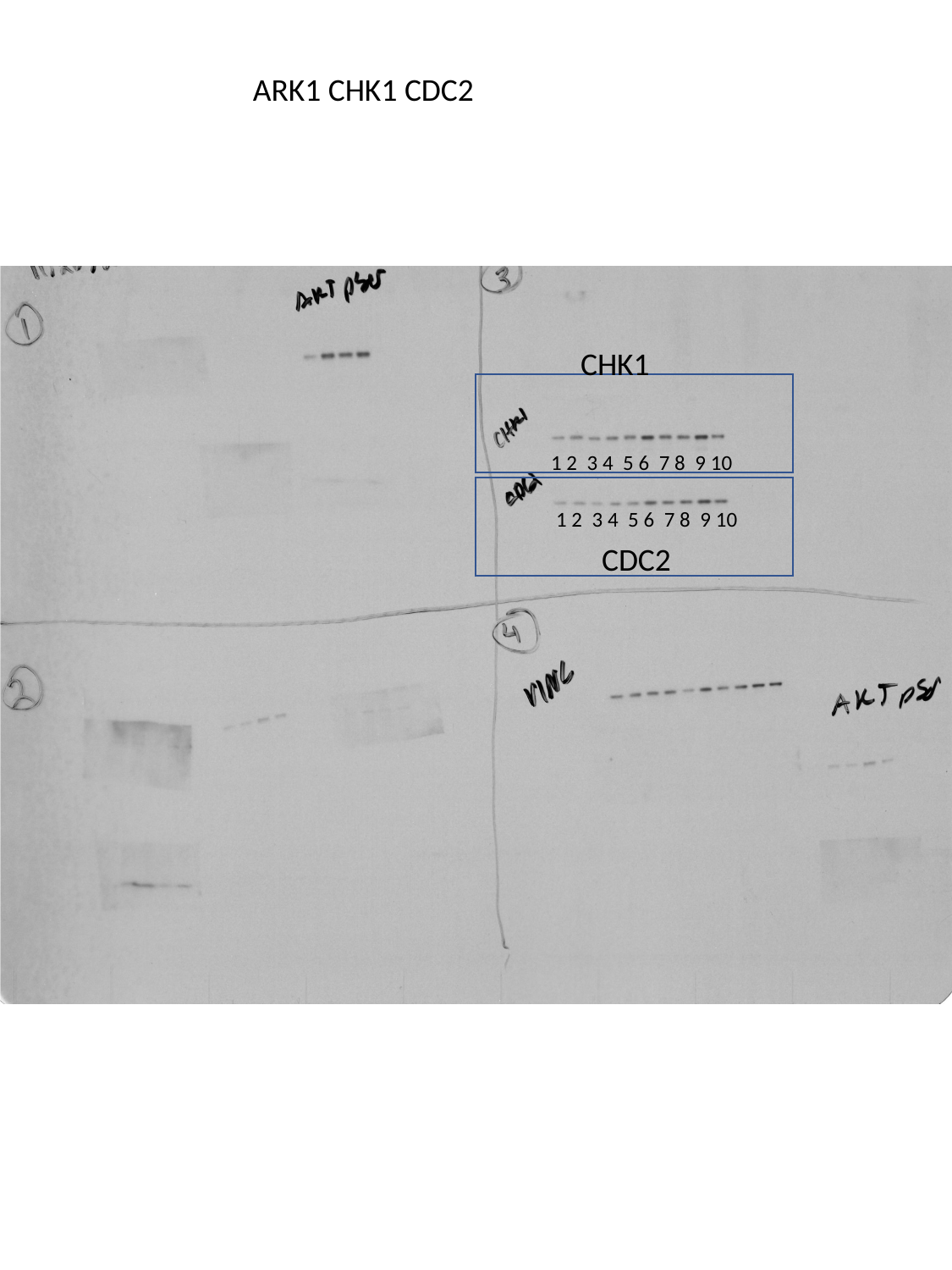

ARK1 CHK1 CDC2
CHK1
1 2 3 4 5 6 7 8 9 10
1 2 3 4 5 6 7 8 9 10
CDC2

## Slide 3
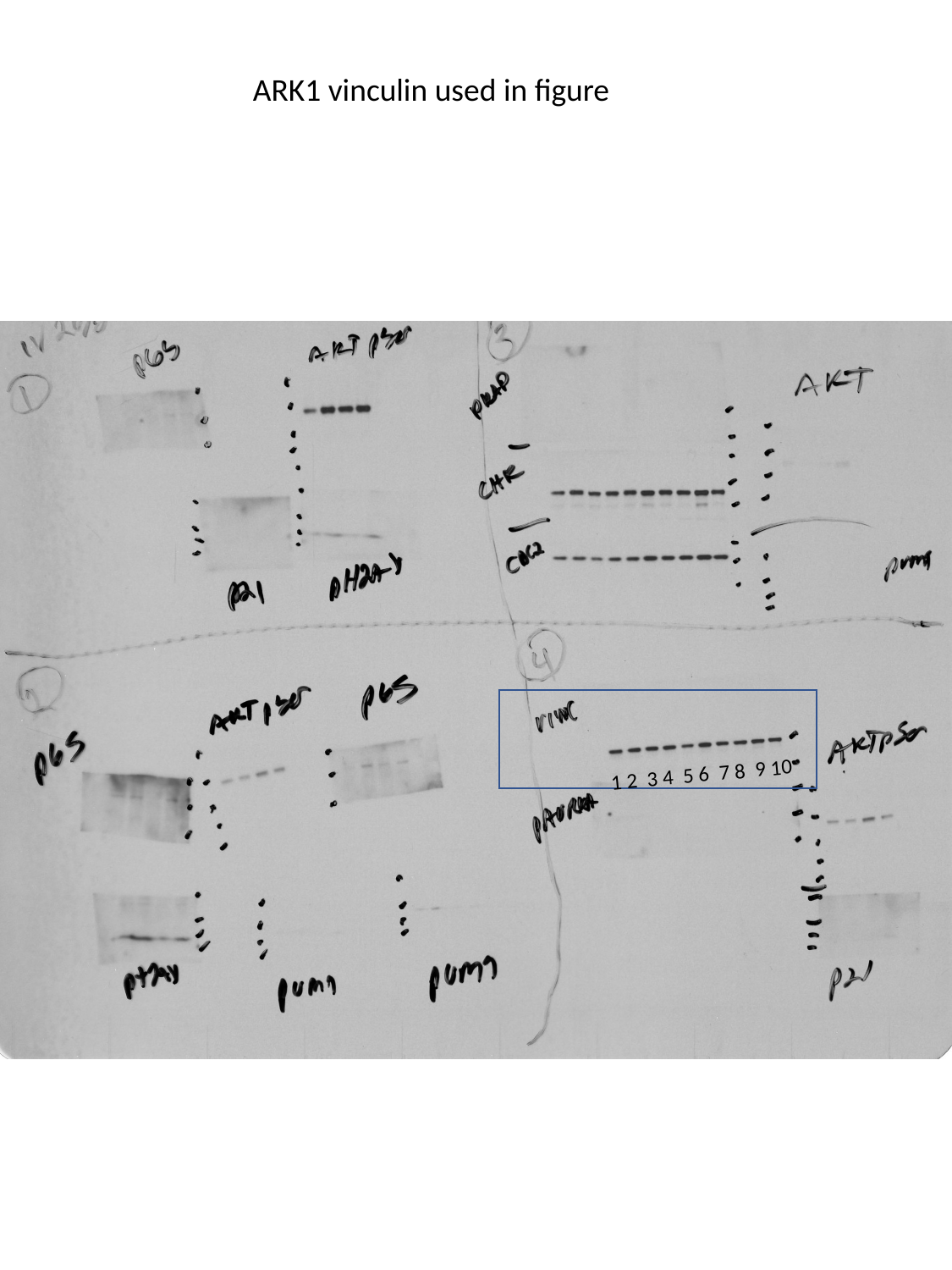

ARK1 vinculin used in figure
1 2 3 4 5 6 7 8 9 10

## Slide 4
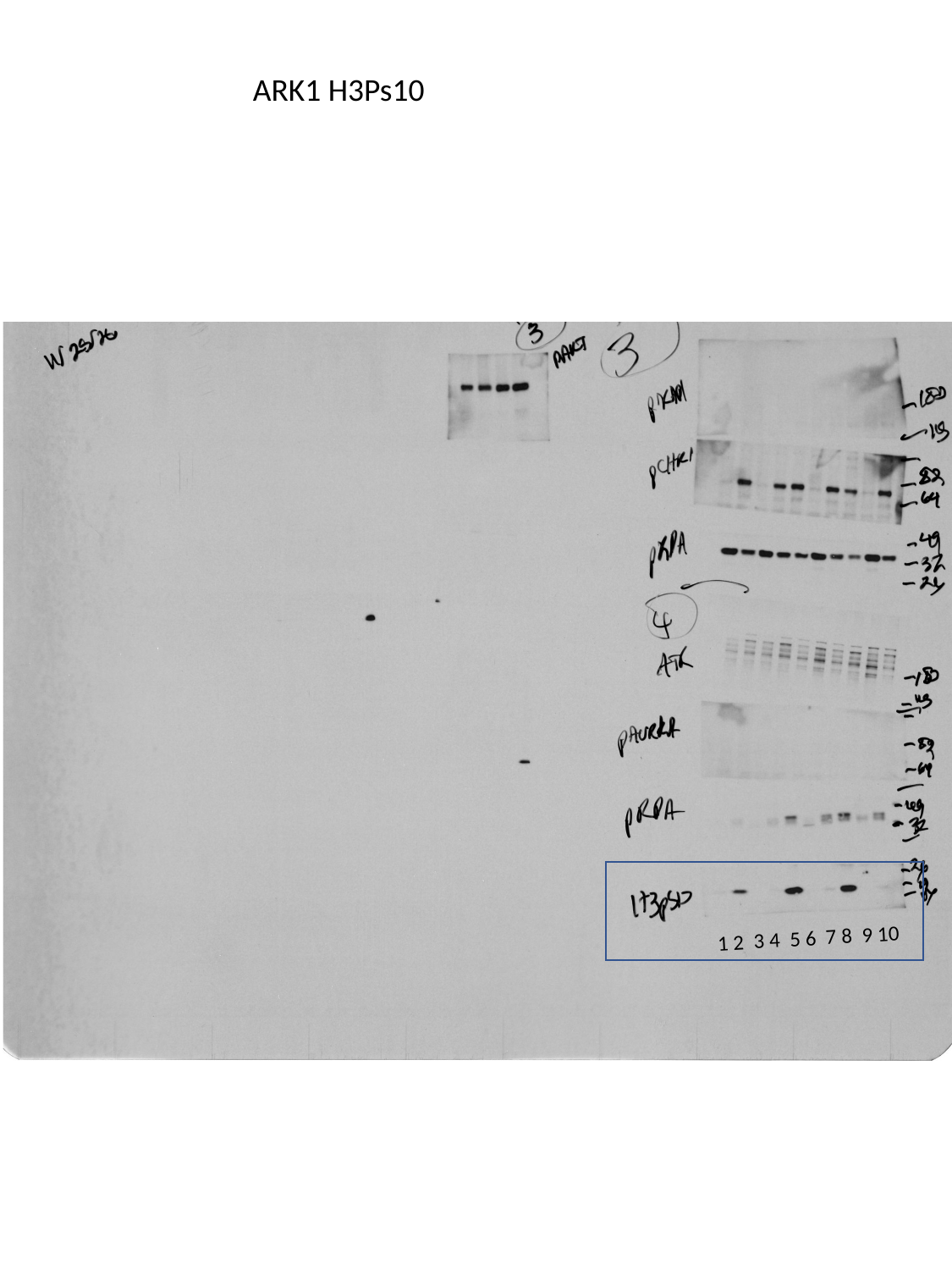

ARK1 H3Ps10
1 2 3 4 5 6 7 8 9 10

## Slide 5
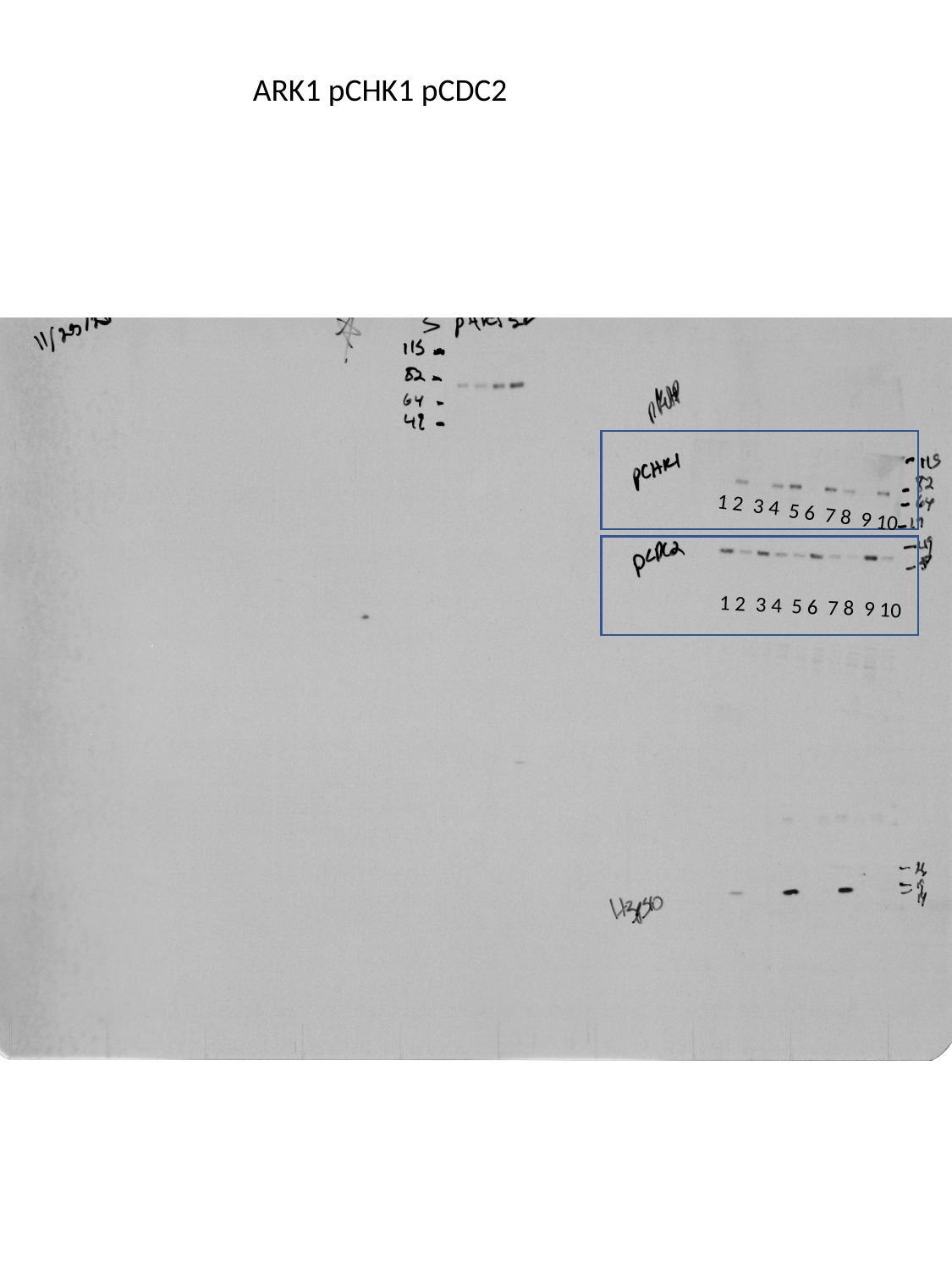

ARK1 pCHK1 pCDC2
1 2 3 4 5 6 7 8 9 10
1 2 3 4 5 6 7 8 9 10

## Slide 6
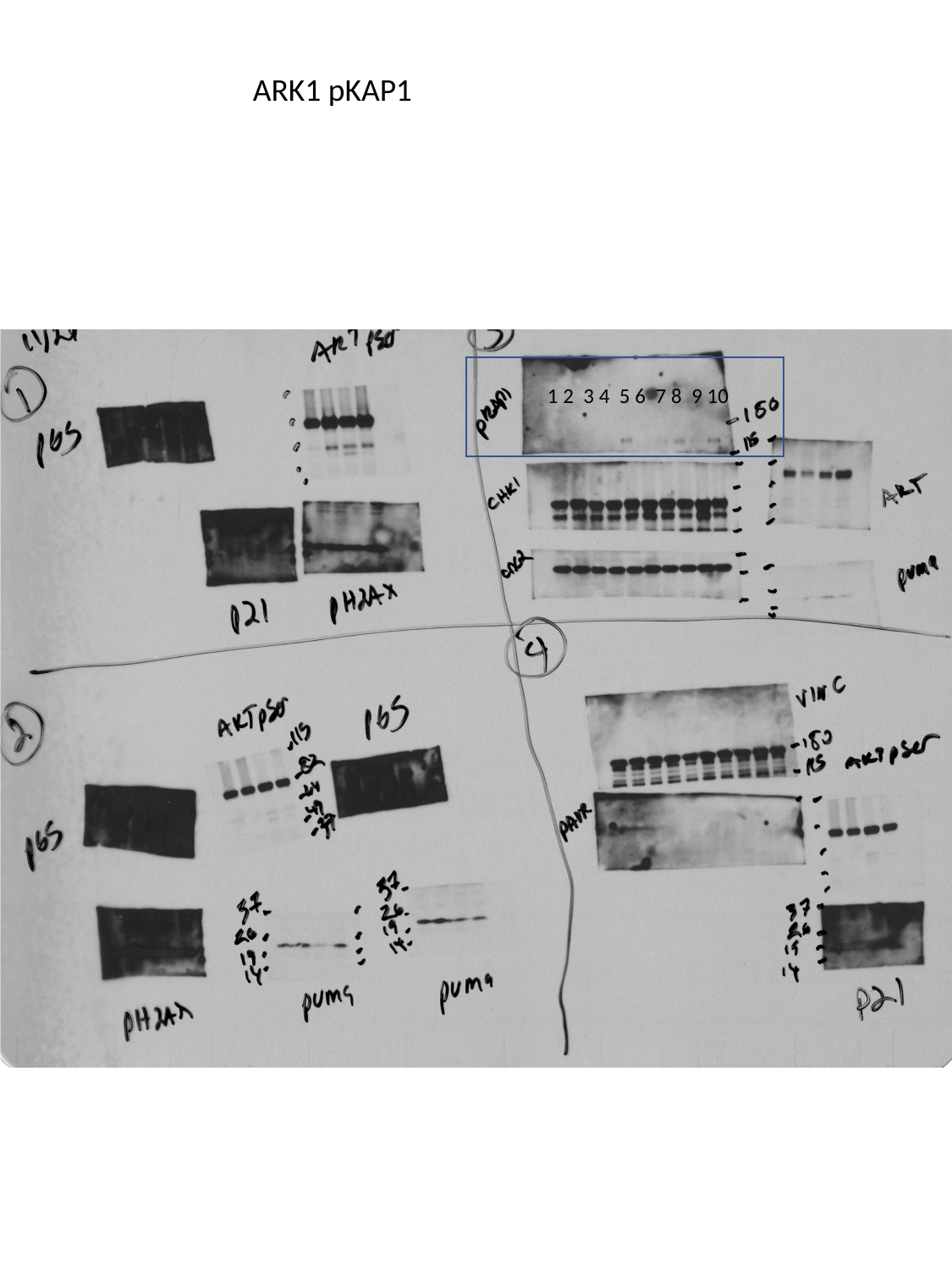

ARK1 pKAP1
1 2 3 4 5 6 7 8 9 10

## Slide 7
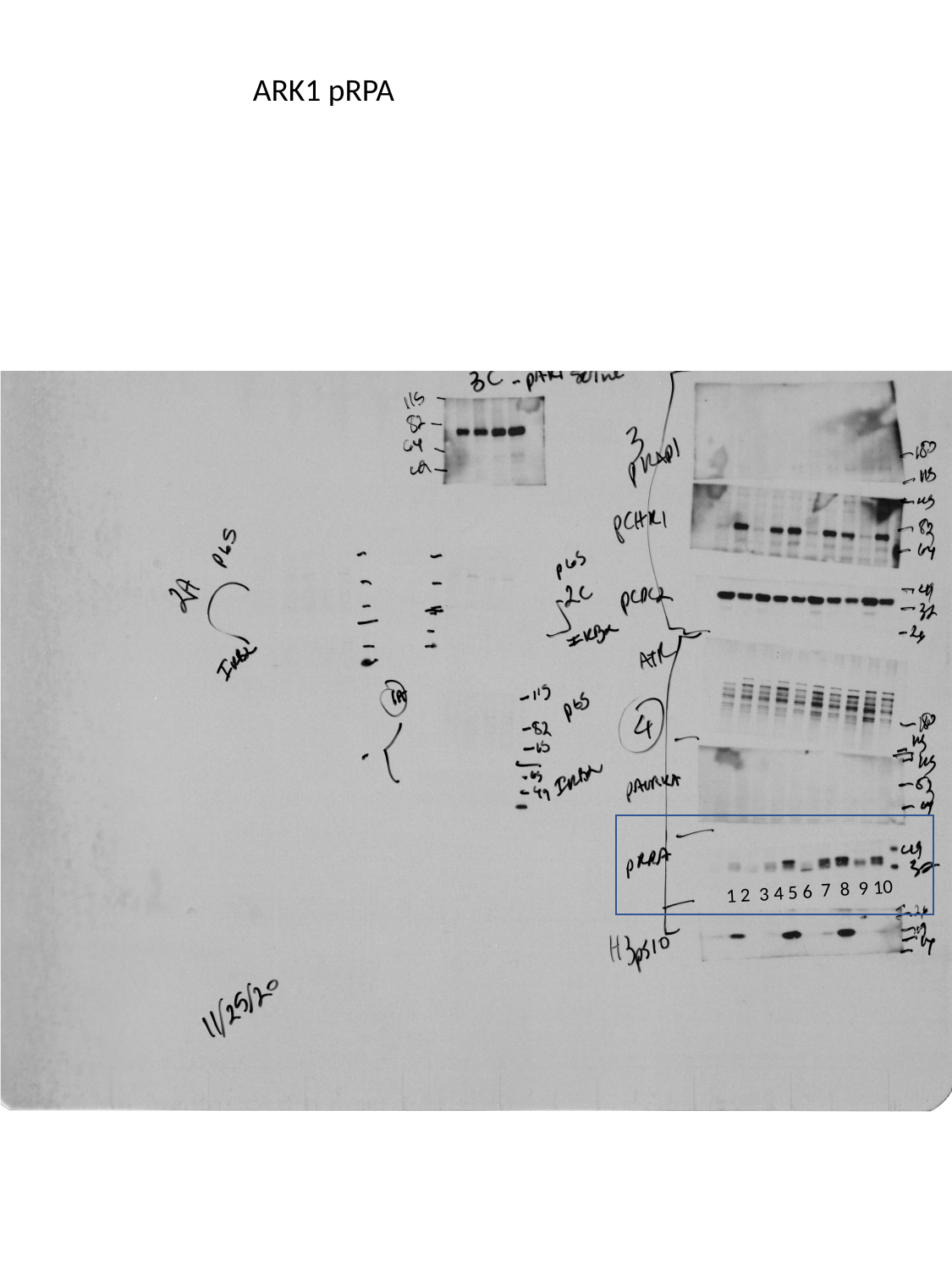

ARK1 pRPA
1 2 3 4 5 6 7 8 9 10

## Slide 8
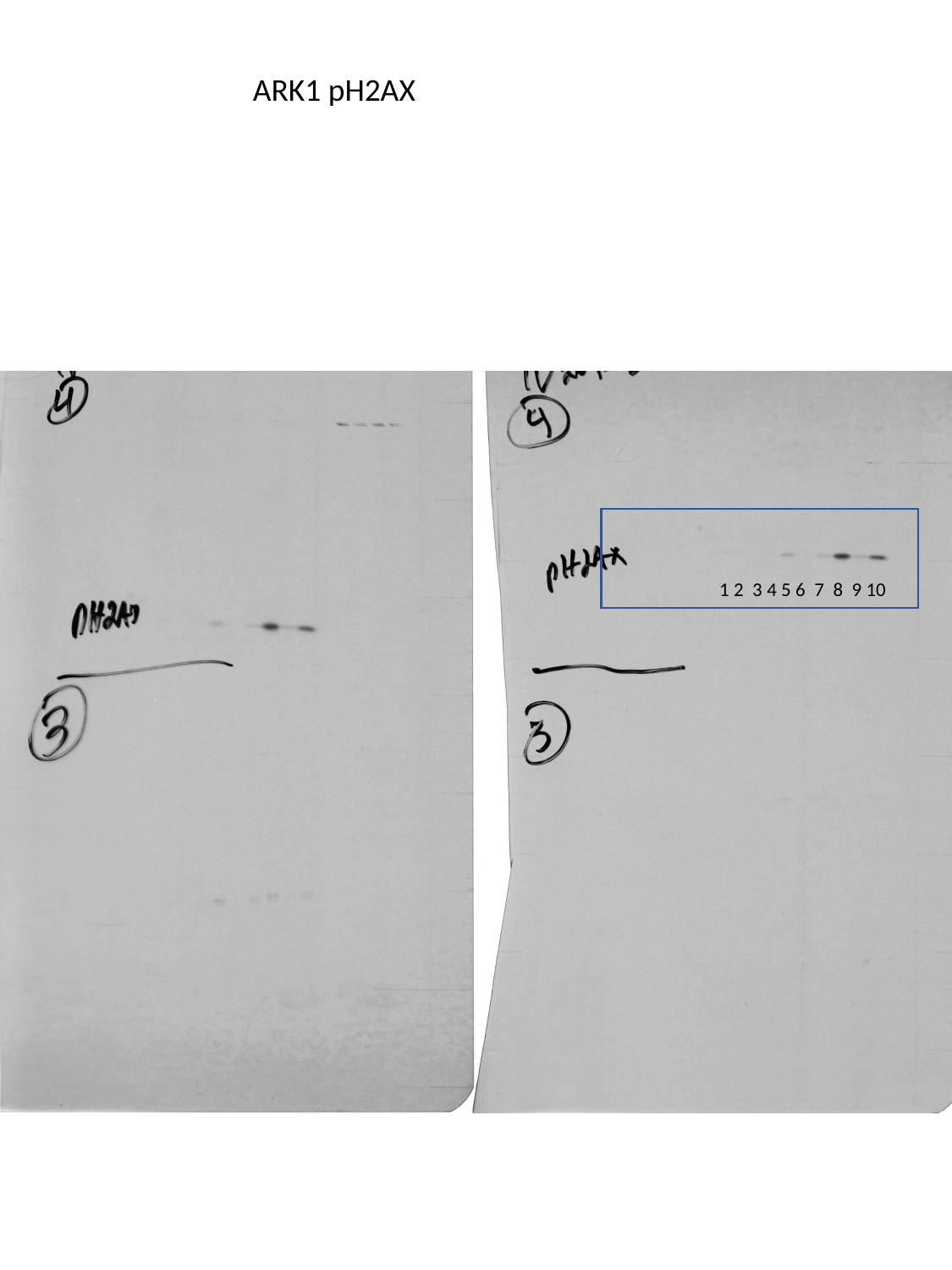

ARK1 pH2AX
1 2 3 4 5 6 7 8 9 10

## Slide 9
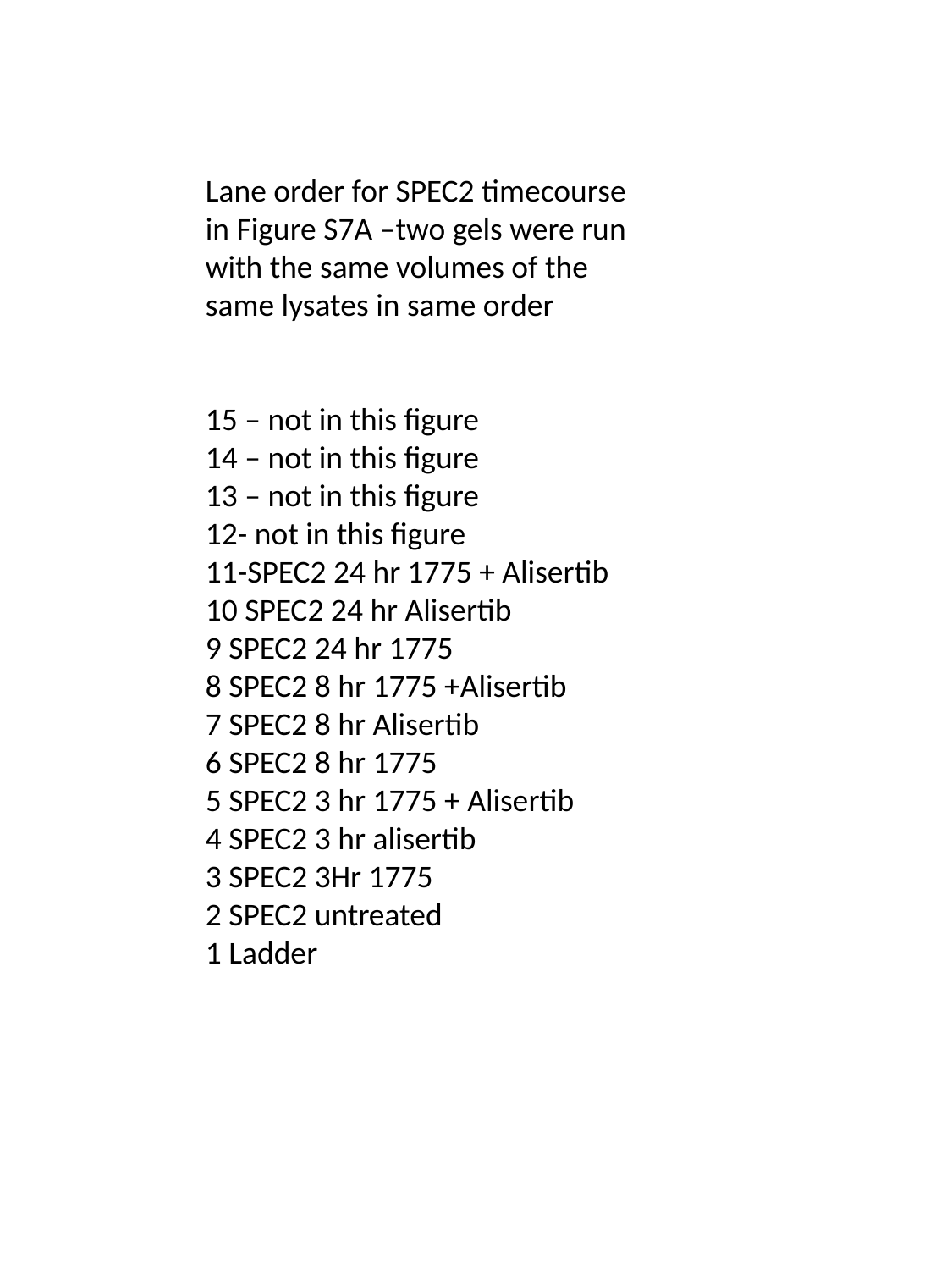

Lane order for SPEC2 timecourse in Figure S7A –two gels were run with the same volumes of the same lysates in same order
15 – not in this figure
14 – not in this figure
13 – not in this figure
12- not in this figure
11-SPEC2 24 hr 1775 + Alisertib
10 SPEC2 24 hr Alisertib
9 SPEC2 24 hr 1775
8 SPEC2 8 hr 1775 +Alisertib
7 SPEC2 8 hr Alisertib
6 SPEC2 8 hr 1775
5 SPEC2 3 hr 1775 + Alisertib
4 SPEC2 3 hr alisertib
3 SPEC2 3Hr 1775
2 SPEC2 untreated
1 Ladder

## Slide 10
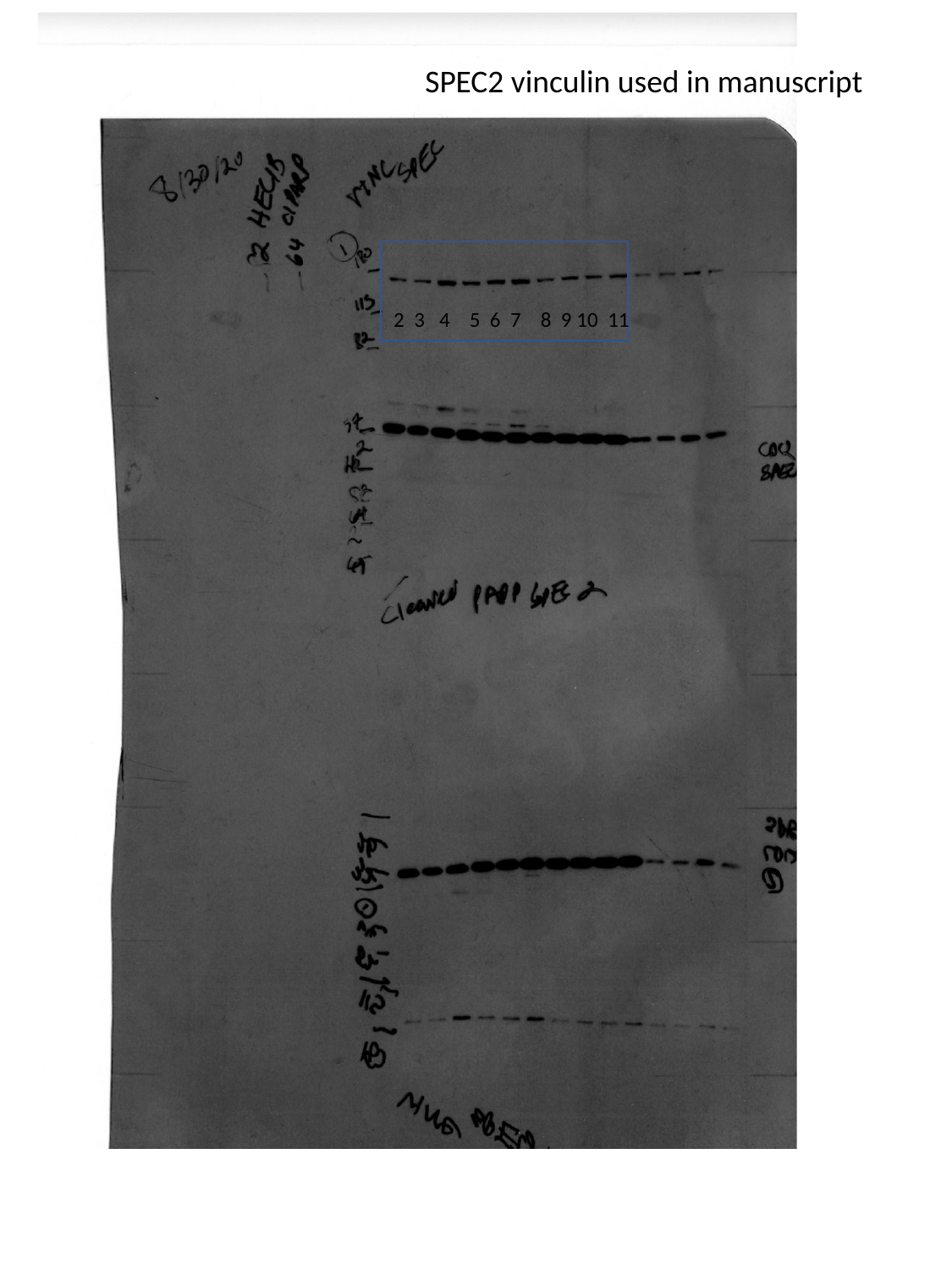

SPEC2 vinculin used in manuscript
2 3 4 5 6 7 8 9 10 11

## Slide 11
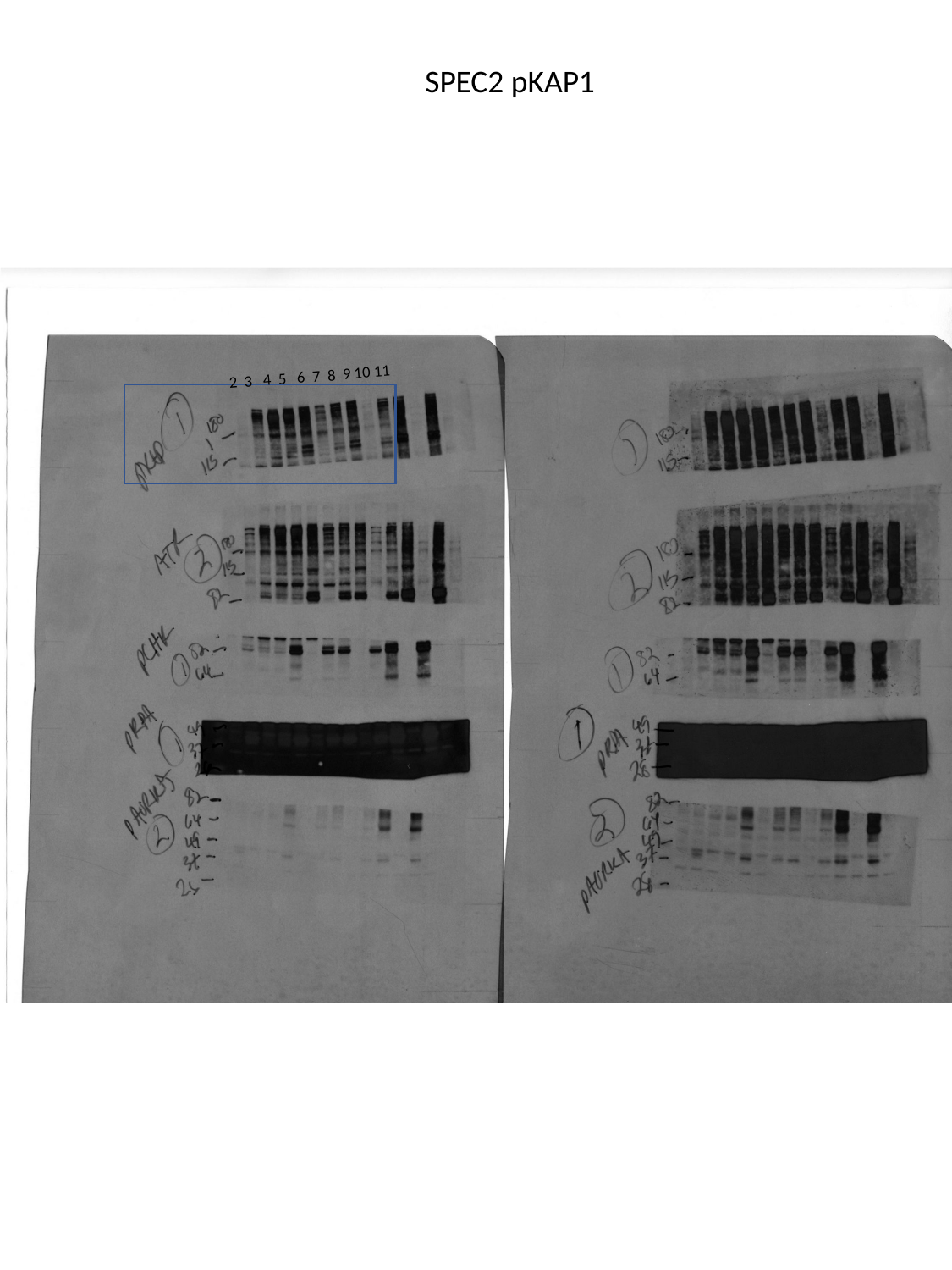

SPEC2 pKAP1
2 3 4 5 6 7 8 9 10 11

## Slide 12
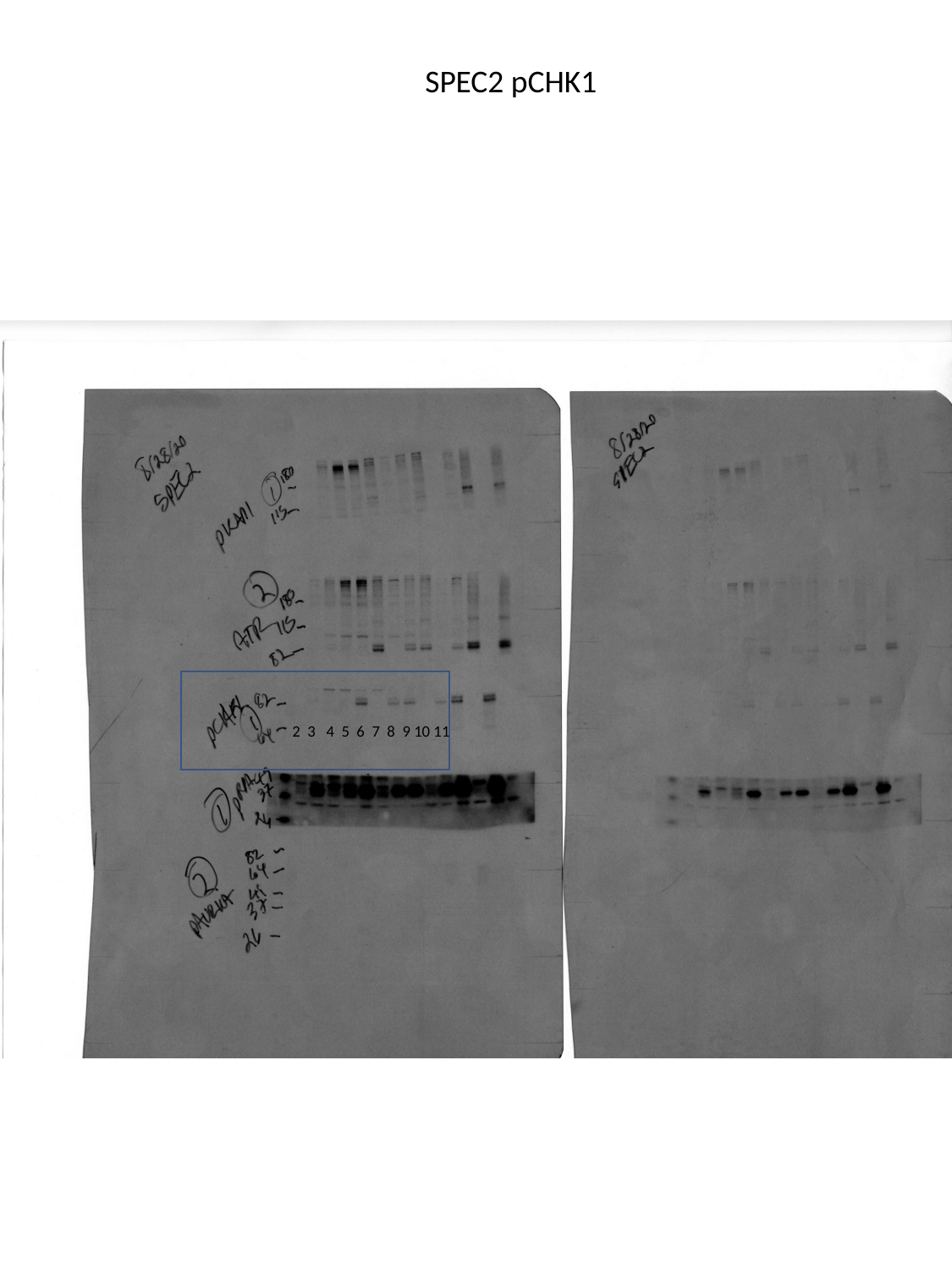

SPEC2 pCHK1
2 3 4 5 6 7 8 9 10 11

## Slide 13
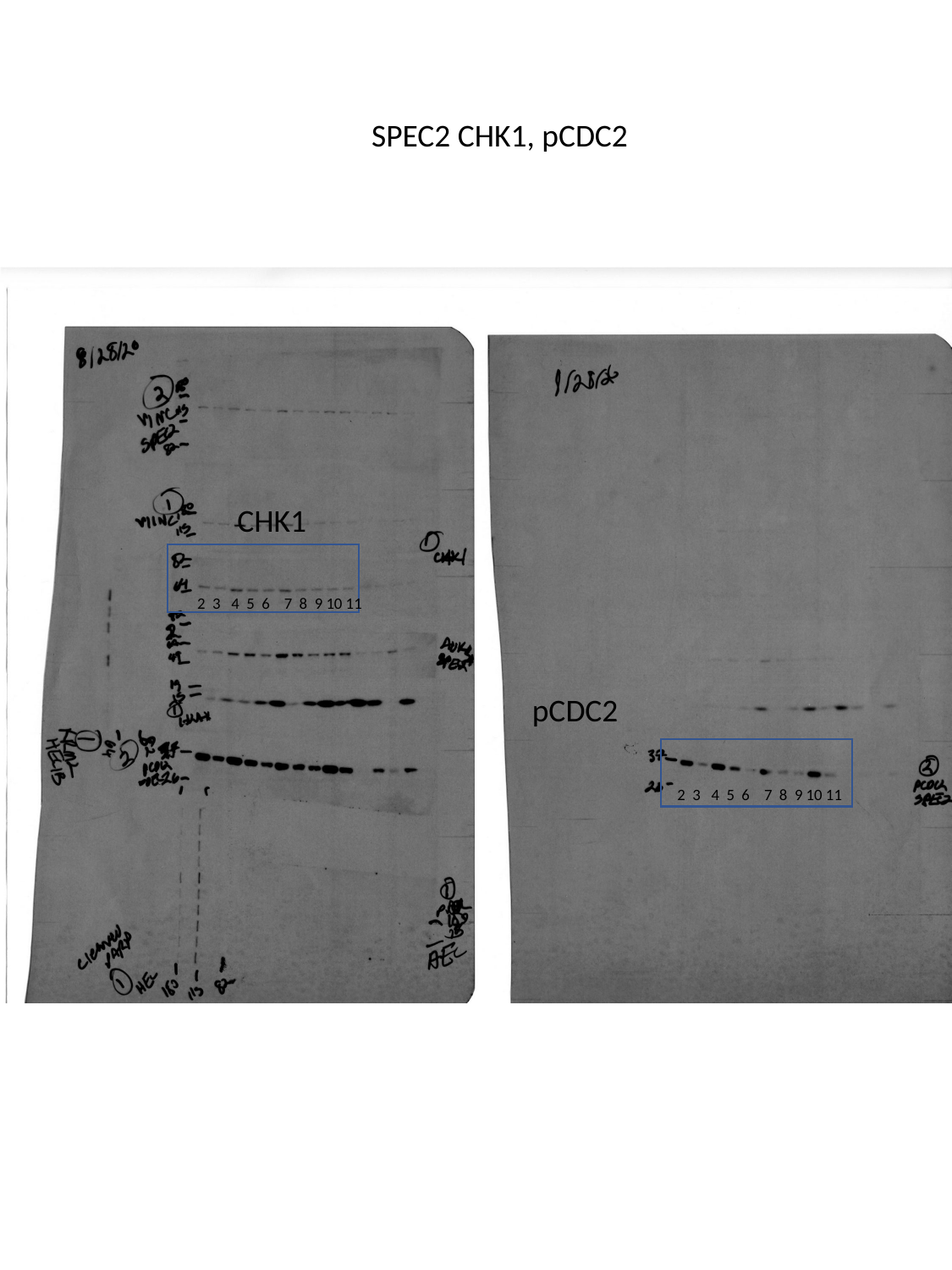

SPEC2 CHK1, pCDC2
CHK1
2 3 4 5 6 7 8 9 10 11
pCDC2
2 3 4 5 6 7 8 9 10 11

## Slide 14
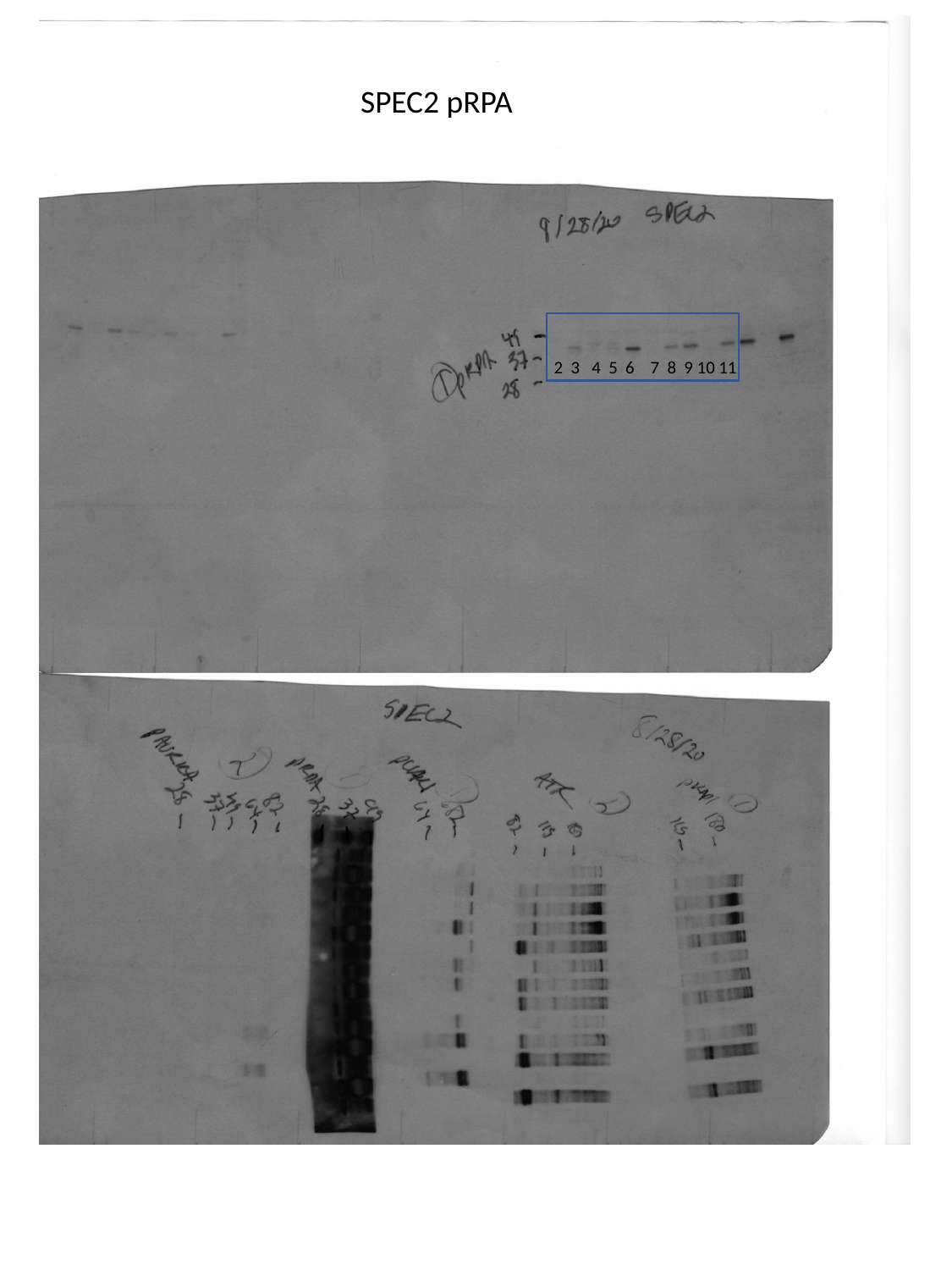

#
SPEC2 pRPA
2 3 4 5 6 7 8 9 10 11

## Slide 15
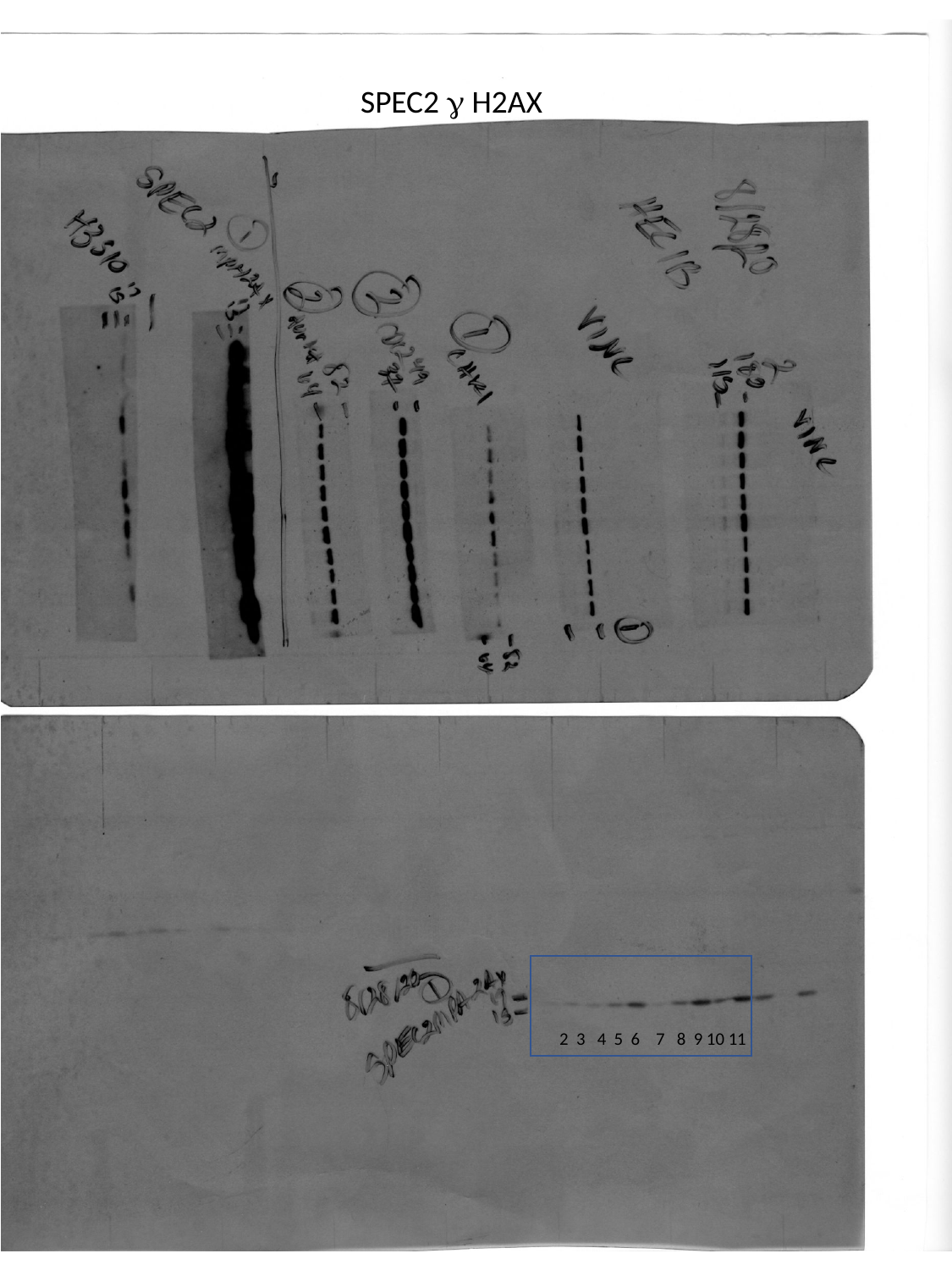

SPEC2 γ H2AX
2 3 4 5 6 7 8 9 10 11

## Slide 16
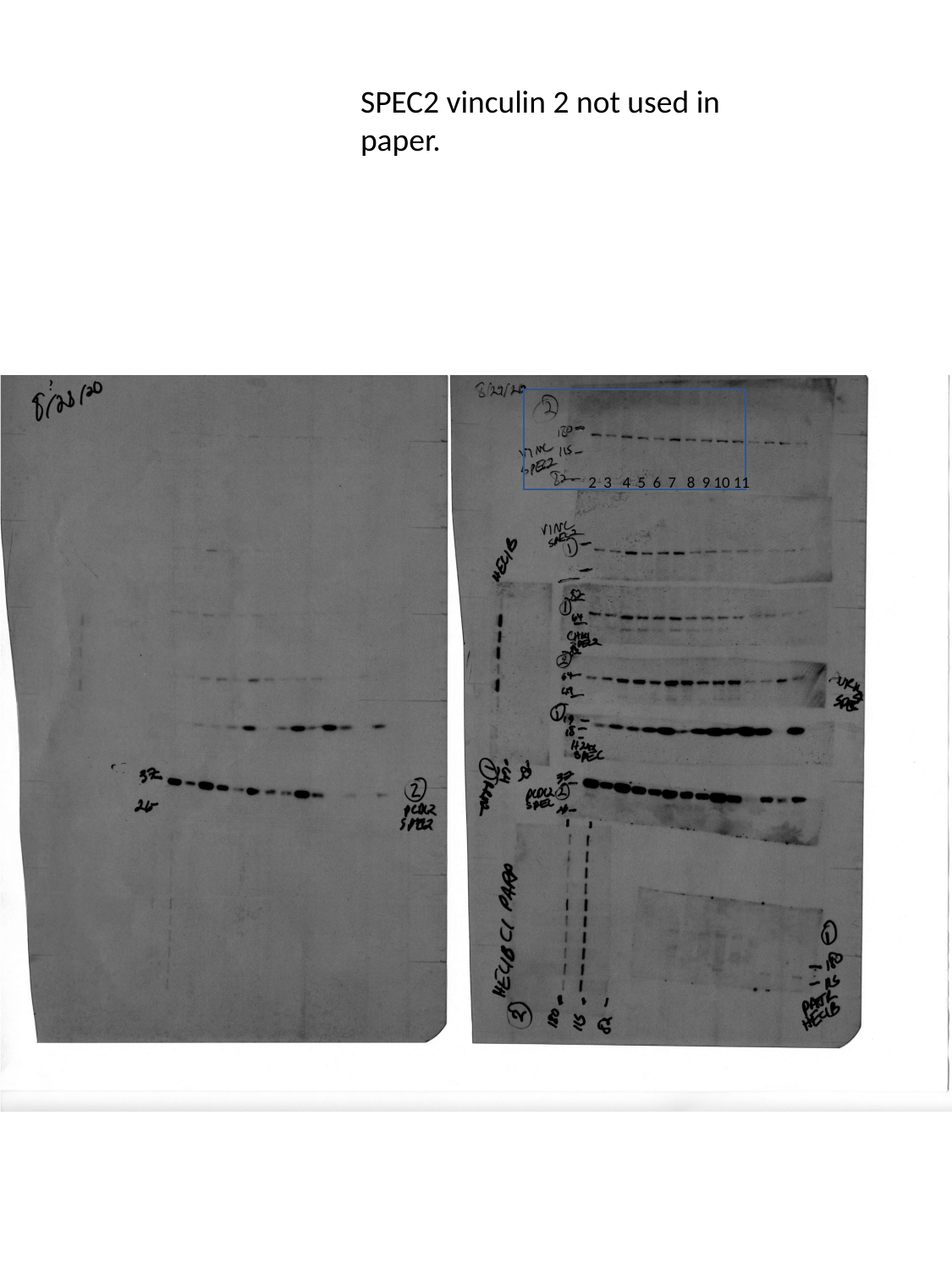

SPEC2 vinculin 2 not used in paper.
2 3 4 5 6 7 8 9 10 11

## Slide 17
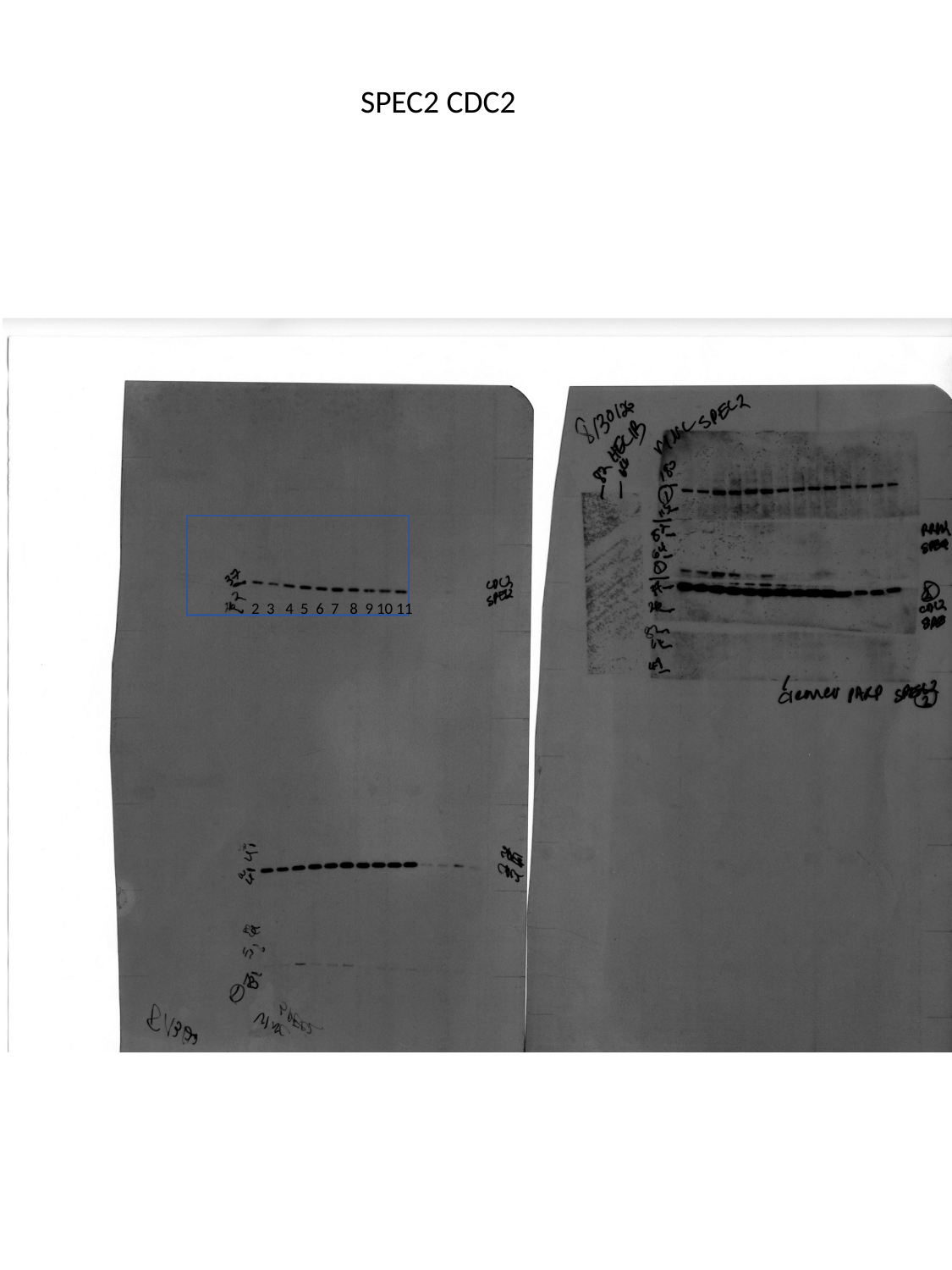

SPEC2 CDC2
2 3 4 5 6 7 8 9 10 11

## Slide 18
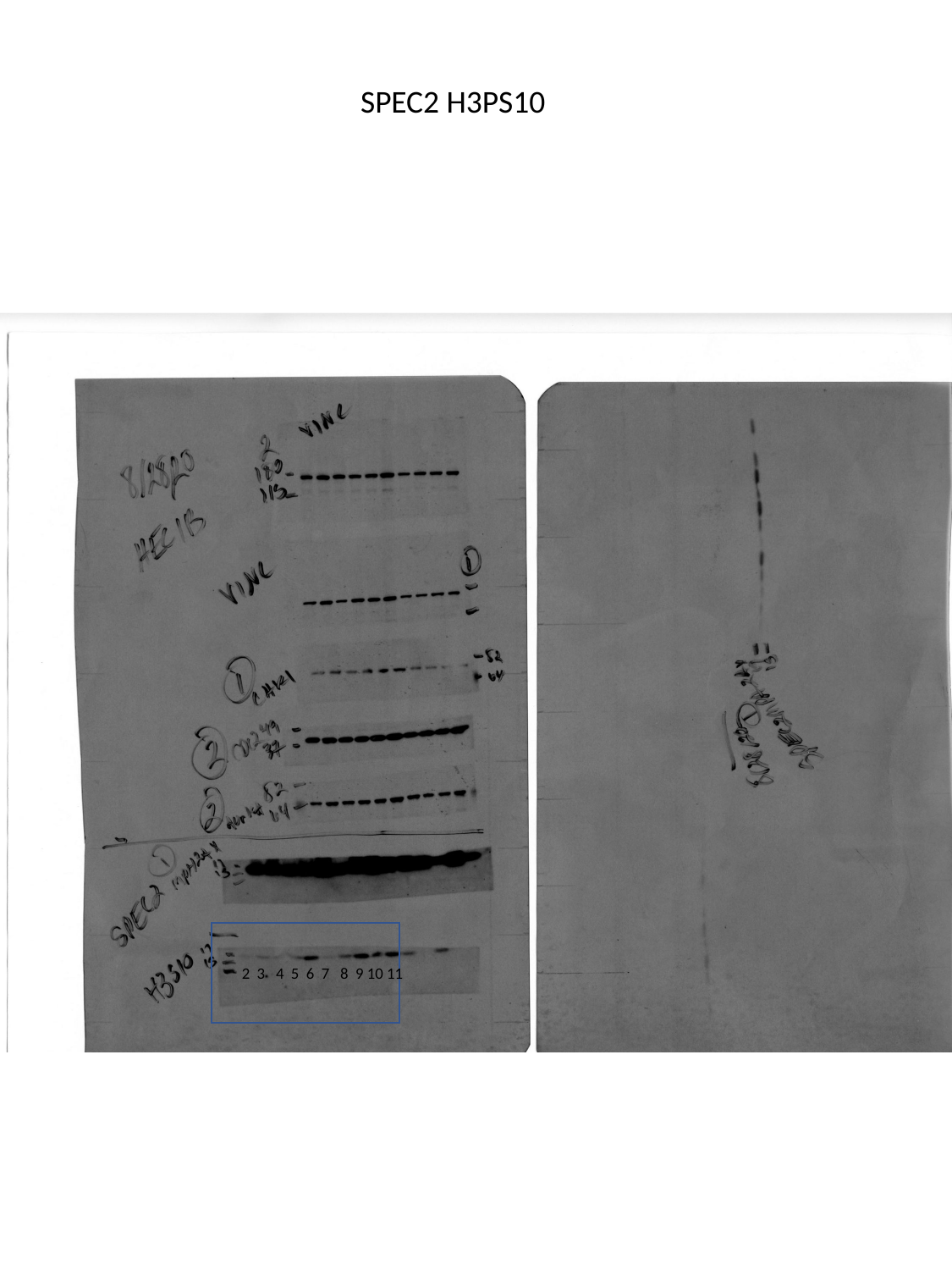

#
SPEC2 H3PS10
2 3 4 5 6 7 8 9 10 11

## Slide 19
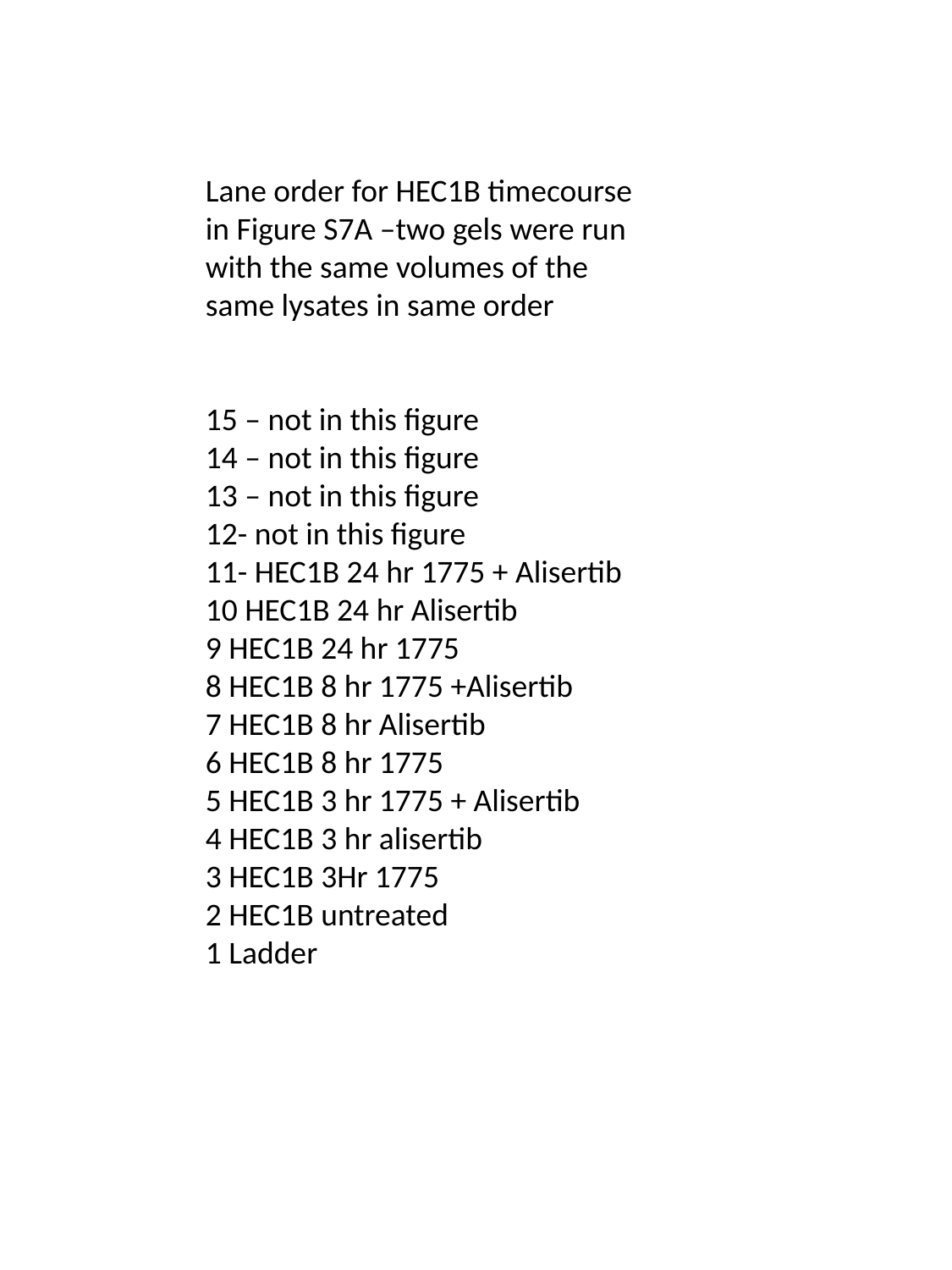

Lane order for HEC1B timecourse in Figure S7A –two gels were run with the same volumes of the same lysates in same order
15 – not in this figure
14 – not in this figure
13 – not in this figure
12- not in this figure
11- HEC1B 24 hr 1775 + Alisertib
10 HEC1B 24 hr Alisertib
9 HEC1B 24 hr 1775
8 HEC1B 8 hr 1775 +Alisertib
7 HEC1B 8 hr Alisertib
6 HEC1B 8 hr 1775
5 HEC1B 3 hr 1775 + Alisertib
4 HEC1B 3 hr alisertib
3 HEC1B 3Hr 1775
2 HEC1B untreated
1 Ladder

## Slide 20
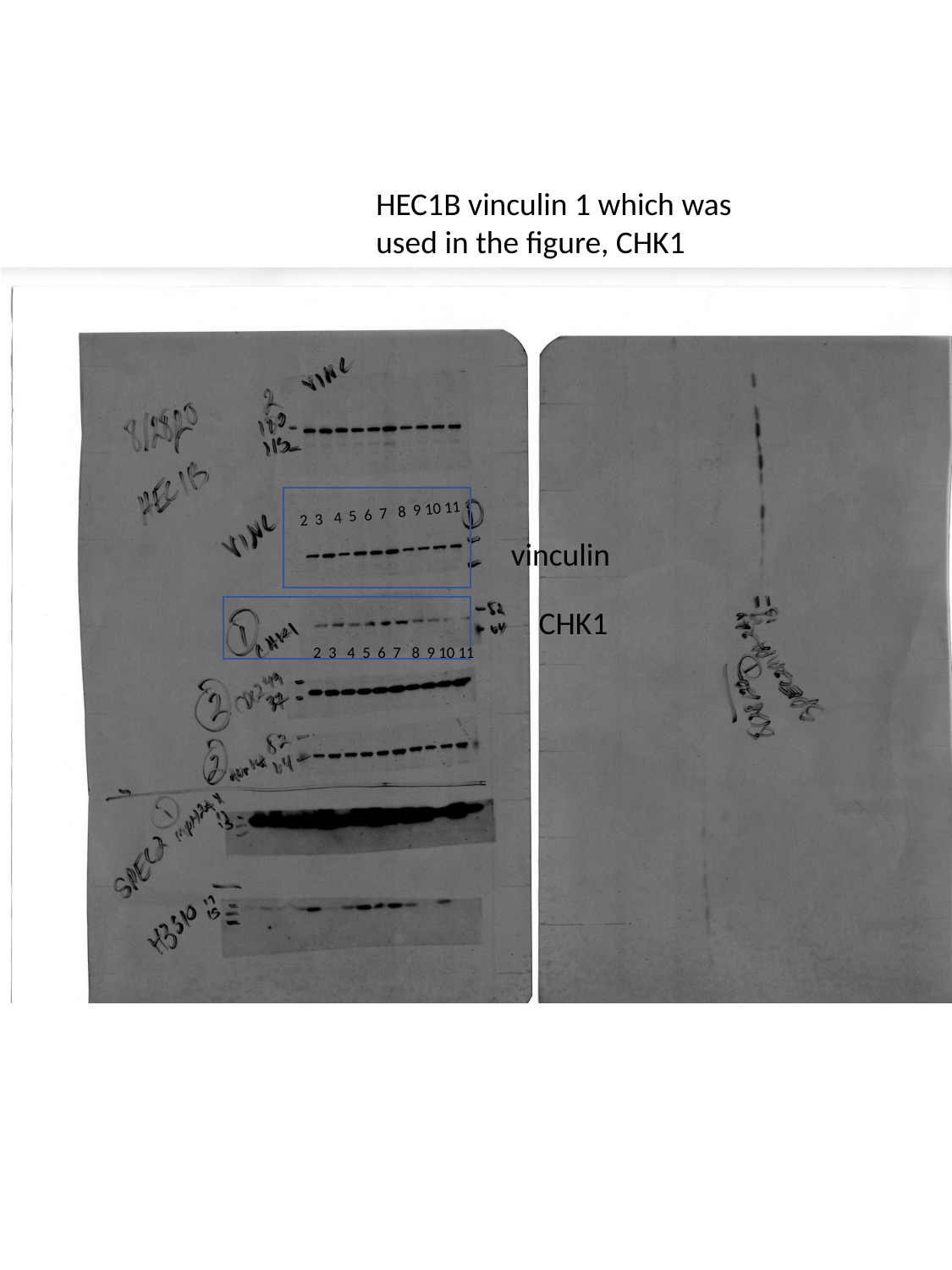

HEC1B vinculin 1 which was used in the figure, CHK1
2 3 4 5 6 7 8 9 10 11
vinculin
CHK1
2 3 4 5 6 7 8 9 10 11

## Slide 21
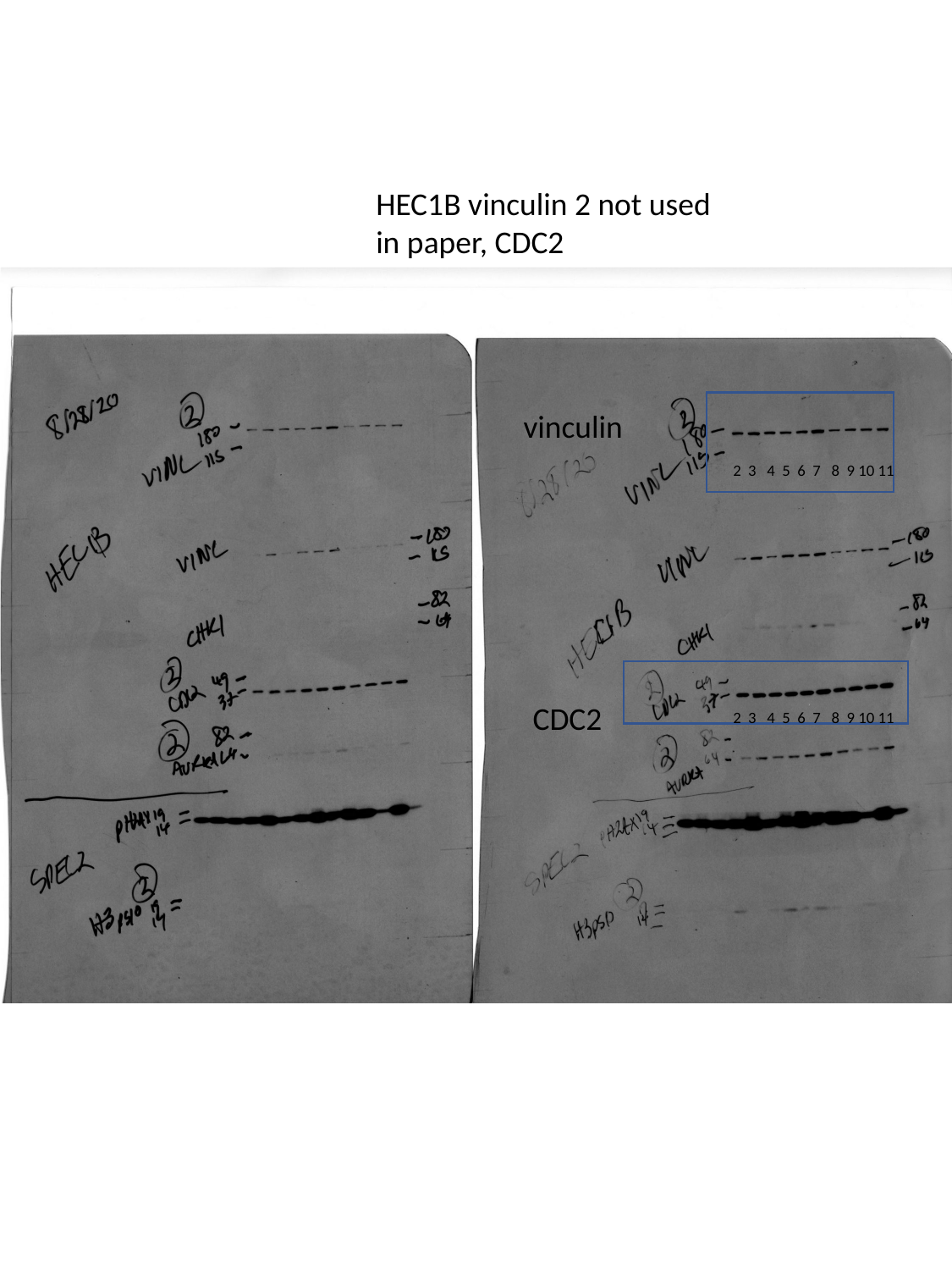

HEC1B vinculin 2 not used in paper, CDC2
vinculin
2 3 4 5 6 7 8 9 10 11
CDC2
2 3 4 5 6 7 8 9 10 11

## Slide 22
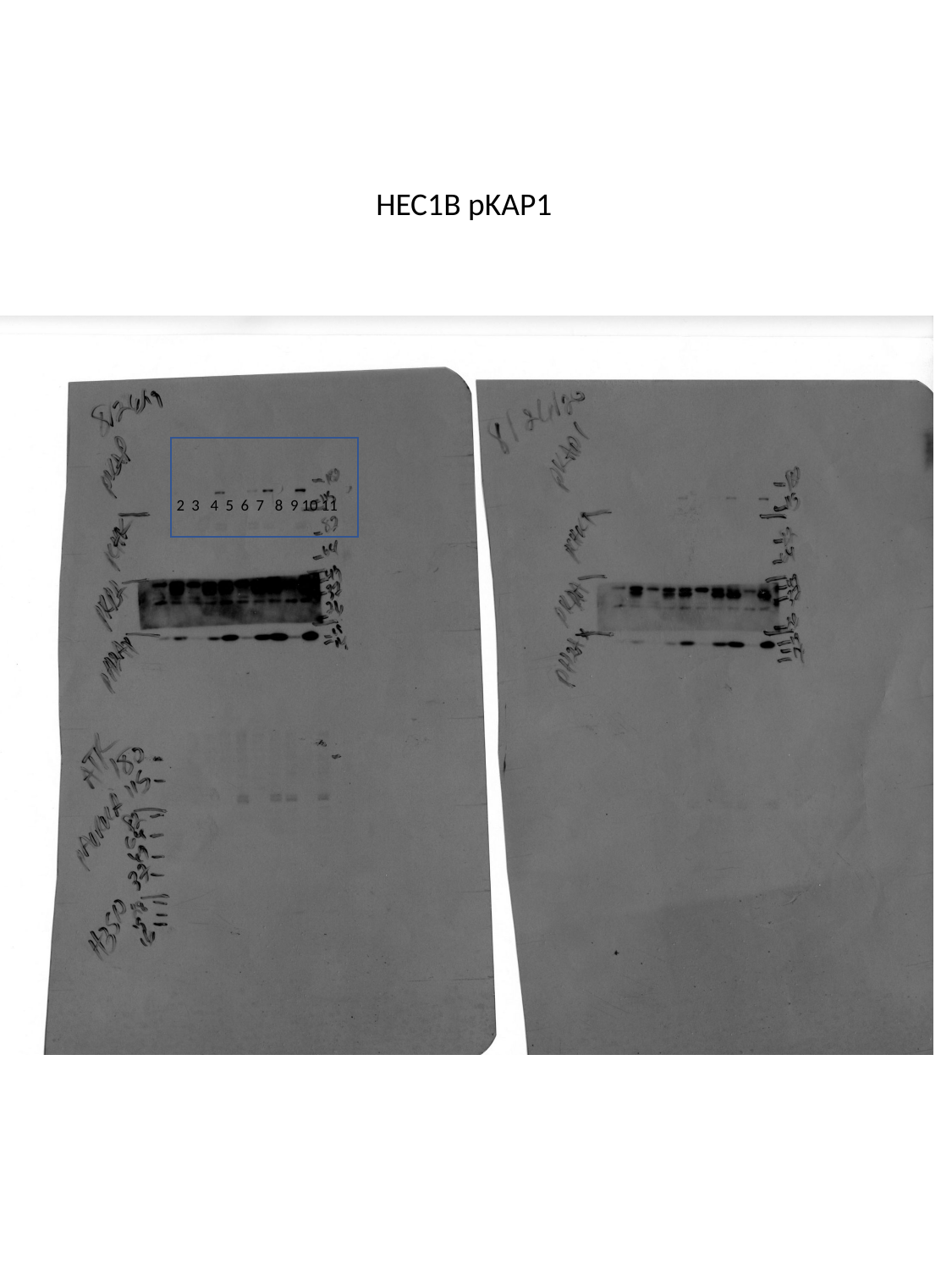

HEC1B pKAP1
2 3 4 5 6 7 8 9 10 11

## Slide 23
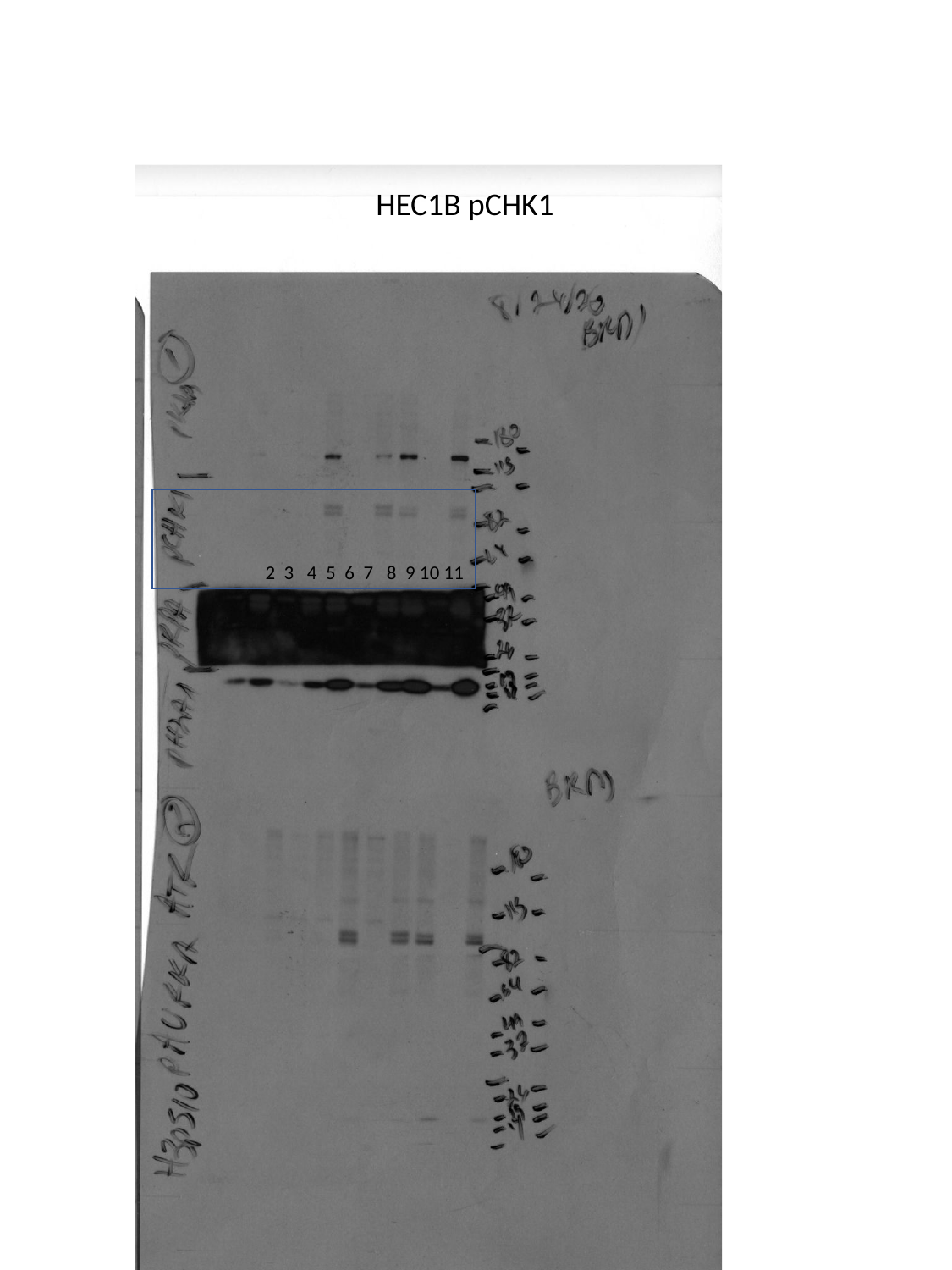

HEC1B pCHK1
2 3 4 5 6 7 8 9 10 11

## Slide 24
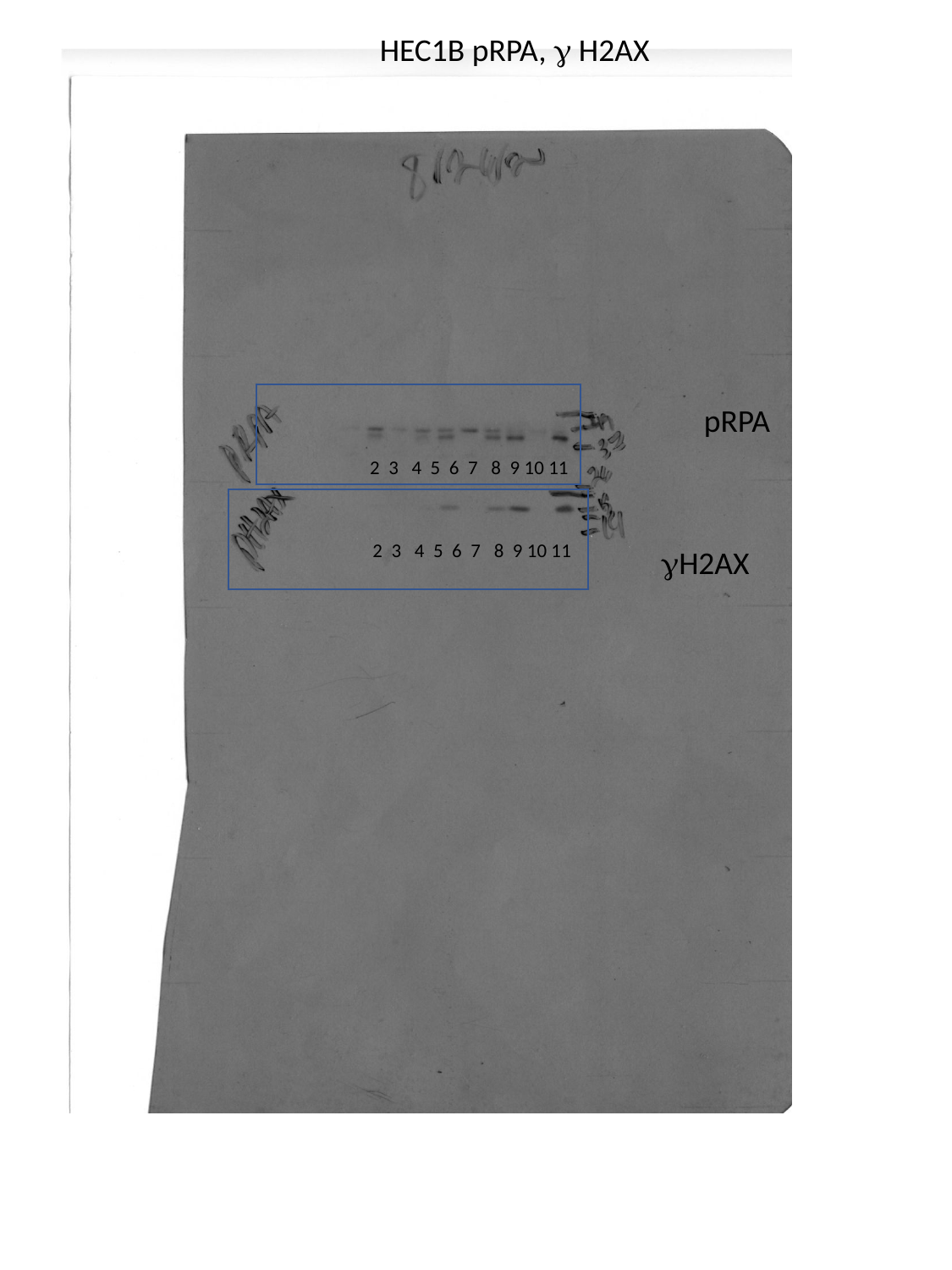

HEC1B pRPA, γ H2AX
pRPA
2 3 4 5 6 7 8 9 10 11
2 3 4 5 6 7 8 9 10 11
γH2AX

## Slide 25
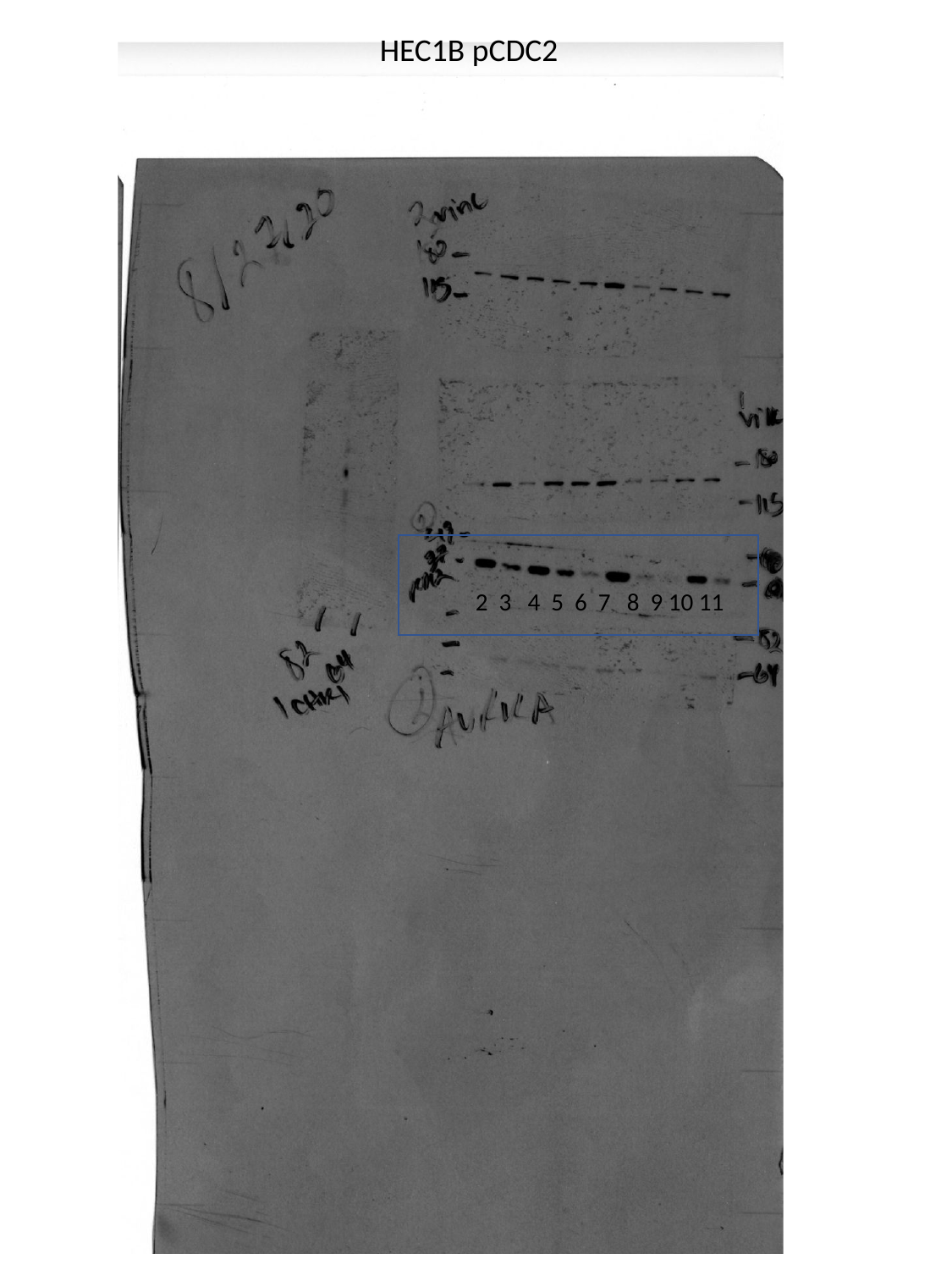

HEC1B pCDC2
#
2 3 4 5 6 7 8 9 10 11

## Slide 26
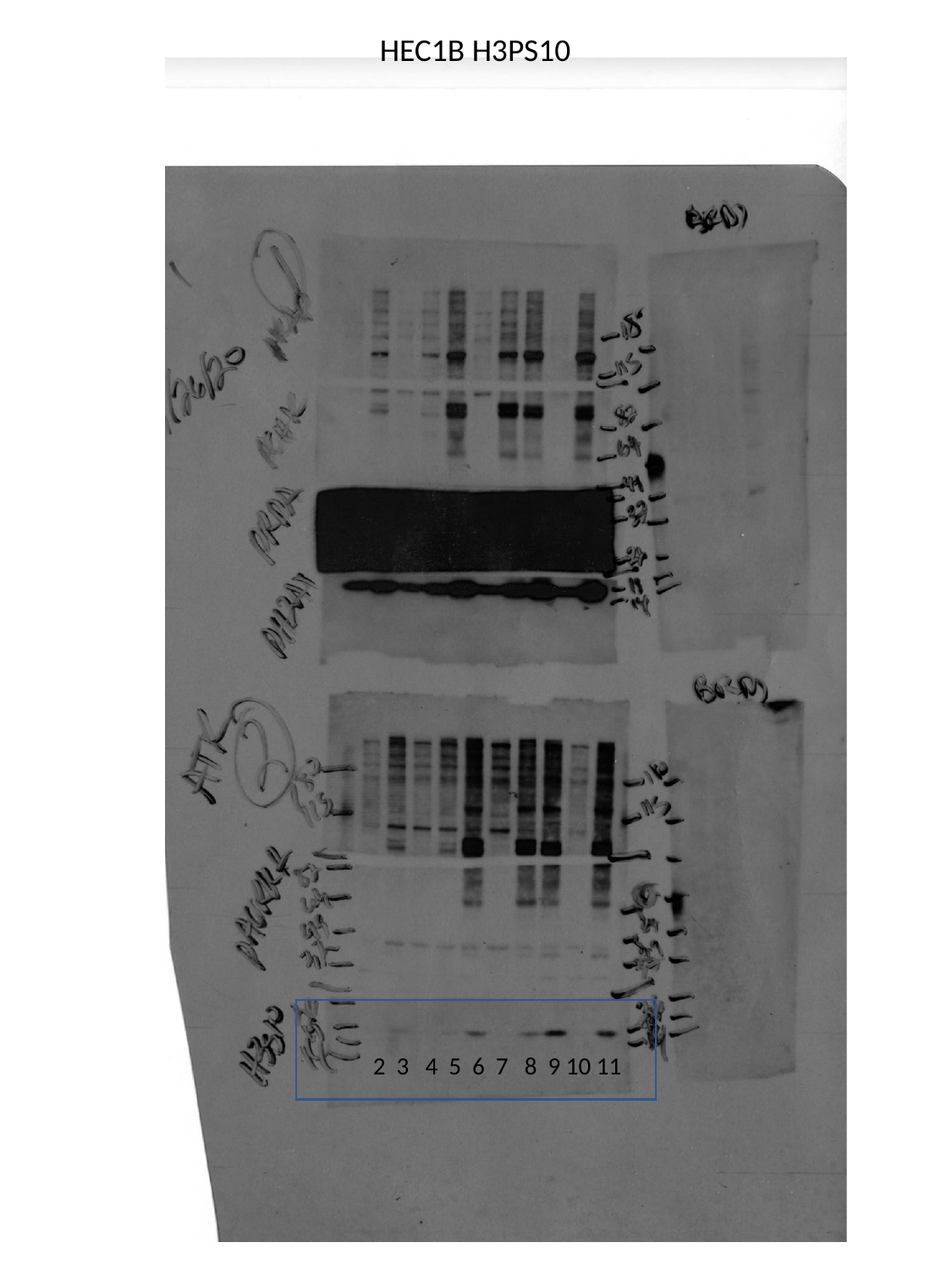

HEC1B H3PS10
2 3 4 5 6 7 8 9 10 11

## Slide 27
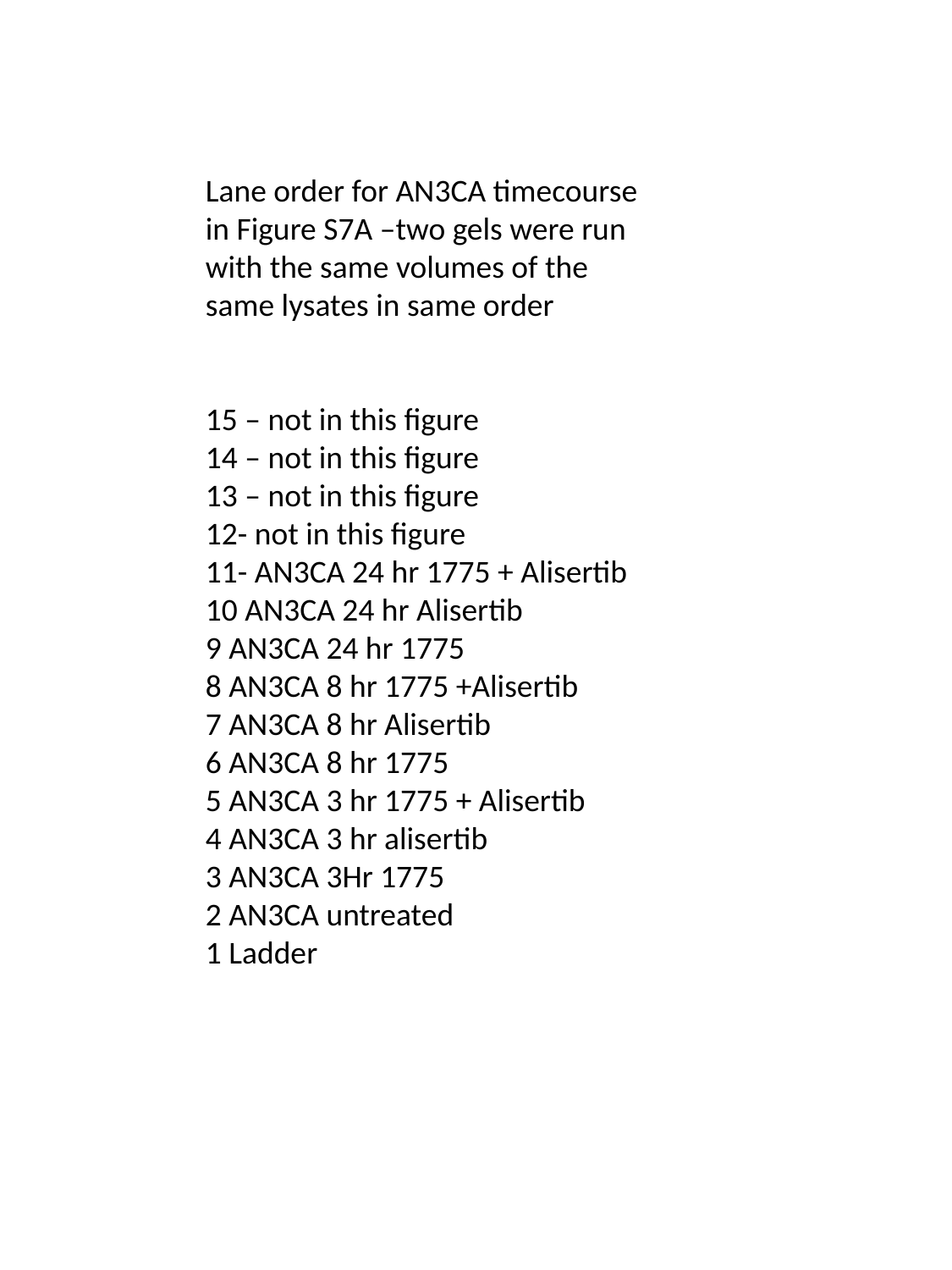

Lane order for AN3CA timecourse in Figure S7A –two gels were run with the same volumes of the same lysates in same order
15 – not in this figure
14 – not in this figure
13 – not in this figure
12- not in this figure
11- AN3CA 24 hr 1775 + Alisertib
10 AN3CA 24 hr Alisertib
9 AN3CA 24 hr 1775
8 AN3CA 8 hr 1775 +Alisertib
7 AN3CA 8 hr Alisertib
6 AN3CA 8 hr 1775
5 AN3CA 3 hr 1775 + Alisertib
4 AN3CA 3 hr alisertib
3 AN3CA 3Hr 1775
2 AN3CA untreated
1 Ladder

## Slide 28
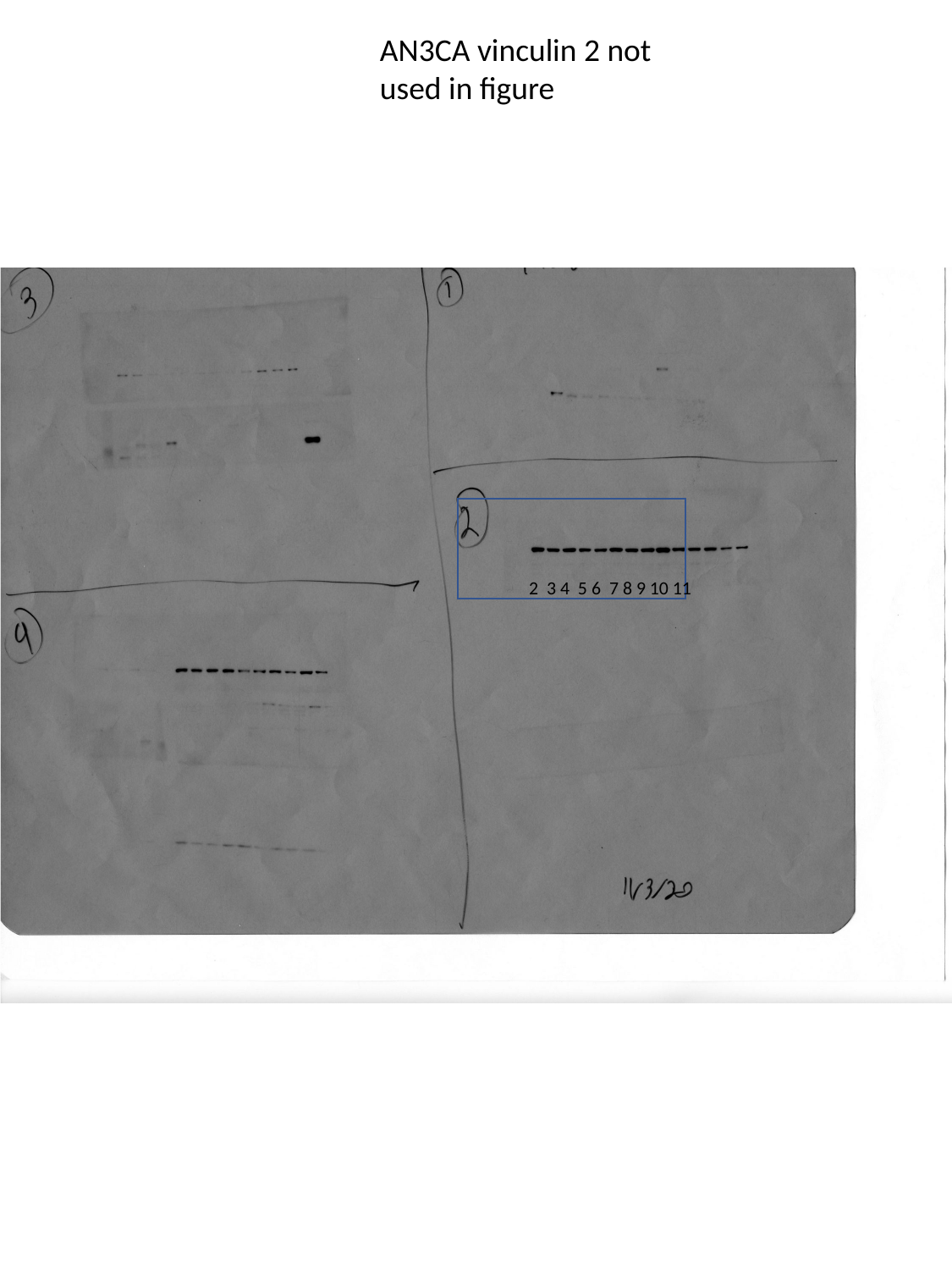

AN3CA vinculin 2 not used in figure
2 3 4 5 6 7 8 9 10 11

## Slide 29
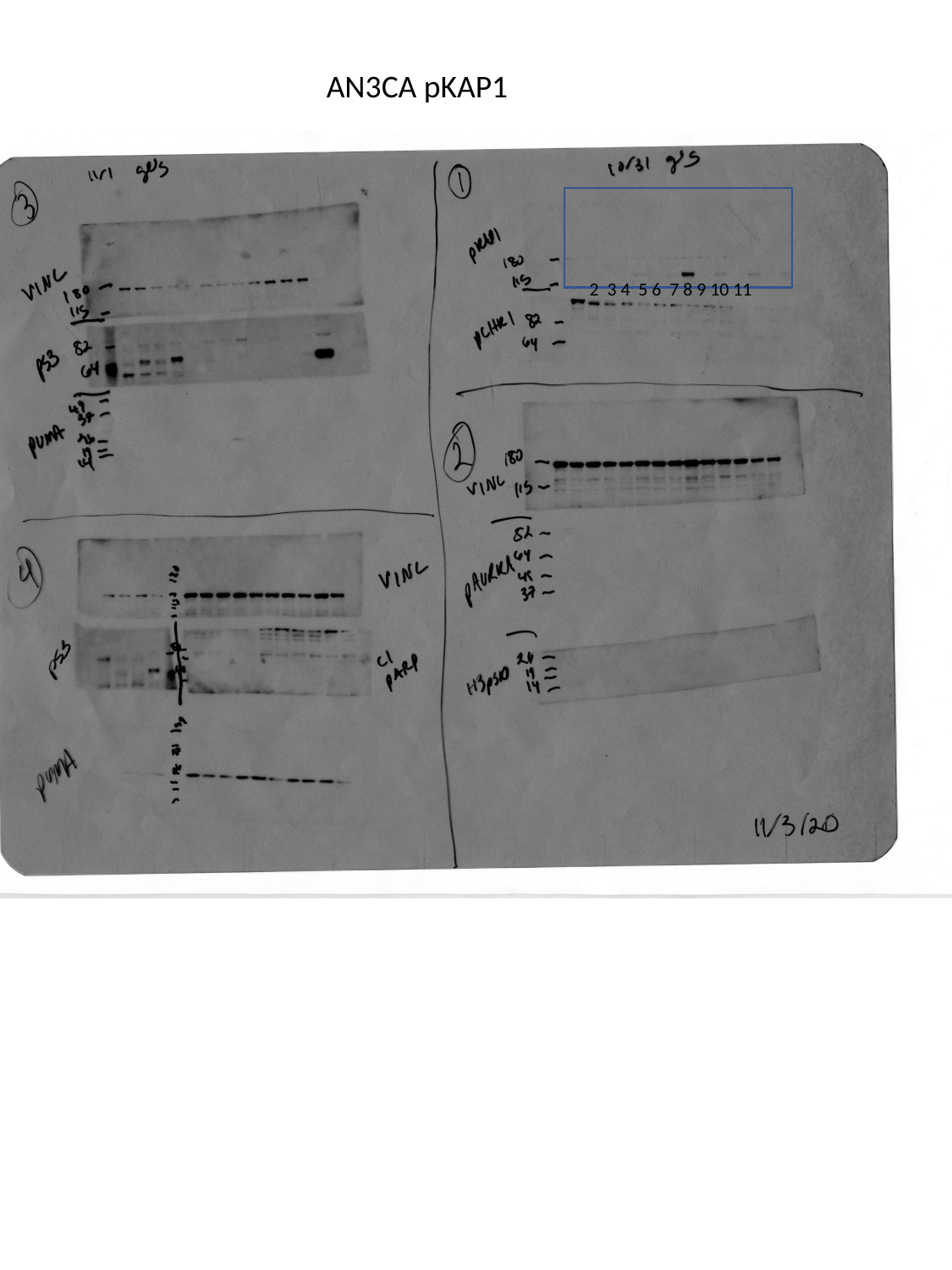

AN3CA pKAP1
2 3 4 5 6 7 8 9 10 11

## Slide 30
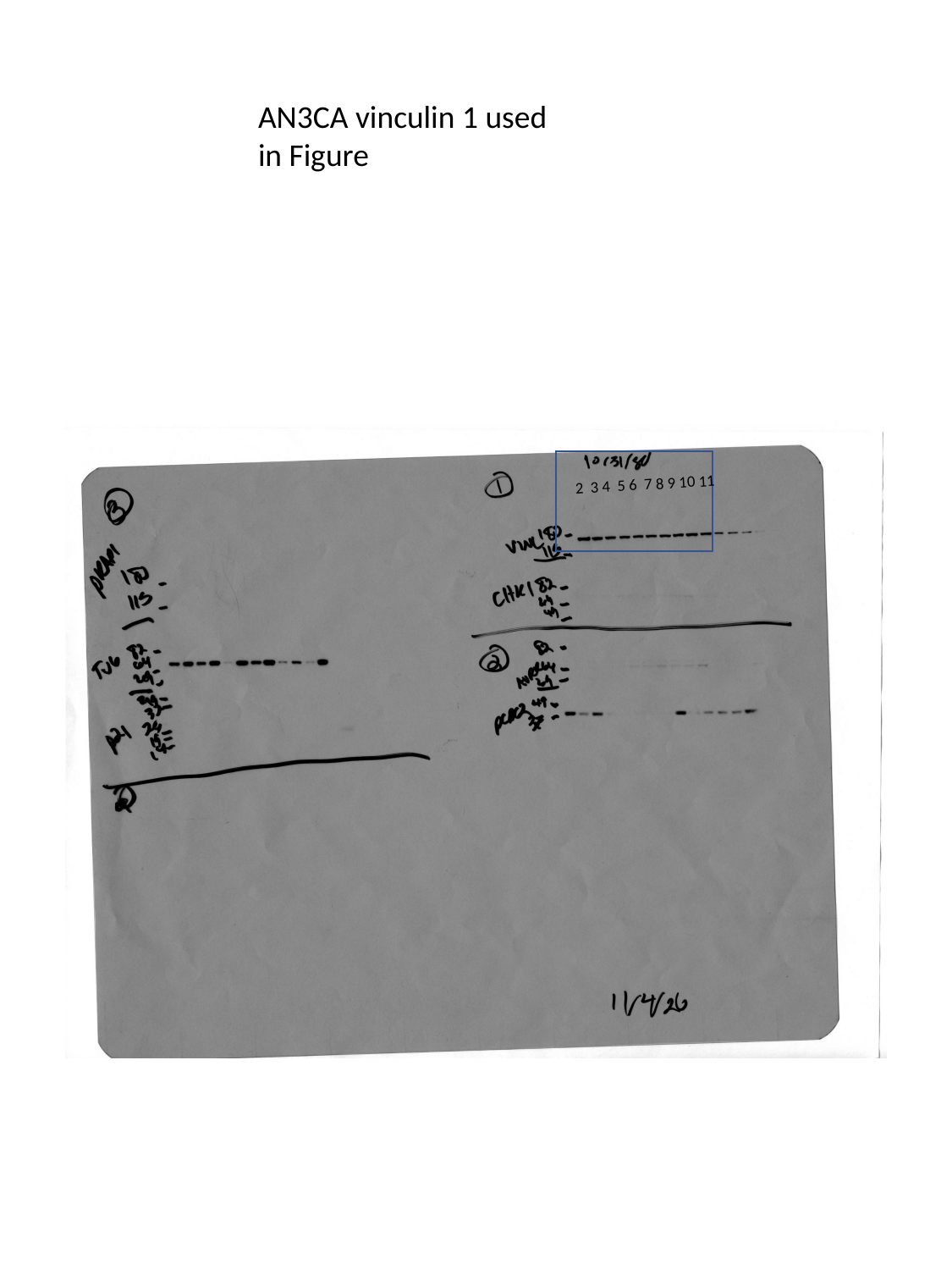

AN3CA vinculin 1 used in Figure
2 3 4 5 6 7 8 9 10 11

## Slide 31
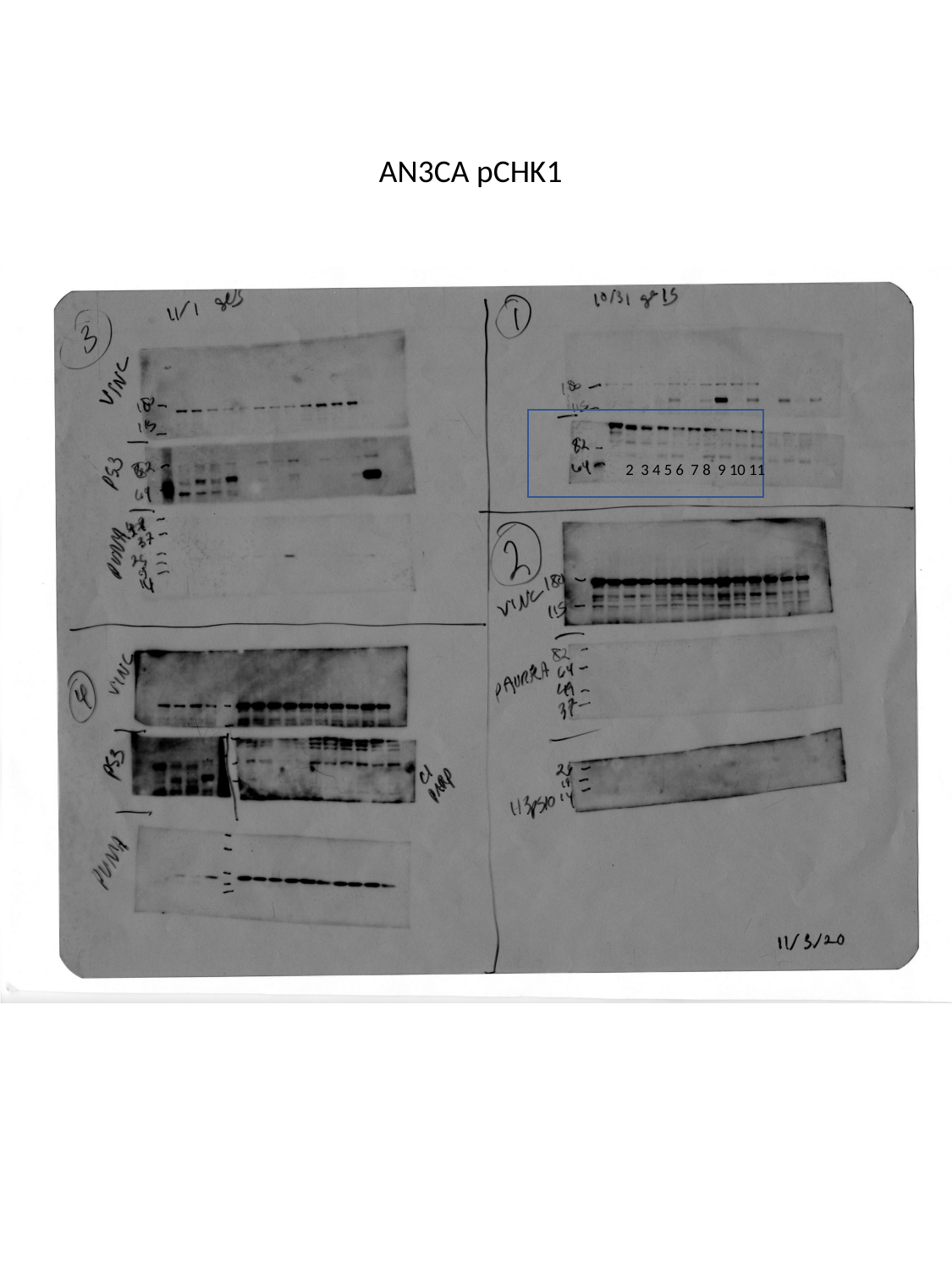

AN3CA pCHK1
2 3 4 5 6 7 8 9 10 11

## Slide 32
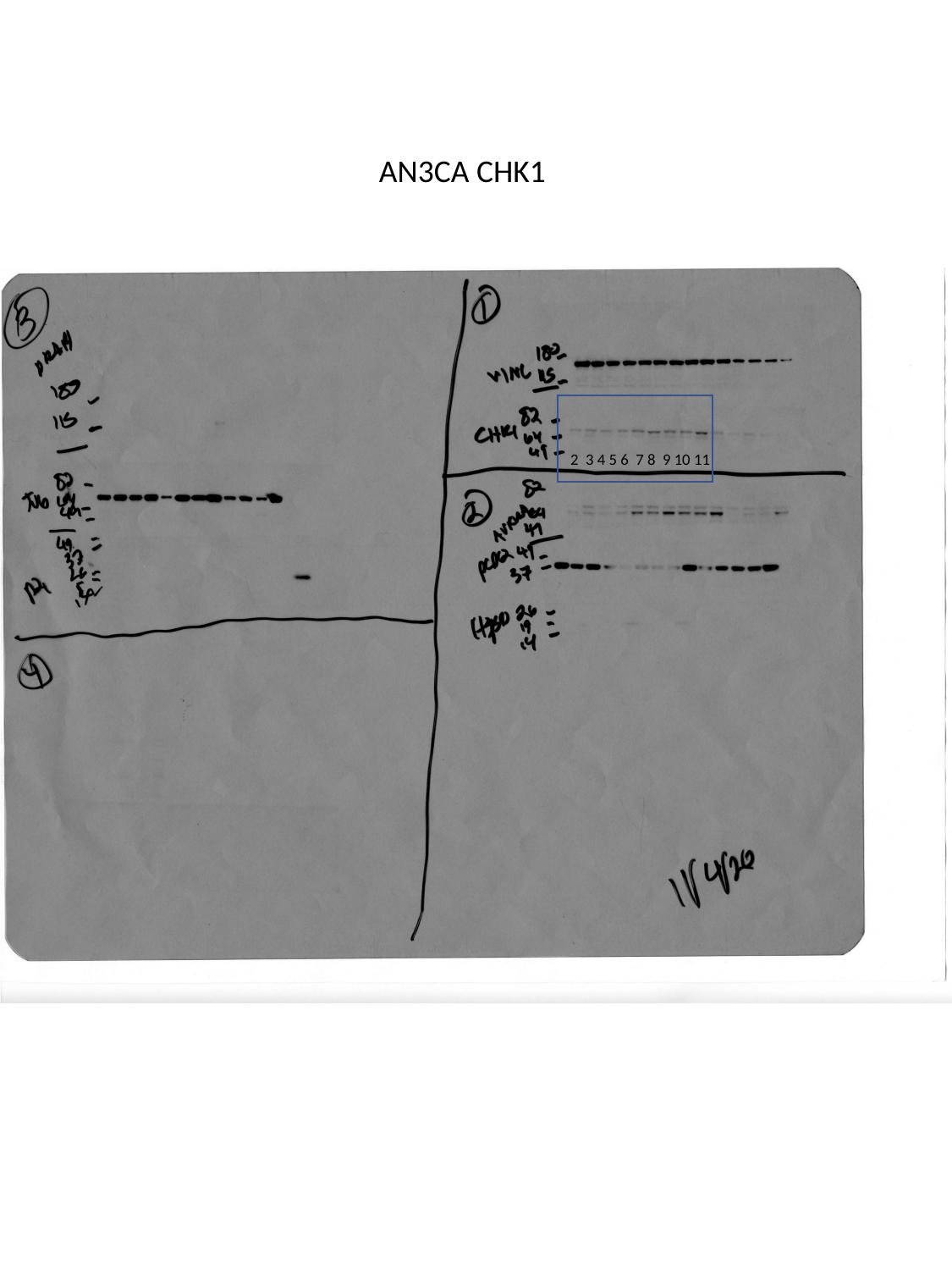

AN3CA CHK1
2 3 4 5 6 7 8 9 10 11

## Slide 33
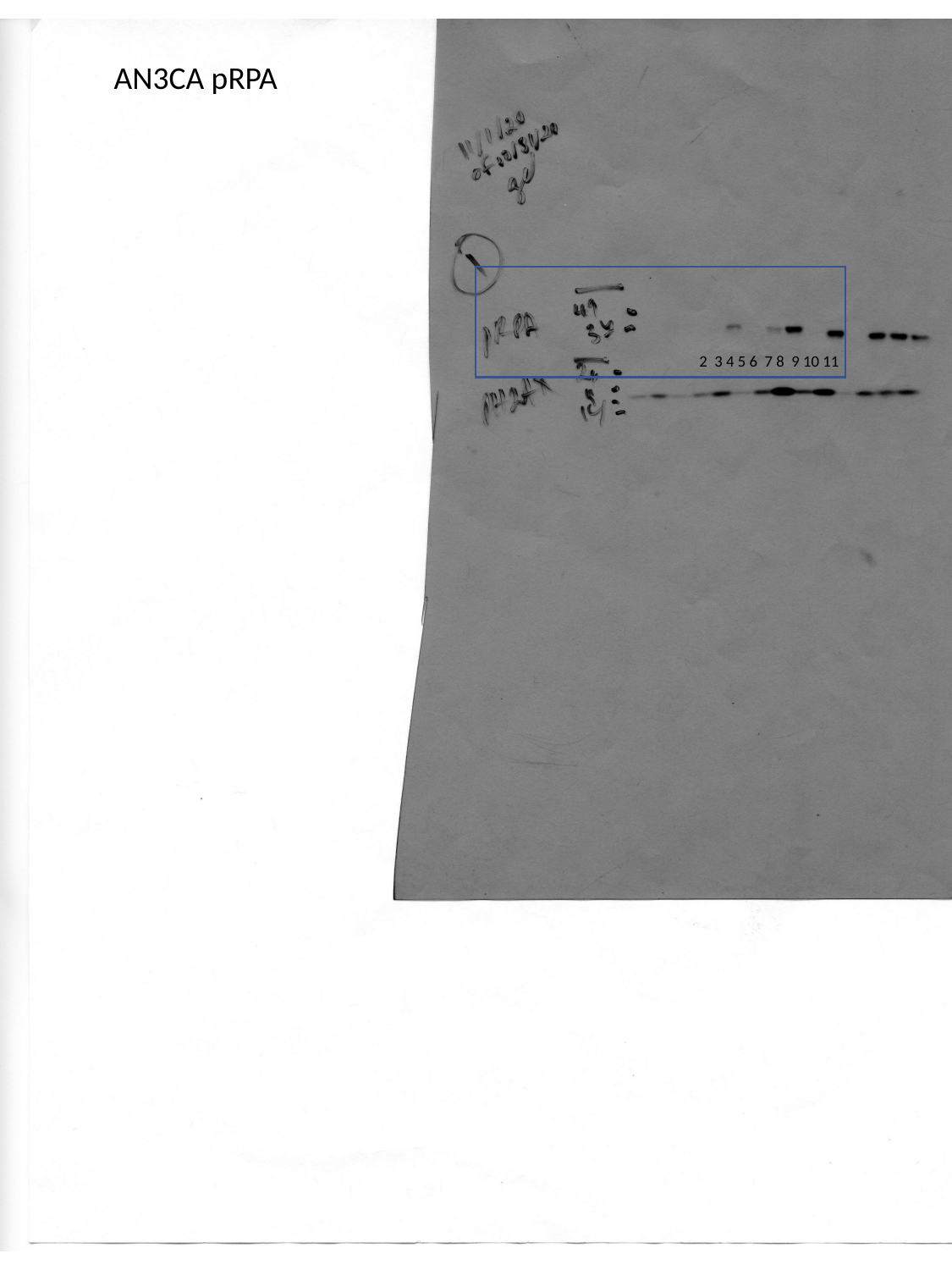

AN3CA pRPA
2 3 4 5 6 7 8 9 10 11

## Slide 34
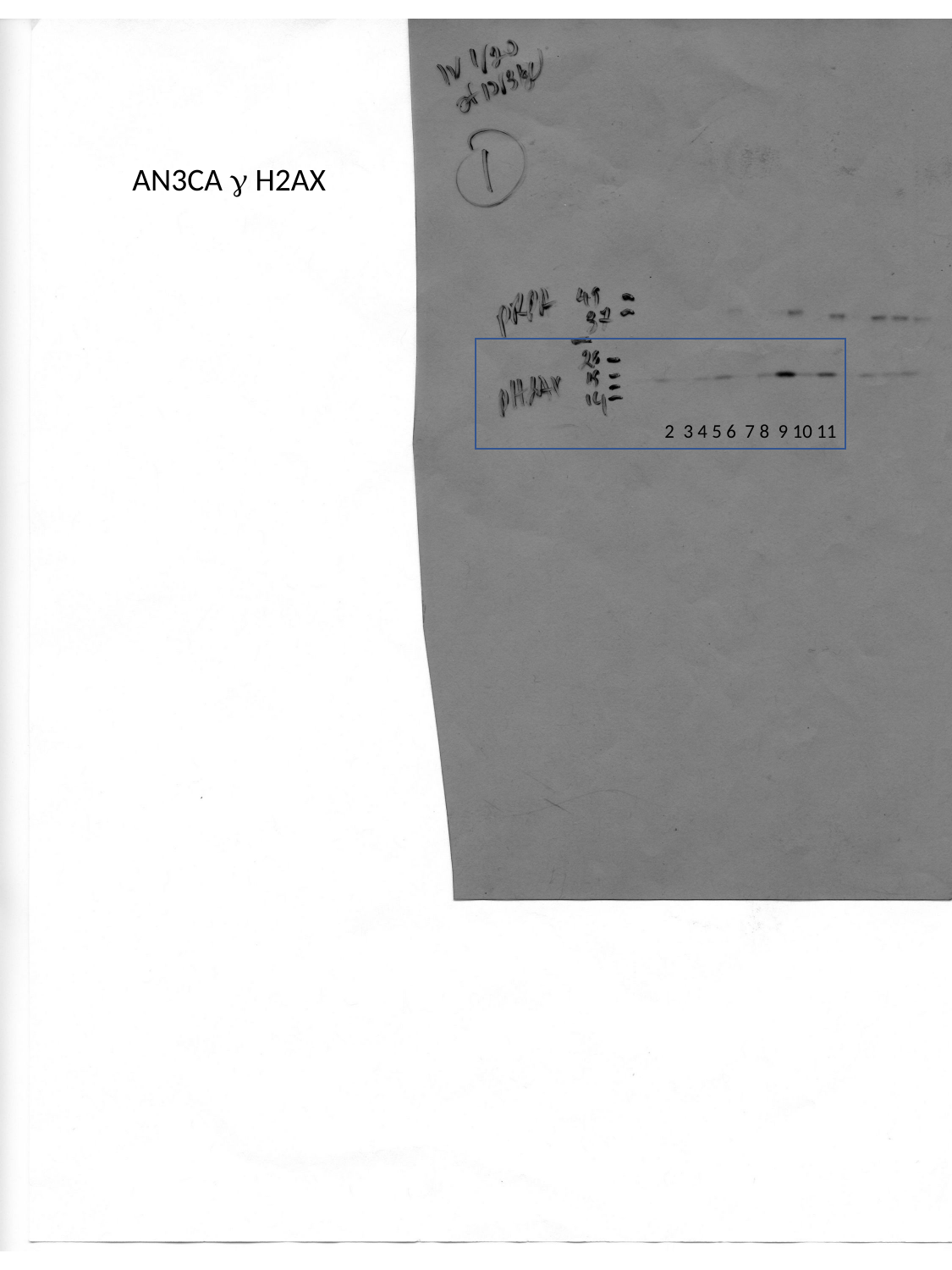

AN3CA γ H2AX
2 3 4 5 6 7 8 9 10 11

## Slide 35
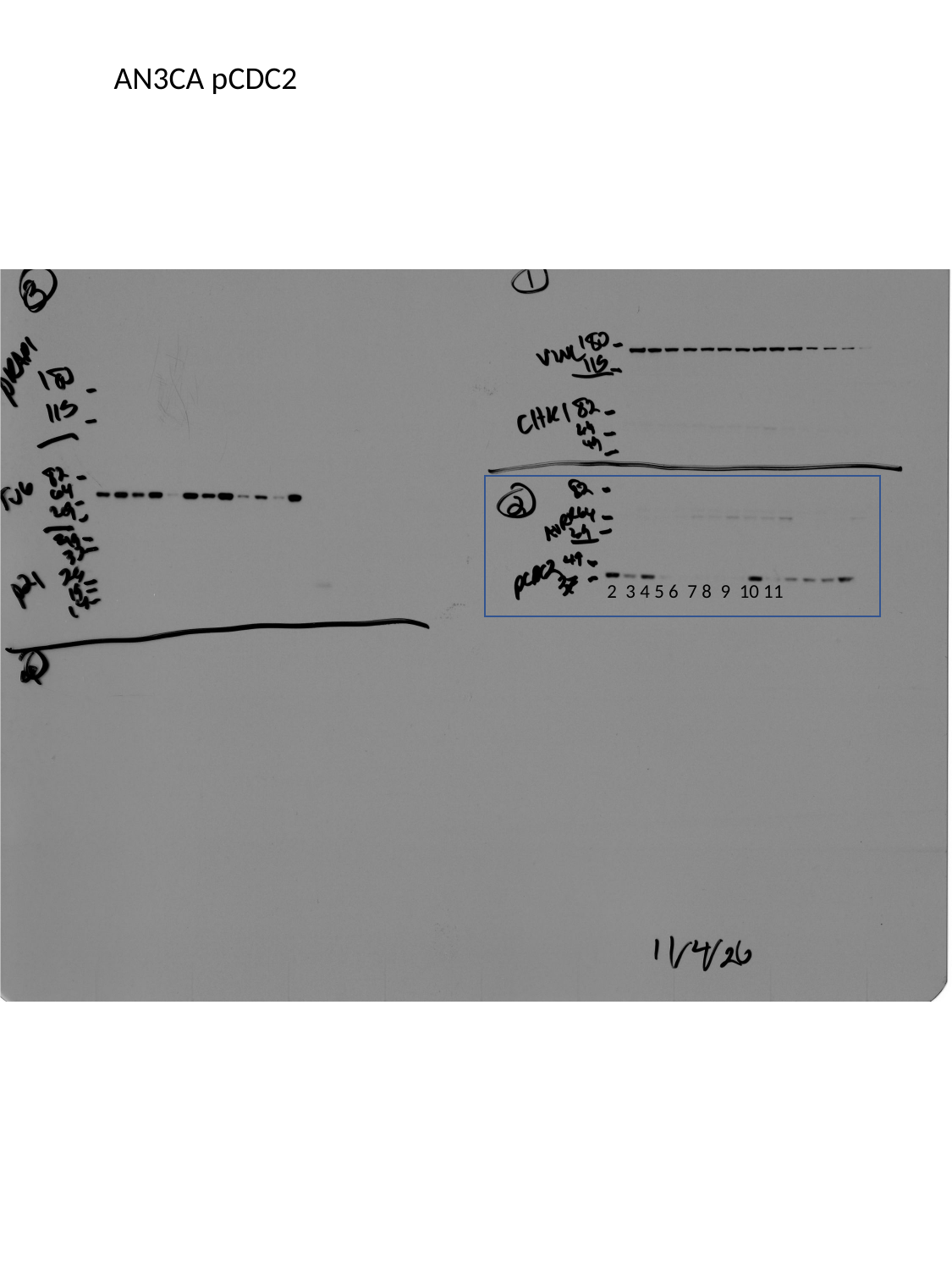

AN3CA pCDC2
2 3 4 5 6 7 8 9 10 11

## Slide 36
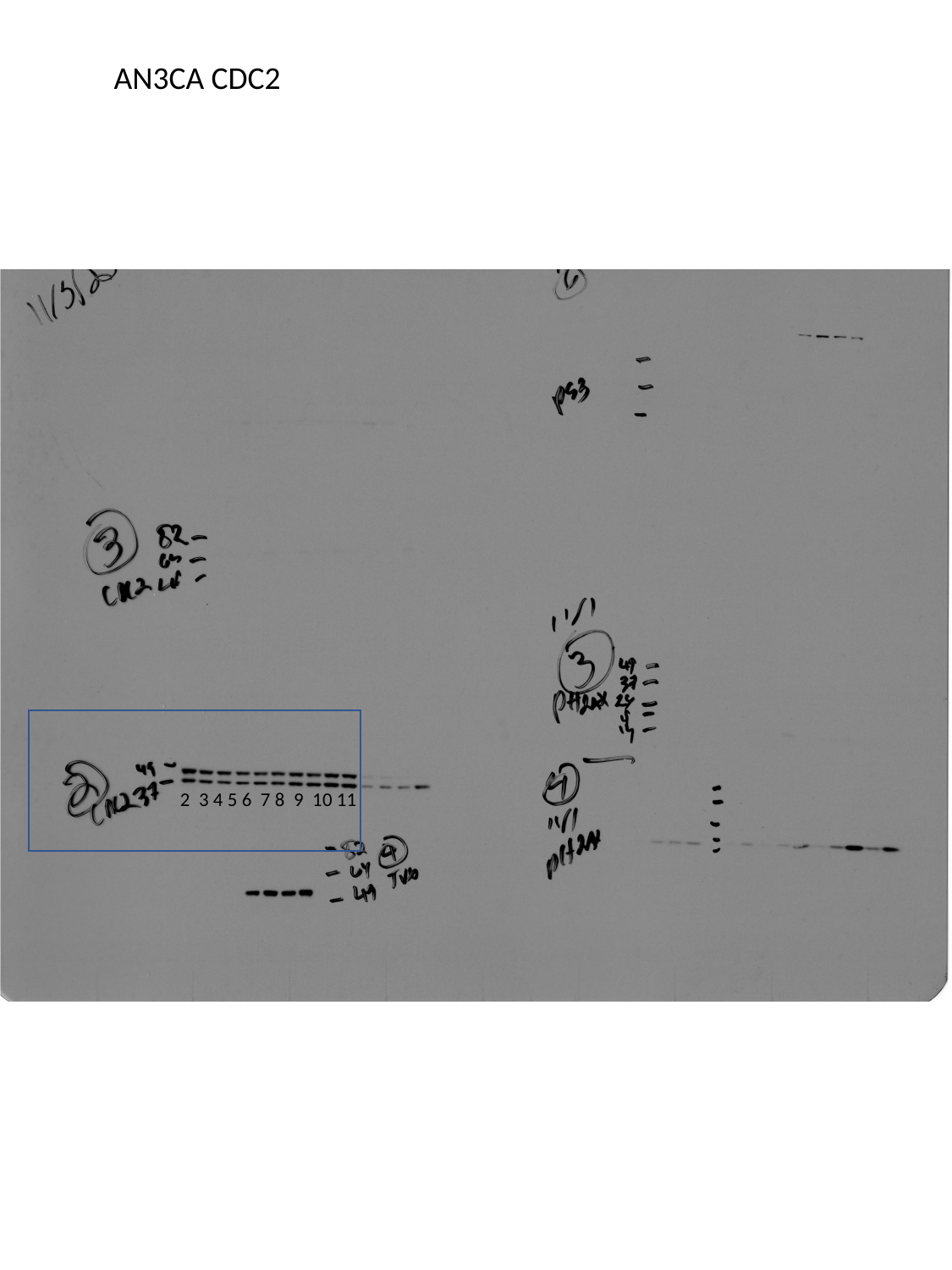

AN3CA CDC2
2 3 4 5 6 7 8 9 10 11

## Slide 37
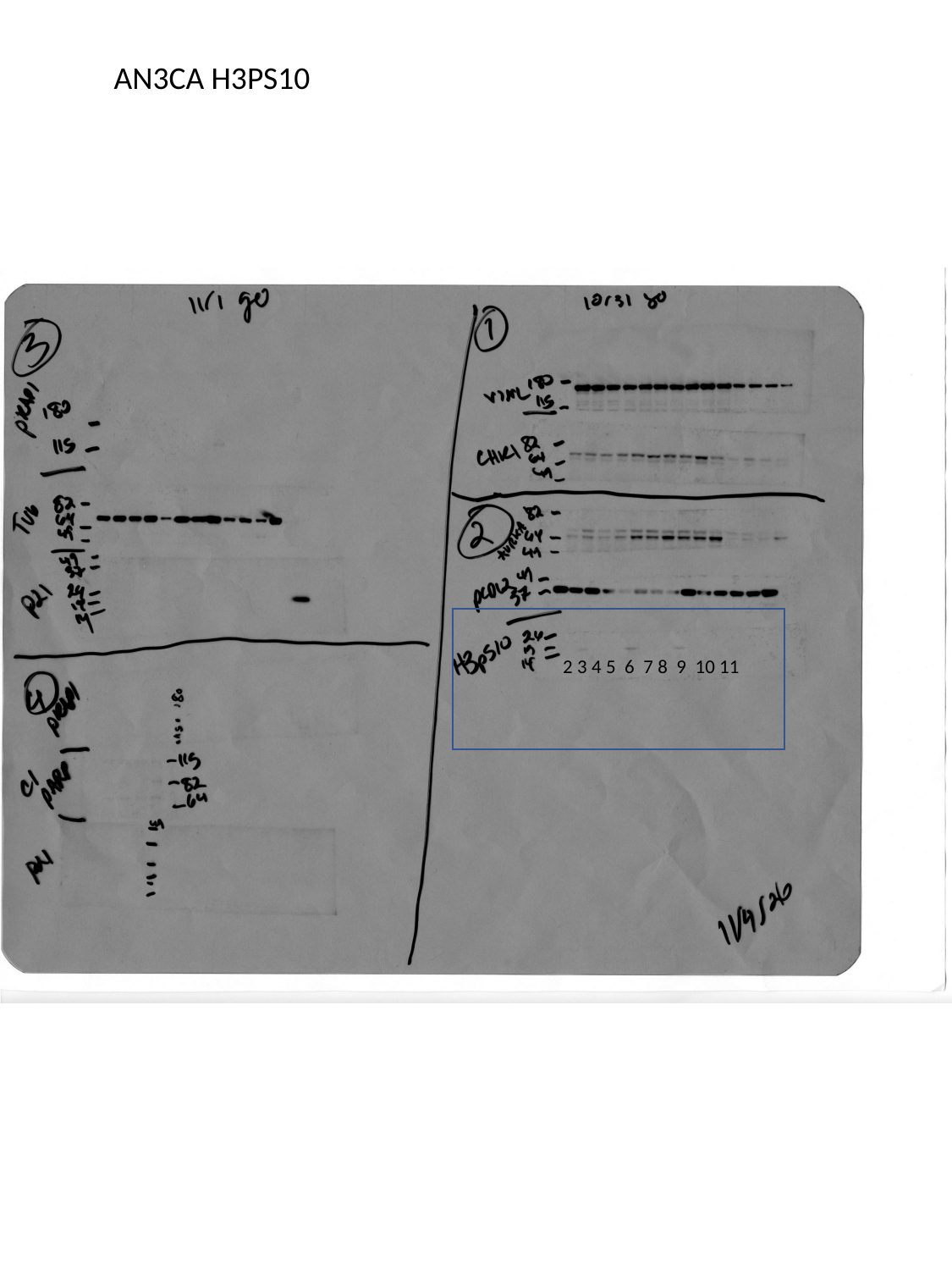

AN3CA H3PS10
2 3 4 5 6 7 8 9 10 11
