# Supplementary material for: Enhanced Efficacy of Aurora Kinase Inhibitors in G2/M Checkpoint Deficient TP53 Mutant Uterine Carcinomas Is Linked to the Summation of LKB1–AKT–p53 Interactions
Source: Cancers (Basel). 2021 May 3;13(9):2195. doi: 10.3390/cancers13092195 (PMC8125555; doi:10.3390/cancers13092195)
Supplement: Supplementary file 1 [file cancers-13-02195-s001.zip › Lynch and Hill Supplementary Matierals/original blot/Figure S7C.pptx]

## Slide 1
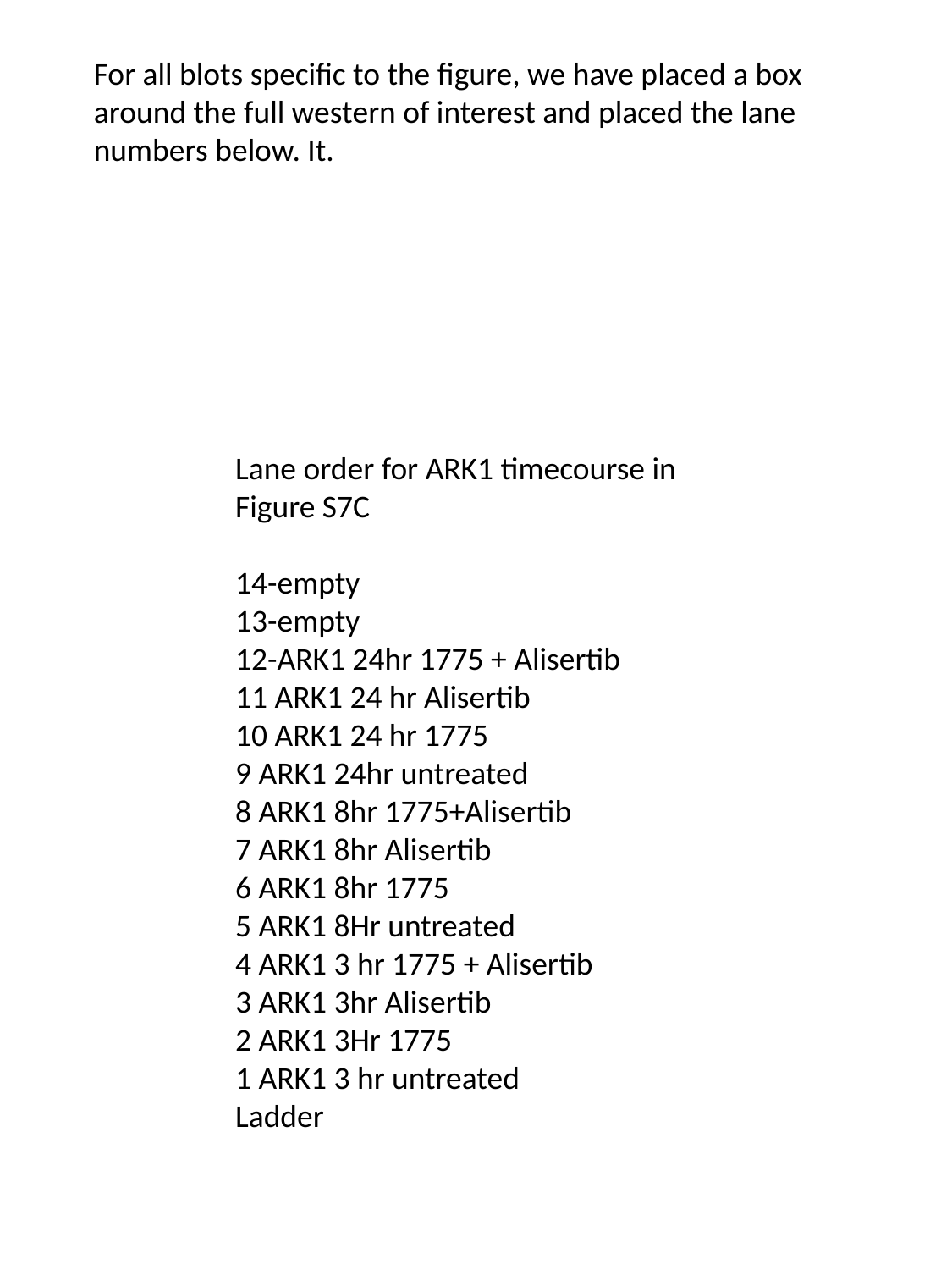

For all blots specific to the figure, we have placed a box around the full western of interest and placed the lane numbers below. It.
Lane order for ARK1 timecourse in Figure S7C
14-empty
13-empty
12-ARK1 24hr 1775 + Alisertib
11 ARK1 24 hr Alisertib
10 ARK1 24 hr 1775
9 ARK1 24hr untreated
8 ARK1 8hr 1775+Alisertib
7 ARK1 8hr Alisertib
6 ARK1 8hr 1775
5 ARK1 8Hr untreated
4 ARK1 3 hr 1775 + Alisertib
3 ARK1 3hr Alisertib
2 ARK1 3Hr 1775
1 ARK1 3 hr untreated
Ladder

## Slide 2
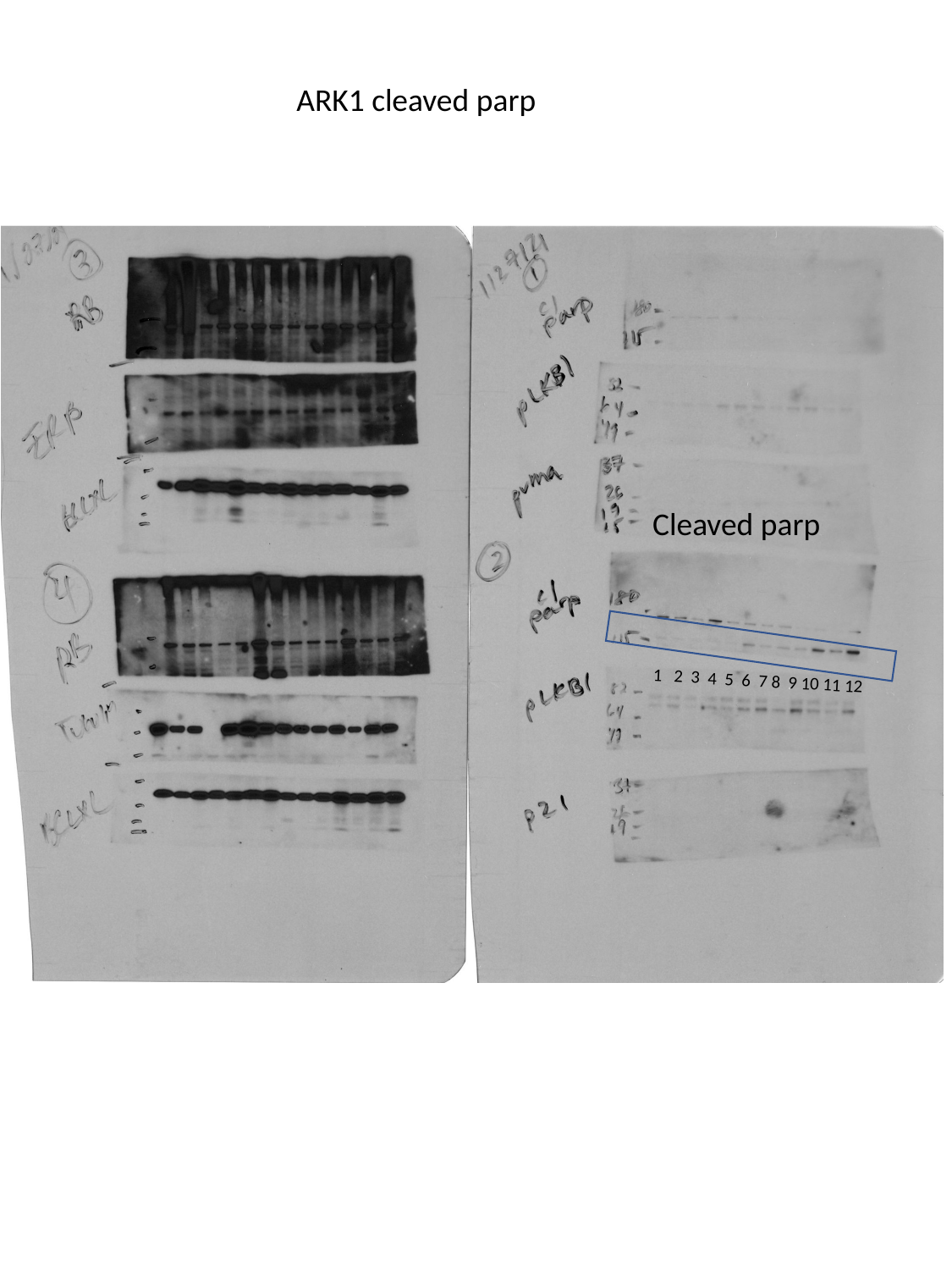

ARK1 cleaved parp
Cleaved parp
1 2 3 4 5 6 7 8 9 10 11 12

## Slide 3
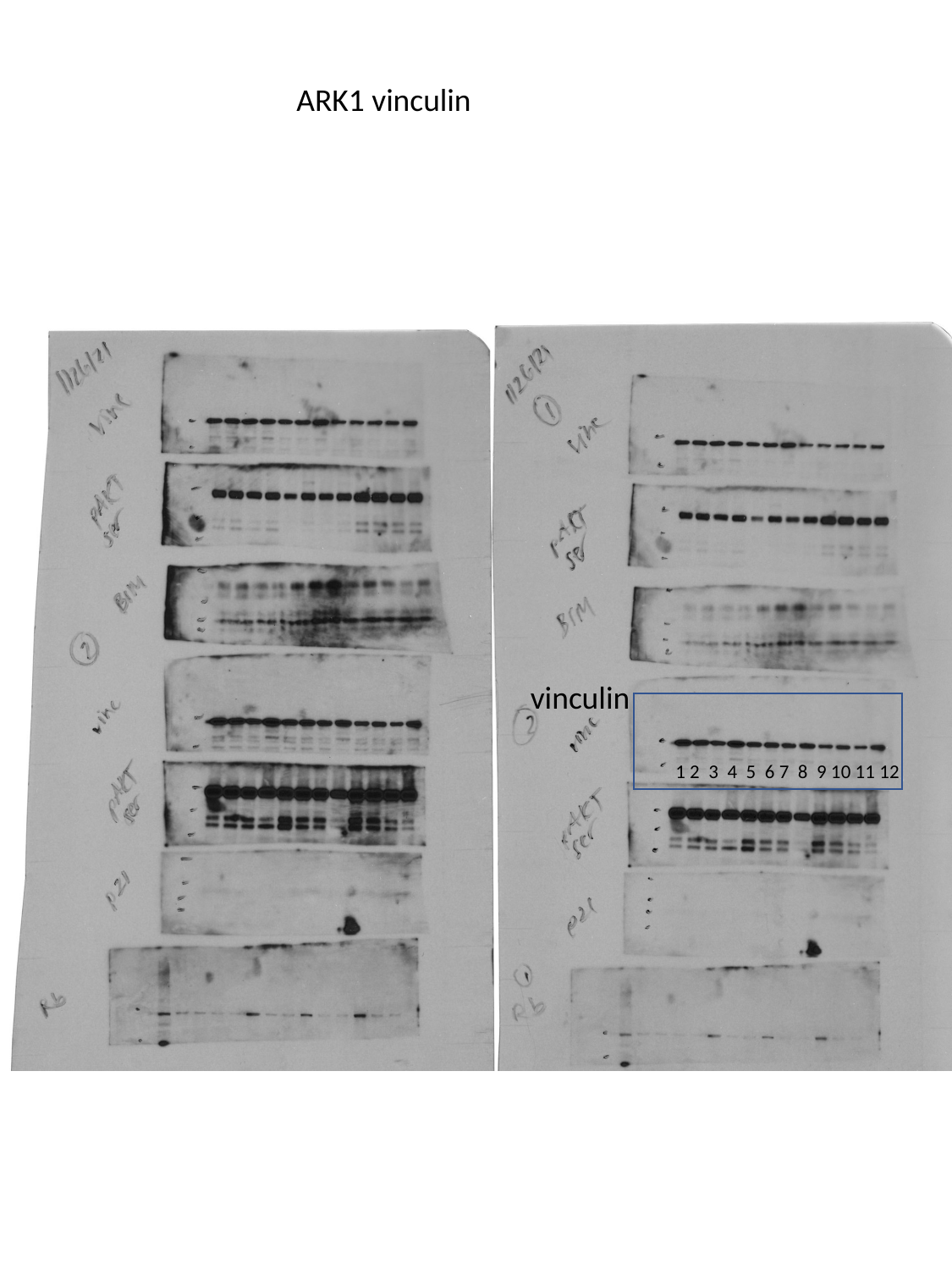

ARK1 vinculin
vinculin
1 2 3 4 5 6 7 8 9 10 11 12
